# Human fetal mesoangioblasts reveal tissue‐dependent transcriptional signatures

**DOI:** 10.1002/sctm.19-0209

**Published:** 2020-01-23

**Authors:** Flavio L. Ronzoni, Sylvain Lemeille, Rostyslav Kuzyakiv, Maurilio Sampaolesi, Marisa E. Jaconi

**Affiliations:** ^1^ Department of Pathology and Immunology, Faculty of Medicine University of Geneva Geneva Switzerland; ^2^ Stem Cell Institute KU Leuven Leuven Belgium; ^3^ Department of Public Health, Forensic and Experimental Medicine University of Pavia Pavia Italy; ^4^ Center for Health Technologies (CHT) University of Pavia Pavia Italy

**Keywords:** cardiogenesis, fetal stem/progenitor cells, mesoangioblasts, myogenesis, transcription factors, transcriptome analysis

## Abstract

Mesoangioblasts (MABs) derived from adult skeletal muscles are well‐studied adult stem/progenitor cells that already entered clinical trials for muscle regeneration in genetic diseases; however, the transcriptional identity of human fetal MABs (fMABs) remains largely unknown. Herein we analyzed the transcriptome of MABs isolated according to canonical markers from fetal atrium, ventricle, aorta, and skeletal muscles (from 9.5 to 13 weeks of age) to uncover specific gene signatures correlating with their peculiar myogenic differentiation properties inherent to their tissue of origin. RNA‐seq analysis revealed for the first time that human MABs from fetal aorta, cardiac (atrial and ventricular), and skeletal muscles display subsets of differentially expressed genes likely representing distinct expression signatures indicative of their original tissue. Identified GO biological processes and KEGG pathways likely account for their distinct differentiation outcomes and provide a set of critical genes possibly predicting future specific differentiation outcomes. This study reveals novel information regarding the potential of human fMABs that may help to improve specific differentiation outcomes relevant for therapeutic muscle regeneration.

## Graphical abstract

RNAseq analysis of human foetal mesoangioblasts (MABs) derived from atrium, ventricle, aorta and skeletal muscle uncovers specific gene signatures correlating with their peculiar myogenic differentiation properties inherent to their tissue of origin. Bioinformatics analysis provides a set of critical genes predicting their future specific differentiation outcomes relevant for therapeutic muscle regeneration.

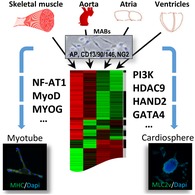

**Significance statement**

The current study reveals transcriptional identities in human fetal mesoangioblasts (fMABs) from aorta, cardiac, and skeletal muscle tissues, with specific gene signatures correlating with their peculiar myogenic differentiation properties inherent to their derivative tissues. Gene network analysis identified four major superclusters of differentially expressed genes and uncovered a global opposite set of upregulated and downregulated genes between skeletal and cardiac muscle MABs, with the ones from aorta showing an intermediate profile. Collectively, the current work provides a set of critical genes accounting of, and possibly predicting, lineage‐specific differentiation commitments during development. This knowledge may help improve the future management of muscle regeneration.

## 1 INTRODUCTION

Mesoangioblasts (MABs) are characterized as a subpopulation of pericytes or vessel‐associated stem/progenitor cells capable to self‐renew and differentiate into several mesoderm cell types, including skeletal and cardiac muscle.^1^, ^2^ Originally isolated from the mouse embryonic dorsal aorta (Ao), MABs can be obtained from postnatal vessels of skeletal muscle (Sk) and heart of larger mammalian species, including humans^3^, ^4^, ^5^, ^6^ and expanded in culture for up to 20‐25 passages before undergoing senescence. Although skeletal MABs can significantly restore muscle structure and function, thus ameliorating symptoms in dystrophic mice and dogs,^7^, ^8^ their functional efficacy in clinical trials remain an unmet challenge,^9^ with issues of engraftment, migration, and cell fusion efficiencies.^10^ On the other hand, preclinical studies indicated that murine cardiac MABs delivered into an ischemic heart ventricle end up locating at the periphery of the necrotic area and seem to differentiate into cardiomyocytes (CMs), possibly participating in the myocardial regeneration.^9^ Similarly, MABs associated to Ao could also differentiate into cardiac and endothelial cells when transplanted in murine dystrophic hearts, thereby preventing the onset of dilated cardiomyopathy.^11^ Although more prone to senescence and less efficient in vivo than other resident cardiac stem cell types, cardiomyogenic MABs may constitute an interesting and plastic reservoir, given their relative abundance around the rich microvasculature network bedewing the cardiac muscle. So far, however, the clinical potential of human MABs from heart or other tissues remains unexplored.

In vitro studies have shown that rodent or human postnatal Sk‐MABs efficiently differentiate into skeletal myotubes upon serum reduction (2%).^7^, ^12^ Notably, Sampaolesi et al showed that the intra‐arterial delivery of normal or dystrophic genetically corrected MABs to α‐sarcoglycan KO mice modeling Limb‐Girdle muscular dystrophy produced a dramatic functional amelioration of the dystrophic phenotype,^7^ thanks to their enhanced migration and homing potential compared with satellite cells, even if treated with recombinant proteins.^13^, ^14^ In contrast, murine, canine, and human cardiac MABs resulting positive for pericytic markers (eg, NG2 and alkaline phosphatase [AP]) and cardiac transcription factors (TFs) such as Nkx2.5 and Gata4 rather differentiate into cardiac cells only when co‐cultured with rodent neonatal CMs^15^, ^16^ or upon treatment with 5‐azacytidine,^12^, ^17^ and with a variable efficiency according to the tissue source.^17^ In those culture conditions, only low‐passage murine cardiac MABs could differentiate into beating CMs presenting sarcomeric structures and Cx43 junctions, although with unclear efficiency.^18^ Remarkably instead, human cardiac MABs seem unable to contract in vitro, and when isolated from samples of human cardiomyopathic hearts, they presented impairment of several markers of proliferation and plasticity.^16^ This questions their therapeutic effect as such, unless precise treatment cues are set up to unleash and/or maximize their differentiation before or upon transplantation. In this view, a better understanding of the MAB cellular identity among different tissues is clearly mandatory and will be instructive to uncover actionable signatures capable of coaxing stem cell differentiation.

Notably, nothing is currently known about the differentiation potential of MABs isolated from human fetal tissues and how they compare with postnatal/adult cells. In particular, it is presently not possible to acknowledge if intra‐individual human MABs derived from different tissues display molecular signatures peculiar of their tissue niche and anticipating their differentiation potential. This knowledge could clarify whether cardiac MABs may be the most appropriate stem/progenitor cell source for the regeneration of the myocardium, or whether MABs derived from skeletal or Ao tissues could also efficiently provide cardiac derivatives in addition to the preferred Sk fate. Recent controversies have questioned the real existence of cardiac stem or progenitor cells highlighting the lack of reproducibility of isolation methods and specific marker identification. In this respect, our whole transcriptome study of heart progenitor cells may clarify MABs as stem/progenitor cells particularly suitable for cardiac therapeutic approaches.

The major goal of this study is to uncover the transcriptomic identity of side by side compared human fMABs originating for heart, Ao, and Sk muscles. Such information is fundamental to understand the observed distinctive differentiation propensities according to origin, in particular skeletal vs cardiac muscle lineage identity and will be key for a future efficient generation of the most suitable cell source for muscle tissue regeneration. In particular, as dilated cardiomyopathy is a leading cause of chronic morbidity and mortality in muscular dystrophy patients, grasping the cardiogenic potential of human MAB cell sources, possibly extracardiac for obvious reasons of accessibility, would be of great interest for regenerative medicine purposes.

## 2 MATERIALS AND METHODS

### 2.1 Tissue sample collection

Fetal tissue samples were obtained from an aborted material of gestational age between 9.5 and 13 weeks and donated to research under informed consent according to authorization #02‐088 (Gyn 02‐007) delivered by the Central Ethics Committee of the Geneva University Hospitals. Independent genetically matched samples were harvested for each fetal donor and processed separately.

### 2.2 Cell isolation and culture

Human fetal mesoangioblasts (fMABs) were isolated from Ao, cardiac (atrium [At] and ventricle [V]), and Sk muscle fragments as previously described.^18^ Sk and cardiac fragments were rinsed in phosphate‐buffered saline (PBS) (w/o Ca^2+^ Mg ^2+^), cut into very small pieces (1‐2 mm diameter) and transferred to a Petri dish coated with type I collagen (Sigma‐Aldrich) and 1% gelatin (Sigma‐Aldrich). The medium consisted of MegaCell DMEM (Sigma‐Aldrich) supplemented with 5% fetal bovine serum (FBS, Lonza BioWhittaker), 5 ng/mL basic fibroblast growth factor (bFGF, R&D Systems), 2 mM l‐glutamine, 0.1 mM β‐mercaptoethanol, 1% nonessential amino acids, and 1% penicillin/streptomycin (all from Gibco, Invitrogen). The tissue fragments were cultured for 7‐10 days, and after the initial outgrowth of fibroblast‐like cells, small, round, and reflective cells could be observed. fMAB cultures were maintained at 5000 cells per cm^2^ in a 5.5% CO_2_ humidified incubator under hypoxic conditions (5% O_2_) and were split every 2‐3 days.

### 2.3 FACS sorting and analysis

Cells were detached with 0.05% trypsin‐EDTA and washed with PBS containing 3% FBS. After washing, 2‐3 × 10^6^ cells were incubated for 20 minutes in the dark at 4°C with conjugated mouse anti‐human antibodies or isotype controls (1 μg/mL) as follows: PE‐Cy7‐conjugated anti‐AP (R&D Systems), FITC‐conjugated anti‐CD90 (BD‐Pharmingen), APC‐conjugated anti‐CD13 (e‐Bioscience), Alexa Fluor 647‐conjugated anti‐NG2, PE‐Cy7‐conjugated anti‐CD146 (BD‐Pharmingen), Alexa Fluor 488 anti‐CD56 (BD‐Pharmingen). 7‐AAD (Thermofisher Scientific) was used as a marker to exclude dead cells from the analysis. All cells were sorted and/or analyzed using an FACS Astrios flow cytometer (BD Biosciences) with at least 10 000 recorded events. Data were analyzed with FlowJo software (Tree Star, Ashland, Oregon).

### 2.4 Cell characterization and differentiation potentials

#### 2.4.1 *Immunocytochemistry*

For immunofluorescence staining, cells were fixed with 4% paraformaldehyde for 10 minutes at room temperature (RT). After permeabilization with 0.2% Triton‐X‐100 in PBS, nonspecific binding of antibodies was blocked by incubating cells in PBS + 5% serum from the species in which secondary antibodies were raised. Cells were kept overnight at RT with specific primary antibodies (1 μg/mL; anti‐alpha‐actinin (Sigma), anti‐MLC2v (BD Pharmingen) anti‐MyoD (SantaCruz), anti‐MHC (Hybridoma Bank). The next day, cells were washed with PBS and blocking buffer, and then incubated with specific fluorescent‐labeled secondary Alexa Fluor‐conjugated antibodies (Invitrogen) at a dilution of 1:1000 for 45 minutes at RT in the dark. Hoechst 33258 or DAPI (Sigma‐Aldrich) were used to identify cell nuclei (1:1000 dilution). Unbound antibodies were washed away with PBS and 0.1% Tween 20 in PBS, and samples were then mounted with Mowiol (Sigma‐Aldrich) before their analysis under a Nikon Ar1 spectral or a confocal Zeiss LSM700 microscope.

AP staining was performed using BM purple (Roche) according to the kit instructions.

#### 2.4.2 *RNA extraction, RT‐PCR, and real‐time PCR (qPCR)*

Total RNA was extracted and purified after DNase treatment (Quiagen) using the RNAeasy kit (Quiagen). The RNA concentration of each sample was determined by NanoDrop 2000 (Thermo Scientific). cDNAs obtained from 100 ng of RNA were amplified using the M‐MLV Reverse Transcriptase kit (Promega), and q‐PCR was performed using a StepOnePlus system (Thermofisher Scientific).

#### 2.4.3 *RNA‐seq: Library preparation, sequencing, and reads mapping to the reference genome*

cDNA libraries were constructed by the Genomics Platform of the Geneva Faculty of Medicine using the Illumina TruSeq RNA Sample Preparation Kit according to the manufacturer's protocol (Illumina). Libraries were sequenced using single reads (50 nt‐long) on an Illumina HiSeq2000. FastQ reads were mapped to the ENSEMBL reference genome (GRCh38.76) using Bowtie^19^ with standard settings, except that any reads mapping to more than one location in the genome (ambiguous reads) were discarded (m = 1). Sequence data were submitted to the GEO database under the accession number GSE90069.

#### 2.4.4 *Unique gene model construction and gene coverage reporting*

We used a unique gene model to quantify reads per gene. Briefly, the model considers all annotated exons of all annotated protein coding isoforms of a gene to create a unique gene where the genomic region of all exons are considered coming from the same RNA molecule and merged together.

#### 2.4.5 *RNA‐seq analysis*

All reads reporting the exons of each unique gene model were reported using BEDtools.^20^ Library size normalizations and differential gene expression calculations were performed using the package edgeR^21^ designed for the R software.^22^ Only genes having a significant fold‐change (*P*‐value ≤.001) were considered for the rest of the RNA‐seq analysis.

#### 2.4.6 *Gene Ontology and KEGG analysis*

GO term (http://www.geneontology.org) and KEGG metabolic pathways enrichment (https://www.genome.jp/kegg/pathway.html) was performed using homemade scripts for the R software.^22^ Enriched Biological Processes were sorted into five categories of interest: Cardiac Development, Angiogenesis, Cell Adhesion, Skeletal Muscle Development and General Terms. Biological Processes having a *P*‐value (significance of the enrichment) lower than .05 and an odds ratio (enrichment value) higher than 2 in at least one supercluster, were considered for the analysis. If the *P*‐value was higher than .05 or the odds ratio of a Biological Process was lower than two in a supercluster, than the enrichment value for this Biological Process in this supercluster was set to zero. When an odds ratio was infinite (in case all the genes involved in a particular Biological Process are present in a supercluster), then the enrichment value was set to 150.

Each Biological Process term was always plotted with the same angle, the radius corresponding to the enrichment value (in log2(1 + odds ratio) scale, with the maximum radius equivalent to log2(151)) for this term in the supercluster.

#### 2.4.7 *Software analysis*

We used the MetaCore software (version 6.29, Thomson Reuters) to compare the relative gene expression levels of each gene using an algorithm recognizing common or unique lineage enrichment patterns to which genes belong. Statistical significance of gene expression set with a fold change −3 < FC > 3 and *P* < .05.

## 3 RESULTS

### 3.1 Isolation and characterization of human fMABs from different tissues

**Figure 1:**
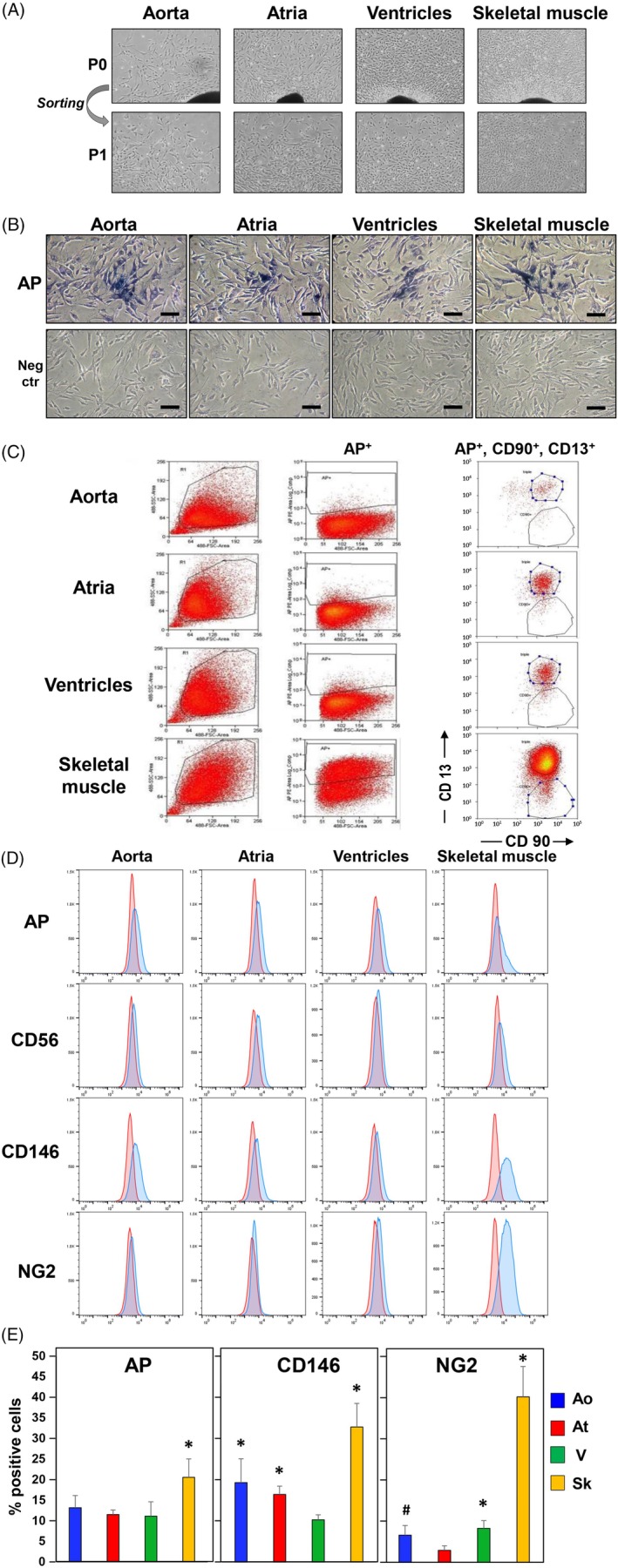
Isolation, sorting, and marker characterization of human fetal mesoangioblasts (fMABs) from different tissue sources: aorta, atrium, ventricle, and skeletal muscle. A, fMABs migrate out of tissue fragments over 1‐week culture (black spots) and show a comparable cell morphology, both before and after sorting using surface markers such as alkaline phosphatase (AP), CD13, and CD90. B, AP staining of the four different fMAB populations after sorting. Scale bar: 100 μM. Neg ctr, negative control. C and D, Sorting and FACS analysis of fMAB surface markers AP, CD56, CD146, and NG2 (neuron/glial type 2 antigen). E, Percentage of cells positive for AP, CD146, and NG2 determined by FACS. *P < .01; ^#^ *P* < .05

We isolated fMABs from fragments of genetically‐matched Ao, atria, V, and Sk from several donor individuals (N = 4) ranging from 9.5 to 13 weeks of gestation according to previously published protocols.^4^, ^5^, ^15^, ^19^ Specifically, fMABs were sorted for AP, CD90, and CD13. We fully characterized the two closest age‐matched samples in their exponential proliferation phase at passage 2, to minimize bias due to sex and age variations. Figure 1A illustrates the typical morphology of cells migrating out of the tissue clumps in culture at passage 0 and after sorting at passage 1. Overall, human fMABs derived from the four tissue types did not show any gross morphological differences and presented a normal euploid karyotype (Figure 1A). Cells were small, triangular (Figure 1A), and resulted all positive for AP staining (Figure 1B). As previously reported for postnatal MABs,^5^ fMABs could proliferate for up to 20‐25 passages before entering senescence, and their population doublings were similar up to day 10 in culture. Thereafter, At‐MABs showed a slightly slower population‐doubling rate compared with the other MABs (Figure S1B). In addition, fMABs expressed the typical MAB surface markers although the numbers of positive cells were quite different between the four tissues of origin (Figure 1C,D,E). In particular, fetal skeletal MABs (Sk‐fMABs) expressed higher levels of AP, CD146, and the pericyte marker NG2, when compared with Ao and At, or V fMABs (Figure 1C,D,E). As expected, all fMABs were negative for hematopoietic cell surface proteins (CD34, CD45, data not shown) and for CD56, although could be sorted as AP‐, CD13‐, and CD90‐positive cells from the four fetal sources (see Figure 1C,D).

### 3.2 Human fMABs can differentiate into adipocytes, chondrocytes, osteocytes, and smooth muscle cells

To ascertain the differentiation potential toward adipogenic, osteogenic, and chondrogenic fates, human fMABs were differentiated using the standard kit protocols (Thermo Fisher Scientific). Typically, adipogenic differentiation occurred after 2 weeks of adipogenic induction and cells contained fat vacuoles that stained positive with oil red O solution (Figure S2A) and displayed a significant upregulation of the adipogenic‐specific transcripts such as fatty acid‐binding protein 4 (*FABP4*) and perilipin 1 (*PLIN1*) as detected by qPCR (Figure S2B). Upon day 28 of differentiation toward osteoblasts, calcium deposits were stained with Alizarin red solution (Figure S2A) and a significant increase in alkaline phosphatase (*ALPL*) and secreted protein acidic and cysteine rich (SPARC) expression was evident in all four populations (Figure S2B). Upon 21 days of induced chondrogenic differentiation, cells stained positive for proteoglycans and glycosaminoglycans using Safranin. Comparable qualitative staining patterns were seen in all fMABs (Figure S2A), with an increase of chondrogenic aggrecan (*ACAN*) and collagen type II alpha (*COL2a*) transcripts (Figure S2B). A consistent increase in the smooth muscle marker calponin 1 (*CNN1*) was obtained by stimulating cells with TGFβ (5 ng/mL) for 7 days (Figure S2B).

**Figure 2:**
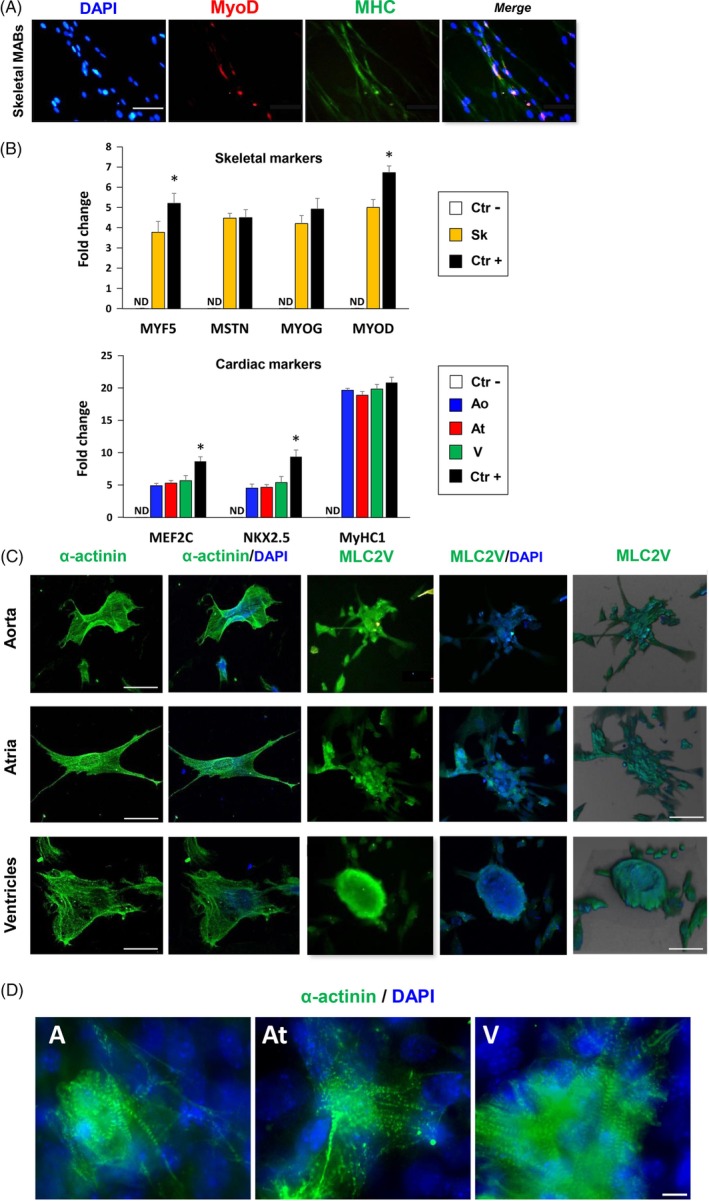
Tissue‐specific marker analysis of the different fetal mesoangioblast (fMAB) populations upon skeletal or cardiac muscle differentiation in culture. A, Typical immunofluorescence staining of myotube formation in differentiating Sk‐fMABs in low serum (2%). Nuclei are counterstained with DAPI (blue). B, q‐PCR analysis of skeletal (upper panel) and cardiac (lower panel) marker expression in the different fMAB populations. Human foreskin fibroblasts were used as negative control (Ctr−), whereas human satellite cells and human adult cardiomyocytes were used as positive controls (Ctr+). C, Cells from aorta or heart preferentially differentiated by forming 3D structures and expressed typical cardiac markers such as α‐actinin and ventricular myosin light chain 2 (MLC2V). Staining was performed at day 12 of differentiation. Right panels depict 3D projection and reconstruction of the *z*‐stack confocal sections. D, Immunofluorescence staining of typical sarcomere formation in Ao‐, At‐, and V‐fMAB forming aggregates. Ao, aorta; At, atria; Sk, skeletal muscle; V, ventricles. *P < .01. ND: not detectable

### 3.3 Muscle differentiation potentials of fMABs

When the four fMAB populations were cultured under serum‐reduced differentiation conditions (2% horse serum) to induce myogenesis, only Sk‐fMABs formed multinucleated myotubes (Figure 2A). A high expression in transcript levels of myogenic differentiation 1 (*MYOD1*), myogenin (*MYOG*), myostatin (*MSTN*), and myogenic factor 5 (*MYF5*) was seen by RT‐qPCR (Figure 2B, upper panel; Figure S3A). Notably, multinucleated myotubes were positive for MyoD and myosin heavy chain (*MHC*) immunostaining (Figure 2A). In contrast, Ao‐, At‐ and ventricular‐fMABs spontaneously formed 3D cardiac aggregates as shown by α‐actinin and myosin light chain 2v (*MLC2V*) staining (Figure 2C). 3D distribution of *MLC2v* and α‐actinin was particularly evident in these cardiac aggregates as illustrated in the 3D reconstruction of z‐stack confocal images (Figure 2C, far right panels, and Figure 2D). Moreover, the expression of cardiac specific genes such as *MEF2C*, *NKX2.5*, and *MyHC1* was confirmed by RT‐qPCR, and their levels were indistinguishable between the tested individual samples (Figure 2B, lower panel; Figure S3B).

**Figure 3:**
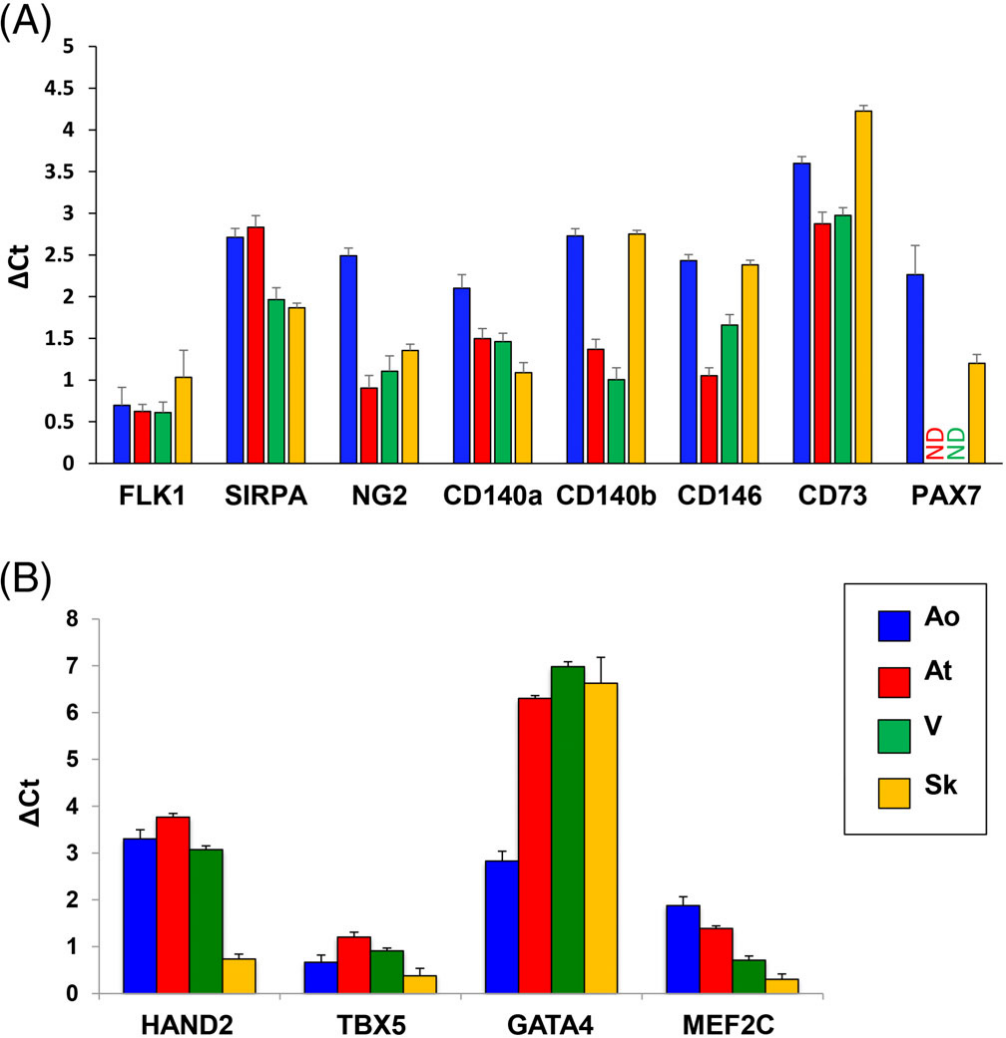
Molecular characterization by qPCR of the different fMAB populations for typical fetal mesoangioblast (fMAB) (A) or cardiac (B) markers. Ao, fMABs from aorta; At, fMABs from atria; ND, not detectable; Sk, fMABs from skeletal muscle; V, fMABs from ventricles

### 3.4 Transcriptome analysis of human fMABs derived from different tissues

To detect the potential differences in the transcript profile of the different fMAB populations that could account for the observed muscle differentiation potentials, we performed RNA‐seq on proliferating fMABs from Ao, At, ventricle (V), and Sk. Paired‐end RNA‐seq using Illumina platform yielded nearly 100 million reads for each cell sample.

We then plotted differentially expressed genes (*P* < .001) according to their fold‐change (log2, FC > 3) in three axes: V, Ao‐, and At‐fMABs (*X*, *Y*, and *Z*, respectively) all compared with Sk‐fMABs (Video S1). When a gene behaved differently in the three comparisons (Ao vs Sk, At vs Sk, and V vs Sk), the color was adjusted to the mean of the FCs (from red to green level). The 3D plot indicates that genes more expressed in cardiac tissues and Ao were less dispersed than genes downregulated in any of these tissues (At, V, Ao) when compared with Sk. Given such a differential pattern, we then first quantified by qPCR the expression of known MAB signature genes in the different MABs (Figure 3; Figure S4). MAB marker genes such as FLK1, SIRPA, NG2, CD140a,b, CD146, and CD73, but also cardiac‐related genes such HAND2, TBX5, GATA4, and MEF2C, were all present in the different fMABs at comparable levels (Figure 3; Figure S4). Notably, the Sk TF Pax7 was present only in Ao‐ and Sk‐MABs (Figure S3A; Figure S4B), whereas HAND2, TBX5, and MEF2C were highly enriched in Ao, At, and ventricular fMABs, but quite reduced in the Sk‐fMABs (as expected) Instead, GATA4 expression resulted lower in Ao‐fMABs compared with the other fMABs (Figure 3B; Figure S4C). Such a trend characterizing the different populations was globally confirmed by RNA‐seq values (Figure S5).

**Figure 4:**
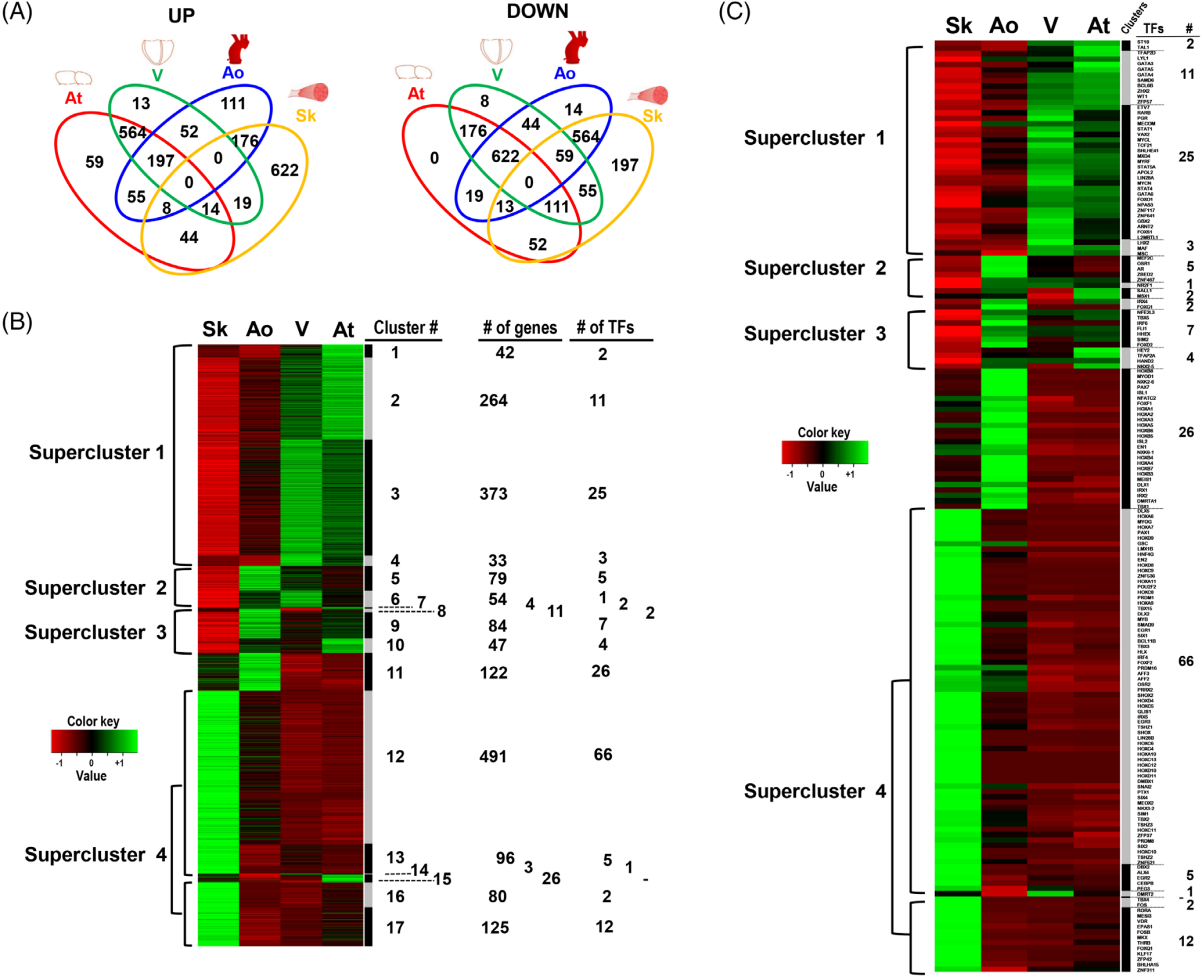
Comparison of the differentially expressed genes. A, The Venn diagrams illustrate the number of significantly upregulated or downregulated genes and their respective commonality between the different fetal mesoangioblast (fMAB) populations. B, Hierarchical clustering based on gene expression levels between the four fMAB populations. The heatmap represents genes calculated as differentially expressed in any paired‐comparison (FC > 3, *P*‐value<.001). Expression values in Reads Per Kilobase Million were used to calculate the *z*‐scores for each gene. Genes in the hierarchical clustering were grouped by a similar expression pattern. Clusters allowing distinction between tissues were merged in four main superclusters.^1^, ^2^, ^3^, ^4^ The scale bar shows the *z*‐score values for the heatmap. Ao, aorta; At, atrium; Sk, skeletal muscle; V, ventricle. C, Heatmap reporting the differentially expressed transcription factors in each cluster

**Figure 5:**
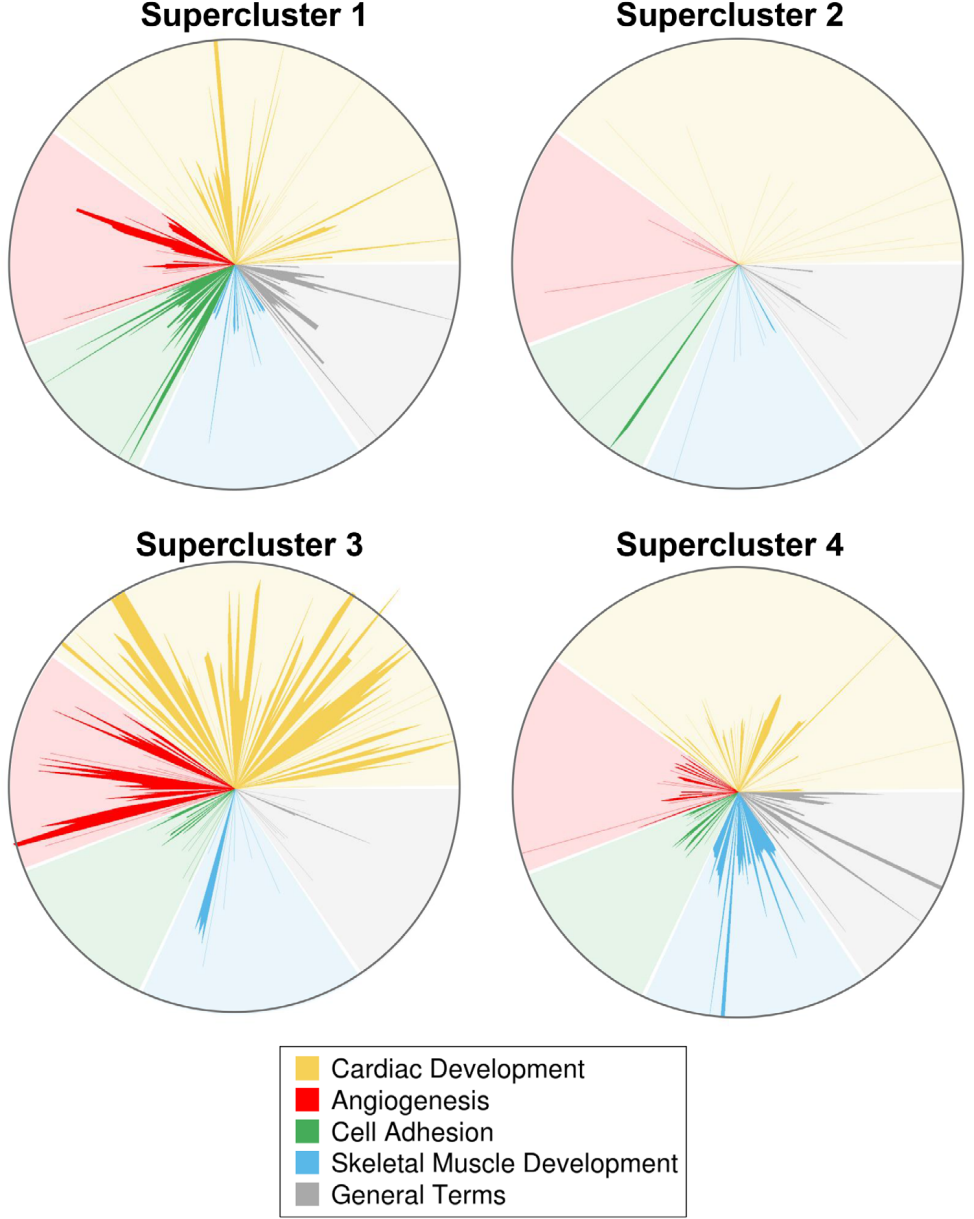
Star‐plot representation of major enriched Gene Ontology biological processes (GOBPs) in the four different fetal mesoangioblast (fMAB) populations. Significantly enriched biological processes (odds ratio >2, *P*‐value <.05) in any of the superclusters were selected and grouped in five general categories (Cardiac Development, Angiogenesis, Cell Adhesion, Skeletal Muscle Development, and General Terms). Each GOBP is always represented with the same angle in order to allow easy and quick comparison between the most enriched GOBP within superclusters. Length of each radius is given by log2(1 + odds ratio). When the odds ratio <2 and/or *P*‐value >.05, the value of the radius was set to zero. Star‐plot representation of major enriched GO biological processes in the four different fMAB populations and expressed as the percentage of the five most represented biological processes of interest. DEV, development; GO, Gene Ontology

### 3.5 Paired comparison of the differentially expressed genes in human fMABs

In order to identify major key genes possibly explaining cardiac or Sk differentiation potentials in different fMABs, we analyzed upregulated/downregulated genes between the four different fetal tissues after performing paired statistical analysis to minimize possible differences in genetic background or developmental stages. We identified high‐ and low‐expressed genes by the following criteria: Log2 FC ≥3 or Log2 FC ≤−3, FDR <0.05, and *P*‐value <.05, and plotted commonalities in Venn diagrams (Figure 4A). Concerning tissue‐specific genes, exclusive up‐ and down‐expressed ones were, respectively, 111 and 14 in Ao, 59 and 0 in At, 13 and 8 in ventricle, 622 and 197 in Sk (Figure 4A).

The hierarchical clustering of gene expression levels (in RPK, reads per kilobase) in different fMABs indicated high similarity between the heart samples (At and V), whereas the skeletal ones were the most distinct cell populations. Interestingly, the gene expression levels of Ao samples appeared to be intermediate between Sk and cardiac fMABs (Figure 4B).

We then performed hierarchical clustering of differentially expressed genes (in RPK, reads per kilobase) between Ao, cardiac, and skeletal fMABs. Figure 4B illustrates the 17 identified gene clusters with the number of genes and the included TFs (Tables S1 and S2). The expression profile differentiated skeletal from heart‐derived cells in terms of upregulated and downregulated genes and showed a high similarity between the heart samples (At and V), whereas the skeletal ones were the most distinct cell population. Notably, Ao displayed an intermediate expression profile compared with cardiac and Sk‐fMABs (Figure 4B). We could identify four major superclusters: the first included gene clusters 1 to 4 (712 genes, of which 41 are TFs), and highlighted genes that were upregulated in the heart (At and V) but downregulated in Ao and Sk. Instead, the second supercluster comprised genes upregulated both in V and Ao but downregulated only in Sk. Regarding supercluster 3, the grouped upregulated genes were present both in Ao and At and were still downregulated only in Sk. Finally, the supercluster 4 contained genes upregulated only in Sk‐fMABs (and down in the three other tissues), such as the expected muscle‐specific TFs *MYOD1*, *MYOG*.

### 3.6 Gene Ontology analysis of enriched genes

To highlight major biological processes related to muscle development and how these compare between the different tissues of origin, we classified all enriched genes into five main biological processes of interest using Gene Ontology (Table S3). We displayed them as star plots (Figure 5), according to the four superclusters previously identified. We focused our attention on cardiac and Sk‐related biological processes, and we thus compared statistically enriched gene groups in categories such as “Cardiac development”, “Skeletal muscle development”, as well as “Angiogenesis” and “Cell adhesion”, that were highly represented only in Ao, At, and Sk. All other terms, including non‐cardiac or non‐Sk‐related terms (such as “Protein oxidation”, “Regulation of cell respiration”, etc., not shown), were reductively included in the category labeled as “General terms”. As expected, the number of GO biological processes included in cardiac development category was much greater in superclusters 2 and 3 (down in skeletal vs heart and Ao, yellow spikes in Figure 5). Analogously, Sk development‐related GO processes were more numerous in cluster 4 (up in skeletal vs heart and Ao, blue spikes). Interestingly, cell adhesion BPs resulted more represented only in At and V (supercluster 1, green spikes).

In order to figure out functional interpretation for these changes, we performed GO analysis based on REVIGO annotation database,^23^ particularly focusing our attention on cardiac, angiogenic, and Sk‐related terms (Figure S6). The results showed that upregulated genes in Ao were annotated with heart formation, cardioblast proliferation, and cardiac chamber development. Heart development, heart morphogenesis, and cardiac muscle tissue development were highly represented GO terms (biological processes) in At samples. Interestingly, cardiovascular system development, heart contraction, cardiac muscle hypertrophy, cardiac vascular smooth muscle cell differentiation, and ventricular cardiac muscle development were the top five most represented biological functions of upregulated genes in Sk‐fMABs.

**Figure 6:**
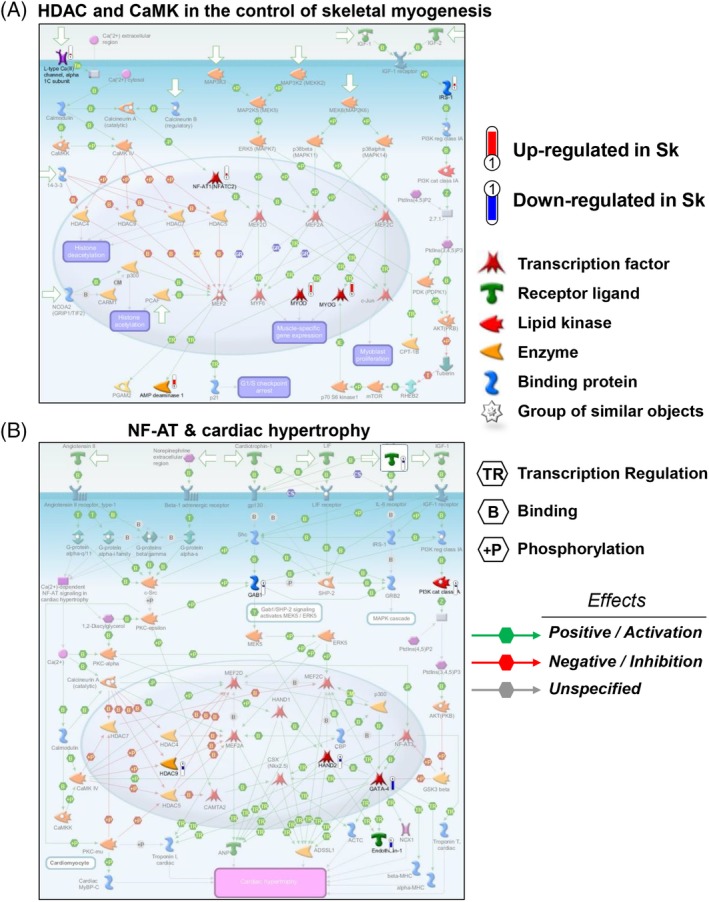
MetaCore‐based analysis of two major KEGG pathways and their regulatory gene interactions in skeletal and cardiac human fetal mesoangioblasts (MABs). A, Canonical pathway representation of the Sk‐MAB‐enriched genes. B, Canonical pathway graph of the ventricle‐enriched genes. Sk, skeletal muscle

We then examined the TFs among the gene list belonging to each particular biological process cluster (cardiogenesis, angiogenesis, and skeletal myogenesis; Figure S6). Interestingly, *ISL1*, *TBX1*, and *FOXF1* were the only three TFs present in At for both cardiogenesis and angiogenesis processes, whereas *HEY2* and *GATA3* were the TFs showing up in Ao. Concerning the Sk‐fMABs, different cardiogenesis‐related TFs, aside *HEY2*, were significantly present, such as *TBX2*, *HAND2*, *IRX5*, *GATA4*, and *GATA6* in both processes. In contrast, Sk‐related TFs were expectedly present only in Sk‐fMABs for skeletal myogenesis, such as *MYOG*, *SIX1*, and homeobox genes like *SHOX2*, *MEOX2*, and *HOXD* members involved in Sk development.

### 3.7 Comparative analysis of ventricle vs Sk fMABs

To compare the relative expression levels of each gene within the four cell types, we analyzed paired data from Ao‐, At‐, V‐, and Sk‐fMABs using MetaCore software, recognizing common or unique lineage enrichment patterns to which genes belong (statistical significance of gene expression set with a fold change −3 < FC > 3 and *P* < .05). In particular, we focused our attention on the comparison between V‐ and Sk‐fMABs. All statistically significant upregulated or downregulated genes from these two cell types were grouped based on their pathway categories, indicating patterns of differentially expressed genes in both samples. Sk vs V analysis revealed that tissue‐specific genes were strongly enriched into distinct pathways, namely HDAC and CaMK‐dependent control of skeletal myogenesis (Figure 6A) and NF‐AT and cardiac hypertrophy (Figure 6B). As expected, genes with high enrichment in V‐fMABs were mostly responsible for cardiac functions, such as cardiac muscle contraction, heart rate, angiogenesis, and hypertrophic cardiomyopathy. Alternatively, enriched genes in Sk‐fMABs mainly related to the development of blood vessel, muscle contraction, and Sk development, which is consistent with the known functions of Sk‐fMABs. Specifically, these enriched genes were basically involved in functional categories related to Sk myogenesis with genes like *NF‐AT*, *MYOD*, *MYOG* being upregulated (Figure 6A), whereas most V‐fMAB genes, like *PI3K* and *HDAC9*, were downregulated (Figure 6A). Conversely, these latter genes together with *GATA4* and *HAND2* resulted upregulated in V samples (Figure 6B), belonging to functional categories of heart development and cardiac hypertrophy. Specific differentially expressed TFs belonging to each cluster are shown in Figure 4C.

**Figure 7:**
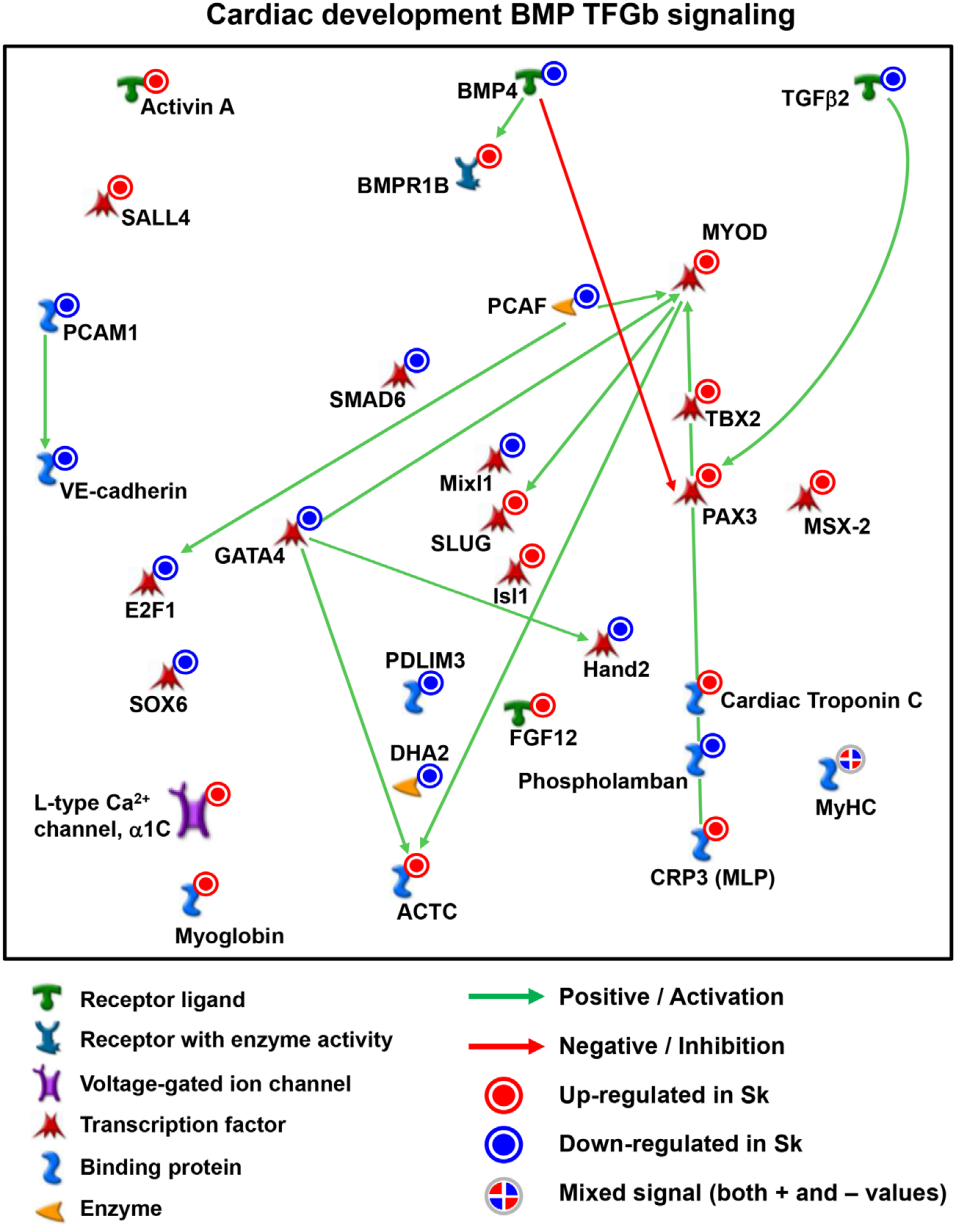
Network connection plot of commonly enriched transcription factors in ventricular and skeletal muscle mesoangioblasts (MABs). Upregulated (red) and downregulated (blue) genes are identified by thermometer‐like scales aside significantly expressed genes

We then identified that many genes upregulated or downregulated in V‐ or Sk‐fMABs, respectively, were commonly involved in specific networks such as muscle contraction (data not shown). Those include blood vessel formation and cardiac differentiation, likely involving common but dosage‐dependence effects in these two perivascular stem/progenitor cell types for these functions. Interestingly, MetaCore analysis revealed the enrichment of few upregulated genes in Sk‐fMABs such as *TBX2* and *Isl1* that are involved both in cardiac‐ and muscle‐related processes (Figure 7; Table S4).

As for Sk‐fMAB enriched genes, V‐fMAB genes showed other specific functional enrichments as well. Genes involved in cell cycle progression, mitosis‐related functions, cell adhesion, integrin‐mediated cell matrix adhesion, and signal transduction (WNT and NOTCH) were enriched in both cell lines (data not shown). Genes enriched in Sk‐fMABs were also involved in vascular formation and calcium transport‐related functions, and other genes enriched in V‐fMABs were concentrated into the bio‐functions of potassium transport and heart disease (data not shown). Similarly, we observed that key component genes of some signaling pathways known to play important roles in cardiovascular and Sk development, such as *Wnt/NOTCH* and *BMP*, exhibited common specific enrichment in both cell types (data not shown). Taken together, these results suggest that lineage enriched genes underlie the dynamic biofunctional specializations and the temporal activations of signaling pathways to control the stepwise development of cardiovascular and Sk lineages from human fMABs. Moreover, comparisons of biological pathways that were enriched in V‐MABs vs Sk‐fMABs could further define cardiac vs Sk cell differentiation commitment.

### 3.8 Analysis of TF network modeled the gene interaction of cardiovascular and Sk lineages in human fMABs

It has been previously demonstrated that HAND2, GATA4, and TBX5 are among the central TFs in adult cardiac MABs.^9^ As TFs regulating different lineage commitments may be interconnected, we dissected common and unique TF networks that may allow us to predict MAB propensity toward cardiovascular and/or Sk fates. Additionally, we built the lineage‐specific TFs in Sk‐fMABs. TFs of each single lineage indicated the dominant gene programs controlling lineage‐specific biofunctions. Whereas V‐fMAB TFs reflected the dynamic TF transition during cardiovascular development, Sk‐fMAB TFs revealed commonly shared mechanisms during Sk formation (Figure 7; Table S4). The expression level of some cardiac specific TFs, such as HAND2 and GATA4, was higher in V‐fMABs but still expressed in Sk‐fMABs (Figure 7; Table S4), indicating that these TFs may function at stages of both heart and Sk progenitor formation and cell specification.

## 4 DISCUSSION

Several studies have addressed the in vitro differentiation characteristics of rodent and human MABs derived from adult individuals,^4^ reviewed in^24^, and the first MAB‐based clinical trial for muscle regeneration in genetic diseases (Duchenne muscular dystrophy) has already shown safety.^15^ Concerning heart applications, a cardiac impact of MABs has been reported in mice featuring limb‐girdle muscular dystrophy,^25^ and MAB‐derived CMs have been described,^9^ yet an efficient in vitro differentiation of MABs into cardiac cells is far from been achieved. Since the transcriptional identity of human fMABs was missing, we analyzed their transcriptome to uncover specific gene signatures correlating with their peculiar myogenic differentiation properties due to their tissue of origin. Indeed, a differential tissue‐dependent potential of fMABs could influence cellular treatments of muscular degeneration, in particular when restoration of both cardiac and Sk functionality is required.^5^ We chose heart and Sk fMABs because their myogenic bias has already been partially documented,^26^ and their intrinsic propensity was hypothesized to be of potential relevance in the context of muscle repair. In this view, a refined understanding of the source‐related myogenic propensity may ease the protocols of tuning the intrinsic fate of tissue‐specific muscular progenitor cells.

Here, we provide the first systematic intra‐individual analysis of human fMABs from Sk, heart (At and V), and Ao, this latter developmentally originating from Isl1^+^ progenitors of the secondary heart field which forms the entire outflow tract, the atria^2^ and the right ventricle.^27^ All sorted according to a common marker profile (CD13/CD90/CD146/AP/NG2‐positive and CD34/CD45‐negative), the fetal heart MABs (At, V, and Ao) displayed differentiation propensities upon reduced serum exposure that were reminiscent of their tissue of origin, namely 3D aggregates positive for typical cardiac myofibrillar proteins (α‐actinin and MLC2v) and TFs (MEF2C, Nkx2.5, etc.). The Sk‐fMABs, instead, always formed multinucleated myotubes.

Bioinformatics analysis of RNA‐seq data revealed for the first time that the fMABs from Ao, cardiac (At and V), and Sk display subsets of differentially expressed (upregulated or downregulated) genes likely representing distinct expression signatures indicative of their origin. The comparative transcriptional profiling identified 17 clusters of differentially expressed genes and clearly demonstrated a global opposite set of upregulated and downregulated genes between Sk and cardiac (At, V) fMABs. Expectedly, the highly expressed genes clustering within two of the four major superclusters were involved in major biological processes regulating cardiac development, cell adhesion, and angiogenesis in cardiac fMABs (supercluster 1) or Sk development in Sk‐fMABs (supercluster 4), respectively. Interestingly, genes of Ao‐fMABs revealed an intermediate profile between Sk and heart muscle: they were upregulated only in Ao or V (supercluster 2) and fallen into processes involved in angiogenesis and cell adhesion, whereas the upregulated genes in Ao or At highlighted processes regulating angiogenesis and cardiac development (supercluster 2).

In our experimental settings, the genetic cues found on cardiomyogenic markers correlated with a robust cardiomyogenic potential of At and V‐fMABs. Conversely, tissue‐related conservation of genetic signatures on Sk genes was associated with a differential intrinsic propensity of Sk‐fMABs toward the Sk lineage.

Our transcriptome analysis intended to provide possible insights of a retained genetic imprinting reminiscent of the niche of origin and uncover key regulators susceptible to skew intrinsic propensities. Notably, we identified putative key regulators of myogenic bias retainment and, thereby, putative targets enabling to specifically increase or decrease skeletal or cardiac myogenic propensity during differentiation.

We are aware that our RNA‐seq analysis was limited to a small number of regulatory elements and genes, thereby only sufficient for describing a correlation between the differential propensity of the four tissues (Ao, At, V, and Sk) as sources of myogenic bias in the context of striated muscle differentiation/regeneration.

Specifically, we consistently isolated fMABs as an alkaline phosphatase‐sorted (AP^+^‐sorted) subfraction of pericytes in all four tissues (Ao, At, V, and Sk). Although the mechanisms are still largely unknown, AP apparently marks a myogenic subset of resident pericytes in the Sk that are locally committed to skeletal myogenesis, especially in vivo.^18^ Importantly, the intrinsic myogenic differentiation capacity of resident and injected murine MABs to form Sk tissue was confirmed in a transgenic, inducible genetic system relying on *AP:CreERT2* recombinase.^28^ Notably, although restricted to MABs of embryonic origin, the genetic ablation of *Pax3* blunted the skeletal myogenic potential of MABs.^29^ Moreover, by means of a dedicated scaffold, murine MABs could generate functional artificial Sk tissue in vivo.^30^ Thus, resident AP^+^ MABs from Sks appear intrinsically myogenic, probably to a species‐specific variable extent, and, as shown by the present work, this intrinsic myogenic bias appears already in fetal muscles and durable in our standard culture conditions.

Based on the whole‐transcriptomic differences in myogenic commitment, we are keen to hypothesize possible tunable shifts in differentiation between Sk and cardiac MABs in vitro. This could be achieved by activating and/or inhibiting key signaling pathways. For example, small‐molecule regulators of miRNAs, known to regulate muscle development, could be instrumental to modulate MyoD protein levels^31^ that are increased in Sk‐ vs V‐fMABs, thereby addressing the role of suppressing primary myomiRs and their effect on differentiation‐related genes. Furthermore, the use of human recombinant proteins able to modulate myogenic TFs can be a valid tool to improve myogenic commitment.^32^ To this end, studies that are more comprehensive are required to shed light on miRNAs fingerprints between the different MAB populations in order to crystallize the molecular cues possibly discriminating between myogenic propensities in these cells. In this perspective, an intriguing experimental question would be whether Sk‐fMABs retain a differentiation propensity similar to Ao‐ or even cardiac‐fMABs, when exposed to discriminant differentiative cues. Whether such maneuvers would eventually restrict the general properties of “transdifferentiating” MABs (ie, proliferation and/or differentiation efficiencies) remains to be fully addressed.

As expected, Sk differentiation of Sk‐fMABs correlates with the expression levels of myogenic markers such as MyoD and MyoG. Similarly, significantly higher levels of GATA4 and HAND2 were found in cardiac MABs when compared with Sk MABs (Figure 6). We can speculate that interventions in Sk‐MABs, modulating MyoD, or cardiac TFs such as GATA4 and HAND2, could increase cardiac differentiation in vitro. Indeed, these two latter genes are synergistically transcribed in association with p300 recruitment.^33^

With regard to the spontaneous cardiac differentiation of V‐MABs, their high efficiency in generating cardiac clusters in vitro was counter‐balanced by their immature state (the absence of spontaneous beating, immature pattern of terminal myofibril markers). Further studies are thus required to augment the terminal maturation of ventricle‐derived myocytes in vitro, particularly the effect of cell‐specific microvesicles mimicking in vivo cardiac engraftment.

From the comparative expression profile of both Sk and cardiac MABs, some shared key genes such as Cardiac Troponin C, PAX3, TBX2, MyoD, Isl1, GATA4, and HAND2 (although with differential expression levels, Figure 7 and Table S4) let us to speculate that (aside from MyoD and PAX3) both MABs may be susceptible to in vitro differentiate into either cardiac or skeletal striated muscles. Furthermore, a refined assessment of the in vivo regenerative potential of human MABs (“wild‐type” or gene/signaling‐boosted cardiac or skeletal cells into the myocardium) will be crucial for progression along the translational path of MAB‐based therapies for striated muscle regeneration.

In vitro, both cardiac and Sk‐MABs express different levels of *MyoD* and *Myogenin* as markers of skeletal myogenesis, and of *Bmp4*, *Gata4* and *Isl1*, as markers of early cardiomyogenesis. The coexpression of cardiac and skeletal myogenic TFs was demonstrated in cardiac MABs isolated from β‐Sarcoglycan‐null (*Sgcb*‐*null*) dystrophic mice. Notably, cardiac *Sgcb* MABs depleted of miR‐669a/q spontaneously differentiated into skeletal myotubes in vitro and into arrhythmogenic Sk patches into infarcted hearts in vivo. The robust lineage shift relies on downregulation of these miRNAs, normally repressing MyoD translation. Reintroduction of *miR*‐*669a*, in fact, partially rescues the cardiomyogenic commitment of *Sgcb*‐*null* both in vitro and in vivo.^34^

Concerning aorta‐derived MABs (Ao‐MABs) which have plasticity toward mesodermal derivatives, their injection intramyocardially leads to engraftment into the myocardium of dystrophic mice, and differentiation into CMs and ECs, thus preventing the onset of dilated cardiomyopathy.^17^ Similarly, it promoted angiogenesis and endogenous cardiac stem/progenitor cell proliferation in *mdx/utrn* ^*−/−*^ but not aged *mdx* mouse models for Duchenne muscular dystrophy. In contrast, under appropriate inducing conditions, Ao‐MABs could also transdifferentiate both in vitro and in vivo toward myelinating glial cells or into oligodendrocytes by inhibition of the rho kinase signaling pathway.^35^

## 5 CONCLUSION

In summary, we identified GO biological processes and KEGG pathways that may account for their distinct differentiation outcomes. Collectively, our analysis provides a set of critical genes possibly predicting future specific differentiation outcomes. We thus provide compelling evidence that TF networks of each single cell type underlie its dominant gene program to control the lineage‐specific signature biofunction during development. The refined lineage‐specificity map may ease the identification of key factors pertaining to specific temporal windows during differentiation or to a specific cell fate according to the relative KEGG pathways.

In essence, our results suggest that the comparative gene signature analyses are crucial to uncover combinatorial gene modules at the basis of differential cell type outcomes. This study therefore stems for future development of methods to improve cardiac differentiation from human extra‐cardiac MAB sources, approaching novel therapeutic applications for muscle regeneration.

## CONFLICT OF INTEREST

The authors declared no potential conflicts of interest.

## AUTHOR CONTRIBUTIONS

F.L.R.: conception and design, collection and/or assembly of data, data analysis and interpretation, manuscript writing, final approval of manuscript; R.K., S.L.: data analysis and interpretation, final approval of manuscript; M.S.: manuscript writing, final approval of manuscript; M.E.J.: conception and design, collection and/or assembly of data, financial support, manuscript writing, final approval of manuscript.

## Supporting information


**Figure S1** Karyotype analysis of the two fetuses used for RNA‐seq analysis (A) and proliferation curves (B) of the four isolated mesoangioblast cell types. After cell isolation, cells were considered to be at 1 population doubling. (* *P* < .01). N = 3 independent experiments.

**Figure S2** Induced differentiation of fMABs derived from aorta (Ao), atria (At), ventricles (V) and skeletal muscle (Sk) toward adipogenic, osteogenic, chondrogenic and smooth muscle fate.
**(A)** Adipogenesis was revealed by Oil Red O, osteogenesis by Alizarin Red, and chondrogenesis by Safranin staining. **(B)** q‐PCR marker characterization: *FABP4 (Fatty Acid Binding Protein 4)* and *PLIN1 (Perilipin 1)* are adipocyte markers; *SPARC* (*Secreted protein acid and cysteine rich osteonectin*) and *ALPL (Alkaline Phosphatase*) are osteogenic markers; *COL2a* (*Collagen Type II alpha*) and *ACAN* (*Aggrecan*) are chondrogenic markers. *CNN1 (calponin 1)* is a marker of smooth muscle differentiation. The data are plotted separately for each individual (12 and 13 weeks of age, respectively). Human foreskin fibroblasts were used as negative control (Ctr‐) while human mesenchymal stem cells (properly differentiated as osteo−/chondro−/adipocytic derivatives) were used as positive control (Ctr+). * *P* < .01, ND, not detectable.

**Figure S3** q‐PCR analysis of skeletal **(A)** and cardiac **(B)** marker expression in the different fMAB populations (Ao, At, V and Sk). Data are plotted separately for each individual (12 and 13 weeks of age, respectively). Human foreskin fibroblasts were used as negative control (Ctr‐) in both panels, while human satellite cells and human cardiomyocytes were used as positive controls (Ctr+) in panels A and B respectively. * *P* < .01, ND, not detectable.

**Figure S4** qPCR characterization of markers present in the different fMAB populations. Typical markers are plotted separately for each individual (12 and 13 weeks of age, respectively). Ao: fMABs from aorta; At: fMABs from atria; V: fMABs from ventricles; Sk: fMABs from skeletal muscle. ND: not detectable.

**Figure S5** RNA‐seq expression analysis of typical **(A)** and cardiac **(B)** fMAB markers expressed by cells derived from the four tissues. Data are consistent with qPCR characterization (see Figure 3 A, B).

**Figure S6** Biological process clustering. Significant Gene Ontology analysis for three major selected Biological Processes is expressed as tables including GO terms, number of genes, log10 P‐value and the included transcription factors (TFs). Frequency indicates the percentage of human proteins in UniProt that were annotated with a GO term in the GOA database. Main representative clusters are given in black letters, while sub‐cluster members are in grey italics. TF list indicates the transcription factors belonging to that particular biological process.

**Table S1** Gene clustering with the relative z‐scores calculated for the four fMAB populations.
17 clusters generated by hierarchical clustering of differentially expressed genes between Ao‐, At‐, V‐ and Sk‐fMABs (see Figure 4B). Cases highlighted in blue indicate transcription factors.

**Table S2** List of transcriptionally enriched transcription factors within the different gene clusters (list related to Figure 4C).
Z‐score were expressed by 1, 2, or 3 + symbols according to these values: +: 0.5 < z‐score < 1; ++: 1 < z‐score < 1.25; +++: z‐score > 1.25.

**Table S3** OddRatio values (*P* < .05) used for the generation of star‐plots in Figure 5.

**Table S4** Interaction report of up‐ and down‐regulated genes in V‐ *versus* Sk‐MABs (as depicted in Figure 7).

**Video S1** Graph of the differentially expressed genes plotted according to their fold‐change (log2) in 3‐axes (X = Ventricle; Y = Aorta and Z = Atrium, all compared to Skeletal fMABs). Only genes with significant P‐values lower than 0.001 and fold‐changes (FC) above three were plotted. Up‐regulated and down‐regulated genes compared to the ones expressed in skeletal tissue are in green and red, respectively. When a gene behaves differently in the three comparisons (Aorta vs Skeletal, Atrium vs Skeletal and Ventricle vs Skeletal), the colour is adjusted to the mean of the fold‐changes (from red to green level).

## Data Availability

The sequence data that supports this study are accessible through the GEO database under the accession number GSE90069.
