# Supplementary figures and images for: Human fetal mesoangioblasts reveal tissue‐dependent transcriptional signatures
Source: Stem Cells Transl Med. 2020 Jan 23;9(5):575–89. doi: 10.1002/sctm.19-0209 (PMC7180296; doi:10.1002/sctm.19-0209)

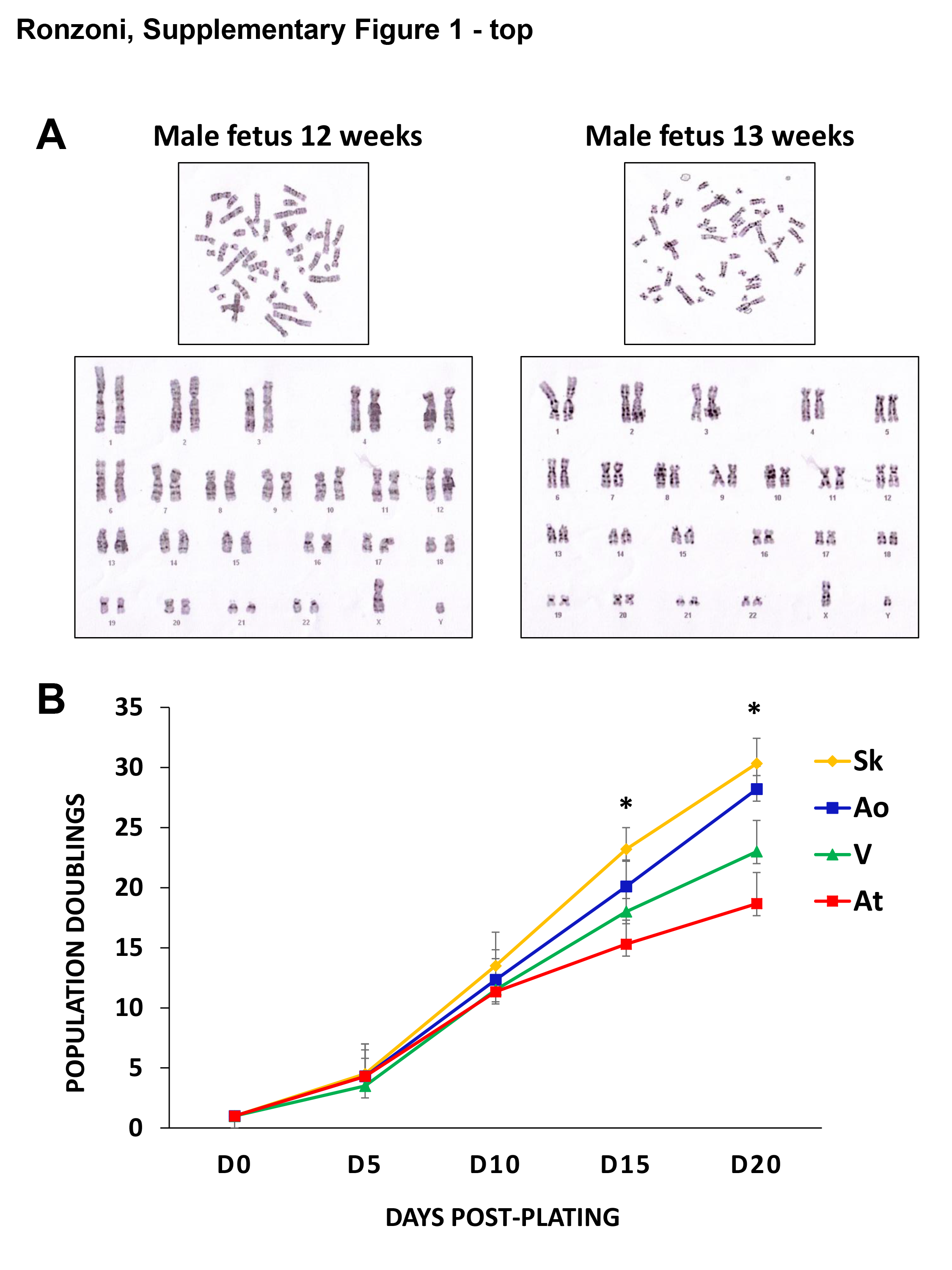

Supplement: Supplementary file 1 — Figure S1 Karyotype analysis of the two fetuses used for RNA‐seq analysis (A) and proliferation curves (B) of the four isolated mesoangioblast cell types. After cell isolation, cells were considered to be at 1 population doubling. (* P < .01). N = 3 independent experiments. [file SCT3-9-575-s001.tif]

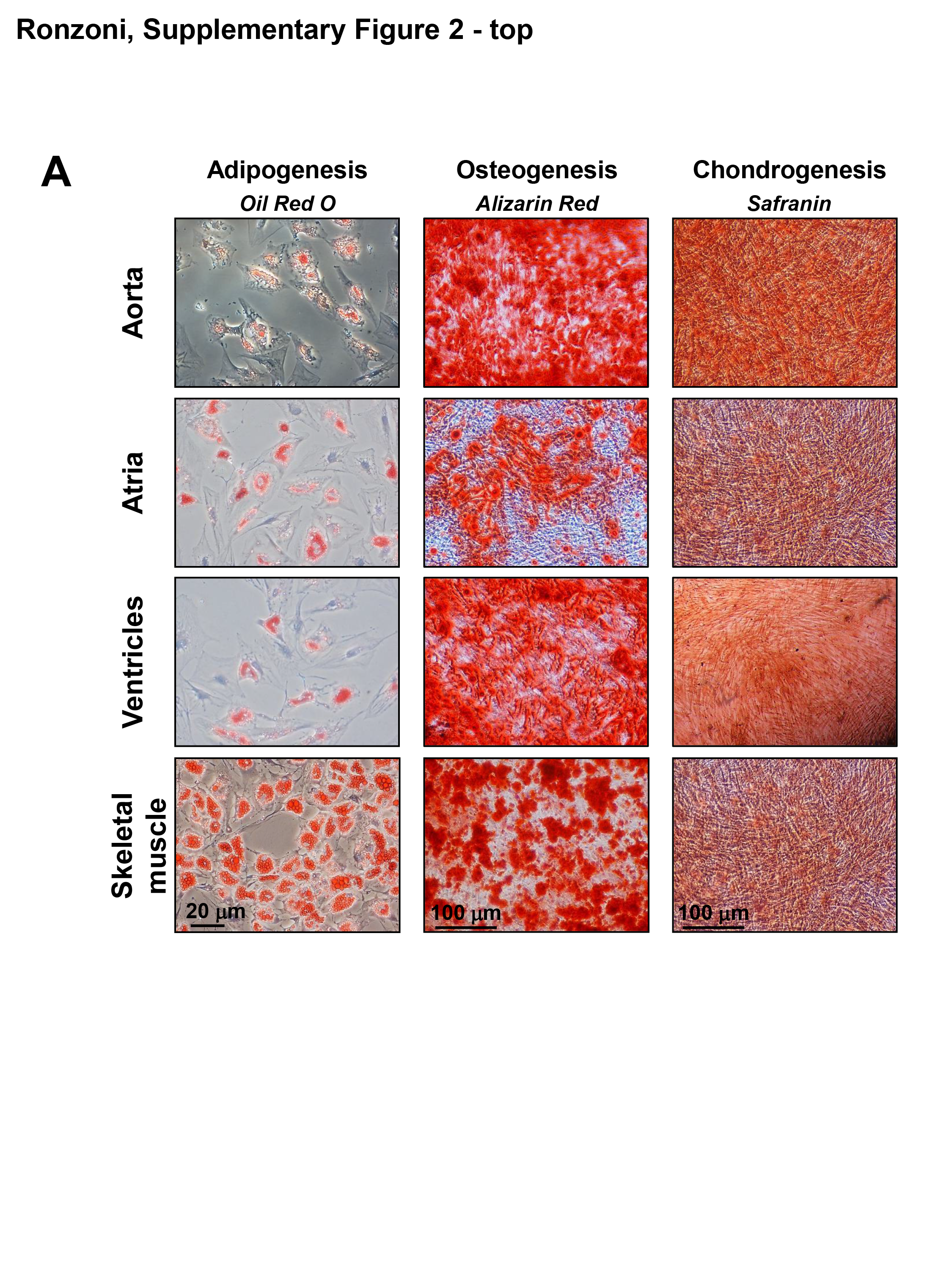

Supplement: Supplementary file 2 — Figure S2 Induced differentiation of fMABs derived from aorta (Ao), atria (At), ventricles (V) and skeletal muscle (Sk) toward adipogenic, osteogenic, chondrogenic and smooth muscle fate. (A) Adipogenesis was revealed by Oil Red O, osteogenesis by Alizarin Red, and chondrogenesis by Safranin staining. (B) q‐PCR marker characterization: FABP4 (Fatty Acid Binding Protein 4) and PLIN1 (Perilipin 1) are adipocyte markers; SPARC (Secreted protein acid and cysteine rich osteonectin) and ALPL (Alkaline Phosphatase) are osteogenic markers; COL2a (Collagen Type II alpha) and ACAN (Aggrecan) are chondrogenic markers. CNN1 (calponin 1) is a marker of smooth muscle differentiation. The data are plotted separately for each individual (12 and 13 weeks of age, respectively). Human foreskin fibroblasts were used as negative control (Ctr‐) while human mesenchymal stem cells (properly differentiated as osteo−/chondro−/adipocytic derivatives) were used as positive control (Ctr+). * P < .01, ND, not detectable. [file SCT3-9-575-s002.zip › SCT3_12662_Ronzoni_Suppl-Figure_2A_revised.tif]

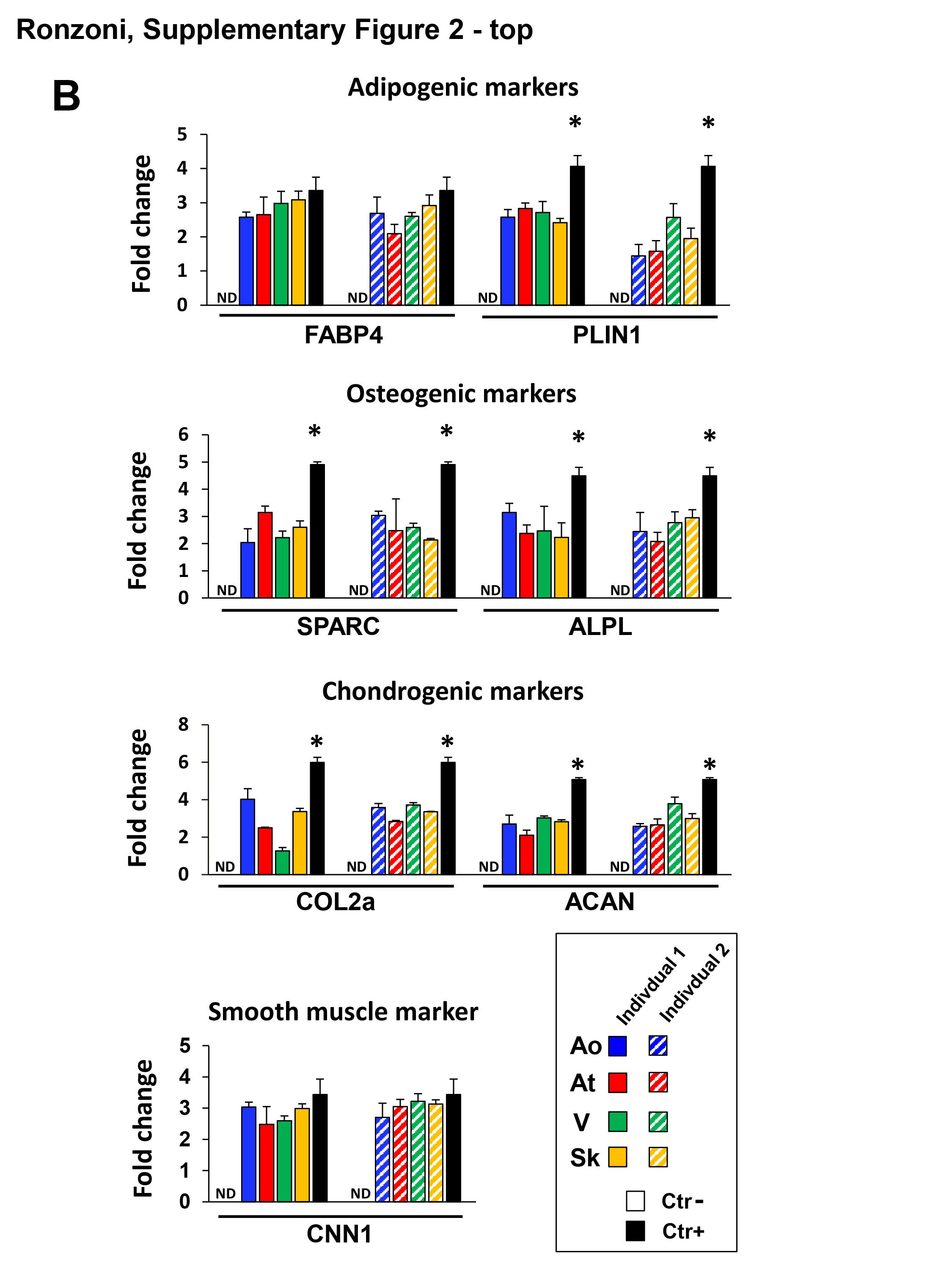

Supplement: Supplementary file 2 — Figure S2 Induced differentiation of fMABs derived from aorta (Ao), atria (At), ventricles (V) and skeletal muscle (Sk) toward adipogenic, osteogenic, chondrogenic and smooth muscle fate. (A) Adipogenesis was revealed by Oil Red O, osteogenesis by Alizarin Red, and chondrogenesis by Safranin staining. (B) q‐PCR marker characterization: FABP4 (Fatty Acid Binding Protein 4) and PLIN1 (Perilipin 1) are adipocyte markers; SPARC (Secreted protein acid and cysteine rich osteonectin) and ALPL (Alkaline Phosphatase) are osteogenic markers; COL2a (Collagen Type II alpha) and ACAN (Aggrecan) are chondrogenic markers. CNN1 (calponin 1) is a marker of smooth muscle differentiation. The data are plotted separately for each individual (12 and 13 weeks of age, respectively). Human foreskin fibroblasts were used as negative control (Ctr‐) while human mesenchymal stem cells (properly differentiated as osteo−/chondro−/adipocytic derivatives) were used as positive control (Ctr+). * P < .01, ND, not detectable. [file SCT3-9-575-s002.zip › SCT3_12662_Ronzoni_Suppl-Figure_2B_revised.tif]

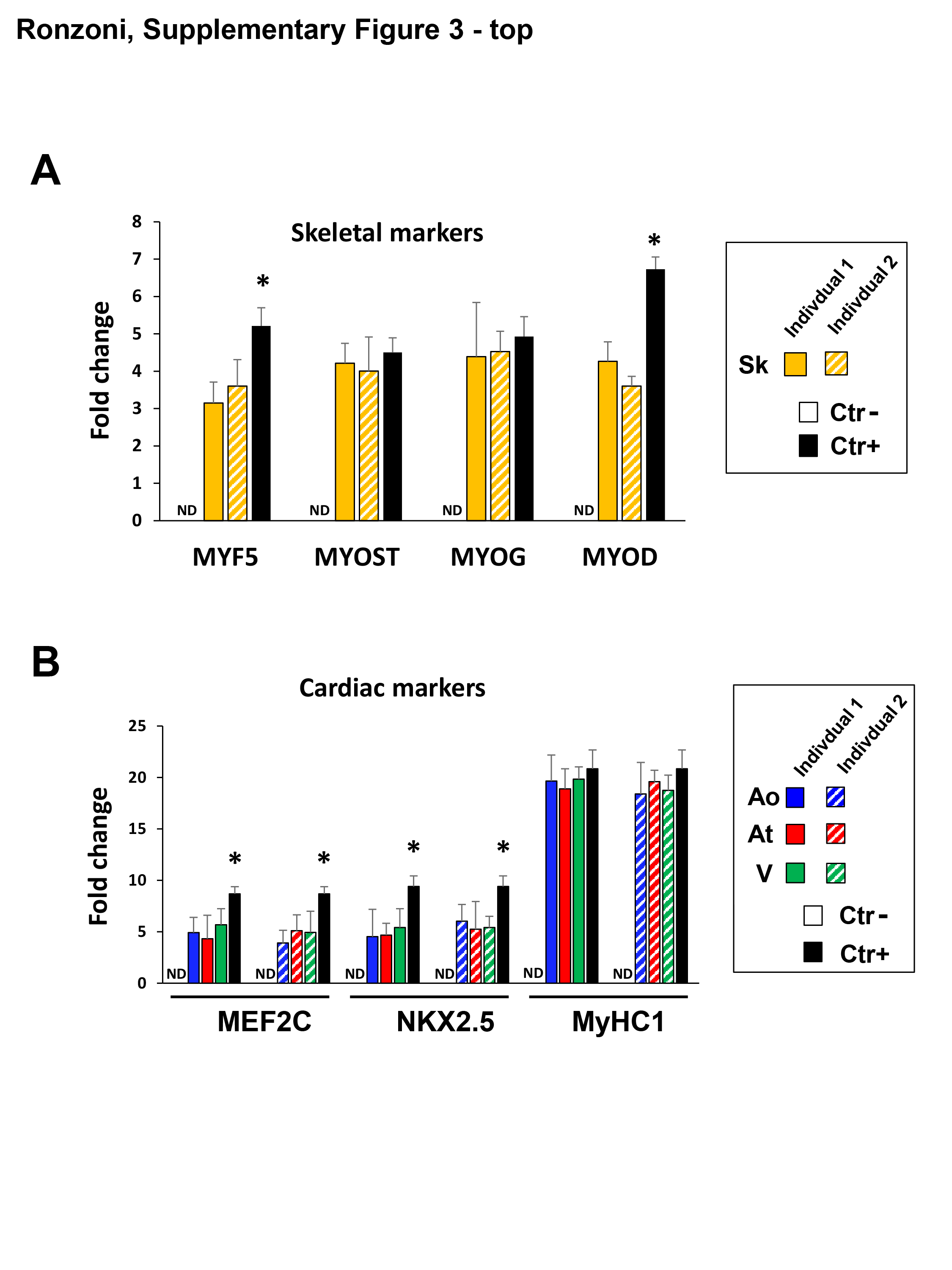

Supplement: Supplementary file 3 — Figure S3 q‐PCR analysis of skeletal (A) and cardiac (B) marker expression in the different fMAB populations (Ao, At, V and Sk). Data are plotted separately for each individual (12 and 13 weeks of age, respectively). Human foreskin fibroblasts were used as negative control (Ctr‐) in both panels, while human satellite cells and human cardiomyocytes were used as positive controls (Ctr+) in panels A and B respectively. * P < .01, ND, not detectable. [file SCT3-9-575-s003.tif]

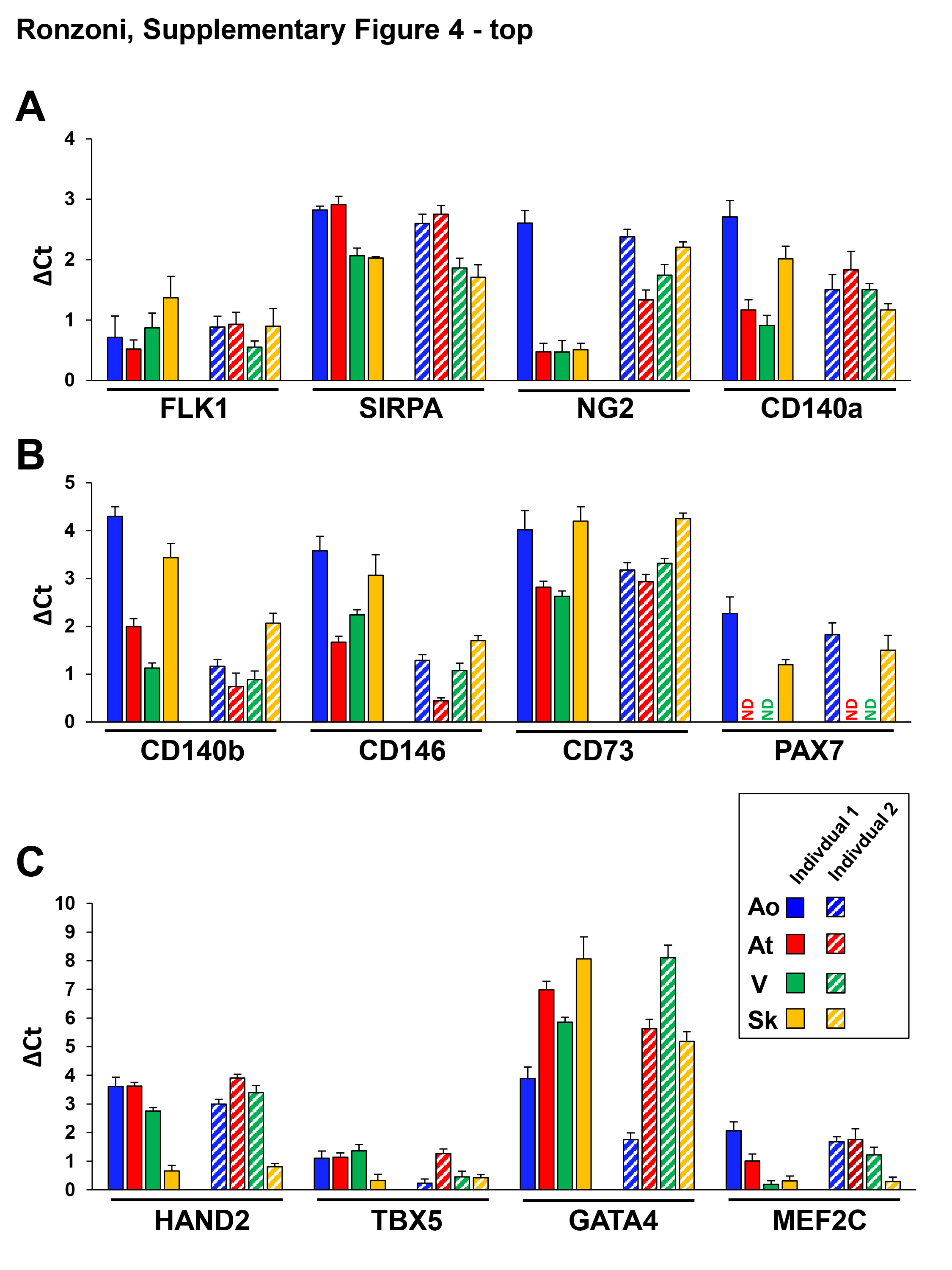

Supplement: Supplementary file 4 — Figure S4 qPCR characterization of markers present in the different fMAB populations. Typical markers are plotted separately for each individual (12 and 13 weeks of age, respectively). Ao: fMABs from aorta; At: fMABs from atria; V: fMABs from ventricles; Sk: fMABs from skeletal muscle. ND: not detectable. [file SCT3-9-575-s004.tif]

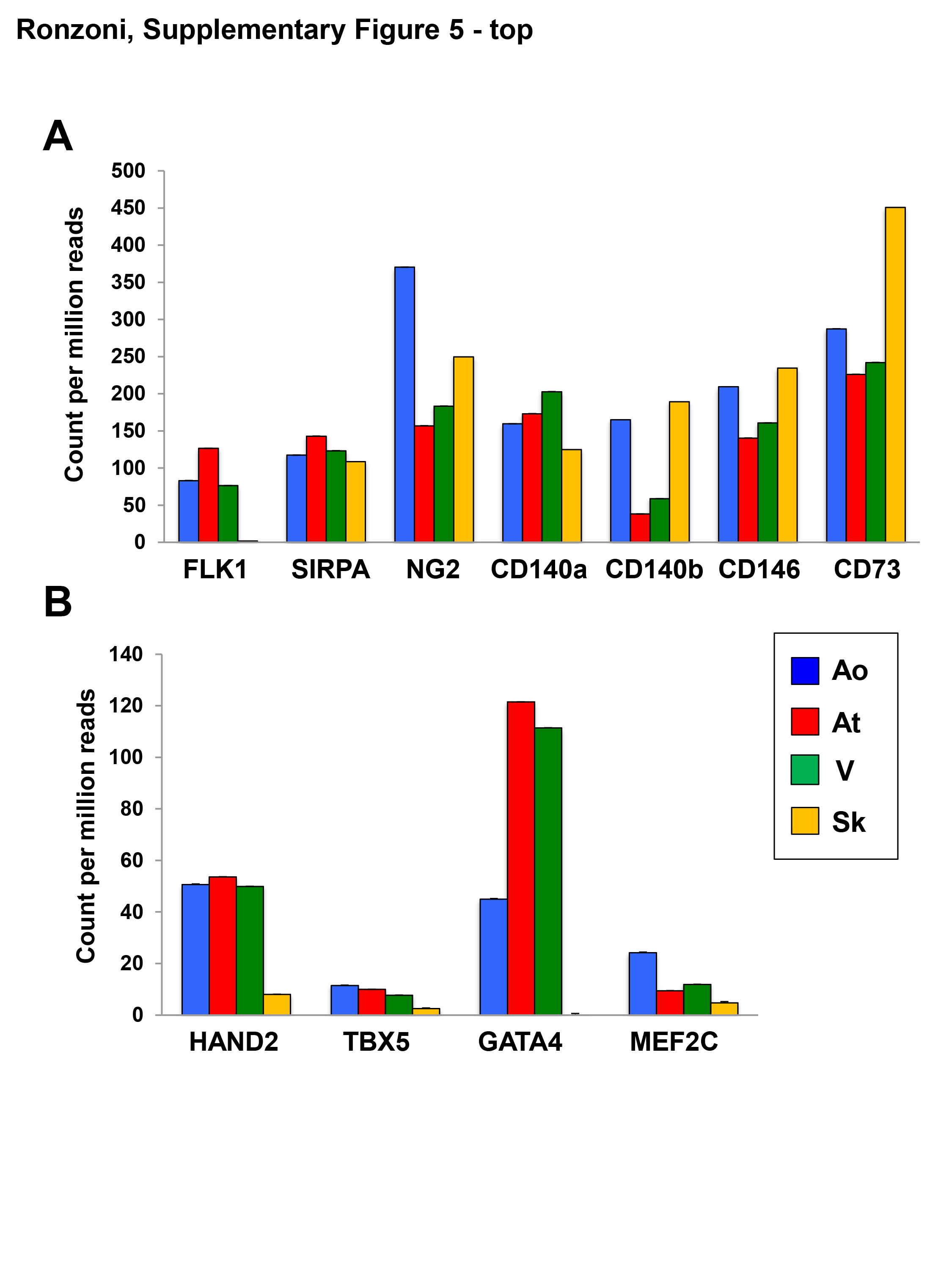

Supplement: Supplementary file 5 — Figure S5 RNA‐seq expression analysis of typical (A) and cardiac (B) fMAB markers expressed by cells derived from the four tissues. Data are consistent with qPCR characterization (see Figure 3 A, B). [file SCT3-9-575-s005.tif]

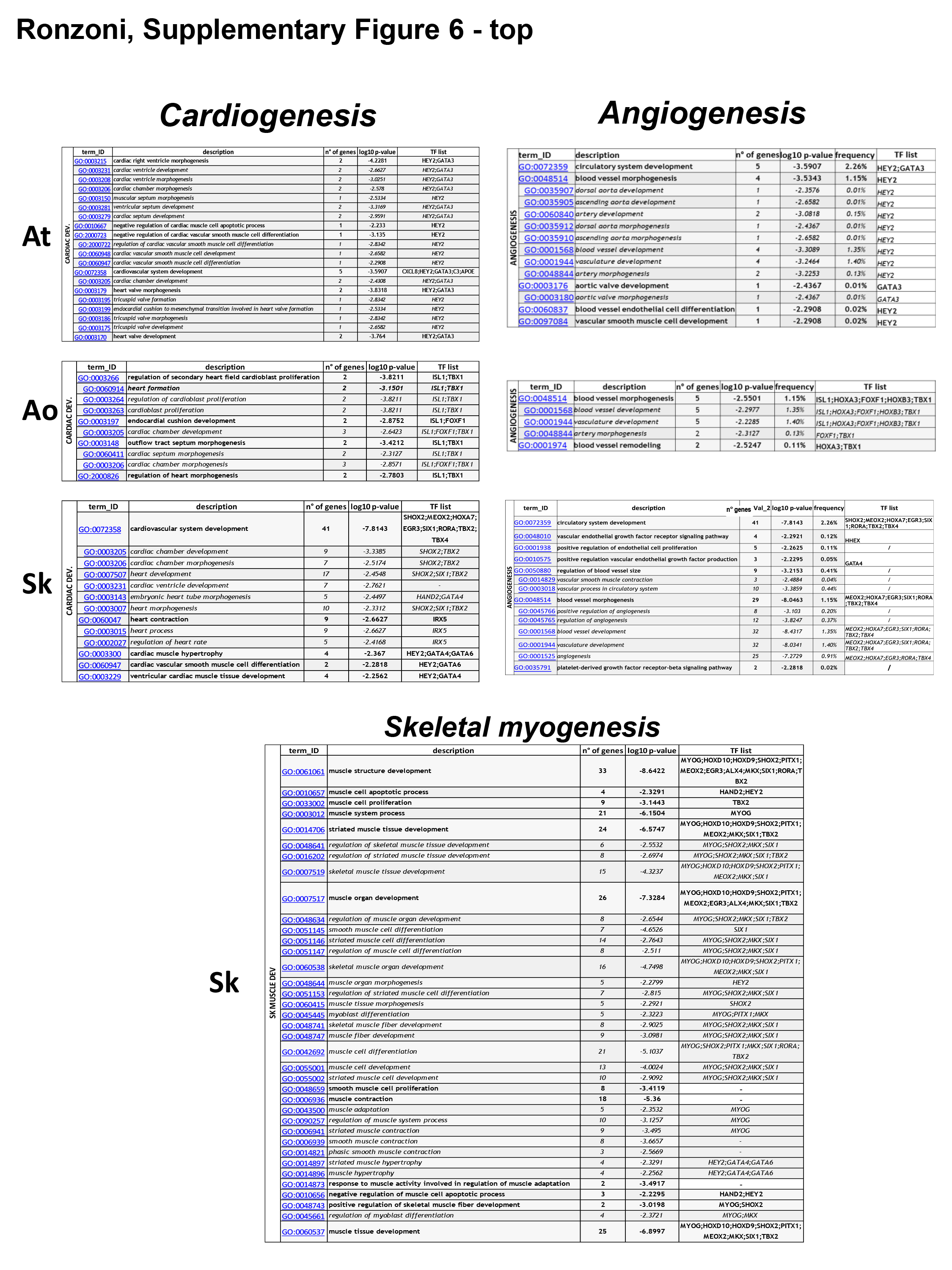

Supplement: Supplementary file 6 — Figure S6 Biological process clustering. Significant Gene Ontology analysis for three major selected Biological Processes is expressed as tables including GO terms, number of genes, log10 P‐value and the included transcription factors (TFs). Frequency indicates the percentage of human proteins in UniProt that were annotated with a GO term in the GOA database. Main representative clusters are given in black letters, while sub‐cluster members are in grey italics. TF list indicates the transcription factors belonging to that particular biological process. [file SCT3-9-575-s006.tif]
