# Supplementary material for: Human fetal mesoangioblasts reveal tissue‐dependent transcriptional signatures
Source: Stem Cells Transl Med. 2020 Jan 23;9(5):575–89. doi: 10.1002/sctm.19-0209 (PMC7180296; doi:10.1002/sctm.19-0209)
Supplement: Supplementary file 7 — Table S1 Gene clustering with the relative z‐scores calculated for the four fMAB populations. 17 clusters generated by hierarchical clustering of differentially expressed genes between Ao‐, At‐, V‐ and Sk‐fMABs (see Figure 4B). Cases highlighted in blue indicate transcription factors. [file SCT3-9-575-s007.pdf]

Supplemantary Table 1: Gene culstering with the relative z-scores calculated for the four MAB populations.

17 clusters are generated by hierarchical clustering of differentially-expressed genes between Ao, At, V and Sk-MABs (see Fig. 4B).

Cases highlited in blue indicate transcription factors.

				Z-scores				
	Gene_id	Gene_name	Gene_description	Atrium	Ventricle	Aorta	Skeletal	Cluster
1	ENSG00000179914	ITLN1	intelectin 1 (galactofuranose binding) [Source:HGNC Symbol;Acc:HGNC:1825	1.499873223	-0.481571424	-0.509150899	-0.5091509	1
2	ENSG00000039537	C6	complement component 6 [Source:HGNC Symbol;Acc:HGNC:1339]	1.496674814	-0.404778198	-0.545948308	-0.54594831	1
3	ENSG00000012171	SEMA3B	sema domain, immunoglobulin domain (Ig), short basic domain, secreted, (se	1.357002363	0.137139723	-0.855484598	-0.63865749	1
4	ENSG00000006606	CCL26	chemokine (C-C motif) ligand 26 [Source:HGNC Symbol;Acc:HGNC:10625]	1.198198789	0.449201597	-0.876490203	-0.77091018	1
5	ENSG00000076554	TPD52	tumor protein D52 [Source:HGNC Symbol;Acc:HGNC:12005]	1.166725992	0.499580205	-0.854152031	-0.81215417	1
6	ENSG00000100593	ISM2	isthmin 2 [Source:HGNC Symbol;Acc:HGNC:23176]	1.446763986	-0.218040957	-0.842891567	-0.38583146	1
7	ENSG00000103485	QPRT	quinolinate phosphoribosyltransferase [Source:HGNC Symbol;Acc:HGNC:975	0.988576017	0.21491195	-1.393052605	0.189564639	1
8	ENSG00000103710	RASL12	RAS-like, family 12 [Source:HGNC Symbol;Acc:HGNC:30289]	1.357457685	-0.055355404	-1.042528623	-0.25957366	1
9	ENSG00000106538	RARRES2	retinoic acid receptor responder (tazarotene induced) 2 [Source:HGNC Symbo	1.433711149	-0.266430698	-0.893652484	-0.27362797	1
10	ENSG00000106789	CORO2A	coronin, actin binding protein, 2A [Source:HGNC Symbol;Acc:HGNC:2255]	0.867462986	0.862135945	-0.929889206	-0.79970972	1
11	ENSG00000115318	LOXL3	lysyl oxidase-like 3 [Source:HGNC Symbol;Acc:HGNC:13869]	1.007128174	0.668501627	-1.097555926	-0.57807388	1
12	ENSG00000118849	RARRES1	retinoic acid receptor responder (tazarotene induced) 1 [Source:HGNC Symbo	1.418960751	-0.014807665	-0.718016667	-0.68613642	1
13	ENSG00000125735	TNFSF14	tumor necrosis factor (ligand) superfamily, member 14 [Source:HGNC Symbol	1.227580635	0.380583747	-0.970072338	-0.63809204	1
14	ENSG00000125845	BMP2	bone morphogenetic protein 2 [Source:HGNC Symbol;Acc:HGNC:1069]	1.09952678	0.443855184	-1.220589351	-0.32279261	1
15	ENSG00000130203	APOE	apolipoprotein E [Source:HGNC Symbol;Acc:HGNC:613]	1.494867201	-0.421251735	-0.612945571	-0.4606699	1
16	ENSG00000130707	ASS1	argininosuccinate synthase 1 [Source:HGNC Symbol;Acc:HGNC:758]	1.125512393	0.457533761	-1.159536085	-0.42351007	1
17	ENSG00000136244	IL6	interleukin 6 [Source:HGNC Symbol;Acc:HGNC:6018]	0.929772421	0.788735559	-0.994872246	-0.72363573	1
18	ENSG00000136542	GALNT5	polypeptide N-acetylgalactosaminyltransferase 5 [Source:HGNC Symbol;Acc:H	0.999689536	0.699808262	-1.032427368	-0.66707043	1
19	ENSG00000137033	IL33	interleukin 33 [Source:HGNC Symbol;Acc:HGNC:16028]	1.418688176	-0.044494146	-0.830518624	-0.54367541	1
20	ENSG00000138061	CYP1B1	cytochrome P450, family 1, subfamily B, polypeptide 1 [Source:HGNC Symbol	1.27372572	0.319892629	-0.849162652	-0.7444557	1
21	ENSG00000140450	ARRDC4	arrestin domain containing 4 [Source:HGNC Symbol;Acc:HGNC:28087]	0.936446614	0.608963469	-1.300949811	-0.24446027	1
22	ENSG00000144476	ACKR3	atypical chemokine receptor 3 [Source:HGNC Symbol;Acc:HGNC:23692]	0.968158005	0.757317583	-0.878661897	-0.84681369	1
23	ENSG00000145934	TENM2	teneurin transmembrane protein 2 [Source:HGNC Symbol;Acc:HGNC:29943]	1.468281998	-0.213831005	-0.703385294	-0.5510657	1
24	ENSG00000146013	GFRA3	GDNF family receptor alpha 3 [Source:HGNC Symbol;Acc:HGNC:4245]	1.445171095	-0.119711164	-0.759497031	-0.5659629	1
25	ENSG00000147488	ST18	suppression of tumorigenicity 18, zinc finger [Source:HGNC Symbol;Acc:HGN	1.023857224	0.686897188	-0.946339589	-0.76441482	1
26	ENSG00000158258	CLSTN2	calsyntenin 2 [Source:HGNC Symbol;Acc:HGNC:17448]	1.169500284	0.433790133	-1.083447556	-0.51984286	1
27	ENSG00000158473	CD1D	CD1d molecule [Source:HGNC Symbol;Acc:HGNC:1637]	1.21182851	0.41135581	-0.961416041	-0.66176828	1
28	ENSG00000162367	TAL1	T-cell acute lymphocytic leukemia 1 [Source:HGNC Symbol;Acc:HGNC:11556]	1.334507391	0.17766892	-0.904723921	-0.60745239	1
29	ENSG00000162722	TRIM58	tripartite motif containing 58 [Source:HGNC Symbol;Acc:HGNC:24150]	1.007248113	0.710628672	-0.908475269	-0.80940152	1
30	ENSG00000167614	TTYH1	tweety family member 1 [Source:HGNC Symbol;Acc:HGNC:13476]	1.445467308	-0.143165228	-0.796293426	-0.50600865	1
31	ENSG00000169933	FRMPD4	FERM and PDZ domain containing 4 [Source:HGNC Symbol;Acc:HGNC:29007]	1.364237434	-0.03005512	-1.019178994	-0.31500332	1
32	ENSG00000172137	CALB2	calbindin 2 [Source:HGNC Symbol;Acc:HGNC:1435]	1.406913939	-0.005318102	-0.839234619	-0.56236122	1
33	ENSG00000184254	ALDH1A3	aldehyde dehydrogenase 1 family, member A3 [Source:HGNC Symbol;Acc:HG	1.40177637	0.032713342	-0.767605944	-0.66688377	1
34	ENSG00000184347	SLIT3	slit homolog 3 (Drosophila) [Source:HGNC Symbol;Acc:HGNC:11087]	1.240116288	0.342374144	-1.006597896	-0.57589254	1
35	ENSG00000188869	TMC3	transmembrane channel-like 3 [Source:HGNC Symbol;Acc:HGNC:22995]	1.372254314	0.109069275	-0.803360293	-0.6779633	1
36	ENSG00000188910	GJB3	gap junction protein, beta 3, 31kDa [Source:HGNC Symbol;Acc:HGNC:4285]	1.258389821	0.350157066	-0.812444815	-0.79610207	1
37	ENSG00000189058	APOD	apolipoprotein D [Source:HGNC Symbol;Acc:HGNC:612]	0.82598489	0.685837035	-1.349448559	-0.16237337	1
38	ENSG00000197859	ADAMTSL2	ADAMTS-like 2 [Source:HGNC Symbol;Acc:HGNC:14631]	1.440272536	-0.170422768	-0.84742416	-0.42242561	1
39	ENSG00000221869	CEBPD	CCAAT/enhancer binding protein (C/EBP), delta [Source:HGNC Symbol;Acc:HG	0.869173715	0.861723329	-0.909988775	-0.82090827	1
40	ENSG00000243649	CFB	complement factor B [Source:HGNC Symbol;Acc:HGNC:1037]	1.459514811	-0.271946377	-0.806742302	-0.38082613	1
41	ENSG00000244255	CFB	Uncharacterized protein {ECO:0000313|Ensembl:ENSP00000410815}; cDNA F	1.461034256	-0.28077241	-0.802306256	-0.37795559	1
42	ENSG00000277669	AC009133.22	Uncharacterized protein {ECO:0000313|Ensembl:ENSP00000404873} [Source	0.924626973	0.287344817	-1.420866287	0.208894497	1
1	ENSG00000010278	CD9	CD9 molecule [Source:HGNC Symbol;Acc:HGNC:1709]	0.796713459	0.509434832	0.138352458	-1.44450075	2
2	ENSG00000008197	TFAP2D	transcription factor AP-2 delta (activating enhancer binding protein 2 delta) [S	1.300074164	0.091998331	-0.28835343	-1.10371906	2
3	ENSG00000010310	GIPR	gastric inhibitory polypeptide receptor [Source:HGNC Symbol;Acc:HGNC:4271	0.882812138	0.837468838	-0.719418498	-1.00086248	2
4	ENSG00000021300	PLEKHB1	pleckstrin homology domain containing, family B (evectins) member 1 [Sourc	1.241783106	0.369770105	-0.699256862	-0.91229635	2
5	ENSG00000021645	NRXN3	neurexin 3 [Source:HGNC Symbol;Acc:HGNC:8010]	0.60979319	0.447452436	0.438086663	-1.49533229	2
6	ENSG00000026751	SLAMF7	SLAM family member 7 [Source:HGNC Symbol;Acc:HGNC:21394]	1.318587995	0.227798478	-0.690193269	-0.85619321	2
7	ENSG00000042062	FAM65C	family with sequence similarity 65, member C [Source:HGNC Symbol;Acc:HGN	1.026356487	0.687869015	-0.811593287	-0.90263222	2
8	ENSG00000049249	TNFRSF9	tumor necrosis factor receptor superfamily, member 9 [Source:HGNC Symbol	1.199311485	0.435662394	-0.684670437	-0.95030344	2
9	ENSG00000053328	METTL24	methyltransferase like 24 [Source:HGNC Symbol;Acc:HGNC:21566]	1.091834231	0.604516194	-0.805421261	-0.89092916	2
10	ENSG00000058404	CAMK2B	calcium/calmodulin-dependent protein kinase II beta [Source:HGNC Symbol;A	0.86134179	0.848704055	-0.660519258	-1.04952659	2
11	ENSG00000070404	FSTL3	follistatin-like 3 (secreted glycoprotein) [Source:HGNC Symbol;Acc:HGNC:397	0.883585623	0.578482286	-0.092358212	-1.3697097	2
12	ENSG00000070526	ST6GALNAC1	ST6 (alpha-N-acetyl-neuraminyl-2,3-beta-galactosyl-1,3)-N-acetylgalactosami	1.374192439	0.075042696	-0.557550969	-0.89168417	2
13	ENSG00000079385	CEACAM1	carcinoembryonic antigen-related cell adhesion molecule 1 (biliary glycoprote	1.408333741	-0.221721475	-0.230393383	-0.95621888	2
14	ENSG00000080031	PTPRH	protein tyrosine phosphatase, receptor type, H [Source:HGNC Symbol;Acc:HG	0.890040767	0.74782567	-0.426963214	-1.21090322	2
15	ENSG00000081052	COL4A4	collagen, type IV, alpha 4 [Source:HGNC Symbol;Acc:HGNC:2206]	1.46721446	-0.243101229	-0.47249136	-0.75162187	2
16	ENSG00000082512	TRAF5	TNF receptor-associated factor 5 [Source:HGNC Symbol;Acc:HGNC:12035]	0.866265788	0.803905236	-0.512142534	-1.15802849	2
17	ENSG00000082684	SEMA5B	sema domain, seven thrombospondin repeats (type 1 and type 1-like), transm	1.481178197	-0.27046455	-0.600544908	-0.61016874	2
18	ENSG00000085871	MGST2	microsomal glutathione S-transferase 2 [Source:HGNC Symbol;Acc:HGNC:706	0.91070012	0.637293685	-0.241859871	-1.30613393	2
19	ENSG00000086289	EPDR1	ependymin related 1 [Source:HGNC Symbol;Acc:HGNC:17572]	0.844947945	0.840060521	-0.559013031	-1.12599544	2
20	ENSG00000087253	LPCAT2	lysophosphatidylcholine acyltransferase 2 [Source:HGNC Symbol;Acc:HGNC:2	0.758632528	0.701450503	-0.071815623	-1.38826741	2
21	ENSG00000090339	ICAM1	intercellular adhesion molecule 1 [Source:HGNC Symbol;Acc:HGNC:5344]	1.266490963	0.320139768	-0.661382366	-0.92524836	2
22	ENSG00000091490	SEL1L3	sel-1 suppressor of lin-12-like 3 (C. elegans) [Source:HGNC Symbol;Acc:HGNC:	0.838472725	0.472427655	0.123826942	-1.43472732	2
23	ENSG00000095303	PTGS1	prostaglandin-endoperoxide synthase 1 (prostaglandin G/H synthase and cycl	1.350181106	0.122849346	-0.540288749	-0.9327417	2
24	ENSG00000095637	SORBS1	sorbin and SH3 domain containing 1 [Source:HGNC Symbol;Acc:HGNC:14565]	0.993133202	0.719805058	-0.737120099	-0.97581816	2
25	ENSG00000095739	BAMBI	BMP and activin membrane-bound inhibitor [Source:HGNC Symbol;Acc:HGNC	1.054910521	0.609112948	-0.575439957	-1.08858351	2
26	ENSG00000099337	KCNK6	potassium channel, subfamily K, member 6 [Source:HGNC Symbol;Acc:HGNC:	0.956279167	0.756542583	-0.704268994	-1.00855276	2
27	ENSG00000100100	PIK3IP1	phosphoinositide-3-kinase interacting protein 1 [Source:HGNC Symbol;Acc:HG	0.748694505	0.464607952	0.255771106	-1.46907356	2
28	ENSG00000100427	MLC1	megalencephalic leukoencephalopathy with subcortical cysts 1 [Source:HGNC	0.896730074	0.824820965	-0.731013122	-0.99053792	2
29	ENSG00000100918	REC8	REC8 meiotic recombination protein [Source:HGNC Symbol;Acc:HGNC:16879]	1.069458804	0.476424307	-0.306894106	-1.238989	2
30	ENSG00000101445	PPP1R16B	protein phosphatase 1, regulatory subunit 16B [Source:HGNC Symbol;Acc:HG	0.689806068	0.575278748	0.202023992	-1.46710881	2
31	ENSG00000102010	BMX	BMX non-receptor tyrosine kinase [Source:HGNC Symbol;Acc:HGNC:1079]	0.91471718	0.78507154	-0.623714881	-1.07607384	2
32	ENSG00000102385	DRP2	dystrophin related protein 2 [Source:HGNC Symbol;Acc:HGNC:3032]	0.745210428	0.523445184	0.192087049	-1.46074266	2
33	ENSG00000102854	MSLN	mesothelin [Source:HGNC Symbol;Acc:HGNC:7371]	0.876137666	0.843085434	-0.711750626	-1.00747247	2
34	ENSG00000102996	MMP15	matrix metallopeptidase 15 (membrane-inserted) [Source:HGNC Symbol;Acc:	0.83538334	0.831272218	-0.499973013	-1.16668255	2
35	ENSG00000103888	CEMIP	cell migration inducing protein, hyaluronan binding [Source:HGNC Symbol;Ac	1.015219531	0.698726297	-0.778532766	-0.93541306	2
36	ENSG00000104368	PLAT	plasminogen activator, tissue [Source:HGNC Symbol;Acc:HGNC:9051]	1.321229018	0.153982	-0.470660147	-1.00455087	2

37	ENSG00000104369	JPH1	junctophilin 1 [Source:HGNC Symbol;Acc:HGNC:14201]	1.311446419	0.096023956	-0.32930144	-1.07816894	2
38	ENSG00000104689	TNFRSF10A	tumor necrosis factor receptor superfamily, member 10a [Source:HGNC Symt	0.994451814	0.340365169	0.041234208	-1.37605119	2
39	ENSG00000104903	LYL1	lymphoblastic leukemia associated hematopoiesis regulator 1 [Source:HGNC !	0.673111409	0.498199552	0.312323794	-1.48363475	2
40	ENSG00000105290	APLP1	amyloid beta (A4) precursor-like protein 1 [Source:HGNC Symbol;Acc:HGNC:5	0.849224355	0.70629165	-0.243831474	-1.31168453	2
41	ENSG00000105371	ICAM4	intercellular adhesion molecule 4 (Landsteiner-Wiener blood group) [Source:I	0.935792288	0.770552372	-0.65986456	-1.0464801	2
42	ENSG00000105464	GRIN2D	glutamate receptor, ionotropic, N-methyl D-aspartate 2D [Source:HGNC Symt	1.245762655	0.279631509	-0.44139664	-1.08399752	2
43	ENSG00000105612	DNASE2	deoxyribonuclease II, lysosomal [Source:HGNC Symbol;Acc:HGNC:2960]	0.79474277	0.70173463	-0.133328082	-1.36314932	2
44	ENSG00000105655	ISYNA1	inositol-3-phosphate synthase 1 [Source:HGNC Symbol;Acc:HGNC:29821]	0.707348979	0.680309182	0.038987149	-1.42664531	2
45	ENSG00000105695	MAG	myelin associated glycoprotein [Source:HGNC Symbol;Acc:HGNC:6783]	1.099972189	0.555720547	-0.592711685	-1.06298105	2
46	ENSG00000105963	ADAP1	ArfGAP with dual PH domains 1 [Source:HGNC Symbol;Acc:HGNC:16486]	1.099968204	0.573677875	-0.663015606	-1.01063047	2
47	ENSG00000106537	TSPAN13	tetraspanin 13 [Source:HGNC Symbol;Acc:HGNC:21643]	1.341214264	0.125679198	-0.499501416	-0.96739205	2
48	ENSG00000106631	MYL7	myosin, light chain 7, regulatory [Source:HGNC Symbol;Acc:HGNC:21719]	1.350673929	0.16486917	-0.752174619	-0.76336848	2
49	ENSG00000107485	GATA3	GATA binding protein 3 [Source:HGNC Symbol;Acc:HGNC:4172]	1.416421306	-0.008377986	-0.669470985	-0.73857234	2
50	ENSG00000107738	C10orf54	chromosome 10 open reading frame 54 [Source:HGNC Symbol;Acc:HGNC:30C	1.189142499	0.46041008	-0.742767698	-0.90678488	2
51	ENSG00000107796	ACTA2	actin, alpha 2, smooth muscle, aorta [Source:HGNC Symbol;Acc:HGNC:130]	1.070078748	0.6333977	-0.81416947	-0.88930698	2
52	ENSG00000107968	MAP3K8	mitogen-activated protein kinase kinase kinase 8 [Source:HGNC Symbol;Acc:H	0.844614491	0.696450303	-0.216392078	-1.32467272	2
53	ENSG00000108370	RGS9	regulator of G-protein signaling 9 [Source:HGNC Symbol;Acc:HGNC:10004]	0.945117944	0.774408745	-0.740061316	-0.97946537	2
54	ENSG00000108576	SLC6A4	solute carrier family 6 (neurotransmitter transporter), member 4 [Source:HGI	1.111616063	0.528889127	-0.556664489	-1.0838407	2
55	ENSG00000108679	LGALS3BP	lectin, galactoside-binding, soluble, 3 binding protein [Source:HGNC Symbol;A	0.982378254	0.607893551	-0.347410434	-1.24286137	2
56	ENSG00000108691	CCL2	chemokine (C-C motif) ligand 2 [Source:HGNC Symbol;Acc:HGNC:10618]	0.995687651	0.707304631	-0.680857775	-1.02213451	2
57	ENSG00000109072	VTN	vitronectin [Source:HGNC Symbol;Acc:HGNC:12724]	1.456005947	-0.160053772	-0.562292212	-0.73365996	2
58	ENSG00000110888	CAPRIN2	caprin family member 2 [Source:HGNC Symbol;Acc:HGNC:21259]	0.783815022	0.587920747	0.05546662	-1.42720239	2
59	ENSG00000111341	MGP	matrix Gla protein [Source:HGNC Symbol;Acc:HGNC:7060]	1.498152269	-0.451725534	-0.478607516	-0.56781922	2
60	ENSG00000111912	NCOA7	nuclear receptor coactivator 7 [Source:HGNC Symbol;Acc:HGNC:21081]	0.833346604	0.756566148	-0.310585743	-1.27932701	2
61	ENSG00000112936	C7	complement component 7 [Source:HGNC Symbol;Acc:HGNC:1346]	1.498138999	-0.4309697	-0.5191019	-0.5480674	2
62	ENSG00000113361	CDH6	cadherin 6, type 2, K-cadherin (fetal kidney) [Source:HGNC Symbol;Acc:HGNC	0.935098995	0.293140568	0.187557293	-1.41579686	2
63	ENSG00000113657	DPYSL3	dihydropyrimidinase-like 3 [Source:HGNC Symbol;Acc:HGNC:3015]	0.806580935	0.760778398	-0.262941833	-1.3044175	2
64	ENSG00000113739	STC2	stanniocalcin 2 [Source:HGNC Symbol;Acc:HGNC:11374]	1.015184553	0.686702542	-0.693415613	-1.00847148	2
65	ENSG00000114638	UPK1B	uroplakin 1B [Source:HGNC Symbol;Acc:HGNC:12578]	1.344000856	0.179968329	-0.757980897	-0.76598829	2
66	ENSG00000114790	ARHGEF26	Rho guanine nucleotide exchange factor (GEF) 26 [Source:HGNC Symbol;Acc:I	0.644815798	0.544267934	0.294565817	-1.48364955	2
67	ENSG00000114812	VIPR1	vasoactive intestinal peptide receptor 1 [Source:HGNC Symbol;Acc:HGNC:126	1.237608805	0.382614395	-0.741667964	-0.87855524	2
68	ENSG00000115008	IL1A	interleukin 1, alpha [Source:HGNC Symbol;Acc:HGNC:5991]	1.132578852	0.493059571	-0.536407145	-1.08923128	2
69	ENSG00000115183	TANC1	tetratricopeptide repeat, ankyrin repeat and coiled-coil containing 1 [Source:I	0.908706614	0.757953448	-0.50861402	-1.15804604	2
70	ENSG00000115232	ITGA4	integrin, alpha 4 (antigen CD49D, alpha 4 subunit of VLA-4 receptor) [Source:I	0.890790743	0.804751435	-0.601361529	-1.09418065	2
71	ENSG00000115271	GCA	grancalcin, EF-hand calcium binding protein [Source:HGNC Symbol;Acc:HGNC	0.68632281	0.649297233	0.111751924	-1.44737197	2
72	ENSG00000116096	SPR	sepiapterin reductase (7,8-dihydrobiopterin:NADP+ oxidoreductase) [Source:	0.736428404	0.669677056	0.011313971	-1.41741943	2
73	ENSG00000116544	DLGAP3	discs, large (Drosophila) homolog-associated protein 3 [Source:HGNC Symbol	1.404741858	-0.051496131	-0.443801621	-0.90944411	2
74	ENSG00000116991	SIPA1L2	signal-induced proliferation-associated 1 like 2 [Source:HGNC Symbol;Acc:HG	0.846809344	0.676299086	-0.18466435	-1.33844408	2
75	ENSG00000117298	ECE1	endothelin converting enzyme 1 [Source:HGNC Symbol;Acc:HGNC:3146]	0.799923071	0.714205009	-0.163876731	-1.35025135	2
76	ENSG00000118004	COLEC11	collectin sub-family member 11 [Source:HGNC Symbol;Acc:HGNC:17213]	1.231193368	0.369930264	-0.619570037	-0.98155359	2
77	ENSG00000118137	APOA1	apolipoprotein A-I [Source:HGNC Symbol;Acc:HGNC:600]	1.119015323	0.471427462	-0.43410733	-1.15633545	2
78	ENSG00000118515	SGK1	serum/glucocorticoid regulated kinase 1 [Source:HGNC Symbol;Acc:HGNC:10	1.23284998	0.157036532	-0.200206865	-1.18967965	2
79	ENSG00000118523	CTGF	connective tissue growth factor [Source:HGNC Symbol;Acc:HGNC:2500]	0.871577779	0.563951464	-0.049955212	-1.38557403	2
80	ENSG00000118640	VAMP8	vesicle-associated membrane protein 8 [Source:HGNC Symbol;Acc:HGNC:126	0.918052316	0.81162899	-0.829206278	-0.90047503	2
81	ENSG00000119514	GALNT12	polypeptide N-acetylgalactosaminyltransferase 12 [Source:HGNC Symbol;Acc	1.472513411	-0.354041939	-0.358151994	-0.76031948	2
82	ENSG00000120594	PLXDC2	plexin domain containing 2 [Source:HGNC Symbol;Acc:HGNC:21013]	0.951921288	0.650138219	-0.360649903	-1.2414096	2
83	ENSG00000121039	RDH10	retinol dehydrogenase 10 (all-trans) [Source:HGNC Symbol;Acc:HGNC:19975]	1.067539734	0.570547731	-0.508263318	-1.12982415	2
84	ENSG00000121966	CXCR4	chemokine (C-X-C motif) receptor 4 [Source:HGNC Symbol;Acc:HGNC:2561]	1.232238635	0.017005342	-0.032594325	-1.21664965	2
85	ENSG00000123243	ITIH5	inter-alpha-trypsin inhibitor heavy chain family, member 5 [Source:HGNC Syn	1.446250006	-0.139345975	-0.521269287	-0.78563474	2
86	ENSG00000123342	MMP19	matrix metallopeptidase 19 [Source:HGNC Symbol;Acc:HGNC:7165]	0.930132585	0.695251978	-0.4061578	-1.21922676	2
87	ENSG00000123453	SARDH	sarcosine dehydrogenase [Source:HGNC Symbol;Acc:HGNC:10536]	0.886407749	0.658766317	-0.231049041	-1.31412503	2
88	ENSG00000123977	DAW1	dynein assembly factor with WDR repeat domains 1 [Source:HGNC Symbol;Ac	1.210768447	0.421517171	-0.706177196	-0.92610842	2
89	ENSG00000125538	IL1B	interleukin 1, beta [Source:HGNC Symbol;Acc:HGNC:5992]	1.376441601	0.040861985	-0.485757101	-0.93154649	2
90	ENSG00000125730	C3	complement component 3 [Source:HGNC Symbol;Acc:HGNC:1318]	1.461046883	-0.16784244	-0.624443064	-0.66876138	2
91	ENSG00000125872	LRRN4	leucine rich repeat neuronal 4 [Source:HGNC Symbol;Acc:HGNC:16208]	1.222917262	0.37930615	-0.604846217	-0.9973772	2
92	ENSG00000126785	RHOJ	ras homolog family member J [Source:HGNC Symbol;Acc:HGNC:688]	0.834670986	0.52685514	0.060476207	-1.42200233	2
93	ENSG00000128918	ALDH1A2	aldehyde dehydrogenase 1 family, member A2 [Source:HGNC Symbol;Acc:HG	0.898688757	0.814918393	-0.683538172	-1.03006898	2
94	ENSG00000129521	EGLN3	egl-9 family hypoxia-inducible factor 3 [Source:HGNC Symbol;Acc:HGNC:1466	0.904439769	0.647139888	-0.246876342	-1.30470332	2
95	ENSG00000129538	RNASE1	ribonuclease, RNase A family, 1 (pancreatic) [Source:HGNC Symbol;Acc:HGNC	1.256646976	0.352654442	-0.776832593	-0.83246882	2
96	ENSG00000129667	RHBDF2	rhomboid 5 homolog 2 (Drosophila) [Source:HGNC Symbol;Acc:HGNC:20788]	0.837578198	0.796407868	-0.411257874	-1.22272819	2
97	ENSG00000130700	GATA5	GATA binding protein 5 [Source:HGNC Symbol;Acc:HGNC:15802]	1.170899415	0.214000198	-0.133779508	-1.25112011	2
98	ENSG00000130702	LAMA5	laminin, alpha 5 [Source:HGNC Symbol;Acc:HGNC:6485]	0.984787521	0.599246698	-0.335664871	-1.24836935	2
99	ENSG00000131477	RAMP2	receptor (G protein-coupled) activity modifying protein 2 [Source:HGNC Symt	1.185776434	0.202713307	-0.151617151	-1.23687259	2
100	ENSG00000132164	SLC6A11	solute carrier family 6 (neurotransmitter transporter), member 11 [Source:HG	1.194361699	0.456060176	-0.783002736	-0.86741914	2
101	ENSG00000132321	IQCA1	IQ motif containing with AAA domain 1 [Source:HGNC Symbol;Acc:HGNC:261!	1.10377394	0.581743216	-0.736048976	-0.94946818	2
102	ENSG00000134470	IL15RA	interleukin 15 receptor, alpha [Source:HGNC Symbol;Acc:HGNC:5978]	0.948880086	0.631255575	-0.314290242	-1.26584542	2
103	ENSG00000135127	CCDC64	coiled-coil domain containing 64 [Source:HGNC Symbol;Acc:HGNC:28095]	1.207925	0.323181285	-0.402009594	-1.12909669	2
104	ENSG00000135312	HTR1B	5-hydroxytryptamine (serotonin) receptor 1B, G protein-coupled [Source:HGN	0.868119553	0.789525927	-0.475890038	-1.18175544	2
105	ENSG00000135604	STX11	syntaxin 11 [Source:HGNC Symbol;Acc:HGNC:11429]	0.93105815	0.378656412	0.097497	-1.40721156	2
106	ENSG00000135919	SERPINE2	serpin peptidase inhibitor, clade E (nexin, plasminogen activator inhibitor typ	1.345334275	0.124703157	-0.518201066	-0.95183637	2
107	ENSG00000136205	TNS3	tensin 3 [Source:HGNC Symbol;Acc:HGNC:21616]	0.803721636	0.63611303	-0.04433247	-1.3955022	2
108	ENSG00000136275	C7orf69	chromosome 7 open reading frame 69 [Source:HGNC Symbol;Acc:HGNC:2191	1.174709024	0.421288517	-0.507335203	-1.08866234	2
109	ENSG00000136574	GATA4	GATA binding protein 4 [Source:HGNC Symbol;Acc:HGNC:4173]	0.888476814	0.739362655	-0.402203118	-1.22563635	2
110	ENSG00000136943	CTSV	cathepsin V [Source:HGNC Symbol;Acc:HGNC:2538]	0.964402382	0.685469652	-0.479233442	-1.17063859	2
111	ENSG00000137834	SMAD6	SMAD family member 6 [Source:HGNC Symbol;Acc:HGNC:6772]	0.929324467	0.767114432	-0.61483242	-1.08160648	2
112	ENSG00000138411	HECW2	HECT, C2 and WW domain containing E3 ubiquitin protein ligase 2 [Source:HG	1.04817159	0.585205168	-0.481344219	-1.15203254	2
113	ENSG00000138759	FRAS1	Fraser extracellular matrix complex subunit 1 [Source:HGNC Symbol;Acc:HGN	1.132290048	0.550074736	-0.83219917	-0.85016561	2
114	ENSG00000138835	RGS3	regulator of G-protein signaling 3 [Source:HGNC Symbol;Acc:HGNC:9999]	0.752157145	0.572420039	0.121721157	-1.44629834	2
115	ENSG00000139182	CLSTN3	calsyntenin 3 [Source:HGNC Symbol;Acc:HGNC:18371]	0.951899462	0.66882977	-0.402214808	-1.21851442	2
116	ENSG00000139209	SLC38A4	solute carrier family 38, member 4 [Source:HGNC Symbol;Acc:HGNC:14679]	0.748023463	0.665219896	0.000242873	-1.41348623	2
117	ENSG00000140465	CYP1A1	cytochrome P450, family 1, subfamily A, polypeptide 1 [Source:HGNC Symbol	1.411657181	-0.050668481	-0.4823636	-0.8786251	2
118	ENSG00000140545	MFGE8	milk fat globule-EGF factor 8 protein [Source:HGNC Symbol;Acc:HGNC:7036]	0.932697939	0.751275562	-0.570163088	-1.11381041	2
119	ENSG00000140873	ADAMTS18	ADAM metallopeptidase with thrombospondin type 1 motif, 18 [Source:HGN(	1.475160408	-0.242911012	-0.562848412	-0.66940098	2
120	ENSG00000141449	GREB1L	growth regulation by estrogen in breast cancer-like [Source:HGNC Symbol;Ac	1.0490145	0.393274775	-0.127509921	-1.31477935	2

121	ENSG00000141655	TNFRSF11A	tumor necrosis factor receptor superfamily, member 11a, NFKB activator [Sou	0.903758521	0.738585691	-0.440998161	-1.20134605	2
122	ENSG00000142408	CACNG8	calcium channel, voltage-dependent, gamma subunit 8 [Source:HGNC Symbo	0.691422043	0.617585104	0.14667504	-1.45568219	2
123	ENSG00000142798	HSPG2	heparan sulfate proteoglycan 2 [Source:HGNC Symbol;Acc:HGNC:5273]	0.991524745	0.557694477	-0.271710291	-1.27750893	2
124	ENSG00000142910	TINAGL1	tubulointerstitial nephritis antigen-like 1 [Source:HGNC Symbol;Acc:HGNC:19	0.983890987	0.620422315	-0.377819466	-1.22649384	2
125	ENSG00000143107	FNDC7	fibronectin type III domain containing 7 [Source:HGNC Symbol;Acc:HGNC:266	1.001168654	0.699859226	-0.67571339	-1.02531449	2
126	ENSG00000143367	TUFT1	tuftelin 1 [Source:HGNC Symbol;Acc:HGNC:12422]	0.93042289	0.755741932	-0.577007727	-1.10915709	2
127	ENSG00000143375	CGN	cingulin [Source:HGNC Symbol;Acc:HGNC:17429]	1.017437775	0.678130302	-0.664093921	-1.03147416	2
128	ENSG00000143595	AQP10	aquaporin 10 [Source:HGNC Symbol;Acc:HGNC:16029]	1.403735408	-0.142743863	-0.303309849	-0.9576817	2
129	ENSG00000143869	GDF7	growth differentiation factor 7 [Source:HGNC Symbol;Acc:HGNC:4222]	0.935115889	0.790972271	-0.791521457	-0.9345667	2
130	ENSG00000145242	EPHA5	EPH receptor A5 [Source:HGNC Symbol;Acc:HGNC:3389]	0.518280413	0.495260305	0.486323355	-1.49986407	2
131	ENSG00000145247	OCIAD2	OCIA domain containing 2 [Source:HGNC Symbol;Acc:HGNC:28685]	0.917245254	0.586064594	-0.166326305	-1.33698354	2
132	ENSG00000145506	NKD2	naked cuticle homolog 2 (Drosophila) [Source:HGNC Symbol;Acc:HGNC:17046	1.021030467	0.690089401	-0.762533583	-0.94858628	2
133	ENSG00000147883	CDKN2B	cyclin-dependent kinase inhibitor 2B (p15, inhibits CDK4) [Source:HGNC Symb	0.910302115	0.752722381	-0.498395271	-1.16462922	2
134	ENSG00000148426	PROSER2	proline and serine rich 2 [Source:HGNC Symbol;Acc:HGNC:23728]	1.220418	0.306423463	-0.409117089	-1.11772437	2
135	ENSG00000148948	LRRC4C	leucine rich repeat containing 4C [Source:HGNC Symbol;Acc:HGNC:29317]	1.003044862	0.371430179	-0.012200556	-1.36227449	2
136	ENSG00000149591	TAGLN	transgelin [Source:HGNC Symbol;Acc:HGNC:11553]	0.944995219	0.772344218	-0.724802112	-0.99253733	2
137	ENSG00000150394	CDH8	cadherin 8, type 2 [Source:HGNC Symbol;Acc:HGNC:1767]	0.903408862	0.792925881	-0.606977411	-1.08935733	2
138	ENSG00000151025	GPR158	G protein-coupled receptor 158 [Source:HGNC Symbol;Acc:HGNC:23689]	0.997581583	0.701646215	-0.664092983	-1.03513481	2
139	ENSG00000151320	AKAP6	A kinase (PRKA) anchor protein 6 [Source:HGNC Symbol;Acc:HGNC:376]	1.425311804	-0.116030688	-0.433377569	-0.87590355	2
140	ENSG00000151572	ANO4	anoctamin 4 [Source:HGNC Symbol;Acc:HGNC:23837]	1.266500447	0.17258094	-0.312899072	-1.12618231	2
141	ENSG00000151693	ASAP2	ArfGAP with SH3 domain, ankyrin repeat and PH domain 2 [Source:HGNC Sym	0.684557383	0.649450888	0.113958636	-1.44796691	2
142	ENSG00000152154	TMEM178A	transmembrane protein 178A [Source:HGNC Symbol;Acc:HGNC:28517]	1.25905273	0.345391414	-0.737528614	-0.86691553	2
143	ENSG00000152661	GJA1	gap junction protein, alpha 1, 43kDa [Source:HGNC Symbol;Acc:HGNC:4274]	0.891697956	0.7989668	-0.58327022	-1.10739454	2
144	ENSG00000153283	CD96	CD96 molecule [Source:HGNC Symbol;Acc:HGNC:16892]	1.256648548	0.085326018	-0.164476657	-1.17749791	2
145	ENSG00000153294	GPR115	G protein-coupled receptor 115 [Source:HGNC Symbol;Acc:HGNC:19011]	1.147645373	0.520406213	-0.731826417	-0.93622517	2
146	ENSG00000154529	CNTNAP3B	contactin associated protein-like 3B [Source:HGNC Symbol;Acc:HGNC:32035]	0.688529796	0.648416857	0.109912542	-1.4468592	2
147	ENSG00000154553	PDLIM3	PDZ and LIM domain 3 [Source:HGNC Symbol;Acc:HGNC:20767]	0.924759755	0.76975676	-0.605803485	-1.08871303	2
148	ENSG00000154556	SORBS2	sorbin and SH3 domain containing 2 [Source:HGNC Symbol;Acc:HGNC:24098]	0.974967617	0.723879733	-0.646451855	-1.0523955	2
149	ENSG00000155052	CNTNAP5	contactin associated protein-like 5 [Source:HGNC Symbol;Acc:HGNC:18748]	0.973717631	0.610514879	-0.331546371	-1.25268614	2
150	ENSG00000155465	SLC7A7	solute carrier family 7 (amino acid transporter light chain, y+L system), memb	0.931428156	0.792386318	-0.76496049	-0.95885398	2
151	ENSG00000157765	SLC34A2	solute carrier family 34 (type II sodium/phosphate contransporter), member 2	1.266536694	0.33440915	-0.76482516	-0.83612068	2
152	ENSG00000158769	F11R	F11 receptor [Source:HGNC Symbol;Acc:HGNC:14685]	1.074099043	0.593577306	-0.606456311	-1.06122004	2
153	ENSG00000158856	DMTN	dematin actin binding protein [Source:HGNC Symbol;Acc:HGNC:3382]	0.740174685	0.517441365	0.205936488	-1.46355254	2
154	ENSG00000160179	ABCG1	ATP-binding cassette, sub-family G (WHITE), member 1 [Source:HGNC Symbo	1.298776347	0.060648675	-0.240688854	-1.11873617	2
155	ENSG00000160613	PCSK7	proprotein convertase subtilisin/kexin type 7 [Source:HGNC Symbol;Acc:HGN	0.984867353	0.725019303	-0.709123026	-1.00076363	2
156	ENSG00000161940	BCL6B	B-cell CLL/lymphoma 6, member B [Source:HGNC Symbol;Acc:HGNC:1002]	0.885041023	0.610729992	-0.145786762	-1.34998425	2
157	ENSG00000162267	ITIH3	inter-alpha-trypsin inhibitor heavy chain 3 [Source:HGNC Symbol;Acc:HGNC:6	0.96228222	0.757001139	-0.752416281	-0.96686708	2
158	ENSG00000162496	DHRS3	dehydrogenase/reductase (SDR family) member 3 [Source:HGNC Symbol;Acc:	1.070240354	0.632706384	-0.805351677	-0.89759506	2
159	ENSG00000162522	KIAA1522	KIAA1522 [Source:HGNC Symbol;Acc:HGNC:29301]	0.757087977	0.688654248	-0.04926595	-1.39647628	2
160	ENSG00000162552	WNT4	wingless-type MMTV integration site family, member 4 [Source:HGNC Symbo	1.284195355	0.244706926	-0.5172774	-1.01162488	2
161	ENSG00000162878	PKDCC	protein kinase domain containing, cytoplasmic [Source:HGNC Symbol;Acc:HG	0.968264435	0.725219534	-0.620272745	-1.07321122	2
162	ENSG00000163283	ALPP	alkaline phosphatase, placental [Source:HGNC Symbol;Acc:HGNC:439]	1.234273435	0.391736529	-0.788949002	-0.83706096	2
163	ENSG00000163293	NIPAL1	NIPA-like domain containing 1 [Source:HGNC Symbol;Acc:HGNC:27194]	0.985718348	0.514552325	-0.185250747	-1.31501993	2
164	ENSG00000163431	LMOD1	leiomodin 1 (smooth muscle) [Source:HGNC Symbol;Acc:HGNC:6647]	0.937018745	0.790777705	-0.819030609	-0.90876584	2
165	ENSG00000163694	RBM47	RNA binding motif protein 47 [Source:HGNC Symbol;Acc:HGNC:30358]	0.892542264	0.815531638	-0.654640078	-1.05343382	2
166	ENSG00000163874	ZC3H12A	zinc finger CCCH-type containing 12A [Source:HGNC Symbol;Acc:HGNC:26259	1.11223609	0.535045449	-0.578805098	-1.06847644	2
167	ENSG00000163898	LIPH	lipase, member H [Source:HGNC Symbol;Acc:HGNC:18483]	0.942755544	0.785440333	-0.84213236	-0.88606352	2
168	ENSG00000163947	ARHGEF3	Rho guanine nucleotide exchange factor (GEF) 3 [Source:HGNC Symbol;Acc:H	0.753409176	0.603326287	0.079262272	-1.43599774	2
169	ENSG00000163975	MFI2	antigen p97 (melanoma associated) identified by monoclonal antibodies 133.	1.417526439	-0.021918013	-0.607590701	-0.78801773	2
170	ENSG00000164342	TLR3	toll-like receptor 3 [Source:HGNC Symbol;Acc:HGNC:11849]	0.795432818	0.716499152	-0.159750228	-1.35218174	2
171	ENSG00000164532	TBX20	T-box 20 [Source:HGNC Symbol;Acc:HGNC:11598]	0.878911895	0.746680217	-0.395239069	-1.23035304	2
172	ENSG00000164588	HCN1	hyperpolarization activated cyclic nucleotide-gated potassium channel 1 [Sou	0.955243492	0.767598216	-0.777126497	-0.94571521	2
173	ENSG00000164736	SOX17	SRY (sex determining region Y)-box 17 [Source:HGNC Symbol;Acc:HGNC:1812	0.884822451	0.552271562	-0.055423431	-1.38167058	2
174	ENSG00000164855	TMEM184A	transmembrane protein 184A [Source:HGNC Symbol;Acc:HGNC:28797]	0.89368593	0.763391838	-0.47810701	-1.17897076	2
175	ENSG00000165061	ZMAT4	zinc finger, matrin-type 4 [Source:HGNC Symbol;Acc:HGNC:25844]	1.403616491	-0.019192559	-0.503562051	-0.88086188	2
176	ENSG00000165124	SVEP1	sushi, von Willebrand factor type A, EGF and pentraxin domain containing 1 [S	1.021119087	0.687695506	-0.744028308	-0.96478629	2
177	ENSG00000165272	AQP3	aquaporin 3 (Gill blood group) [Source:HGNC Symbol;Acc:HGNC:636]	0.89622169	0.829325768	-0.76215455	-0.96339291	2
178	ENSG00000166394	CYB5R2	cytochrome b5 reductase 2 [Source:HGNC Symbol;Acc:HGNC:24376]	1.192271276	0.389279639	-0.493875712	-1.0876752	2
179	ENSG00000166689	PLEKHA7	pleckstrin homology domain containing, family A member 7 [Source:HGNC Sy	1.350757379	0.025056672	-0.349976179	-1.02583787	2
180	ENSG00000166833	NAV2	neuron navigator 2 [Source:HGNC Symbol;Acc:HGNC:15997]	0.773283699	0.582407789	0.078422655	-1.43411414	2
181	ENSG00000167191	GPRC5B	G protein-coupled receptor, class C, group 5, member B [Source:HGNC Symbo	1.040647631	0.619371287	-0.551082665	-1.10893625	2
182	ENSG00000167641	PPP1R14A	protein phosphatase 1, regulatory (inhibitor) subunit 14A [Source:HGNC Symb	0.913432117	0.80073876	-0.690850198	-1.02332068	2
183	ENSG00000168394	TAP1	transporter 1, ATP-binding cassette, sub-family B (MDR/TAP) [Source:HGNC S	0.796949497	0.747031144	-0.217518563	-1.32646208	2
184	ENSG00000168672	FAM84B	family with sequence similarity 84, member B [Source:HGNC Symbol;Acc:HGN	1.137647454	0.443508052	-0.430694082	-1.15046142	2
185	ENSG00000168824	D4S234E	Neuron-specific protein family member 1 [Source:UniProtKB/Swiss-Prot;Acc:	1.281548206	0.159415637	-0.337054971	-1.10390887	2
186	ENSG00000169067	ACTBL2	actin, beta-like 2 [Source:HGNC Symbol;Acc:HGNC:17780]	0.973116057	0.75077414	-0.820204087	-0.90368611	2
187	ENSG00000169184	MN1	meningioma (disrupted in balanced translocation) 1 [Source:HGNC Symbol;Ac	0.981579322	0.47354932	-0.113742932	-1.34138571	2
188	ENSG00000169302	STK32A	serine/threonine kinase 32A [Source:HGNC Symbol;Acc:HGNC:28317]	1.205806362	0.279995111	-0.316385351	-1.16941612	2
189	ENSG00000169418	NPR1	natriuretic peptide receptor 1 [Source:HGNC Symbol;Acc:HGNC:7943]	1.452489812	-0.281700713	-0.334850646	-0.83593845	2
190	ENSG00000169429	CXCL8	chemokine (C-X-C motif) ligand 8 [Source:HGNC Symbol;Acc:HGNC:6025]	1.488763513	-0.328600325	-0.543917265	-0.61624592	2
191	ENSG00000170458	CD14	CD14 molecule [Source:HGNC Symbol;Acc:HGNC:1628]	1.19178376	0.39417673	-0.504327482	-1.08163301	2
192	ENSG00000170542	SERPINB9	serpin peptidase inhibitor, clade B (ovalbumin), member 9 [Source:HGNC Sym	0.897629316	0.698719761	-0.333477262	-1.26287182	2
193	ENSG00000171992	SYNPO	synaptopodin [Source:HGNC Symbol;Acc:HGNC:30672]	0.997342294	0.602784557	-0.374765789	-1.22536106	2
194	ENSG00000172578	KLHL6	kelch-like family member 6 [Source:HGNC Symbol;Acc:HGNC:18653]	1.161747754	0.505814453	-0.788437748	-0.87912446	2
195	ENSG00000172602	RND1	Rho family GTPase 1 [Source:HGNC Symbol;Acc:HGNC:18314]	1.068503742	0.494789549	-0.339187552	-1.22410574	2
196	ENSG00000173714	WFIKKN2	WAP, follistatin/kazal, immunoglobulin, kunitz and netrin domain containing 2	0.914831351	0.796945527	-0.678587305	-1.03318957	2
197	ENSG00000173838	MARCH10	membrane-associated ring finger (C3HC4) 10, E3 ubiquitin protein ligase [Sou	1.004935526	0.700263118	-0.701131595	-1.00406705	2
198	ENSG00000173926	MARCH3	membrane-associated ring finger (C3HC4) 3, E3 ubiquitin protein ligase [Sourc	0.848872742	0.572059183	-0.023938617	-1.39699331	2
199	ENSG00000174059	CD34	CD34 molecule [Source:HGNC Symbol;Acc:HGNC:1662]	0.956018648	0.427994927	-0.004583374	-1.3794302	2
200	ENSG00000174175	SELP	selectin P (granule membrane protein 140kDa, antigen CD62) [Source:HGNC S	1.170843934	0.339210704	-0.322633936	-1.1874207	2
201	ENSG00000174348	PODN	podocan [Source:HGNC Symbol;Acc:HGNC:23174]	1.203506487	0.413687231	-0.617838832	-0.99935489	2
202	ENSG00000174628	IQCK	IQ motif containing K [Source:HGNC Symbol;Acc:HGNC:28556]	0.971480394	0.700497606	-0.546356706	-1.12562129	2
203	ENSG00000174938	SEZ6L2	seizure related 6 homolog (mouse)-like 2 [Source:HGNC Symbol;Acc:HGNC:30	1.142583716	0.533472938	-0.786423462	-0.88963319	2
204	ENSG00000175482	POLD4	polymerase (DNA-directed), delta 4, accessory subunit [Source:HGNC Symbol;	0.816985436	0.509600327	0.109017746	-1.43560351	2

205	ENSG00000175535	PNLIP	pancreatic lipase [Source:HGNC Symbol;Acc:HGNC:9155]	0.895566449	0.552638941	-0.074525328	-1.37368006	2
206	ENSG00000177409	SAMD9L	sterile alpha motif domain containing 9-like [Source:HGNC Symbol;Acc:HGNC	0.946994775	0.745512686	-0.606336058	-1.0861714	2
207	ENSG00000178015	GPR150	G protein-coupled receptor 150 [Source:HGNC Symbol;Acc:HGNC:23628]	0.99694455	0.441807786	-0.09651852	-1.34223382	2
208	ENSG00000178038	ALS2CL	ALS2 C-terminal like [Source:HGNC Symbol;Acc:HGNC:20605]	0.956888784	0.76864894	-0.813766592	-0.91177113	2
209	ENSG00000178568	ERBB4	v-erb-b2 avian erythroblastic leukemia viral oncogene homolog 4 [Source:HGI	1.117789562	0.463201644	-0.412194047	-1.16879716	2
210	ENSG00000178597	PSAPL1	prosaposin-like 1 (gene/pseudogene) [Source:HGNC Symbol;Acc:HGNC:33131	1.081243395	0.603859298	-0.690102787	-0.99499991	2
211	ENSG00000178718	RPP25	ribonuclease P/MRP 25kDa subunit [Source:HGNC Symbol;Acc:HGNC:30361]	1.003663696	0.463898526	-0.141924749	-1.32563747	2
212	ENSG00000178764	ZHX2	zinc fingers and homeoboxes 2 [Source:HGNC Symbol;Acc:HGNC:18513]	0.931592365	0.732127735	-0.506174342	-1.15754576	2
213	ENSG00000179546	HTR1D	5-hydroxytryptamine (serotonin) receptor 1D, G protein-coupled [Source:HGI	1.050686321	0.515205486	-0.333160963	-1.23273084	2
214	ENSG00000181013	C17orf47	chromosome 17 open reading frame 47 [Source:HGNC Symbol;Acc:HGNC:268	0.857038453	0.795161118	-0.459819719	-1.19237985	2
215	ENSG00000182836	PLCXD3	phosphatidylinositol-specific phospholipase C, X domain containing 3 [Source	1.195878966	0.173474263	-0.135943507	-1.23340972	2
216	ENSG00000183111	ARHGEF37	Rho guanine nucleotide exchange factor (GEF) 37 [Source:HGNC Symbol;Acc:I	0.855097186	0.817105411	-0.517497479	-1.15470512	2
217	ENSG00000183607	GKN2	gastrokine 2 [Source:HGNC Symbol;Acc:HGNC:24588]	1.408442222	0.01316666	-0.657742024	-0.76386686	2
218	ENSG00000184292	TACSTD2	tumor-associated calcium signal transducer 2 [Source:HGNC Symbol;Acc:HGN	1.138230669	0.225123414	-0.079853472	-1.28350061	2
219	ENSG00000184545	DUSP8	dual specificity phosphatase 8 [Source:HGNC Symbol;Acc:HGNC:3074]	0.89568035	0.794893457	-0.583675356	-1.10689845	2
220	ENSG00000184731	FAM110C	family with sequence similarity 110, member C [Source:HGNC Symbol;Acc:HG	1.146687006	0.527923661	-0.79123432	-0.88337635	2
221	ENSG00000184916	JAG2	jagged 2 [Source:HGNC Symbol;Acc:HGNC:6189]	0.981962239	0.691708112	-0.556921057	-1.11674929	2
222	ENSG00000184937	WT1	Wilms tumor 1 [Source:HGNC Symbol;Acc:HGNC:12796]	0.859800649	0.677080017	-0.211078508	-1.32580216	2
223	ENSG00000187513	GJA4	gap junction protein, alpha 4, 37kDa [Source:HGNC Symbol;Acc:HGNC:4278]	0.971970565	0.667813571	-0.455988065	-1.18379607	2
224	ENSG00000187942	LDLRAD2	low density lipoprotein receptor class A domain containing 2 [Source:HGNC S	0.986636042	0.556679255	-0.258945998	-1.2843693	2
225	ENSG00000188404	SELL	selectin L [Source:HGNC Symbol;Acc:HGNC:10720]	1.111265637	0.547879011	-0.619171764	-1.03997288	2
226	ENSG00000196083	IL1RAP	interleukin 1 receptor accessory protein [Source:HGNC Symbol;Acc:HGNC:59	1.110534995	0.443339276	-0.350967837	-1.20290643	2
227	ENSG00000196196	HRCT1	histidine rich carboxyl terminus 1 [Source:HGNC Symbol;Acc:HGNC:33872]	1.129101295	0.483526366	-0.497243826	-1.11538383	2
228	ENSG00000196562	SULF2	sulfatase 2 [Source:HGNC Symbol;Acc:HGNC:20392]	1.329737622	0.211041162	-0.758990839	-0.78178794	2
229	ENSG00000197070	ARRDC1	arrestin domain containing 1 [Source:HGNC Symbol;Acc:HGNC:28633]	0.816611625	0.624873038	-0.048529521	-1.39295514	2
230	ENSG00000197375	SLC22A5	solute carrier family 22 (organic cation/carnitine transporter), member 5 [Sou	0.937896431	0.67929034	-0.389506049	-1.22768072	2
231	ENSG00000197635	DPP4	dipeptidyl-peptidase 4 [Source:HGNC Symbol;Acc:HGNC:3009]	0.951358554	0.701817066	-0.483167594	-1.17000803	2
232	ENSG00000197991	PCDH20	protocadherin 20 [Source:HGNC Symbol;Acc:HGNC:14257]	1.263048035	0.309829341	-0.597039873	-0.9758375	2
233	ENSG00000198286	CARD11	caspase recruitment domain family, member 11 [Source:HGNC Symbol;Acc:H	0.778046569	0.486577072	0.191778529	-1.45640217	2
234	ENSG00000198515	CNGA1	cyclic nucleotide gated channel alpha 1 [Source:HGNC Symbol;Acc:HGNC:214	0.852186115	0.750819768	-0.340398521	-1.26260736	2
235	ENSG00000198873	GRK5	G protein-coupled receptor kinase 5 [Source:HGNC Symbol;Acc:HGNC:4544]	0.833078915	0.561069485	0.016843048	-1.41099145	2
236	ENSG00000204525	HLA-C	major histocompatibility complex, class I, C [Source:HGNC Symbol;Acc:HGNC:	0.915212842	0.432120661	0.057087449	-1.40442095	2
237	ENSG00000204580	DDR1	discoidin domain receptor tyrosine kinase 1 [Source:HGNC Symbol;Acc:HGNC	0.731609281	0.717738921	-0.05411702	-1.39523118	2
238	ENSG00000204644	ZFP57	ZFP57 zinc finger protein [Source:HGNC Symbol;Acc:HGNC:18791]	0.921004743	0.794772397	-0.702741056	-1.01303608	2
239	ENSG00000204961	PCDHA9	protocadherin alpha 9 [Source:HGNC Symbol;Acc:HGNC:8675]	1.0967596	0.591914681	-0.742001963	-0.94667232	2
240	ENSG00000204962	PCDHA8	protocadherin alpha 8 [Source:HGNC Symbol;Acc:HGNC:8674]	1.106857818	0.577222688	-0.733375252	-0.95070525	2
241	ENSG00000204965	PCDHA5	protocadherin alpha 5 [Source:HGNC Symbol;Acc:HGNC:8671]	1.102881018	0.58383507	-0.742649334	-0.94406675	2
242	ENSG00000204969	PCDHA2	protocadherin alpha 2 [Source:HGNC Symbol;Acc:HGNC:8668]	1.107822484	0.577129123	-0.742175357	-0.94277625	2
243	ENSG00000204970	PCDHA1	protocadherin alpha 1 [Source:HGNC Symbol;Acc:HGNC:8663]	1.1298489	0.536966137	-0.68179511	-0.98501993	2
244	ENSG00000205038	PKHD1L1	polycystic kidney and hepatic disease 1 (autosomal recessive)-like 1 [Source:H	1.336054146	0.099732704	-0.422706391	-1.01308046	2
245	ENSG00000205089	CCNI2	cyclin I family, member 2 [Source:HGNC Symbol;Acc:HGNC:33869]	0.663882614	0.651745614	0.138756258	-1.45438449	2
246	ENSG00000205413	SAMD9	sterile alpha motif domain containing 9 [Source:HGNC Symbol;Acc:HGNC:134	0.865752159	0.686170755	-0.239579817	-1.3123431	2
247	ENSG00000215252	GOLGA8B	golgin A8 family, member B [Source:HGNC Symbol;Acc:HGNC:31973]	0.969563491	0.611620585	-0.323796924	-1.25738715	2
248	ENSG00000238243	OR2W3	olfactory receptor, family 2, subfamily W, member 3 [Source:HGNC Symbol;A	1.052830679	0.653173458	-0.783407041	-0.9225971	2
249	ENSG00000239389	PCDHA13	protocadherin alpha 13 [Source:HGNC Symbol;Acc:HGNC:8667]	1.103599702	0.583384439	-0.746609287	-0.94037485	2
250	ENSG00000243232	PCDHAC2	protocadherin alpha subfamily C, 2 [Source:HGNC Symbol;Acc:HGNC:8677]	1.038526968	0.64445301	-0.629438993	-1.05354099	2
251	ENSG00000243566	UPK3B	uroplakin 3B [Source:HGNC Symbol;Acc:HGNC:21444]	1.449011671	-0.117780998	-0.651380511	-0.67985016	2
252	ENSG00000248383	PCDHAC1	protocadherin alpha subfamily C, 1 [Source:HGNC Symbol;Acc:HGNC:8676]	1.121310662	0.559959224	-0.751826578	-0.92944331	2
253	ENSG00000249158	PCDHA11	protocadherin alpha 11 [Source:HGNC Symbol;Acc:HGNC:8665]	1.072882255	0.624024741	-0.749029877	-0.94787712	2
254	ENSG00000251012	RP11-484M3.5		1.448470358	-0.115858265	-0.649232837	-0.68337926	2
255	ENSG00000251664	PCDHA12	protocadherin alpha 12 [Source:HGNC Symbol;Acc:HGNC:8666]	1.095355719	0.593908857	-0.742993927	-0.94627065	2
256	ENSG00000256463	SALL3	spalt-like transcription factor 3 [Source:HGNC Symbol;Acc:HGNC:10527]	1.226301774	0.168868525	-0.200384336	-1.19478596	2
257	ENSG00000256514	AP003419.11		0.831111412	0.494847995	0.106856354	-1.43281576	2
258	ENSG00000259471	LINC01169	long intergenic non-protein coding RNA 1169 [Source:HGNC Symbol;Acc:HGN	1.075190844	0.617271403	-0.722282675	-0.97017957	2
259	ENSG00000267060	PTGES3L	prostaglandin E synthase 3 (cytosolic)-like [Source:HGNC Symbol;Acc:HGNC:4	0.872933195	0.851913142	-0.751328186	-0.97351815	2
260	ENSG00000267110	CTD-2587H24.4		0.781378373	0.713934816	-0.130500151	-1.36481304	2
261	ENSG00000270149	RP11-544M22.13	Uncharacterized protein {ECO:0000313|Ensembl:ENSP00000473373} [Source	1.091650267	0.57184978	-0.610762105	-1.05273794	2
262	ENSG00000273171	CTB-96E2.2	Uncharacterized protein {ECO:0000313|Ensembl:ENSP00000452347} [Source	1.467524115	-0.216465445	-0.533491886	-0.71756678	2
263	ENSG00000273706	LHX1	LIM homeobox 1 [Source:HGNC Symbol;Acc:HGNC:6593]	1.267207716	0.116839657	-0.232274008	-1.15177336	2
264	ENSG00000275832	ARHGAP23	Rho GTPase activating protein 23 [Source:HGNC Symbol;Acc:HGNC:29293]	0.609085027	0.606376626	0.264808371	-1.48027002	2
1	ENSG00000164265	SCGB3A2	secretoglobin, family 3A, member 2 [Source:HGNC Symbol;Acc:HGNC:18391]	0.398989223	1.230270127	-0.814629675	-0.81462968	3
2	ENSG00000105851	PIK3CG	phosphatidylinositol-4,5-bisphosphate 3-kinase, catalytic subunit gamma [Sou	0.427340643	1.213160396	-0.820250519	-0.82025052	3
3	ENSG00000177272	KCNA3	potassium voltage-gated channel, shaker-related subfamily, member 3 [Sourc	-0.1879278	1.465713245	-0.638892724	-0.63889272	3
4	ENSG00000003400	CASP10	caspase 10, apoptosis-related cysteine peptidase [Source:HGNC Symbol;Acc:I	0.442307492	1.024285018	-0.15030505	-1.31628746	3
5	ENSG00000000971	CFH	complement factor H [Source:HGNC Symbol;Acc:HGNC:4883]	0.801557146	0.848994933	-0.454767834	-1.19578425	3
6	ENSG00000003989	SLC7A2	solute carrier family 7 (cationic amino acid transporter, y+ system), member 2	0.178851223	1.020513715	0.177275018	-1.37663996	3
7	ENSG00000004399	PLXND1	plexin D1 [Source:HGNC Symbol;Acc:HGNC:9107]	0.520538158	0.920366494	-0.070889999	-1.37001465	3
8	ENSG00000005884	ITGA3	integrin, alpha 3 (antigen CD49C, alpha 3 subunit of VLA-3 receptor) [Source:I	0.388171311	0.8056676	0.2655181	-1.45935701	3
9	ENSG00000006788	MYH13	myosin, heavy chain 13, skeletal muscle [Source:HGNC Symbol;Acc:HGNC:757	0.608083323	1.027309844	-0.473012852	-1.16238032	3
10	ENSG00000007908	SELE	selectin E [Source:HGNC Symbol;Acc:HGNC:10718]	0.180871894	1.338417099	-0.65522825	-0.86406074	3
11	ENSG00000008517	IL32	interleukin 32 [Source:HGNC Symbol;Acc:HGNC:16830]	0.692914626	1.023094157	-0.837947023	-0.87806176	3
12	ENSG00000010030	ETV7	ets variant 7 [Source:HGNC Symbol;Acc:HGNC:18160]	0.782382952	0.944661829	-0.817975132	-0.90906965	3
13	ENSG00000012124	CD22	CD22 molecule [Source:HGNC Symbol;Acc:HGNC:1643]	0.81618149	0.879936898	-0.601939265	-1.09417912	3
14	ENSG00000019505	SYT13	synaptotagmin XIII [Source:HGNC Symbol;Acc:HGNC:14962]	0.181715799	1.331635069	-0.60077164	-0.91257923	3
15	ENSG00000022267	FHL1	four and a half LIM domains 1 [Source:HGNC Symbol;Acc:HGNC:3702]	0.382895398	1.143947769	-0.328019709	-1.19882346	3
16	ENSG00000029534	ANK1	ankyrin 1, erythrocytic [Source:HGNC Symbol;Acc:HGNC:492]	0.441182676	1.174135448	-0.560538167	-1.05477996	3
17	ENSG00000039068	CDH1	cadherin 1, type 1, E-cadherin (epithelial) [Source:HGNC Symbol;Acc:HGNC:1	0.428702122	1.205888376	-0.703048444	-0.93154205	3
18	ENSG00000046653	GPM6B	glycoprotein M6B [Source:HGNC Symbol;Acc:HGNC:4461]	0.632286938	0.922403815	-0.257320012	-1.29737074	3
19	ENSG00000048052	HDAC9	histone deacetylase 9 [Source:HGNC Symbol;Acc:HGNC:14065]	0.339721426	0.949375896	0.114526041	-1.40362336	3
20	ENSG00000048740	CELF2	CUGBP, Elav-like family member 2 [Source:HGNC Symbol;Acc:HGNC:2550]	0.371484499	1.163443698	-0.361018688	-1.17390951	3
21	ENSG00000054219	LY75	lymphocyte antigen 75 [Source:HGNC Symbol;Acc:HGNC:6729]	0.747074783	0.936072739	-0.568485583	-1.11466194	3
22	ENSG00000057294	PKP2	plakophilin 2 [Source:HGNC Symbol;Acc:HGNC:9024]	0.749658183	0.968173463	-0.746710719	-0.97112093	3
23	ENSG00000059378	PARP12	poly (ADP-ribose) polymerase family, member 12 [Source:HGNC Symbol;Acc:I	0.474336816	0.641644988	0.374904751	-1.49088656	3
24	ENSG00000064042	LIMCH1	LIM and calponin homology domains 1 [Source:HGNC Symbol;Acc:HGNC:291	0.42488586	1.13691766	-0.388935483	-1.17286804	3

25	ENSG00000065054	SLC9A3R2	solute carrier family 9, subfamily A (NHE3, cation proton antiporter 3), memb	0.471757458	0.580974485	0.444662163	-1.49739411	3
26	ENSG00000065809	FAM107B	family with sequence similarity 107, member B [Source:HGNC Symbol;Acc:HG	0.405909958	0.829334982	0.214400836	-1.44964578	3
27	ENSG00000065989	PDE4A	phosphodiesterase 4A, cAMP-specific [Source:HGNC Symbol;Acc:HGNC:8780]	0.493231386	0.679044021	0.310664171	-1.48293958	3
28	ENSG00000066583	ISOC1	isochorismatase domain containing 1 [Source:HGNC Symbol;Acc:HGNC:24254	0.439369558	0.716893171	0.323130757	-1.47939349	3
29	ENSG00000067208	EVI5	ecotropic viral integration site 5 [Source:HGNC Symbol;Acc:HGNC:3501]	0.414513	1.098847157	-0.269040655	-1.2443195	3
30	ENSG00000068079	IFI35	interferon-induced protein 35 [Source:HGNC Symbol;Acc:HGNC:5399]	0.328615543	1.022053476	0.008502137	-1.35917116	3
31	ENSG00000068976	PYGM	phosphorylase, glycogen, muscle [Source:HGNC Symbol;Acc:HGNC:9726]	0.532422641	0.636041698	0.318353737	-1.48681808	3
32	ENSG00000070159	PTPN3	protein tyrosine phosphatase, non-receptor type 3 [Source:HGNC Symbol;Acc	0.642396078	0.820596728	-0.081967992	-1.38102481	3
33	ENSG00000072163	LIMS2	LIM and senescent cell antigen-like domains 2 [Source:HGNC Symbol;Acc:HGN	0.795465002	0.930517195	-0.785965426	-0.94001677	3
34	ENSG00000072952	MRVI1	murine retrovirus integration site 1 homolog [Source:HGNC Symbol;Acc:HGN	0.66043582	1.048772285	-0.814572512	-0.89463559	3
35	ENSG00000073146	MOV10L1	Mov10 RISC complex RNA helicase like 1 [Source:HGNC Symbol;Acc:HGNC:72	0.54464444	0.969029149	-0.20062592	-1.31304767	3
36	ENSG00000073464	CLCN4	chloride channel, voltage-sensitive 4 [Source:HGNC Symbol;Acc:HGNC:2022]	0.451910423	0.877145935	0.091513016	-1.42056937	3
37	ENSG00000073910	FRY	furry homolog (Drosophila) [Source:HGNC Symbol;Acc:HGNC:20367]	0.339669291	1.217121325	-0.469007248	-1.08778337	3
38	ENSG00000074527	NTN4	netrin 4 [Source:HGNC Symbol;Acc:HGNC:13658]	0.567512645	0.98992026	-0.2861778	-1.27125511	3
39	ENSG00000074590	NUAK1	NUAK family, SNF1-like kinase, 1 [Source:HGNC Symbol;Acc:HGNC:14311]	0.782732284	0.840346709	-0.384951362	-1.23812763	3
40	ENSG00000076716	GPC4	glypican 4 [Source:HGNC Symbol;Acc:HGNC:4452]	0.802995675	0.923803729	-0.789689025	-0.93711038	3
41	ENSG00000076944	STXBP2	syntaxin binding protein 2 [Source:HGNC Symbol;Acc:HGNC:11445]	0.809146942	0.877158679	-0.565609522	-1.1206961	3
42	ENSG00000077092	RARB	retinoic acid receptor, beta [Source:HGNC Symbol;Acc:HGNC:9865]	0.166907941	1.130779426	0.003645159	-1.30133253	3
43	ENSG00000078401	EDN1	endothelin 1 [Source:HGNC Symbol;Acc:HGNC:3176]	0.689220767	0.905497923	-0.331803549	-1.26291514	3
44	ENSG00000079482	OPHN1	oligophrenin 1 [Source:HGNC Symbol;Acc:HGNC:8148]	0.554672847	0.756011556	0.138961819	-1.44964622	3
45	ENSG00000079819	EPB41L2	erythrocyte membrane protein band 4.1-like 2 [Source:HGNC Symbol;Acc:HG	0.189415136	1.287373974	-0.409504666	-1.06728444	3
46	ENSG00000081923	ATP8B1	ATPase, aminophospholipid transporter, class I, type 8B, member 1 [Source:H	0.199809464	1.008660334	0.174379539	-1.38284934	3
47	ENSG00000082175	PGR	progesterone receptor [Source:HGNC Symbol;Acc:HGNC:8910]	0.126998288	1.357350904	-0.601065251	-0.88328394	3
48	ENSG00000085276	MECOM	MDS1 and EVI1 complex locus [Source:HGNC Symbol;Acc:HGNC:3498]	0.480260219	0.577737266	0.439476976	-1.49747446	3
49	ENSG00000087589	CASS4	Cas scaffolding protein family member 4 [Source:HGNC Symbol;Acc:HGNC:15	0.080965798	1.188120053	-0.011435459	-1.25765039	3
50	ENSG00000088320	REM1	RAS (RAD and GEM)-like GTP-binding 1 [Source:HGNC Symbol;Acc:HGNC:1592	0.601153384	0.991972786	-0.357485013	-1.23564116	3
51	ENSG00000089127	OAS1	2'-5'-oligoadenylate synthetase 1, 40/46kDa [Source:HGNC Symbol;Acc:HGNC	0.403865711	1.224827566	-0.74234308	-0.8863502	3
52	ENSG00000091972	CD200	CD200 molecule [Source:HGNC Symbol;Acc:HGNC:7203]	0.779016367	0.849017888	-0.397451191	-1.23058306	3
53	ENSG00000092929	UNC13D	unc-13 homolog D (C. elegans) [Source:HGNC Symbol;Acc:HGNC:23147]	0.240052608	1.302979215	-0.606976692	-0.93605513	3
54	ENSG00000092969	TGFB2	transforming growth factor, beta 2 [Source:HGNC Symbol;Acc:HGNC:11768]	0.767649486	0.947620451	-0.71331888	-1.00195106	3
55	ENSG00000093072	CECR1	cat eye syndrome chromosome region, candidate 1 [Source:HGNC Symbol;Ac	0.498198785	0.918500515	-0.035803622	-1.38089568	3
56	ENSG00000094963	FMO2	flavin containing monooxygenase 2 (non-functional) [Source:HGNC Symbol;A	0.011476298	1.397053013	-0.536732925	-0.87179639	3
57	ENSG00000099260	PALMD	palmdelphin [Source:HGNC Symbol;Acc:HGNC:15846]	0.602130659	1.090383798	-0.756259315	-0.93625514	3
58	ENSG00000099849	RASSF7	Ras association (RalGDS/AF-6) domain family (N-terminal) member 7 [Source:	0.760250576	0.912069947	-0.526467263	-1.14585326	3
59	ENSG00000099889	ARVCF	armadillo repeat gene deleted in velocardiofacial syndrome [Source:HGNC Sy	0.657544359	0.723241564	0.048278879	-1.4290648	3
60	ENSG00000099954	CECR2	cat eye syndrome chromosome region, candidate 2 [Source:HGNC Symbol;Ac	0.483309649	1.056838587	-0.288682547	-1.25146569	3
61	ENSG00000100024	UPB1	ureidopropionase, beta [Source:HGNC Symbol;Acc:HGNC:16297]	0.832612673	0.885997334	-0.709380549	-1.00922946	3
62	ENSG00000100311	PDGFB	platelet-derived growth factor beta polypeptide [Source:HGNC Symbol;Acc:H	0.786338706	0.918830778	-0.64810986	-1.05705962	3
63	ENSG00000100368	CSF2RB	colony stimulating factor 2 receptor, beta, low-affinity (granulocyte-macroph	0.323034433	1.271586492	-0.736859639	-0.85776128	3
64	ENSG00000101194	SLC17A9	solute carrier family 17 (vesicular nucleotide transporter), member 9 [Source:	0.461073863	1.120819467	-0.41679338	-1.16509995	3
65	ENSG00000101210	EEF1A2	eukaryotic translation elongation factor 1 alpha 2 [Source:HGNC Symbol;Acc:I	0.503853812	1.155289957	-0.697175833	-0.96196794	3
66	ENSG00000101850	GPR143	G protein-coupled receptor 143 [Source:HGNC Symbol;Acc:HGNC:20145]	0.433754108	0.732218796	0.310312658	-1.47628556	3
67	ENSG00000103642	LACTB	lactamase, beta [Source:HGNC Symbol;Acc:HGNC:16468]	0.381032348	1.064405853	-0.140821887	-1.30461631	3
68	ENSG00000104518	GSDMD	gasdermin D [Source:HGNC Symbol;Acc:HGNC:25697]	0.560526427	0.731377852	0.165339494	-1.45724377	3
69	ENSG00000105855	ITGB8	integrin, beta 8 [Source:HGNC Symbol;Acc:HGNC:6163]	0.394206101	0.965276664	0.023375186	-1.38285795	3
70	ENSG00000105928	DFNA5	deafness, autosomal dominant 5 [Source:HGNC Symbol;Acc:HGNC:2810]	0.531090045	0.644390592	0.309986467	-1.4854671	3
71	ENSG00000105929	ATP6V0A4	ATPase, H+ transporting, lysosomal V0 subunit a4 [Source:HGNC Symbol;Acc:	0.682919647	1.030128922	-0.8055956	-0.90745297	3
72	ENSG00000106069	CHN2	chimerin 2 [Source:HGNC Symbol;Acc:HGNC:1944]	0.337353877	1.233207664	-0.528318284	-1.04224326	3
73	ENSG00000106785	TRIM14	tripartite motif containing 14 [Source:HGNC Symbol;Acc:HGNC:16283]	0.630726598	0.796366792	-0.024458123	-1.40263527	3
74	ENSG00000106868	SUSD1	sushi domain containing 1 [Source:HGNC Symbol;Acc:HGNC:25413]	0.505999391	0.932546862	-0.071348244	-1.36719801	3
75	ENSG00000107438	PDLIM1	PDZ and LIM domain 1 [Source:HGNC Symbol;Acc:HGNC:2067]	0.326154936	0.953845729	0.123085649	-1.40308631	3
76	ENSG00000108387	SEPT4	septin 4 [Source:HGNC Symbol;Acc:HGNC:9165]	0.753767821	0.918935997	-0.52941407	-1.14328975	3
77	ENSG00000108639	SYNGR2	synaptogyrin 2 [Source:HGNC Symbol;Acc:HGNC:11499]	0.696407178	0.968259305	-0.522548064	-1.14211842	3
78	ENSG00000109062	SLC9A3R1	solute carrier family 9, subfamily A (NHE3, cation proton antiporter 3), memb	0.48616835	0.957632466	-0.087747431	-1.35605339	3
79	ENSG00000109193	SULT1E1	sulfotransferase family 1E, estrogen-preferring, member 1 [Source:HGNC Sym	0.4242857	1.11322834	-0.322254166	-1.21525987	3
80	ENSG00000109743	BST1	bone marrow stromal cell antigen 1 [Source:HGNC Symbol;Acc:HGNC:1118]	0.658472317	0.814921686	-0.097607059	-1.37578694	3
81	ENSG00000110090	CPT1A	carnitine palmitoyltransferase 1A (liver) [Source:HGNC Symbol;Acc:HGNC:232	0.473979078	0.622728219	0.396678084	-1.49338538	3
82	ENSG00000111057	KRT18	keratin 18 [Source:HGNC Symbol;Acc:HGNC:6430]	0.523593661	0.911386432	-0.059599282	-1.37538081	3
83	ENSG00000111261	MANSC1	MANSC domain containing 1 [Source:HGNC Symbol;Acc:HGNC:25505]	0.49329384	0.794154137	0.16163905	-1.44908703	3
84	ENSG00000111331	OAS3	2'-5'-oligoadenylate synthetase 3, 100kDa [Source:HGNC Symbol;Acc:HGNC:8	0.581479051	1.078278032	-0.583097455	-1.07665963	3
85	ENSG00000111335	OAS2	2'-5'-oligoadenylate synthetase 2, 69/71kDa [Source:HGNC Symbol;Acc:HGNC	-0.28486346	1.479106648	-0.502285058	-0.69195813	3
86	ENSG00000111816	FRK	fyn-related Src family tyrosine kinase [Source:HGNC Symbol;Acc:HGNC:3955]	0.601237472	0.877062916	-0.116017735	-1.36228265	3
87	ENSG00000111859	NEDD9	neural precursor cell expressed, developmentally down-regulated 9 [Source:H	0.599920751	0.714426893	0.138418549	-1.45276619	3
88	ENSG00000111907	TPD52L1	tumor protein D52-like 1 [Source:HGNC Symbol;Acc:HGNC:12006]	0.702921293	0.996530341	-0.664306015	-1.03514562	3
89	ENSG00000111913	FAM65B	family with sequence similarity 65, member B [Source:HGNC Symbol;Acc:HGN	0.602667309	1.076899016	-0.657287816	-1.02227851	3
90	ENSG00000112294	ALDH5A1	aldehyde dehydrogenase 5 family, member A1 [Source:HGNC Symbol;Acc:HG	0.452101672	1.050135481	-0.219864721	-1.28237243	3
91	ENSG00000112541	PDE10A	phosphodiesterase 10A [Source:HGNC Symbol;Acc:HGNC:8772]	0.359314255	0.945262921	0.098181421	-1.4027586	3
92	ENSG00000113319	RASGRF2	Ras protein-specific guanine nucleotide-releasing factor 2 [Source:HGNC Sym	0.584687838	0.887820081	-0.109446235	-1.36306168	3
93	ENSG00000113924	HGD	homogentisate 1,2-dioxygenase [Source:HGNC Symbol;Acc:HGNC:4892]	0.74796067	0.942887658	-0.598408311	-1.09244002	3
94	ENSG00000114166	KAT2B	K(lysine) acetyltransferase 2B [Source:HGNC Symbol;Acc:HGNC:8638]	0.46829973	0.710294071	0.299982589	-1.47857639	3
95	ENSG00000115380	EFEMP1	EGF containing fibulin-like extracellular matrix protein 1 [Source:HGNC Symbo	0.82628565	0.872482288	-0.611560057	-1.08720788	3
96	ENSG00000115415	STAT1	signal transducer and activator of transcription 1, 91kDa [Source:HGNC Symb	0.536803721	1.105077545	-0.553771833	-1.08810943	3
97	ENSG00000116035	VAX2	ventral anterior homeobox 2 [Source:HGNC Symbol;Acc:HGNC:12661]	0.049060639	1.373875218	-0.490462597	-0.93247326	3
98	ENSG00000116039	ATP6V1B1	ATPase, H+ transporting, lysosomal 56/58kDa, V1 subunit B1 [Source:HGNC S	0.195120716	1.194356729	-0.160830434	-1.22864701	3
99	ENSG00000116990	MYCL	v-myc avian myelocytomatosis viral oncogene lung carcinoma derived homol	0.601975077	0.828579697	-0.034161314	-1.39639346	3
100	ENSG00000117791	MARC2	mitochondrial amidoxime reducing component 2 [Source:HGNC Symbol;Acc:H	0.573021717	0.831248283	0.003090243	-1.40736024	3
101	ENSG00000118271	TTR	transthyretin [Source:HGNC Symbol;Acc:HGNC:12405]	0.085228077	1.383280705	-0.711763907	-0.75674488	3
102	ENSG00000118407	FILIP1	filamin A interacting protein 1 [Source:HGNC Symbol;Acc:HGNC:21015]	0.488525029	1.167921483	-0.715539664	-0.94090685	3
103	ENSG00000118526	TCF21	transcription factor 21 [Source:HGNC Symbol;Acc:HGNC:11632]	0.226961932	1.271643087	-0.426422009	-1.07218301	3
104	ENSG00000119227	PIGZ	phosphatidylinositol glycan anchor biosynthesis, class Z [Source:HGNC Symbo	0.608257303	0.936195807	-0.242654671	-1.30179844	3
105	ENSG00000119280	C1orf198	chromosome 1 open reading frame 198 [Source:HGNC Symbol;Acc:HGNC:259	0.739693215	0.900591708	-0.435096225	-1.2051887	3
106	ENSG00000119782	FKBP1B	FK506 binding protein 1B, 12.6 kDa [Source:HGNC Symbol;Acc:HGNC:3712]	0.47426392	0.604060495	0.417078578	-1.49540299	3
107	ENSG00000120875	DUSP4	dual specificity phosphatase 4 [Source:HGNC Symbol;Acc:HGNC:3070]	0.472951169	0.975079223	-0.100660685	-1.34736971	3
108	ENSG00000120937	NPPB	natriuretic peptide B [Source:HGNC Symbol;Acc:HGNC:7940]	0.794322722	0.934852977	-0.858382032	-0.87079367	3

109	ENSG00000121310	ECHDC2	enoyl CoA hydratase domain containing 2 [Source:HGNC Symbol;Acc:HGNC:2	0.798743643	0.847989327	-0.4445143	-1.20221867	3
110	ENSG00000121410	A1BG	alpha-1-B glycoprotein [Source:HGNC Symbol;Acc:HGNC:5]	0.740249125	0.90289355	-0.442852745	-1.20028993	3
111	ENSG00000123095	BHLHE41	basic helix-loop-helix family, member e41 [Source:HGNC Symbol;Acc:HGNC:1	0.420187191	1.025697622	-0.120407899	-1.32547691	3
112	ENSG00000123240	OPTN	optineurin [Source:HGNC Symbol;Acc:HGNC:17142]	0.658064064	0.929032586	-0.322160875	-1.26493577	3
113	ENSG00000123610	TNFAIP6	tumor necrosis factor, alpha-induced protein 6 [Source:HGNC Symbol;Acc:HG	0.479303059	1.173106946	-0.706081616	-0.94632839	3
114	ENSG00000123933	MXD4	MAX dimerization protein 4 [Source:HGNC Symbol;Acc:HGNC:13906]	0.679142218	0.915992638	-0.335238687	-1.25989617	3
115	ENSG00000124134	KCNS1	potassium voltage-gated channel, delayed-rectifier, subfamily S, member 1 [S	0.427402834	1.149974102	-0.434550514	-1.14282642	3
116	ENSG00000124171	PARD6B	par-6 family cell polarity regulator beta [Source:HGNC Symbol;Acc:HGNC:162	0.670615724	1.002502277	-0.566742568	-1.10637543	3
117	ENSG00000124212	PTGIS	prostaglandin I2 (prostacyclin) synthase [Source:HGNC Symbol;Acc:HGNC:960	0.808934492	0.905065935	-0.687321238	-1.02667919	3
118	ENSG00000124839	RAB17	RAB17, member RAS oncogene family [Source:HGNC Symbol;Acc:HGNC:1652	0.424899299	1.120445883	-0.342547178	-1.202798	3
119	ENSG00000124920	MYRF	myelin regulatory factor [Source:HGNC Symbol;Acc:HGNC:1181]	0.601774412	0.781744051	0.039569055	-1.42308752	3
120	ENSG00000125089	SH3TC1	SH3 domain and tetratricopeptide repeats 1 [Source:HGNC Symbol;Acc:HGNC	0.422031848	1.075369663	-0.226990316	-1.27041119	3
121	ENSG00000125266	EFNB2	ephrin-B2 [Source:HGNC Symbol;Acc:HGNC:3227]	0.749505313	0.883515339	-0.413854018	-1.21916663	3
122	ENSG00000125378	BMP4	bone morphogenetic protein 4 [Source:HGNC Symbol;Acc:HGNC:1071]	0.423740195	1.198924298	-0.628979913	-0.99368458	3
123	ENSG00000125414	MYH2	myosin, heavy chain 2, skeletal muscle, adult [Source:HGNC Symbol;Acc:HGN	0.371987286	1.184959551	-0.428148859	-1.12879798	3
124	ENSG00000126561	STAT5A	signal transducer and activator of transcription 5A [Source:HGNC Symbol;Acc	0.583536837	0.966291707	-0.262673683	-1.28715486	3
125	ENSG00000128335	APOL2	apolipoprotein L, 2 [Source:HGNC Symbol;Acc:HGNC:619]	0.533107144	1.10352123	-0.536568336	-1.10006004	3
126	ENSG00000128510	CPA4	carboxypeptidase A4 [Source:HGNC Symbol;Acc:HGNC:15740]	0.353038273	1.23162898	-0.565541999	-1.01912525	3
127	ENSG00000129354	AP1M2	adaptor-related protein complex 1, mu 2 subunit [Source:HGNC Symbol;Acc:H	0.772067692	0.89681723	-0.512695751	-1.15618917	3
128	ENSG00000130433	CACNG6	calcium channel, voltage-dependent, gamma subunit 6 [Source:HGNC Symbo	0.545503078	0.727859606	0.188521822	-1.46188451	3
129	ENSG00000130517	PGPEP1	pyroglutamyl-peptidase I [Source:HGNC Symbol;Acc:HGNC:13568]	0.532341534	0.976032399	-0.19455437	-1.31381956	3
130	ENSG00000130762	ARHGEF16	Rho guanine nucleotide exchange factor (GEF) 16 [Source:HGNC Symbol;Acc:H	0.811154501	0.90717039	-0.712960454	-1.00536444	3
131	ENSG00000130940	CASZ1	castor zinc finger 1 [Source:HGNC Symbol;Acc:HGNC:26002]	0.700944824	1.001205317	-0.681359549	-1.02079059	3
132	ENSG00000131914	LIN28A	lin-28 homolog A (C. elegans) [Source:HGNC Symbol;Acc:HGNC:15986]	0.361659534	1.251865963	-0.79448012	-0.81904538	3
133	ENSG00000131941	RHPN2	rhophilin, Rho GTPase binding protein 2 [Source:HGNC Symbol;Acc:HGNC:19	0.593643627	0.775329837	0.060297369	-1.42927083	3
134	ENSG00000132274	TRIM22	tripartite motif containing 22 [Source:HGNC Symbol;Acc:HGNC:16379]	0.556045225	1.014069581	-0.321459334	-1.24865547	3
135	ENSG00000132530	XAF1	XIAP associated factor 1 [Source:HGNC Symbol;Acc:HGNC:30932]	0.399239713	1.200047977	-0.553034896	-1.04625279	3
136	ENSG00000132561	MATN2	matrilin 2 [Source:HGNC Symbol;Acc:HGNC:6908]	0.533188708	1.130646543	-0.668861918	-0.99497333	3
137	ENSG00000132563	REEP2	receptor accessory protein 2 [Source:HGNC Symbol;Acc:HGNC:17975]	0.670920265	0.720969364	0.032543622	-1.42443325	3
138	ENSG00000133020	MYH8	myosin, heavy chain 8, skeletal muscle, perinatal [Source:HGNC Symbol;Acc:H	0.410988231	1.098525131	-0.262305713	-1.24720765	3
139	ENSG00000133056	PIK3C2B	phosphatidylinositol-4-phosphate 3-kinase, catalytic subunit type 2 beta [Sou	0.518116567	1.091819524	-0.45601372	-1.15392237	3
140	ENSG00000133657	ATP13A3	ATPase type 13A3 [Source:HGNC Symbol;Acc:HGNC:24113]	0.584875838	0.844249584	-0.034870444	-1.39425498	3
141	ENSG00000134243	SORT1	sortilin 1 [Source:HGNC Symbol;Acc:HGNC:11186]	0.032524653	1.309144004	-0.232059482	-1.10960917	3
142	ENSG00000134323	MYCN	v-myc avian myelocytomatosis viral oncogene neuroblastoma derived homolo	0.172430026	1.346166287	-0.709114332	-0.80948198	3
143	ENSG00000134363	FST	follistatin [Source:HGNC Symbol;Acc:HGNC:3971]	0.800669082	0.892001191	-0.590702029	-1.10196824	3
144	ENSG00000134508	CABLES1	Cdk5 and Abl enzyme substrate 1 [Source:HGNC Symbol;Acc:HGNC:25097]	0.361409321	1.17669111	-0.38052424	-1.15757619	3
145	ENSG00000134574	DDB2	damage-specific DNA binding protein 2, 48kDa [Source:HGNC Symbol;Acc:HG	0.524162936	0.853349822	0.035217584	-1.41273034	3
146	ENSG00000134755	DSC2	desmocollin 2 [Source:HGNC Symbol;Acc:HGNC:3036]	0.771476777	0.942959199	-0.705550305	-1.00888567	3
147	ENSG00000134762	DSC3	desmocollin 3 [Source:HGNC Symbol;Acc:HGNC:3037]	0.638898574	1.045735922	-0.64325909	-1.04137541	3
148	ENSG00000134871	COL4A2	collagen, type IV, alpha 2 [Source:HGNC Symbol;Acc:HGNC:2203]	0.669492649	0.986100211	-0.504615283	-1.15097758	3
149	ENSG00000135299	ANKRD6	ankyrin repeat domain 6 [Source:HGNC Symbol;Acc:HGNC:17280]	0.658140869	1.026919371	-0.627386916	-1.05767332	3
150	ENSG00000135363	LMO2	LIM domain only 2 (rhombotin-like 1) [Source:HGNC Symbol;Acc:HGNC:6642]	0.645905603	0.704408272	0.091309349	-1.44162323	3
151	ENSG00000135480	KRT7	keratin 7 [Source:HGNC Symbol;Acc:HGNC:6445]	0.606318651	1.04844115	-0.54099393	-1.11376587	3
152	ENSG00000135636	DYSF	dysferlin [Source:HGNC Symbol;Acc:HGNC:3097]	0.677711994	1.01255149	-0.636624416	-1.05363907	3
153	ENSG00000135643	KCNMB4	potassium large conductance calcium-activated channel, subfamily M, beta m	0.342480857	0.905480276	0.177302415	-1.42526355	3
154	ENSG00000136383	ALPK3	alpha-kinase 3 [Source:HGNC Symbol;Acc:HGNC:17574]	-0.00849625	1.281061622	-0.112308631	-1.16025674	3
155	ENSG00000136869	TLR4	toll-like receptor 4 [Source:HGNC Symbol;Acc:HGNC:11850]	0.051998358	1.210919904	-0.025865254	-1.23705301	3
156	ENSG00000137463	MGARP	mitochondria-localized glutamic acid-rich protein [Source:HGNC Symbol;Acc:H	0.289281469	1.137862466	-0.164383777	-1.26276016	3
157	ENSG00000137486	ARRB1	arrestin, beta 1 [Source:HGNC Symbol;Acc:HGNC:711]	0.131498557	1.149219803	0.008440565	-1.28915892	3
158	ENSG00000137809	ITGA11	integrin, alpha 11 [Source:HGNC Symbol;Acc:HGNC:6136]	0.78431718	0.828228606	-0.359768704	-1.25277708	3
159	ENSG00000138190	EXOC6	exocyst complex component 6 [Source:HGNC Symbol;Acc:HGNC:23196]	0.3505984	0.813284065	0.295081741	-1.45896421	3
160	ENSG00000138378	STAT4	signal transducer and activator of transcription 4 [Source:HGNC Symbol;Acc:H	0.678059306	0.956809501	-0.437728499	-1.19714031	3
161	ENSG00000138650	PCDH10	protocadherin 10 [Source:HGNC Symbol;Acc:HGNC:13404]	0.633660325	0.88849999	-0.190748522	-1.33141179	3
162	ENSG00000138771	SHROOM3	shroom family member 3 [Source:HGNC Symbol;Acc:HGNC:30422]	0.376029685	1.24307262	-0.773315797	-0.84578651	3
163	ENSG00000139116	KIF21A	kinesin family member 21A [Source:HGNC Symbol;Acc:HGNC:19349]	0.525024962	0.950725032	-0.13308095	-1.34266904	3
164	ENSG00000139211	AMIGO2	adhesion molecule with Ig-like domain 2 [Source:HGNC Symbol;Acc:HGNC:24	0.353310383	1.132056288	-0.247474662	-1.23789201	3
165	ENSG00000139304	PTPRQ	protein tyrosine phosphatase, receptor type, Q [Source:HGNC Symbol;Acc:HG	0.094675887	1.37861373	-0.685804314	-0.7874853	3
166	ENSG00000140403	DNAJA4	DnaJ (Hsp40) homolog, subfamily A, member 4 [Source:HGNC Symbol;Acc:HG	0.591986179	0.856113311	-0.065215055	-1.38288443	3
167	ENSG00000140470	ADAMTS17	ADAM metallopeptidase with thrombospondin type 1 motif, 17 [Source:HGN	0.409917071	1.032709532	-0.119277897	-1.32334871	3
168	ENSG00000140479	PCSK6	proprotein convertase subtilisin/kexin type 6 [Source:HGNC Symbol;Acc:HGN	0.651302578	1.055914516	-0.8114518	-0.89576529	3
169	ENSG00000140937	CDH11	cadherin 11, type 2, OB-cadherin (osteoblast) [Source:HGNC Symbol;Acc:HGN	0.457524778	1.085718809	-0.312772491	-1.23047109	3
170	ENSG00000140945	CDH13	cadherin 13 [Source:HGNC Symbol;Acc:HGNC:1753]	0.526580967	0.913458839	-0.067544573	-1.37249523	3
171	ENSG00000141068	KSR1	kinase suppressor of ras 1 [Source:HGNC Symbol;Acc:HGNC:6465]	0.694415657	0.929290415	-0.401977741	-1.22172833	3
172	ENSG00000141448	GATA6	GATA binding protein 6 [Source:HGNC Symbol;Acc:HGNC:4174]	0.717002597	0.83263152	-0.230781637	-1.31885248	3
173	ENSG00000142623	PADI1	peptidyl arginine deiminase, type I [Source:HGNC Symbol;Acc:HGNC:18367]	0.14501363	1.3588395	-0.722438069	-0.78141506	3
174	ENSG00000143248	RGS5	regulator of G-protein signaling 5 [Source:HGNC Symbol;Acc:HGNC:10001]	0.060817645	1.325792023	-0.320992101	-1.06561757	3
175	ENSG00000144152	FBLN7	fibulin 7 [Source:HGNC Symbol;Acc:HGNC:26740]	0.661531393	0.738166793	0.020535699	-1.42023388	3
176	ENSG00000144366	GULP1	GULP, engulfment adaptor PTB domain containing 1 [Source:HGNC Symbol;A	0.498738496	0.996215759	-0.181186583	-1.31376767	3
177	ENSG00000144810	COL8A1	collagen, type VIII, alpha 1 [Source:HGNC Symbol;Acc:HGNC:2215]	0.637641856	1.048251992	-0.651294125	-1.03459972	3
178	ENSG00000144868	TMEM108	transmembrane protein 108 [Source:HGNC Symbol;Acc:HGNC:28451]	0.653654384	0.813877785	-0.088199061	-1.37933311	3
179	ENSG00000145014	TMEM44	transmembrane protein 44 [Source:HGNC Symbol;Acc:HGNC:25120]	0.512688793	0.904402147	-0.032108631	-1.38498231	3
180	ENSG00000145246	ATP10D	ATPase, class V, type 10D [Source:HGNC Symbol;Acc:HGNC:13549]	0.415039179	0.844944458	0.182421858	-1.44240549	3
181	ENSG00000145284	SCD5	stearoyl-CoA desaturase 5 [Source:HGNC Symbol;Acc:HGNC:21088]	0.229603405	0.959131623	0.218283495	-1.40701852	3
182	ENSG00000145632	PLK2	polo-like kinase 2 [Source:HGNC Symbol;Acc:HGNC:19699]	0.663135143	0.915579369	-0.301609143	-1.27710537	3
183	ENSG00000145681	HAPLN1	hyaluronan and proteoglycan link protein 1 [Source:HGNC Symbol;Acc:HGNC:	0.623245982	1.013768331	-0.469272075	-1.16774224	3
184	ENSG00000145808	ADAMTS19	ADAM metallopeptidase with thrombospondin type 1 motif, 19 [Source:HGN	0.462097186	1.180335162	-0.672080185	-0.97035216	3
185	ENSG00000146038	DCDC2	doublecortin domain containing 2 [Source:HGNC Symbol;Acc:HGNC:18141]	0.348414934	1.215138089	-0.48202876	-1.08152426	3
186	ENSG00000146072	TNFRSF21	tumor necrosis factor receptor superfamily, member 21 [Source:HGNC Symbo	0.363882386	1.239358523	-0.649322464	-0.95391844	3
187	ENSG00000146950	SHROOM2	shroom family member 2 [Source:HGNC Symbol;Acc:HGNC:630]	0.610504325	0.632219827	0.231691527	-1.47441568	3
188	ENSG00000148288	GBGT1	globoside alpha-1,3-N-acetylgalactosaminyltransferase 1 [Source:HGNC Symb	0.220541097	1.161000774	-0.121073711	-1.26046816	3
189	ENSG00000148677	ANKRD1	ankyrin repeat domain 1 (cardiac muscle) [Source:HGNC Symbol;Acc:HGNC:1	0.551367096	1.101975662	-0.587012424	-1.06633033	3
190	ENSG00000149212	SESN3	sestrin 3 [Source:HGNC Symbol;Acc:HGNC:23060]	0.396596191	0.969986469	0.01243262	-1.37901528	3
191	ENSG00000149596	JPH2	junctophilin 2 [Source:HGNC Symbol;Acc:HGNC:14202]	0.844966847	0.882104019	-0.772605136	-0.95446573	3
192	ENSG00000149781	FERMT3	fermitin family member 3 [Source:HGNC Symbol;Acc:HGNC:23151]	0.68453972	0.915837902	-0.346293425	-1.2540842	3

193	ENSG00000150054	MPP7	membrane protein, palmitoylated 7 (MAGUK p55 subfamily member 7) [Sour	-0.01525946	1.393145781	-0.454820732	-0.92306559	3
194	ENSG00000150907	FOXO1	forkhead box O1 [Source:HGNC Symbol;Acc:HGNC:3819]	0.519559154	0.824259884	0.085628971	-1.42944801	3
195	ENSG00000151322	NPAS3	neuronal PAS domain protein 3 [Source:HGNC Symbol;Acc:HGNC:19311]	0.629293967	0.730541092	0.076958656	-1.43679372	3
196	ENSG00000151640	DPYSL4	dihydropyrimidinase-like 4 [Source:HGNC Symbol;Acc:HGNC:3016]	0.776962642	0.826450925	-0.339191708	-1.26422186	3
197	ENSG00000151729	SLC25A4	solute carrier family 25 (mitochondrial carrier; adenine nucleotide translocato	0.683304958	0.896730287	-0.299755734	-1.28027951	3
198	ENSG00000152056	AP1S3	adaptor-related protein complex 1, sigma 3 subunit [Source:HGNC Symbol;Ac	0.672112242	0.964554143	-0.445129485	-1.1915369	3
199	ENSG00000152229	PSTPIP2	proline-serine-threonine phosphatase interacting protein 2 [Source:HGNC Syr	0.454099587	1.060052349	-0.245336512	-1.26881542	3
200	ENSG00000152377	SPOCK1	sparc/osteonectin, cwcv and kazal-like domains proteoglycan (testican) 1 [Sou	0.478961103	1.03172188	-0.223803776	-1.28687921	3
201	ENSG00000152779	SLC16A12	solute carrier family 16, member 12 [Source:HGNC Symbol;Acc:HGNC:23094]	0.062173625	1.38776424	-0.627283337	-0.82265453	3
202	ENSG00000152926	ZNF117	zinc finger protein 117 [Source:HGNC Symbol;Acc:HGNC:12897]	0.589375867	1.102561769	-0.792737467	-0.89920017	3
203	ENSG00000152939	MARVELD2	MARVEL domain containing 2 [Source:HGNC Symbol;Acc:HGNC:26401]	0.577106451	1.090131201	-0.622999009	-1.04423864	3
204	ENSG00000153208	MERTK	MER proto-oncogene, tyrosine kinase [Source:HGNC Symbol;Acc:HGNC:7027]	0.539389987	0.849359678	0.02093411	-1.40968377	3
205	ENSG00000153246	PLA2R1	phospholipase A2 receptor 1, 180kDa [Source:HGNC Symbol;Acc:HGNC:9042]	0.61408257	1.048767236	-0.566420052	-1.09642975	3
206	ENSG00000153823	PID1	phosphotyrosine interaction domain containing 1 [Source:HGNC Symbol;Acc:I	0.845333537	0.869470373	-0.685419027	-1.02938488	3
207	ENSG00000154310	TNIK	TRAF2 and NCK interacting kinase [Source:HGNC Symbol;Acc:HGNC:30765]	0.516851248	0.761373817	0.178275087	-1.45650015	3
208	ENSG00000154511	FAM69A	family with sequence similarity 69, member A [Source:HGNC Symbol;Acc:HGN	0.393586296	1.194921504	-0.515350771	-1.07315703	3
209	ENSG00000154589	LY96	lymphocyte antigen 96 [Source:HGNC Symbol;Acc:HGNC:17156]	0.671616124	0.753141284	-0.017129414	-1.40762799	3
210	ENSG00000154639	CXADR	coxsackie virus and adenovirus receptor [Source:HGNC Symbol;Acc:HGNC:25!	0.3801469	1.108118879	-0.234185162	-1.25408062	3
211	ENSG00000154760	SLFN13	schlafen family member 13 [Source:HGNC Symbol;Acc:HGNC:26481]	0.398325833	1.216990042	-0.640851043	-0.97446483	3
212	ENSG00000154764	WNT7A	wingless-type MMTV integration site family, member 7A [Source:HGNC Symb	0.208378683	1.331011857	-0.761816655	-0.77757388	3
213	ENSG00000154783	FGD5	FYVE, RhoGEF and PH domain containing 5 [Source:HGNC Symbol;Acc:HGNC::	0.637958534	1.056930491	-0.706763236	-0.98812579	3
214	ENSG00000154864	PIEZO2	piezo-type mechanosensitive ion channel component 2 [Source:HGNC Symbo	0.758770063	0.927826435	-0.577683655	-1.10891284	3
215	ENSG00000155754	ALS2CR11	amyotrophic lateral sclerosis 2 (juvenile) chromosome region, candidate 11 [S	0.651580429	0.991829896	-0.474345369	-1.16906496	3
216	ENSG00000155792	DEPTOR	DEP domain containing MTOR-interacting protein [Source:HGNC Symbol;Acc:	0.625645157	1.074292329	-0.780793598	-0.91914389	3
217	ENSG00000156049	GNA14	guanine nucleotide binding protein (G protein), alpha 14 [Source:HGNC Symb	0.716044443	0.822422407	-0.209116646	-1.3293502	3
218	ENSG00000156140	ADAMTS3	ADAM metallopeptidase with thrombospondin type 1 motif, 3 [Source:HGNC	0.719290119	0.964195486	-0.581083161	-1.10240244	3
219	ENSG00000156265	MAP3K7CL	MAP3K7 C-terminal like [Source:HGNC Symbol;Acc:HGNC:16457]	0.453175172	1.191965562	-0.721065819	-0.92407491	3
220	ENSG00000156463	SH3RF2	SH3 domain containing ring finger 2 [Source:HGNC Symbol;Acc:HGNC:26299]	0.346617645	1.229533552	-0.537100345	-1.03905085	3
221	ENSG00000156466	GDF6	growth differentiation factor 6 [Source:HGNC Symbol;Acc:HGNC:4221]	0.553665891	1.094084569	-0.560348905	-1.08740155	3
222	ENSG00000156675	RAB11FIP1	RAB11 family interacting protein 1 (class I) [Source:HGNC Symbol;Acc:HGNC:3	0.701162107	0.98208776	-0.589424344	-1.09382552	3
223	ENSG00000157111	TMEM171	transmembrane protein 171 [Source:HGNC Symbol;Acc:HGNC:27031]	0.699915923	0.758379363	-0.0689712	-1.38932409	3
224	ENSG00000157404	KIT	v-kit Hardy-Zuckerman 4 feline sarcoma viral oncogene homolog [Source:HGN	0.537609763	1.076551515	-0.454054957	-1.16010632	3
225	ENSG00000159388	BTG2	BTG family, member 2 [Source:HGNC Symbol;Acc:HGNC:1131]	0.691102918	0.943950225	-0.43370673	-1.20134641	3
226	ENSG00000159733	ZFYVE28	zinc finger, FYVE domain containing 28 [Source:HGNC Symbol;Acc:HGNC:2933	0.489741344	0.93763313	-0.057067643	-1.37030683	3
227	ENSG00000161798	AQP5	aquaporin 5 [Source:HGNC Symbol;Acc:HGNC:638]	0.234350484	1.296243659	-0.549110796	-0.98148335	3
228	ENSG00000162383	SLC1A7	solute carrier family 1 (glutamate transporter), member 7 [Source:HGNC Sym	0.586922822	1.099021999	-0.727934865	-0.95800996	3
229	ENSG00000162444	RBP7	retinol binding protein 7, cellular [Source:HGNC Symbol;Acc:HGNC:30316]	0.836281866	0.887928482	-0.748604095	-0.97560625	3
230	ENSG00000162458	FBLIM1	filamin binding LIM protein 1 [Source:HGNC Symbol;Acc:HGNC:24686]	0.611872279	0.768113681	0.046020968	-1.42600693	3
231	ENSG00000162591	MEGF6	multiple EGF-like-domains 6 [Source:HGNC Symbol;Acc:HGNC:3232]	0.81845811	0.908095185	-0.776684733	-0.94986856	3
232	ENSG00000162645	GBP2	guanylate binding protein 2, interferon-inducible [Source:HGNC Symbol;Acc:H	0.600948521	0.997012022	-0.369976221	-1.22798432	3
233	ENSG00000162654	GBP4	guanylate binding protein 4 [Source:HGNC Symbol;Acc:HGNC:20480]	0.151474826	1.307058091	-0.40911385	-1.04941907	3
234	ENSG00000163219	ARHGAP25	Rho GTPase activating protein 25 [Source:HGNC Symbol;Acc:HGNC:28951]	0.344038326	1.234001364	-0.550510273	-1.02752942	3
235	ENSG00000163251	FZD5	frizzled class receptor 5 [Source:HGNC Symbol;Acc:HGNC:4043]	0.666730926	0.950103387	-0.392666735	-1.22416758	3
236	ENSG00000163453	IGFBP7	insulin-like growth factor binding protein 7 [Source:HGNC Symbol;Acc:HGNC:!	0.70959367	0.967358592	-0.560438604	-1.11651366	3
237	ENSG00000163492	CCDC141	coiled-coil domain containing 141 [Source:HGNC Symbol;Acc:HGNC:26821]	0.757643357	0.886653648	-0.442697892	-1.20159911	3
238	ENSG00000163520	FBLN2	fibulin 2 [Source:HGNC Symbol;Acc:HGNC:3601]	0.646332389	0.954229173	-0.358238661	-1.2423229	3
239	ENSG00000163827	LRRC2	leucine rich repeat containing 2 [Source:HGNC Symbol;Acc:HGNC:14676]	0.59159952	0.694356781	0.175873748	-1.46183005	3
240	ENSG00000164056	SPRY1	sprouty homolog 1, antagonist of FGF signaling (Drosophila) [Source:HGNC Sy	0.441917489	1.203322342	-0.781893867	-0.86334596	3
241	ENSG00000164078	MST1R	macrophage stimulating 1 receptor (c-met-related tyrosine kinase) [Source:H	0.716006903	1.002054307	-0.798391833	-0.91966938	3
242	ENSG00000164400	CSF2	colony stimulating factor 2 (granulocyte-macrophage) [Source:HGNC Symbol;	0.853450179	0.859372991	-0.674451084	-1.03837209	3
243	ENSG00000164488	DACT2	dishevelled-binding antagonist of beta-catenin 2 [Source:HGNC Symbol;Acc:H	0.683119951	1.02700916	-0.764708233	-0.94542088	3
244	ENSG00000164512	ANKRD55	ankyrin repeat domain 55 [Source:HGNC Symbol;Acc:HGNC:25681]	0.609824433	0.766816068	0.050779791	-1.42742029	3
245	ENSG00000164619	BMPER	BMP binding endothelial regulator [Source:HGNC Symbol;Acc:HGNC:24154]	0.524188296	0.924205479	-0.083046125	-1.36534765	3
246	ENSG00000164695	CHMP4C	charged multivesicular body protein 4C [Source:HGNC Symbol;Acc:HGNC:305	0.737302717	0.814310807	-0.232883956	-1.31872957	3
247	ENSG00000164761	TNFRSF11B	tumor necrosis factor receptor superfamily, member 11b [Source:HGNC Symt	0.057959256	1.363632361	-0.45911531	-0.96247631	3
248	ENSG00000164930	FZD6	frizzled class receptor 6 [Source:HGNC Symbol;Acc:HGNC:4044]	0.5341824	0.75727523	0.162742577	-1.45420021	3
249	ENSG00000164938	TP53INP1	tumor protein p53 inducible nuclear protein 1 [Source:HGNC Symbol;Acc:HGI	0.500530375	1.04380103	-0.288704738	-1.25562667	3
250	ENSG00000165072	MAMDC2	MAM domain containing 2 [Source:HGNC Symbol;Acc:HGNC:23673]	0.075310069	1.302161296	-0.270411244	-1.10706012	3
251	ENSG00000165626	BEND7	BEN domain containing 7 [Source:HGNC Symbol;Acc:HGNC:23514]	0.686914198	0.893360549	-0.299514465	-1.28076028	3
252	ENSG00000165795	NDRG2	NDRG family member 2 [Source:HGNC Symbol;Acc:HGNC:14460]	0.644761722	0.949659832	-0.343703072	-1.25071848	3
253	ENSG00000165996	PTPLA	protein tyrosine phosphatase-like (proline instead of catalytic arginine), mem	0.599825505	1.017000449	-0.421853559	-1.19497239	3
254	ENSG00000166317	SYNPO2L	synaptopodin 2-like [Source:HGNC Symbol;Acc:HGNC:23532]	0.597213908	1.098198972	-0.841297535	-0.85411535	3
255	ENSG00000166387	PPFIBP2	PTPRF interacting protein, binding protein 2 (liprin beta 2) [Source:HGNC Sym	0.366724133	0.855312521	0.22182833	-1.44386498	3
256	ENSG00000166974	MAPRE2	microtubule-associated protein, RP/EB family, member 2 [Source:HGNC Symt	0.343736431	1.182411766	-0.363584725	-1.16256347	3
257	ENSG00000167528	ZNF641	zinc finger protein 641 [Source:HGNC Symbol;Acc:HGNC:31834]	0.496866106	1.155715388	-0.665149164	-0.98743233	3
258	ENSG00000167549	CORO6	coronin 6 [Source:HGNC Symbol;Acc:HGNC:21356]	0.675417101	0.757442749	-0.029571663	-1.40328819	3
259	ENSG00000167642	SPINT2	serine peptidase inhibitor, Kunitz type, 2 [Source:HGNC Symbol;Acc:HGNC:11	0.835556513	0.891310853	-0.772949358	-0.95391801	3
260	ENSG00000167767	KRT80	keratin 80 [Source:HGNC Symbol;Acc:HGNC:27056]	0.414262995	1.109374395	-0.294411391	-1.229226	3
261	ENSG00000167972	ABCA3	ATP-binding cassette, sub-family A (ABC1), member 3 [Source:HGNC Symbol;/	0.61259316	0.993927325	-0.387096779	-1.21942371	3
262	ENSG00000168016	TRANK1	tetratricopeptide repeat and ankyrin repeat containing 1 [Source:HGNC Symb	0.633359431	1.034611551	-0.571906139	-1.09606484	3
263	ENSG00000168497	SDPR	serum deprivation response [Source:HGNC Symbol;Acc:HGNC:10690]	0.331541933	1.253351694	-0.614618843	-0.97027478	3
264	ENSG00000168505	GBX2	gastrulation brain homeobox 2 [Source:HGNC Symbol;Acc:HGNC:4186]	0.146649133	1.356700877	-0.689120493	-0.81422952	3
265	ENSG00000168899	VAMP5	vesicle-associated membrane protein 5 [Source:HGNC Symbol;Acc:HGNC:126	0.490197386	0.791442603	0.169125866	-1.45076586	3
266	ENSG00000168952	STXBP6	syntaxin binding protein 6 (amisyn) [Source:HGNC Symbol;Acc:HGNC:19666]	0.737027846	0.908063398	-0.44901636	-1.19607488	3
267	ENSG00000168961	LGALS9	lectin, galactoside-binding, soluble, 9 [Source:HGNC Symbol;Acc:HGNC:6570]	0.701614516	0.988903202	-0.620667617	-1.0698501	3
268	ENSG00000169129	AFAP1L2	actin filament associated protein 1-like 2 [Source:HGNC Symbol;Acc:HGNC:25	0.82630732	0.891466313	-0.7053764	-1.01239723	3
269	ENSG00000169242	EFNA1	ephrin-A1 [Source:HGNC Symbol;Acc:HGNC:3221]	0.83317123	0.881632713	-0.686707536	-1.02809641	3
270	ENSG00000169783	LINGO1	leucine rich repeat and Ig domain containing 1 [Source:HGNC Symbol;Acc:HG	0.612427029	1.082360008	-0.754446226	-0.94034081	3
271	ENSG00000169946	ZFPM2	zinc finger protein, FOG family member 2 [Source:HGNC Symbol;Acc:HGNC:1(	0.521825735	1.020852197	-0.273528212	-1.26914972	3
272	ENSG00000170044	ZPLD1	zona pellucida-like domain containing 1 [Source:HGNC Symbol;Acc:HGNC:270	0.282982602	1.294451412	-0.779569508	-0.79786451	3
273	ENSG00000170153	RNF150	ring finger protein 150 [Source:HGNC Symbol;Acc:HGNC:23138]	0.741307158	0.899367519	-0.435827868	-1.20484681	3
274	ENSG00000170421	KRT8	keratin 8 [Source:HGNC Symbol;Acc:HGNC:6446]	0.575248773	0.906985091	-0.129578594	-1.35265527	3
275	ENSG00000170425	ADORA2B	adenosine A2b receptor [Source:HGNC Symbol;Acc:HGNC:264]	0.857346692	0.866923096	-0.746273245	-0.97799654	3
276	ENSG00000170579	DLGAP1	discs, large (Drosophila) homolog-associated protein 1 [Source:HGNC Symbol,	0.403217143	0.946288448	0.043359812	-1.3928654	3

277	ENSG00000170962	PDGFD	platelet derived growth factor D [Source:HGNC Symbol;Acc:HGNC:30620]	0.562600511	1.047894358	-0.425411143	-1.18508373	3
278	ENSG00000171227	TMEM37	transmembrane protein 37 [Source:HGNC Symbol;Acc:HGNC:18216]	0.580394735	0.673876216	0.216201643	-1.47047259	3
279	ENSG00000171316	CHD7	chromodomain helicase DNA binding protein 7 [Source:HGNC Symbol;Acc:HG	0.834205189	0.844382913	-0.537391589	-1.14119651	3
280	ENSG00000171385	KCND3	potassium voltage-gated channel, Shal-related subfamily, member 3 [Source:I	0.184334231	1.341386122	-0.725491293	-0.80022906	3
281	ENSG00000171428	NAT1	N-acetyltransferase 1 (arylamine N-acetyltransferase) [Source:HGNC Symbol;	0.676126384	0.877497122	-0.244844219	-1.30877929	3
282	ENSG00000171444	MCC	mutated in colorectal cancers [Source:HGNC Symbol;Acc:HGNC:6935]	0.736604086	0.755649877	-0.12443705	-1.36781691	3
283	ENSG00000171509	RXFP1	relaxin/insulin-like family peptide receptor 1 [Source:HGNC Symbol;Acc:HGN(	0.458919494	1.192136089	-0.774477933	-0.87657765	3
284	ENSG00000171617	ENC1	ectodermal-neural cortex 1 (with BTB domain) [Source:HGNC Symbol;Acc:HG	0.360525881	1.160636718	-0.332810525	-1.18835207	3
285	ENSG00000172123	SLFN12	schlafen family member 12 [Source:HGNC Symbol;Acc:HGNC:25500]	0.510217295	1.054803071	-0.333943343	-1.23107702	3
286	ENSG00000172379	ARNT2	aryl-hydrocarbon receptor nuclear translocator 2 [Source:HGNC Symbol;Acc:H	0.16327492	1.252836624	-0.260252388	-1.15585916	3
287	ENSG00000172399	MYOZ2	myozenin 2 [Source:HGNC Symbol;Acc:HGNC:1330]	0.340015973	1.252454387	-0.641693563	-0.9507768	3
288	ENSG00000172403	SYNPO2	synaptopodin 2 [Source:HGNC Symbol;Acc:HGNC:17732]	0.65067284	0.985771675	-0.453596603	-1.18284791	3
289	ENSG00000172508	CARNS1	carnosine synthase 1 [Source:HGNC Symbol;Acc:HGNC:29268]	0.347190605	1.154830972	-0.293960625	-1.20806095	3
290	ENSG00000172830	SSH3	slingshot protein phosphatase 3 [Source:HGNC Symbol;Acc:HGNC:30581]	0.495284708	0.754879251	0.212592005	-1.46275596	3
291	ENSG00000172987	HPSE2	heparanase 2 (inactive) [Source:HGNC Symbol;Acc:HGNC:18374]	0.625502661	1.076127161	-0.812574021	-0.8890558	3
292	ENSG00000173535	TNFRSF10C	tumor necrosis factor receptor superfamily, member 10c, decoy without an ir	0.596292109	1.023922297	-0.433629123	-1.18658528	3
293	ENSG00000173801	JUP	junction plakoglobin [Source:HGNC Symbol;Acc:HGNC:6207]	0.521621612	1.006626602	-0.24122714	-1.28702107	3
294	ENSG00000173947	PIFO	primary cilia formation [Source:HGNC Symbol;Acc:HGNC:27009]	0.620265967	1.069891704	-0.699673549	-0.99048412	3
295	ENSG00000174899	C3orf55	PQ loop repeat containing 2-like [Source:HGNC Symbol;Acc:HGNC:25146]	0.63900018	0.936897037	-0.302059376	-1.27383784	3
296	ENSG00000174939	ASPHD1	aspartate beta-hydroxylase domain containing 1 [Source:HGNC Symbol;Acc:H	0.727733119	0.893409567	-0.387385776	-1.23375691	3
297	ENSG00000174951	FUT1	fucosyltransferase 1 (galactoside 2-alpha-L-fucosyltransferase, H blood group	0.666737398	0.968216257	-0.442322327	-1.19263133	3
298	ENSG00000175567	UCP2	uncoupling protein 2 (mitochondrial, proton carrier) [Source:HGNC Symbol;A	0.331523265	1.18565771	-0.350308072	-1.1668729	3
299	ENSG00000175591	P2RY2	purinergic receptor P2Y, G-protein coupled, 2 [Source:HGNC Symbol;Acc:HGN	0.177292769	1.337315834	-0.628591396	-0.88601721	3
300	ENSG00000175906	ARL4D	ADP-ribosylation factor-like 4D [Source:HGNC Symbol;Acc:HGNC:656]	0.647254632	1.036309095	-0.629730583	-1.05383314	3
301	ENSG00000176485	PLA2G16	phospholipase A2, group XVI [Source:HGNC Symbol;Acc:HGNC:17825]	0.508117467	1.014957291	-0.236275549	-1.28679921	3
302	ENSG00000176532	PRR15	proline rich 15 [Source:HGNC Symbol;Acc:HGNC:22310]	0.463864866	1.171623284	-0.62445993	-1.01102822	3
303	ENSG00000176907	C8orf4	chromosome 8 open reading frame 4 [Source:HGNC Symbol;Acc:HGNC:1357]	0.105715054	1.373709869	-0.678899341	-0.80052558	3
304	ENSG00000177106	EPS8L2	EPS8-like 2 [Source:HGNC Symbol;Acc:HGNC:21296]	0.577738563	0.873156385	-0.073041157	-1.37785379	3
305	ENSG00000177425	PAWR	PRKC, apoptosis, WT1, regulator [Source:HGNC Symbol;Acc:HGNC:8614]	0.450973058	1.121496295	-0.397300603	-1.17516875	3
306	ENSG00000177570	SAMD12	sterile alpha motif domain containing 12 [Source:HGNC Symbol;Acc:HGNC:31	0.297581491	1.237144808	-0.448595544	-1.08613076	3
307	ENSG00000178078	STAP2	signal transducing adaptor family member 2 [Source:HGNC Symbol;Acc:HGNC	0.503408493	1.078138358	-0.382391037	-1.19915581	3
308	ENSG00000178776	C5orf46	chromosome 5 open reading frame 46 [Source:HGNC Symbol;Acc:HGNC:3376	0.849310532	0.873647655	-0.736741499	-0.98621669	3
309	ENSG00000178882	FAM101A	family with sequence similarity 101, member A [Source:HGNC Symbol;Acc:HG	0.487859647	1.158299657	-0.642228001	-1.0039313	3
310	ENSG00000179292	TMEM151A	transmembrane protein 151A [Source:HGNC Symbol;Acc:HGNC:28497]	0.639328946	1.051993579	-0.680915905	-1.01040662	3
311	ENSG00000179715	PCED1B	PC-esterase domain containing 1B [Source:HGNC Symbol;Acc:HGNC:28255]	0.484015322	1.027304788	-0.222588856	-1.28873125	3
312	ENSG00000179772	FOXS1	forkhead box S1 [Source:HGNC Symbol;Acc:HGNC:3735]	0.197654529	1.183606741	-0.14014611	-1.24111516	3
313	ENSG00000180914	OXTR	oxytocin receptor [Source:HGNC Symbol;Acc:HGNC:8529]	0.724607799	0.942384407	-0.519311717	-1.14768049	3
314	ENSG00000182022	CHST15	carbohydrate (N-acetylgalactosamine 4-sulfate 6-O) sulfotransferase 15 [Sour	0.591099779	1.091178753	-0.691306945	-0.99097159	3
315	ENSG00000182218	HHIPL1	HHIP-like 1 [Source:HGNC Symbol;Acc:HGNC:19710]	0.594223852	0.818697286	-0.007010894	-1.40591024	3
316	ENSG00000183044	ABAT	4-aminobutyrate aminotransferase [Source:HGNC Symbol;Acc:HGNC:23]	0.741556899	0.980203699	-0.801448422	-0.92031218	3
317	ENSG00000183496	MEX3B	mex-3 RNA binding family member B [Source:HGNC Symbol;Acc:HGNC:25297	0.67681355	0.865390008	-0.221634925	-1.32056863	3
318	ENSG00000183671	GPR1	G protein-coupled receptor 1 [Source:HGNC Symbol;Acc:HGNC:4463]	0.675175418	0.687212338	0.075055801	-1.43744356	3
319	ENSG00000185112	FAM43A	family with sequence similarity 43, member A [Source:HGNC Symbol;Acc:HGN	0.752664484	0.894515562	-0.451610835	-1.19556921	3
320	ENSG00000185477	GPRIN3	GPRIN family member 3 [Source:HGNC Symbol;Acc:HGNC:27733]	0.695395578	0.993474006	-0.61717486	-1.07169472	3
321	ENSG00000185483	ROR1	receptor tyrosine kinase-like orphan receptor 1 [Source:HGNC Symbol;Acc:HG	0.491443759	1.153499072	-0.628816484	-1.01612635	3
322	ENSG00000185513	L3MBTL1	l(3)mbt-like 1 (Drosophila) [Source:HGNC Symbol;Acc:HGNC:15905]	0.326130388	1.24606289	-0.556891063	-1.01530222	3
323	ENSG00000185652	NTF3	neurotrophin 3 [Source:HGNC Symbol;Acc:HGNC:8023]	0.449411569	1.175977084	-0.595711603	-1.02967705	3
324	ENSG00000185761	ADAMTSL5	ADAMTS-like 5 [Source:HGNC Symbol;Acc:HGNC:27912]	0.759945896	0.965958155	-0.858787911	-0.86711614	3
325	ENSG00000185860	C1orf110	chromosome 1 open reading frame 110 [Source:HGNC Symbol;Acc:HGNC:287	0.46286719	1.173254868	-0.62993968	-1.00618238	3
326	ENSG00000187164	KIAA1598	KIAA1598 [Source:HGNC Symbol;Acc:HGNC:29319]	0.69632723	0.801100412	-0.135435031	-1.36199261	3
327	ENSG00000187498	COL4A1	collagen, type IV, alpha 1 [Source:HGNC Symbol;Acc:HGNC:2202]	0.660176123	1.027591054	-0.639011563	-1.04875561	3
328	ENSG00000188157	AGRN	agrin [Source:HGNC Symbol;Acc:HGNC:329]	0.562880888	1.019213411	-0.347844068	-1.23425023	3
329	ENSG00000188452	CERKL	ceramide kinase-like [Source:HGNC Symbol;Acc:HGNC:21699]	0.827513062	0.834373201	-0.48589928	-1.17598698	3
330	ENSG00000188549	C15orf52	chromosome 15 open reading frame 52 [Source:HGNC Symbol;Acc:HGNC:334	0.482258245	0.740614979	0.246236688	-1.46910991	3
331	ENSG00000189221	MAOA	monoamine oxidase A [Source:HGNC Symbol;Acc:HGNC:6833]	0.525417625	1.124996122	-0.605676379	-1.04473737	3
332	ENSG00000196116	TDRD7	tudor domain containing 7 [Source:HGNC Symbol;Acc:HGNC:30831]	0.359692351	0.812055012	0.28725063	-1.45899799	3
333	ENSG00000196730	DAPK1	death-associated protein kinase 1 [Source:HGNC Symbol;Acc:HGNC:2674]	0.689023197	0.994495286	-0.597721419	-1.08579706	3
334	ENSG00000197461	PDGFA	platelet-derived growth factor alpha polypeptide [Source:HGNC Symbol;Acc:H	0.681574425	0.798523722	-0.106617583	-1.37348056	3
335	ENSG00000197822	OCLN	occludin [Source:HGNC Symbol;Acc:HGNC:8104]	0.191102985	1.334512992	-0.665847511	-0.85976847	3
336	ENSG00000197971	MBP	myelin basic protein [Source:HGNC Symbol;Acc:HGNC:6925]	0.428778012	0.861024744	0.143448236	-1.43325099	3
337	ENSG00000197977	ELOVL2	ELOVL fatty acid elongase 2 [Source:HGNC Symbol;Acc:HGNC:14416]	0.244367736	1.306706736	-0.653295862	-0.89777861	3
338	ENSG00000197993	KEL	Kell blood group, metallo-endopeptidase [Source:HGNC Symbol;Acc:HGNC:63	0.053674395	1.340744647	-0.360074075	-1.03434497	3
339	ENSG00000198087	CD2AP	CD2-associated protein [Source:HGNC Symbol;Acc:HGNC:14258]	0.391003987	1.118007229	-0.275643261	-1.23336795	3
340	ENSG00000198208	RPS6KL1	ribosomal protein S6 kinase-like 1 [Source:HGNC Symbol;Acc:HGNC:20222]	0.824900026	0.843327398	-0.504836111	-1.16339131	3
341	ENSG00000198682	PAPSS2	3'-phosphoadenosine 5'-phosphosulfate synthase 2 [Source:HGNC Symbol;Ac	0.374185939	1.02747164	-0.05974965	-1.34190793	3
342	ENSG00000198929	NOS1AP	nitric oxide synthase 1 (neuronal) adaptor protein [Source:HGNC Symbol;Acc:	0.488007204	0.941332957	-0.061127968	-1.36821219	3
343	ENSG00000198947	DMD	dystrophin [Source:HGNC Symbol;Acc:HGNC:2928]	0.455736926	1.195173021	-0.801859677	-0.84905027	3
344	ENSG00000203711	C6orf99	chromosome 6 open reading frame 99 [Source:HGNC Symbol;Acc:HGNC:2117	0.260818481	1.303757584	-0.718097886	-0.84647818	3
345	ENSG00000203995	ZYG11A	zyg-11 family member A, cell cycle regulator [Source:HGNC Symbol;Acc:HGNC	0.604044171	0.842085193	-0.059597238	-1.38653213	3
346	ENSG00000204264	PSMB8	proteasome (prosome, macropain) subunit, beta type, 8 [Source:HGNC Symb	0.491843501	0.663206716	0.331093711	-1.48614393	3
347	ENSG00000204516	MICB	MHC class I polypeptide-related sequence B [Source:HGNC Symbol;Acc:HGNC	0.646436906	0.890874363	-0.217838411	-1.31947286	3
348	ENSG00000204851	PNMAL2	paraneoplastic Ma antigen family-like 2 [Source:HGNC Symbol;Acc:HGNC:292	0.34287548	1.188316694	-0.379548742	-1.15164343	3
349	ENSG00000205639	MFSD2B	major facilitator superfamily domain containing 2B [Source:HGNC Symbol;Acc	0.520945327	0.644582671	0.32108925	-1.48661725	3
350	ENSG00000211455	STK38L	serine/threonine kinase 38 like [Source:HGNC Symbol;Acc:HGNC:17848]	0.259239723	1.215986441	-0.309813117	-1.16541305	3
351	ENSG00000213626	LBH	limb bud and heart development [Source:HGNC Symbol;Acc:HGNC:29532]	0.535202017	1.095252527	-0.511262066	-1.11919248	3
352	ENSG00000214944	ARHGEF28	Rho guanine nucleotide exchange factor (GEF) 28 [Source:HGNC Symbol;Acc:H	0.206955922	1.111980161	-0.007213041	-1.31172304	3
353	ENSG00000225830	ERCC6	excision repair cross-complementation group 6 [Source:HGNC Symbol;Acc:HG	0.542929699	0.83483867	0.038834476	-1.41660285	3
354	ENSG00000228253	MT-ATP8	mitochondrially encoded ATP synthase 8 [Source:HGNC Symbol;Acc:HGNC:74	0.616878172	0.627848797	0.229280395	-1.47400736	3
355	ENSG00000239887	C1orf226	chromosome 1 open reading frame 226 [Source:HGNC Symbol;Acc:HGNC:343	0.682433356	0.943889147	-0.412601923	-1.21372058	3
356	ENSG00000240694	PNMA2	paraneoplastic Ma antigen 2 [Source:HGNC Symbol;Acc:HGNC:9159]	0.59976674	1.074543481	-0.632241948	-1.04206827	3
357	ENSG00000241644	INMT	indolethylamine N-methyltransferase [Source:HGNC Symbol;Acc:HGNC:6069]	-0.11566712	1.433928321	-0.483682036	-0.83457916	3
358	ENSG00000243137	PSG4	pregnancy specific beta-1-glycoprotein 4 [Source:HGNC Symbol;Acc:HGNC:95	0.753823412	0.87631639	-0.405715555	-1.22442425	3
359	ENSG00000243251	PGBD3	piggyBac transposable element derived 3 [Source:HGNC Symbol;Acc:HGNC:1(	0.544013905	0.814417667	0.068482204	-1.42691378	3
360	ENSG00000249853	HS3ST5	heparan sulfate (glucosamine) 3-O-sulfotransferase 5 [Source:HGNC Symbol;/	0.664836339	0.665130124	0.119615126	-1.44958159	3

361	ENSG00000254087	LYN	LYN proto-oncogene, Src family tyrosine kinase [Source:HGNC Symbol;Acc:HG	0.730701412	0.745744041	-0.097911988	-1.37853347	3
362	ENSG00000254706	RP11-565P22.6	Uncharacterized protein {ECO:0000313|Ensembl:ENSP00000356909} [Source	0.653635059	0.972363817	-0.422273419	-1.20372546	3
363	ENSG00000255394	C8orf49	chromosome 8 open reading frame 49 [Source:HGNC Symbol;Acc:HGNC:3220	0.65338962	0.877365999	-0.203370458	-1.32738516	3
364	ENSG00000258659	TRIM34	tripartite motif containing 34 [Source:HGNC Symbol;Acc:HGNC:10063]	0.499852928	0.52801785	0.471733174	-1.49960395	3
365	ENSG00000258838	ERCC6-PGBD3	ERCC6-PGBD3 readthrough [Source:HGNC Symbol;Acc:HGNC:48347]	0.572514594	0.834210565	-0.000909045	-1.40581611	3
366	ENSG00000261115	TMEM178B	transmembrane protein 178B [Source:HGNC Symbol;Acc:HGNC:44112]	0.811635276	0.907927437	-0.721094682	-0.99846803	3
367	ENSG00000261150	EPPK1	epiplakin 1 [Source:HGNC Symbol;Acc:HGNC:15577]	0.409717798	1.223801912	-0.805649331	-0.82787038	3
368	ENSG00000263761	GDF2	growth differentiation factor 2 [Source:HGNC Symbol;Acc:HGNC:4217]	0.238854118	1.315967603	-0.742463158	-0.81235856	3
369	ENSG00000264424	MYH4	myosin, heavy chain 4, skeletal muscle [Source:HGNC Symbol;Acc:HGNC:7574	0.051599324	1.28220945	-0.185368555	-1.14844022	3
370	ENSG00000265107	GJA5	gap junction protein, alpha 5, 40kDa [Source:HGNC Symbol;Acc:HGNC:4279]	0.714000085	0.958836452	-0.54346307	-1.12937347	3
371	ENSG00000266728	AC015688.3	Uncharacterized protein {ECO:0000313|Ensembl:ENSP00000463557} [Source	0.628041728	0.992203158	-0.417375873	-1.20286901	3
372	ENSG00000268400	CTD-3214H19.4	Uncharacterized protein {ECO:0000313|Ensembl:ENSP00000472796} [Source	0.823345625	0.861742567	-0.559930736	-1.12515746	3
373	ENSG00000276644	DACH1	dachshund family transcription factor 1 [Source:HGNC Symbol;Acc:HGNC:266	-0.21334707	1.470013126	-0.583950318	-0.67271574	3
1	ENSG00000090376	IRAK3	interleukin-1 receptor-associated kinase 3 [Source:HGNC Symbol;Acc:HGNC:1	0.755686439	0.963939343	-0.962775341	-0.75685044	4
2	ENSG00000065361	ERBB3	v-erb-b2 avian erythroblastic leukemia viral oncogene homolog 3 [Source:HGI	0.420627926	1.214360133	-0.894333594	-0.74065447	4
3	ENSG00000099960	SLC7A4	solute carrier family 7, member 4 [Source:HGNC Symbol;Acc:HGNC:11062]	0.776662964	0.949029753	-0.922551857	-0.80314086	4
4	ENSG00000106689	LHX2	LIM homeobox 2 [Source:HGNC Symbol;Acc:HGNC:6594]	-0.08978823	1.441346378	-0.699273545	-0.6522846	4
5	ENSG00000112319	EYA4	EYA transcriptional coactivator and phosphatase 4 [Source:HGNC Symbol;Acc	0.144307131	1.313078572	-1.039348264	-0.41803744	4
6	ENSG00000117069	ST6GALNAC5	ST6 (alpha-N-acetyl-neuraminyl-2,3-beta-galactosyl-1,3)-N-acetylgalactosami	0.459006821	1.192457131	-0.868697864	-0.78276609	4
7	ENSG00000117266	CDK18	cyclin-dependent kinase 18 [Source:HGNC Symbol;Acc:HGNC:8751]	0.523441218	1.15038375	-0.866757984	-0.80706698	4
8	ENSG00000117525	F3	coagulation factor III (thromboplastin, tissue factor) [Source:HGNC Symbol;Ac	0.25231363	1.308473858	-0.836094747	-0.72469274	4
9	ENSG00000118898	PPL	periplakin [Source:HGNC Symbol;Acc:HGNC:9273]	0.336369975	1.258032598	-0.925944134	-0.66845844	4
10	ENSG00000122870	BICC1	BicC family RNA binding protein 1 [Source:HGNC Symbol;Acc:HGNC:19351]	0.285980437	1.288792414	-0.880323337	-0.69444951	4
11	ENSG00000130303	BST2	bone marrow stromal cell antigen 2 [Source:HGNC Symbol;Acc:HGNC:1119]	-0.02502441	1.40654025	-0.873412371	-0.50810347	4
12	ENSG00000132182	NUP210	nucleoporin 210kDa [Source:HGNC Symbol;Acc:HGNC:30052]	0.008068814	1.326862488	-1.084808462	-0.25012284	4
13	ENSG00000136040	PLXNC1	plexin C1 [Source:HGNC Symbol;Acc:HGNC:9106]	0.398487038	1.229864301	-0.852108769	-0.77624257	4
14	ENSG00000137975	CLCA2	chloride channel accessory 2 [Source:HGNC Symbol;Acc:HGNC:2016]	0.113699657	1.371760435	-0.774744532	-0.71071556	4
15	ENSG00000143061	IGSF3	immunoglobulin superfamily, member 3 [Source:HGNC Symbol;Acc:HGNC:59	0.677909904	1.031949469	-0.937721349	-0.77213802	4
16	ENSG00000143341	HMCN1	hemicentin 1 [Source:HGNC Symbol;Acc:HGNC:19194]	0.36065912	1.252506239	-0.80755337	-0.80561199	4
17	ENSG00000144802	NFKBIZ	nuclear factor of kappa light polypeptide gene enhancer in B-cells inhibitor, ze	0.67824888	1.034595703	-0.892972967	-0.81987162	4
18	ENSG00000145777	TSLP	thymic stromal lymphopoietin [Source:HGNC Symbol;Acc:HGNC:30743]	0.090868762	1.313413516	-1.076763246	-0.32751903	4
19	ENSG00000151468	CCDC3	coiled-coil domain containing 3 [Source:HGNC Symbol;Acc:HGNC:23813]	0.005766096	1.38370034	-0.939714475	-0.44975196	4
20	ENSG00000170412	GPRC5C	G protein-coupled receptor, class C, group 5, member C [Source:HGNC Symbo	0.72627367	0.985019323	-0.993489947	-0.71780305	4
21	ENSG00000171224	C10orf35	chromosome 10 open reading frame 35 [Source:HGNC Symbol;Acc:HGNC:235	0.571668986	1.105737688	-0.98650753	-0.69089914	4
22	ENSG00000173320	STOX2	storkhead box 2 [Source:HGNC Symbol;Acc:HGNC:25450]	0.266269038	1.27972067	-0.992509909	-0.5534798	4
23	ENSG00000175497	DPP10	dipeptidyl-peptidase 10 (non-functional) [Source:HGNC Symbol;Acc:HGNC:20	-0.13289111	1.452697071	-0.683003717	-0.63680224	4
24	ENSG00000178573	MAF	v-maf avian musculoaponeurotic fibrosarcoma oncogene homolog [Source:H(	0.789109977	0.939620254	-0.873451332	-0.8552789	4
25	ENSG00000178860	MSC	musculin [Source:HGNC Symbol;Acc:HGNC:7321]	0.575472962	0.980047828	-1.277017172	-0.27850362	4
26	ENSG00000180921	FAM83H	family with sequence similarity 83, member H [Source:HGNC Symbol;Acc:HGN	0.760673022	0.939589729	-1.065887003	-0.63437575	4
27	ENSG00000182255	KCNA4	potassium voltage-gated channel, shaker-related subfamily, member 4 [Sourc	0.495898533	1.168947564	-0.862339381	-0.80250672	4
28	ENSG00000182752	PAPPA	pregnancy-associated plasma protein A, pappalysin 1 [Source:HGNC Symbol;A	0.79845663	0.929232963	-0.921017539	-0.80667205	4
29	ENSG00000183914	DNAH2	dynein, axonemal, heavy chain 2 [Source:HGNC Symbol;Acc:HGNC:2948]	0.178729544	1.332769913	-0.913015116	-0.59848434	4
30	ENSG00000188176	SMTNL2	smoothelin-like 2 [Source:HGNC Symbol;Acc:HGNC:24764]	0.450495326	1.194060229	-0.919726856	-0.7248287	4
31	ENSG00000196208	GREB1	growth regulation by estrogen in breast cancer 1 [Source:HGNC Symbol;Acc:H	0.43689532	1.105739735	-1.216626781	-0.32600827	4
32	ENSG00000198691	ABCA4	ATP-binding cassette, sub-family A (ABC1), member 4 [Source:HGNC Symbol;A	0.575515721	1.076643419	-1.095157337	-0.5570018	4
33	ENSG00000198796	ALPK2	alpha-kinase 2 [Source:HGNC Symbol;Acc:HGNC:20565]	0.571487335	0.693058907	-1.466998518	0.202452276	4
1	ENSG00000047365	ARAP2	ArfGAP with RhoGAP domain, ankyrin repeat and PH domain 2 [Source:HGNC	-0.12738123	0.445563263	1.010230903	-1.32841293	5
2	ENSG00000013016	EHD3	EH-domain containing 3 [Source:HGNC Symbol;Acc:HGNC:3244]	-0.31170196	0.040276019	1.332455155	-1.06102921	5
3	ENSG00000067715	SYT1	synaptotagmin I [Source:HGNC Symbol;Acc:HGNC:11509]	-0.01241603	0.115647853	1.169273728	-1.27250555	5
4	ENSG00000078018	MAP2	microtubule-associated protein 2 [Source:HGNC Symbol;Acc:HGNC:6839]	-0.29902298	-0.217147249	1.42646358	-0.91029335	5
5	ENSG00000081189	MEF2C	myocyte enhancer factor 2C [Source:HGNC Symbol;Acc:HGNC:6996]	-0.42367527	-0.079579378	1.410836918	-0.90758227	5
6	ENSG00000081277	PKP1	plakophilin 1 [Source:HGNC Symbol;Acc:HGNC:9023]	-0.27580404	-0.261756886	1.433024765	-0.89546384	5
7	ENSG00000084234	APLP2	amyloid beta (A4) precursor-like protein 2 [Source:HGNC Symbol;Acc:HGNC:5	-0.17972821	-0.167949855	1.373641224	-1.02596316	5
8	ENSG00000091592	NLRP1	NLR family, pyrin domain containing 1 [Source:HGNC Symbol;Acc:HGNC:1437	0.039370455	0.266734721	1.047041652	-1.35314683	5
9	ENSG00000101670	LIPG	lipase, endothelial [Source:HGNC Symbol;Acc:HGNC:6623]	-0.50009584	-0.25566786	1.473087016	-0.71732331	5
10	ENSG00000105339	DENND3	DENN/MADD domain containing 3 [Source:HGNC Symbol;Acc:HGNC:29134]	-0.02834318	0.271680332	1.081614431	-1.32495158	5
11	ENSG00000106123	EPHB6	EPH receptor B6 [Source:HGNC Symbol;Acc:HGNC:3396]	-0.53447035	-0.389902584	1.495405892	-0.57103295	5
12	ENSG00000106714	CNTNAP3	contactin associated protein-like 3 [Source:HGNC Symbol;Acc:HGNC:13834]	0.147540816	0.641011457	0.667898208	-1.45645048	5
13	ENSG00000113532	ST8SIA4	ST8 alpha-N-acetyl-neuraminide alpha-2,8-sialyltransferase 4 [Source:HGNC S	-0.197454	0.17396492	1.222213924	-1.19872484	5
14	ENSG00000115896	PLCL1	phospholipase C-like 1 [Source:HGNC Symbol;Acc:HGNC:9063]	-0.48781255	0.241719321	1.279188851	-1.03309563	5
15	ENSG00000117152	RGS4	regulator of G-protein signaling 4 [Source:HGNC Symbol;Acc:HGNC:10000]	0.165730555	0.495245266	0.789516775	-1.4504926	5
16	ENSG00000126016	AMOT	angiomotin [Source:HGNC Symbol;Acc:HGNC:17810]	-0.40726907	0.334413076	1.202542442	-1.12968645	5
17	ENSG00000126950	TMEM35	transmembrane protein 35 [Source:HGNC Symbol;Acc:HGNC:25864]	-0.4609868	-0.397756113	1.492518095	-0.63377518	5
18	ENSG00000128512	DOCK4	dedicator of cytokinesis 4 [Source:HGNC Symbol;Acc:HGNC:19192]	-0.41174691	-0.236379631	1.456440227	-0.80831369	5
19	ENSG00000128606	LRRC17	leucine rich repeat containing 17 [Source:HGNC Symbol;Acc:HGNC:16895]	-0.45355849	-0.284967879	1.473882441	-0.73535607	5
20	ENSG00000128849	CGNL1	cingulin-like 1 [Source:HGNC Symbol;Acc:HGNC:25931]	0.202450774	0.428135095	0.819805025	-1.45039089	5
21	ENSG00000135454	B4GALNT1	beta-1,4-N-acetyl-galactosaminyl transferase 1 [Source:HGNC Symbol;Acc:HG	-0.40452822	0.211032223	1.274266994	-1.080771	5
22	ENSG00000138735	PDE5A	phosphodiesterase 5A, cGMP-specific [Source:HGNC Symbol;Acc:HGNC:8784]	-0.49721888	0.061915333	1.369649014	-0.93434547	5
23	ENSG00000139292	LGR5	leucine-rich repeat containing G protein-coupled receptor 5 [Source:HGNC Sy	-0.65215425	0.042088405	1.397469015	-0.78740317	5
24	ENSG00000141668	CBLN2	cerebellin 2 precursor [Source:HGNC Symbol;Acc:HGNC:1544]	-0.31815822	-0.134699202	1.405006573	-0.95214915	5
25	ENSG00000143786	CNIH3	cornichon family AMPA receptor auxiliary protein 3 [Source:HGNC Symbol;Ac	-0.46704524	-0.191088213	1.453518642	-0.79538519	5
26	ENSG00000143867	OSR1	odd-skipped related transciption factor 1 [Source:HGNC Symbol;Acc:HGNC:81	-0.52353408	-0.010607948	1.403544878	-0.86940285	5
27	ENSG00000144057	ST6GAL2	ST6 beta-galactosamide alpha-2,6-sialyltranferase 2 [Source:HGNC Symbol;Ac	-0.23247568	0.601757857	0.936888644	-1.30617082	5
28	ENSG00000146411	SLC2A12	solute carrier family 2 (facilitated glucose transporter), member 12 [Source:H	-0.10650527	0.709042664	0.772134648	-1.37467204	5
29	ENSG00000149527	PLCH2	phospholipase C, eta 2 [Source:HGNC Symbol;Acc:HGNC:29037]	-0.56045881	-0.139132874	1.450207971	-0.75061628	5
30	ENSG00000149633	KIAA1755	KIAA1755 [Source:HGNC Symbol;Acc:HGNC:29372]	-0.50866428	0.301161512	1.250275194	-1.04277243	5
31	ENSG00000151150	ANK3	ankyrin 3, node of Ranvier (ankyrin G) [Source:HGNC Symbol;Acc:HGNC:494]	0.059870521	0.534978129	0.828029242	-1.42287789	5
32	ENSG00000152137	HSPB8	heat shock 22kDa protein 8 [Source:HGNC Symbol;Acc:HGNC:30171]	0.007057844	0.517637289	0.876588194	-1.40128333	5
33	ENSG00000154133	ROBO4	roundabout, axon guidance receptor, homolog 4 (Drosophila) [Source:HGNC S	0.126251044	0.652329781	0.672649435	-1.45123026	5
34	ENSG00000154258	ABCA9	ATP-binding cassette, sub-family A (ABC1), member 9 [Source:HGNC Symbol;	-0.30566946	0.725472185	0.860645021	-1.28044775	5
35	ENSG00000154263	ABCA10	ATP-binding cassette, sub-family A (ABC1), member 10 [Source:HGNC Symbol	-0.37077379	0.60492493	0.994045318	-1.22819645	5
36	ENSG00000155980	KIF5A	kinesin family member 5A [Source:HGNC Symbol;Acc:HGNC:6323]	-0.44249054	-0.131273812	1.431951289	-0.85818693	5
37	ENSG00000158458	NRG2	neuregulin 2 [Source:HGNC Symbol;Acc:HGNC:7998]	-0.12457396	0.431279872	1.019558963	-1.32626488	5
38	ENSG00000161040	FBXL13	F-box and leucine-rich repeat protein 13 [Source:HGNC Symbol;Acc:HGNC:21	-0.28618134	-0.174603565	1.409683018	-0.94889811	5

39	ENSG00000162738	VANGL2	VANGL planar cell polarity protein 2 [Source:HGNC Symbol;Acc:HGNC:15511]	0.325762332	0.363365074	0.778912251	-1.46803966	5
40	ENSG00000163288	GABRB1	gamma-aminobutyric acid (GABA) A receptor, beta 1 [Source:HGNC Symbol;A	-0.43028262	-0.409702403	1.491023181	-0.65103816	5
41	ENSG00000163697	APBB2	amyloid beta (A4) precursor protein-binding, family B, member 2 [Source:HGI	0.171021525	0.502320988	0.779512016	-1.45285453	5
42	ENSG00000164035	EMCN	endomucin [Source:HGNC Symbol;Acc:HGNC:16041]	-0.53014285	-0.26940665	1.47836849	-0.67881899	5
43	ENSG00000164418	GRIK2	glutamate receptor, ionotropic, kainate 2 [Source:HGNC Symbol;Acc:HGNC:4!	-0.15365757	0.116954595	1.235322609	-1.19861963	5
44	ENSG00000166428	PLD4	phospholipase D family, member 4 [Source:HGNC Symbol;Acc:HGNC:23792]	-0.29316351	0.612416806	0.955729682	-1.27498297	5
45	ENSG00000166510	CCDC68	coiled-coil domain containing 68 [Source:HGNC Symbol;Acc:HGNC:24350]	-0.10057261	0.413427427	1.020437889	-1.3332927	5
46	ENSG00000168490	PHYHIP	phytanoyl-CoA 2-hydroxylase interacting protein [Source:HGNC Symbol;Acc:H	0.304016226	0.446762752	0.726005681	-1.47678466	5
47	ENSG00000169083	AR	androgen receptor [Source:HGNC Symbol;Acc:HGNC:644]	-0.60368089	-0.105149855	1.443748283	-0.73491754	5
48	ENSG00000169432	SCN9A	sodium channel, voltage-gated, type IX, alpha subunit [Source:HGNC Symbol;	0.000868011	0.460450427	0.927265066	-1.3885835	5
49	ENSG00000171303	KCNK3	potassium channel, subfamily K, member 3 [Source:HGNC Symbol;Acc:HGNC:	-0.52482858	-0.389953011	1.495195915	-0.58041432	5
50	ENSG00000171345	KRT19	keratin 19 [Source:HGNC Symbol;Acc:HGNC:6436]	-0.31376269	0.550890888	1.015004889	-1.25213309	5
51	ENSG00000171517	LPAR3	lysophosphatidic acid receptor 3 [Source:HGNC Symbol;Acc:HGNC:14298]	0.097884378	0.208972751	1.050662044	-1.35751917	5
52	ENSG00000171522	PTGER4	prostaglandin E receptor 4 (subtype EP4) [Source:HGNC Symbol;Acc:HGNC:95	-0.51906708	-0.300744486	1.483236258	-0.66342469	5
53	ENSG00000171860	C3AR1	complement component 3a receptor 1 [Source:HGNC Symbol;Acc:HGNC:131!	-0.00879791	-0.005683349	1.231941701	-1.21746045	5
54	ENSG00000172995	ARPP21	cAMP-regulated phosphoprotein, 21kDa [Source:HGNC Symbol;Acc:HGNC:16	-0.35231745	-0.264469457	1.452009489	-0.83522259	5
55	ENSG00000173597	SULT1B1	sulfotransferase family, cytosolic, 1B, member 1 [Source:HGNC Symbol;Acc:H	0.368077481	0.516831539	0.607753122	-1.49266214	5
56	ENSG00000174611	KY	kyphoscoliosis peptidase [Source:HGNC Symbol;Acc:HGNC:26576]	-0.43483547	0.03304815	1.369035221	-0.9672479	5
57	ENSG00000174804	FZD4	frizzled class receptor 4 [Source:HGNC Symbol;Acc:HGNC:4042]	-0.12065747	0.654619884	0.831735465	-1.36569788	5
58	ENSG00000176595	KBTBD11	kelch repeat and BTB (POZ) domain containing 11 [Source:HGNC Symbol;Acc:	0.044409891	0.552836893	0.822547262	-1.41979405	5
59	ENSG00000177494	ZBED2	zinc finger, BED-type containing 2 [Source:HGNC Symbol;Acc:HGNC:20710]	-0.26041123	-0.052652353	1.356617062	-1.04355348	5
60	ENSG00000178343	SHISA3	shisa family member 3 [Source:HGNC Symbol;Acc:HGNC:25159]	-0.5193665	0.245560013	1.284176909	-1.01037042	5
61	ENSG00000179855	GIPC3	GIPC PDZ domain containing family, member 3 [Source:HGNC Symbol;Acc:HG	-0.46844815	0.503006502	1.106763449	-1.1413218	5
62	ENSG00000181072	CHRM2	cholinergic receptor, muscarinic 2 [Source:HGNC Symbol;Acc:HGNC:1951]	-0.68245904	0.196317744	1.333378477	-0.84723718	5
63	ENSG00000181444	ZNF467	zinc finger protein 467 [Source:HGNC Symbol;Acc:HGNC:23154]	0.360180798	0.484667729	0.644928178	-1.4897767	5
64	ENSG00000182179	UBA7	ubiquitin-like modifier activating enzyme 7 [Source:HGNC Symbol;Acc:HGNC:	0.028301463	0.598817505	0.791876521	-1.41899549	5
65	ENSG00000182511	FES	FES proto-oncogene, tyrosine kinase [Source:HGNC Symbol;Acc:HGNC:3657]	-0.21210608	0.126700246	1.254171235	-1.16876541	5
66	ENSG00000184374	COLEC10	collectin sub-family member 10 (C-type lectin) [Source:HGNC Symbol;Acc:HGI	-0.18101707	-0.078433788	1.339565004	-1.08011414	5
67	ENSG00000184408	KCND2	potassium voltage-gated channel, Shal-related subfamily, member 2 [Source:I	-0.60771144	0.306880305	1.26649388	-0.96566274	5
68	ENSG00000185567	AHNAK2	AHNAK nucleoprotein 2 [Source:HGNC Symbol;Acc:HGNC:20125]	-0.18414708	0.622964675	0.894531155	-1.33334875	5
69	ENSG00000188153	COL4A5	collagen, type IV, alpha 5 [Source:HGNC Symbol;Acc:HGNC:2207]	-0.16128991	0.033470193	1.281427082	-1.15360736	5
70	ENSG00000196468	FGF16	fibroblast growth factor 16 [Source:HGNC Symbol;Acc:HGNC:3672]	-0.48289477	-0.444903674	1.497937032	-0.57013859	5
71	ENSG00000196611	MMP1	matrix metallopeptidase 1 (interstitial collagenase) [Source:HGNC Symbol;Acc	-0.31708387	-0.091374646	1.389068766	-0.98061025	5
72	ENSG00000197077	KIAA1671	KIAA1671 [Source:HGNC Symbol;Acc:HGNC:29345]	0.344785889	0.514043737	0.630794497	-1.48962412	5
73	ENSG00000197355	UAP1L1	UDP-N-acetylglucosamine pyrophosphorylase 1 like 1 [Source:HGNC Symbol;/	0.214645078	0.439191786	0.801639662	-1.45547653	5
74	ENSG00000197872	FAM49A	family with sequence similarity 49, member A [Source:HGNC Symbol;Acc:HGN	-0.01457805	0.621120209	0.799010753	-1.40555291	5
75	ENSG00000198168	SVIP	small VCP/p97-interacting protein [Source:HGNC Symbol;Acc:HGNC:25238]	0.102034031	0.367306148	0.936768161	-1.40610834	5
76	ENSG00000198829	SUCNR1	succinate receptor 1 [Source:HGNC Symbol;Acc:HGNC:4542]	-0.39589407	-0.378278515	1.482645888	-0.70847331	5
77	ENSG00000198959	TGM2	transglutaminase 2 [Source:HGNC Symbol;Acc:HGNC:11778]	-0.32977338	-0.305392473	1.456939572	-0.82177371	5
78	ENSG00000249751	ECSCR	endothelial cell surface expressed chemotaxis and apoptosis regulator [Sourc	0.422395475	0.435905105	0.634623384	-1.49292396	5
79	ENSG00000261371	PECAM1	platelet/endothelial cell adhesion molecule 1 [Source:HGNC Symbol;Acc:HGN	-0.1120885	0.691159978	0.792608466	-1.37167995	5
1	ENSG00000003096	KLHL13	kelch-like family member 13 [Source:HGNC Symbol;Acc:HGNC:22931]	-0.04700438	1.047451991	0.336492154	-1.33693977	6
2	ENSG00000002587	HS3ST1	heparan sulfate (glucosamine) 3-O-sulfotransferase 1 [Source:HGNC Symbol;/	0.311688854	0.70750844	0.460684895	-1.47988219	6
3	ENSG00000065534	MYLK	myosin light chain kinase [Source:HGNC Symbol;Acc:HGNC:7590]	-0.47886173	1.240131771	0.306023045	-1.06729309	6
4	ENSG00000073849	ST6GAL1	ST6 beta-galactosamide alpha-2,6-sialyltranferase 1 [Source:HGNC Symbol;Ac	0.293578708	0.836700975	0.322265629	-1.45254531	6
5	ENSG00000075275	CELSR1	cadherin, EGF LAG seven-pass G-type receptor 1 [Source:HGNC Symbol;Acc:H	-0.00765734	0.762878525	0.655050583	-1.41027177	6
6	ENSG00000101842	VSIG1	V-set and immunoglobulin domain containing 1 [Source:HGNC Symbol;Acc:H(	0.04835906	1.130009458	0.127217809	-1.30558633	6
7	ENSG00000101844	ATG4A	autophagy related 4A, cysteine peptidase [Source:HGNC Symbol;Acc:HGNC:1(	0.089218506	0.891786106	0.435665221	-1.41666983	6
8	ENSG00000102271	KLHL4	kelch-like family member 4 [Source:HGNC Symbol;Acc:HGNC:6355]	0.284798298	0.69064013	0.503786226	-1.47922465	6
9	ENSG00000105122	RASAL3	RAS protein activator like 3 [Source:HGNC Symbol;Acc:HGNC:26129]	0.216872829	0.938786736	0.259986165	-1.41564573	6
10	ENSG00000106484	MEST	mesoderm specific transcript [Source:HGNC Symbol;Acc:HGNC:7028]	-0.08313906	1.24754743	0.033746249	-1.19815462	6
11	ENSG00000107719	PALD1	phosphatase domain containing, paladin 1 [Source:HGNC Symbol;Acc:HGNC:2	-0.53831132	1.281501206	0.257614869	-1.00080476	6
12	ENSG00000115457	IGFBP2	insulin-like growth factor binding protein 2, 36kDa [Source:HGNC Symbol;Acc	-0.10964487	1.303262484	-0.059629412	-1.1339882	6
13	ENSG00000120278	PLEKHG1	pleckstrin homology domain containing, family G (with RhoGef domain) mem	0.298645889	0.850434286	0.299353366	-1.44843354	6
14	ENSG00000122862	SRGN	serglycin [Source:HGNC Symbol;Acc:HGNC:9361]	0.067004033	1.013899063	0.291216473	-1.37211957	6
15	ENSG00000129422	MTUS1	microtubule associated tumor suppressor 1 [Source:HGNC Symbol;Acc:HGNC	-0.21808664	1.280954549	0.079610055	-1.14247796	6
16	ENSG00000136153	LMO7	LIM domain 7 [Source:HGNC Symbol;Acc:HGNC:6646]	0.068967555	1.14294633	0.084893812	-1.2968077	6
17	ENSG00000137642	SORL1	sortilin-related receptor, L(DLR class) A repeats containing [Source:HGNC Sym	0.043562167	1.038955415	0.275059722	-1.3575773	6
18	ENSG00000139132	FGD4	FYVE, RhoGEF and PH domain containing 4 [Source:HGNC Symbol;Acc:HGNC:	-0.03571717	0.973742925	0.428373006	-1.36639876	6
19	ENSG00000139946	PELI2	pellino E3 ubiquitin protein ligase family member 2 [Source:HGNC Symbol;Ac	0.028200534	0.907175511	0.464720994	-1.40009704	6
20	ENSG00000141338	ABCA8	ATP-binding cassette, sub-family A (ABC1), member 8 [Source:HGNC Symbol;/	0.067993094	0.788806643	0.573125998	-1.42992574	6
21	ENSG00000141469	SLC14A1	solute carrier family 14 (urea transporter), member 1 (Kidd blood group) [Sou	-0.05723764	1.106059464	0.257683297	-1.30650512	6
22	ENSG00000146054	TRIM7	tripartite motif containing 7 [Source:HGNC Symbol;Acc:HGNC:16278]	0.025046261	0.825760086	0.563423718	-1.41423007	6
23	ENSG00000149970	CNKSR2	connector enhancer of kinase suppressor of Ras 2 [Source:HGNC Symbol;Acc:	-0.13922379	0.979409285	0.493257772	-1.33344327	6
24	ENSG00000151012	SLC7A11	solute carrier family 7 (anionic amino acid transporter light chain, xc- system)	0.271479004	0.812319103	0.374378103	-1.45817621	6
25	ENSG00000151690	MFSD6	major facilitator superfamily domain containing 6 [Source:HGNC Symbol;Acc:	-0.13213625	1.180513167	0.196707374	-1.24508429	6
26	ENSG00000153253	SCN3A	sodium channel, voltage-gated, type III, alpha subunit [Source:HGNC Symbol;	-0.22304984	1.382201531	-0.15065197	-1.00849972	6
27	ENSG00000154262	ABCA6	ATP-binding cassette, sub-family A (ABC1), member 6 [Source:HGNC Symbol;/	-0.22632579	1.257061696	0.131688565	-1.16242447	6
28	ENSG00000160796	NBEAL2	neurobeachin-like 2 [Source:HGNC Symbol;Acc:HGNC:31928]	0.293122168	0.852348528	0.302344598	-1.44781529	6
29	ENSG00000163995	ABLIM2	actin binding LIM protein family, member 2 [Source:HGNC Symbol;Acc:HGNC:	-0.02407624	1.184579329	0.099015871	-1.25951896	6
30	ENSG00000164161	HHIP	hedgehog interacting protein [Source:HGNC Symbol;Acc:HGNC:14866]	0.065436231	0.703219748	0.66585868	-1.43451466	6
31	ENSG00000164176	EDIL3	EGF-like repeats and discoidin I-like domains 3 [Source:HGNC Symbol;Acc:HGI	0.255315781	0.875127241	0.309425689	-1.43986871	6
32	ENSG00000165376	CLDN2	claudin 2 [Source:HGNC Symbol;Acc:HGNC:2041]	-0.09591934	0.849125768	0.619921983	-1.37312842	6
33	ENSG00000167941	SOST	sclerostin [Source:HGNC Symbol;Acc:HGNC:13771]	-0.06395933	0.942003374	0.489163891	-1.36720793	6
34	ENSG00000168071	CCDC88B	coiled-coil domain containing 88B [Source:HGNC Symbol;Acc:HGNC:26757]	0.241761731	0.92299931	0.257646086	-1.42240713	6
35	ENSG00000170011	MYRIP	myosin VIIA and Rab interacting protein [Source:HGNC Symbol;Acc:HGNC:191	-0.15055899	1.1139951	0.315853994	-1.2792901	6
36	ENSG00000170276	HSPB2	heat shock 27kDa protein 2 [Source:HGNC Symbol;Acc:HGNC:5247]	-0.15561495	1.106758774	0.330198438	-1.28134226	6
37	ENSG00000170373	CST1	cystatin SN [Source:HGNC Symbol;Acc:HGNC:2473]	-0.49015426	1.455976145	-0.188397522	-0.77742436	6
38	ENSG00000171843	MLLT3	myeloid/lymphoid or mixed-lineage leukemia (trithorax homolog, Drosophila)	0.20061275	0.965719536	0.237496639	-1.40382892	6
39	ENSG00000173193	PARP14	poly (ADP-ribose) polymerase family, member 14 [Source:HGNC Symbol;Acc:I	-0.1164475	0.989428581	0.465291883	-1.33827296	6
40	ENSG00000175294	CATSPER1	cation channel, sperm associated 1 [Source:HGNC Symbol;Acc:HGNC:17116]	0.07184542	1.145824951	0.076935495	-1.29460587	6
41	ENSG00000175538	KCNE3	potassium voltage-gated channel, Isk-related family, member 3 [Source:HGN(	-0.52121348	1.491237797	-0.354488684	-0.61553563	6
42	ENSG00000175745	NR2F1	nuclear receptor subfamily 2, group F, member 1 [Source:HGNC Symbol;Acc:I	0.213519596	0.64621584	0.611103454	-1.47083889	6
43	ENSG00000178033	FAM26E	family with sequence similarity 26, member E [Source:HGNC Symbol;Acc:HGN	0.365796883	0.717297693	0.397901947	-1.48099652	6

44	ENSG00000182253	SYNM	synemin, intermediate filament protein [Source:HGNC Symbol;Acc:HGNC:244	-0.28874022	0.915839208	0.656306439	-1.28340543	6
45	ENSG00000182985	CADM1	cell adhesion molecule 1 [Source:HGNC Symbol;Acc:HGNC:5951]	-0.10712566	0.776810134	0.704593058	-1.37427753	6
46	ENSG00000183873	SCN5A	sodium channel, voltage-gated, type V, alpha subunit [Source:HGNC Symbol;A	-0.49914085	0.956814017	0.701387312	-1.15906047	6
47	ENSG00000184349	EFNA5	ephrin-A5 [Source:HGNC Symbol;Acc:HGNC:3225]	0.172772914	1.00220858	0.211081957	-1.38606345	6
48	ENSG00000185088	RPS27L	ribosomal protein S27-like [Source:HGNC Symbol;Acc:HGNC:18476]	0.086777202	0.941124198	0.374436692	-1.40233809	6
49	ENSG00000187398	LUZP2	leucine zipper protein 2 [Source:HGNC Symbol;Acc:HGNC:23206]	0.097328708	0.998223821	0.287076613	-1.38262914	6
50	ENSG00000188158	NHS	Nance-Horan syndrome (congenital cataracts and dental anomalies) [Source:I	0.02146476	0.776801893	0.620058328	-1.41832498	6
51	ENSG00000196139	AKR1C3	aldo-keto reductase family 1, member C3 [Source:HGNC Symbol;Acc:HGNC:3	-0.15013904	0.985801439	0.492247908	-1.32791031	6
52	ENSG00000204539	CDSN	corneodesmosin [Source:HGNC Symbol;Acc:HGNC:1802]	0.313893223	0.809801427	0.336581264	-1.46027591	6
53	ENSG00000204634	TBC1D8	TBC1 domain family, member 8 (with GRAM domain) [Source:HGNC Symbol;A	0.470925151	0.542267168	0.486101298	-1.49929362	6
54	ENSG00000260001	TGFBR3L	transforming growth factor, beta receptor III-like [Source:HGNC Symbol;Acc:H	0.106014019	0.763660742	0.571993341	-1.4416681	6
1	ENSG00000146674	IGFBP3	insulin-like growth factor binding protein 3 [Source:HGNC Symbol;Acc:HGNC:	1.497588932	-0.575029695	-0.438802232	-0.483757	7
2	ENSG00000103449	SALL1	spalt-like transcription factor 1 [Source:HGNC Symbol;Acc:HGNC:10524]	1.134334216	-0.943613232	0.538947283	-0.72966827	7
3	ENSG00000162723	SLAMF9	SLAM family member 9 [Source:HGNC Symbol;Acc:HGNC:18430]	0.911293687	-1.396051401	0.469413303	0.015344411	7
4	ENSG00000163132	MSX1	msh homeobox 1 [Source:HGNC Symbol;Acc:HGNC:7391]	1.197582234	-1.243176911	0.120951618	-0.07535694	7
1	ENSG00000113430	IRX4	iroquois homeobox 4 [Source:HGNC Symbol;Acc:HGNC:6129]	0.589743328	-0.85589165	1.103701491	-0.83755317	8
2	ENSG00000081803	CADPS2	Ca++-dependent secretion activator 2 [Source:HGNC Symbol;Acc:HGNC:1601	0.001295454	-0.978325176	1.373270373	-0.39624065	8
3	ENSG00000121005	CRISPLD1	cysteine-rich secretory protein LCCL domain containing 1 [Source:HGNC Symb	0.274613006	-1.214700908	1.179694136	-0.23960623	8
4	ENSG00000154319	FAM167A	family with sequence similarity 167, member A [Source:HGNC Symbol;Acc:HG	0.391327561	-1.451748239	0.827736625	0.232684054	8
5	ENSG00000154654	NCAM2	neural cell adhesion molecule 2 [Source:HGNC Symbol;Acc:HGNC:7657]	-0.30328836	-0.782475305	1.466544974	-0.38078131	8
6	ENSG00000158089	GALNT14	polypeptide N-acetylgalactosaminyltransferase 14 [Source:HGNC Symbol;Acc	0.407932798	-1.418032726	0.900412511	0.109687418	8
7	ENSG00000164690	SHH	sonic hedgehog [Source:HGNC Symbol;Acc:HGNC:10848]	-0.24789332	-0.68949774	1.47462564	-0.53723458	8
8	ENSG00000176165	FOXG1	forkhead box G1 [Source:HGNC Symbol;Acc:HGNC:3811]	-0.49208169	-0.512448081	1.49994032	-0.49541055	8
9	ENSG00000185352	HS6ST3	heparan sulfate 6-O-sulfotransferase 3 [Source:HGNC Symbol;Acc:HGNC:191	-0.38942778	-0.560974615	1.49550045	-0.54509805	8
10	ENSG00000188906	LRRK2	leucine-rich repeat kinase 2 [Source:HGNC Symbol;Acc:HGNC:18618]	-0.28814662	-0.849243914	1.448731541	-0.31134101	8
11	ENSG00000221887	HMSD	histocompatibility (minor) serpin domain containing [Source:HGNC Symbol;A	-0.3069135	-0.739501395	1.47490323	-0.42848834	8
1	ENSG00000040731	CDH10	cadherin 10, type 2 (T2-cadherin) [Source:HGNC Symbol;Acc:HGNC:1749]	0.261212958	0.210410621	0.942381904	-1.41400548	9
2	ENSG00000019102	VSIG2	V-set and immunoglobulin domain containing 2 [Source:HGNC Symbol;Acc:H	0.297900257	-0.152780717	1.126895033	-1.27201457	9
3	ENSG00000050344	NFE2L3	nuclear factor, erythroid 2-like 3 [Source:HGNC Symbol;Acc:HGNC:7783]	0.463110804	0.341276425	0.680751301	-1.48513853	9
4	ENSG00000055813	CCDC85A	coiled-coil domain containing 85A [Source:HGNC Symbol;Acc:HGNC:29400]	0.060602555	-0.540304099	1.377686882	-0.89798534	9
5	ENSG00000061455	PRDM6	PR domain containing 6 [Source:HGNC Symbol;Acc:HGNC:9350]	-0.49601803	-0.50067198	1.499993195	-0.50330319	9
6	ENSG00000074966	TXK	TXK tyrosine kinase [Source:HGNC Symbol;Acc:HGNC:12434]	-0.06469257	-0.493959185	1.418574161	-0.85992241	9
7	ENSG00000076356	PLXNA2	plexin A2 [Source:HGNC Symbol;Acc:HGNC:9100]	0.234407015	-0.160898604	1.170818411	-1.24432682	9
8	ENSG00000089225	TBX5	T-box 5 [Source:HGNC Symbol;Acc:HGNC:11604]	0.494483947	-0.121740587	0.969309082	-1.34205244	9
9	ENSG00000099937	SERPIND1	serpin peptidase inhibitor, clade D (heparin cofactor), member 1 [Source:HGN	0.386546349	-0.253818007	1.112056368	-1.24478471	9
10	ENSG00000100558	PLEK2	pleckstrin 2 [Source:HGNC Symbol;Acc:HGNC:19238]	-0.12882653	-0.58161743	1.449013456	-0.73856949	9
11	ENSG00000101384	JAG1	jagged 1 [Source:HGNC Symbol;Acc:HGNC:6188]	0.485015511	0.103143145	0.841956795	-1.43011545	9
12	ENSG00000101955	SRPX	sushi-repeat containing protein, X-linked [Source:HGNC Symbol;Acc:HGNC:11	0.122781829	0.082049248	1.113564449	-1.31839553	9
13	ENSG00000102755	FLT1	fms-related tyrosine kinase 1 [Source:HGNC Symbol;Acc:HGNC:3763]	0.00240214	-0.520854352	1.398152931	-0.87970072	9
14	ENSG00000102802	MEDAG	mesenteric estrogen-dependent adipogenesis [Source:HGNC Symbol;Acc:HGN	0.356061581	-0.253445912	1.132693925	-1.23530959	9
15	ENSG00000104967	NOVA2	neuro-oncological ventral antigen 2 [Source:HGNC Symbol;Acc:HGNC:7887]	0.150435772	-0.105954158	1.195370274	-1.23985189	9
16	ENSG00000105605	CACNG7	calcium channel, voltage-dependent, gamma subunit 7 [Source:HGNC Symbo	0.18357921	0.005646318	1.119546131	-1.30877166	9
17	ENSG00000106025	TSPAN12	tetraspanin 12 [Source:HGNC Symbol;Acc:HGNC:21641]	0.582944883	0.170448667	0.70665949	-1.46005304	9
18	ENSG00000108797	CNTNAP1	contactin associated protein 1 [Source:HGNC Symbol;Acc:HGNC:8011]	0.531195146	0.357243018	0.603528015	-1.49196618	9
19	ENSG00000111348	ARHGDIB	Rho GDP dissociation inhibitor (GDI) beta [Source:HGNC Symbol;Acc:HGNC:67	0.496510882	0.385592531	0.611502835	-1.49360625	9
20	ENSG00000111846	GCNT2	glucosaminyl (N-acetyl) transferase 2, I-branching enzyme (I blood group) [So	-0.37203655	-0.394260062	1.481452125	-0.71515552	9
21	ENSG00000114115	RBP1	retinol binding protein 1, cellular [Source:HGNC Symbol;Acc:HGNC:9919]	-0.04814021	-0.255821836	1.353238958	-1.04927691	9
22	ENSG00000117122	MFAP2	microfibrillar-associated protein 2 [Source:HGNC Symbol;Acc:HGNC:7033]	0.522455746	-0.099401547	0.934699732	-1.35775393	9
23	ENSG00000117595	IRF6	interferon regulatory factor 6 [Source:HGNC Symbol;Acc:HGNC:6121]	-0.36799251	-0.414989184	1.483698548	-0.70071685	9
24	ENSG00000118946	PCDH17	protocadherin 17 [Source:HGNC Symbol;Acc:HGNC:14267]	0.017449233	-0.163153234	1.289910281	-1.14420628	9
25	ENSG00000119139	TJP2	tight junction protein 2 [Source:HGNC Symbol;Acc:HGNC:11828]	0.1813766	0.060020161	1.090574486	-1.33197125	9
26	ENSG00000119698	PPP4R4	protein phosphatase 4, regulatory subunit 4 [Source:HGNC Symbol;Acc:HGNC	-0.14155812	-0.641622909	1.45498016	-0.67179913	9
27	ENSG00000125810	CD93	CD93 molecule [Source:HGNC Symbol;Acc:HGNC:15855]	0.695978646	-0.263099566	0.868157204	-1.30103628	9
28	ENSG00000127324	TSPAN8	tetraspanin 8 [Source:HGNC Symbol;Acc:HGNC:11855]	-0.20293576	-0.405420415	1.446413347	-0.83805715	9
29	ENSG00000127329	PTPRB	protein tyrosine phosphatase, receptor type, B [Source:HGNC Symbol;Acc:HG	0.420200379	0.068925801	0.917129353	-1.40625553	9
30	ENSG00000127951	FGL2	fibrinogen-like 2 [Source:HGNC Symbol;Acc:HGNC:3696]	0.014277113	-0.685202598	1.409060214	-0.73813473	9
31	ENSG00000128567	PODXL	podocalyxin-like [Source:HGNC Symbol;Acc:HGNC:9171]	0.049749845	-0.628149703	1.392757745	-0.81435789	9
32	ENSG00000130300	PLVAP	plasmalemma vesicle associated protein [Source:HGNC Symbol;Acc:HGNC:13	0.4589908	-0.167036286	1.019957477	-1.31191199	9
33	ENSG00000130787	HIP1R	huntingtin interacting protein 1 related [Source:HGNC Symbol;Acc:HGNC:184	0.454053074	0.245316629	0.765908391	-1.46527809	9
34	ENSG00000132357	CARD6	caspase recruitment domain family, member 6 [Source:HGNC Symbol;Acc:HG	0.473603682	0.402172303	0.618157746	-1.49393373	9
35	ENSG00000133135	RNF128	ring finger protein 128, E3 ubiquitin protein ligase [Source:HGNC Symbol;Acc:	0.250321204	-0.10089264	1.132761499	-1.28219006	9
36	ENSG00000133216	EPHB2	EPH receptor B2 [Source:HGNC Symbol;Acc:HGNC:3393]	0.083663732	-0.097935345	1.228468523	-1.21419691	9
37	ENSG00000133789	SWAP70	SWAP switching B-cell complex 70kDa subunit [Source:HGNC Symbol;Acc:HGI	0.522060118	0.28895859	0.67038109	-1.4813998	9
38	ENSG00000137198	GMPR	guanosine monophosphate reductase [Source:HGNC Symbol;Acc:HGNC:4376	0.516570981	0.446692023	0.535640058	-1.49890306	9
39	ENSG00000139567	ACVRL1	activin A receptor type II-like 1 [Source:HGNC Symbol;Acc:HGNC:175]	0.54584691	0.231741064	0.694263191	-1.47185116	9
40	ENSG00000139737	SLAIN1	SLAIN motif family, member 1 [Source:HGNC Symbol;Acc:HGNC:26387]	-0.01709981	-0.545059446	1.409043787	-0.84688453	9
41	ENSG00000143603	KCNN3	potassium intermediate/small conductance calcium-activated channel, subfa	0.521395483	-0.486689469	1.098594541	-1.13330055	9
42	ENSG00000144063	MALL	mal, T-cell differentiation protein-like [Source:HGNC Symbol;Acc:HGNC:6818]	-0.31570286	-0.521040682	1.485793532	-0.64904999	9
43	ENSG00000145335	SNCA	synuclein, alpha (non A4 component of amyloid precursor) [Source:HGNC Syn	0.481550935	-0.539404522	1.14146397	-1.08361038	9
44	ENSG00000146374	RSPO3	R-spondin 3 [Source:HGNC Symbol;Acc:HGNC:20866]	-0.09615373	-0.638650409	1.442788407	-0.70798427	9
45	ENSG00000147231	CXorf57	chromosome X open reading frame 57 [Source:HGNC Symbol;Acc:HGNC:2548	0.260550901	-0.765976503	1.305724127	-0.80029852	9
46	ENSG00000147394	ZNF185	zinc finger protein 185 (LIM domain) [Source:HGNC Symbol;Acc:HGNC:12976]	-0.0898357	-0.282318616	1.378338944	-1.00618463	9
47	ENSG00000147573	TRIM55	tripartite motif containing 55 [Source:HGNC Symbol;Acc:HGNC:14215]	-0.13249499	-0.318483078	1.404328083	-0.95335001	9
48	ENSG00000147889	CDKN2A	cyclin-dependent kinase inhibitor 2A [Source:HGNC Symbol;Acc:HGNC:1787]	0.44823583	0.322569861	0.709577205	-1.4803829	9
49	ENSG00000148082	SHC3	SHC (Src homology 2 domain containing) transforming protein 3 [Source:HGN	0.33224567	0.254890306	0.858013407	-1.44514938	9
50	ENSG00000149564	ESAM	endothelial cell adhesion molecule [Source:HGNC Symbol;Acc:HGNC:17474]	-0.12614238	-0.488575956	1.437943602	-0.82322526	9
51	ENSG00000150510	FAM124A	family with sequence similarity 124A [Source:HGNC Symbol;Acc:HGNC:26413	0.680821156	-0.189152286	0.844998922	-1.33666779	9
52	ENSG00000151702	FLI1	Fli-1 proto-oncogene, ETS transcription factor [Source:HGNC Symbol;Acc:HGN	0.439658673	0.309463242	0.727786749	-1.47690866	9
53	ENSG00000152402	GUCY1A2	guanylate cyclase 1, soluble, alpha 2 [Source:HGNC Symbol;Acc:HGNC:4684]	0.268806997	0.187529533	0.952605625	-1.40894215	9
54	ENSG00000152804	HHEX	hematopoietically expressed homeobox [Source:HGNC Symbol;Acc:HGNC:49	0.337167236	0.271975778	0.841469466	-1.45061248	9
55	ENSG00000153993	SEMA3D	sema domain, immunoglobulin domain (Ig), short basic domain, secreted, (se	0.356526935	-0.220883845	1.118538297	-1.25418139	9
56	ENSG00000154146	NRGN	neurogranin (protein kinase C substrate, RC3) [Source:HGNC Symbol;Acc:HGN	-0.03739222	-0.296985629	1.361864004	-1.02748615	9
57	ENSG00000156453	PCDH1	protocadherin 1 [Source:HGNC Symbol;Acc:HGNC:8655]	0.035665796	-0.480408259	1.377585049	-0.93284259	9
58	ENSG00000159263	SIM2	single-minded family bHLH transcription factor 2 [Source:HGNC Symbol;Acc:H	0.439757179	-0.411124251	1.133982398	-1.16261533	9

59	ENSG00000161835	GRASP	GRP1 (general receptor for phosphoinositides 1)-associated scaffold protein [	0.503016798	0.078221479	0.84338627	-1.42462455	9
60	ENSG00000162545	CAMK2N1	calcium/calmodulin-dependent protein kinase II inhibitor 1 [Source:HGNC Syr	0.21543372	0.094761653	1.04833448	-1.35852985	9
61	ENSG00000163710	PCOLCE2	procollagen C-endopeptidase enhancer 2 [Source:HGNC Symbol;Acc:HGNC:87	0.174112226	-0.063796308	1.161297992	-1.27161391	9
62	ENSG00000165092	ALDH1A1	aldehyde dehydrogenase 1 family, member A1 [Source:HGNC Symbol;Acc:HG	0.296697758	-0.759024903	1.286857288	-0.82453014	9
63	ENSG00000166979	EVA1C	eva-1 homolog C (C. elegans) [Source:HGNC Symbol;Acc:HGNC:13239]	0.482164105	0.160141149	0.804812419	-1.44711767	9
64	ENSG00000170379	FAM115C	family with sequence similarity 115, member C [Source:HGNC Symbol;Acc:HG	0.329949763	0.312452985	0.81615645	-1.4585592	9
65	ENSG00000171714	ANO5	anoctamin 5 [Source:HGNC Symbol;Acc:HGNC:27337]	0.439185846	-0.175979009	1.039188677	-1.30239551	9
66	ENSG00000171877	FRMD5	FERM domain containing 5 [Source:HGNC Symbol;Acc:HGNC:28214]	0.077133945	0.077049498	1.14279095	-1.29697439	9
67	ENSG00000172889	EGFL7	EGF-like-domain, multiple 7 [Source:HGNC Symbol;Acc:HGNC:20594]	0.666548194	-0.167905405	0.846853538	-1.34549633	9
68	ENSG00000173269	MMRN2	multimerin 2 [Source:HGNC Symbol;Acc:HGNC:19888]	0.053106446	-0.120407473	1.254391483	-1.18709046	9
69	ENSG00000176435	CLEC14A	C-type lectin domain family 14, member A [Source:HGNC Symbol;Acc:HGNC:1	-0.28295691	-0.474761486	1.476007396	-0.718289	9
70	ENSG00000178401	DNAJC22	DnaJ (Hsp40) homolog, subfamily C, member 22 [Source:HGNC Symbol;Acc:H	0.378044589	0.35415856	0.743761768	-1.47596492	9
71	ENSG00000178462	TUBAL3	tubulin, alpha-like 3 [Source:HGNC Symbol;Acc:HGNC:23534]	0.732695854	-0.593950766	0.955506515	-1.0942516	9
72	ENSG00000179104	TMTC2	transmembrane and tetratricopeptide repeat containing 2 [Source:HGNC Sym	0.235036622	-0.104273577	1.144026457	-1.2747895	9
73	ENSG00000182809	CRIP2	cysteine-rich protein 2 [Source:HGNC Symbol;Acc:HGNC:2361]	0.342986162	0.234962086	0.864312749	-1.442261	9
74	ENSG00000183578	TNFAIP8L3	tumor necrosis factor, alpha-induced protein 8-like 3 [Source:HGNC Symbol;A	0.449126631	-0.169006164	1.028376425	-1.30849689	9
75	ENSG00000183615	FAM167B	family with sequence similarity 167, member B [Source:HGNC Symbol;Acc:HG	0.408875514	0.304526615	0.758111265	-1.47151339	9
76	ENSG00000186564	FOXD2	forkhead box D2 [Source:HGNC Symbol;Acc:HGNC:3803]	-0.21009175	-0.214855455	1.399783277	-0.97483607	9
77	ENSG00000188582	PAQR9	progestin and adipoQ receptor family member IX [Source:HGNC Symbol;Acc:I	-0.15237913	-0.647643724	1.457678653	-0.6576558	9
78	ENSG00000188643	S100A16	S100 calcium binding protein A16 [Source:HGNC Symbol;Acc:HGNC:20441]	0.552532848	0.21913582	0.6978978	-1.46956647	9
79	ENSG00000196781	TLE1	transducin-like enhancer of split 1 (E(sp1) homolog, Drosophila) [Source:HGN	0.314248898	-0.190106056	1.133211306	-1.25735415	9
80	ENSG00000197467	COL13A1	collagen, type XIII, alpha 1 [Source:HGNC Symbol;Acc:HGNC:2190]	0.238177036	0.174590438	0.982649775	-1.39541725	9
81	ENSG00000197555	SIPA1L1	signal-induced proliferation-associated 1 like 1 [Source:HGNC Symbol;Acc:HG	0.376413737	0.309483235	0.781174616	-1.46707159	9
82	ENSG00000197565	COL4A6	collagen, type IV, alpha 6 [Source:HGNC Symbol;Acc:HGNC:2208]	-0.42226391	-0.473829413	1.495826995	-0.59973367	9
83	ENSG00000198435	NRARP	NOTCH-regulated ankyrin repeat protein [Source:HGNC Symbol;Acc:HGNC:33	0.27542991	0.086650927	1.012919111	-1.37499995	9
84	ENSG00000214814	FER1L6	fer-1-like family member 6 [Source:HGNC Symbol;Acc:HGNC:28065]	-0.03841316	-0.265044876	1.35219398	-1.04873595	9
1	ENSG00000061938	TNK2	tyrosine kinase, non-receptor, 2 [Source:HGNC Symbol;Acc:HGNC:19297]	0.600227788	0.342162714	0.548265721	-1.49065622	10
2	ENSG00000010438	PRSS3	protease, serine, 3 [Source:HGNC Symbol;Acc:HGNC:9486]	1.368163113	-0.393159477	0.011617045	-0.98662068	10
3	ENSG00000066056	TIE1	tyrosine kinase with immunoglobulin-like and EGF-like domains 1 [Source:HG	0.981785664	-0.145473236	0.49431987	-1.3306323	10
4	ENSG00000091129	NRCAM	neuronal cell adhesion molecule [Source:HGNC Symbol;Acc:HGNC:7994]	1.338217622	-0.724736455	0.191130432	-0.8046116	10
5	ENSG00000102468	HTR2A	5-hydroxytryptamine (serotonin) receptor 2A, G protein-coupled [Source:HGI	1.309014296	-0.188823919	-0.001787989	-1.11840239	10
6	ENSG00000109511	ANXA10	annexin A10 [Source:HGNC Symbol;Acc:HGNC:534]	0.716393895	0.35395064	0.410523249	-1.48086778	10
7	ENSG00000111339	ART4	ADP-ribosyltransferase 4 (Dombrock blood group) [Source:HGNC Symbol;Acc:	1.293737975	-0.129581831	-0.020018984	-1.14413716	10
8	ENSG00000114771	AADAC	arylacetamide deacetylase [Source:HGNC Symbol;Acc:HGNC:17]	1.322410696	-0.589668542	0.197129335	-0.92987149	10
9	ENSG00000117586	TNFSF4	tumor necrosis factor (ligand) superfamily, member 4 [Source:HGNC Symbol;/	0.84392556	0.033049361	0.536744533	-1.41371945	10
10	ENSG00000120279	MYCT1	myc target 1 [Source:HGNC Symbol;Acc:HGNC:23172]	0.791592437	0.090340479	0.553095204	-1.43502812	10
11	ENSG00000121207	LRAT	lecithin retinol acyltransferase (phosphatidylcholine--retinol O-acyltransfera:	1.486013108	-0.523886461	-0.316041847	-0.6460848	10
12	ENSG00000121898	CPXM2	carboxypeptidase X (M14 family), member 2 [Source:HGNC Symbol;Acc:HGN(	1.361962294	-0.393000765	0.02599249	-0.99495402	10
13	ENSG00000126549	STATH	statherin [Source:HGNC Symbol;Acc:HGNC:11369]	1.498614901	-0.500371347	-0.446506203	-0.55173735	10
14	ENSG00000128052	KDR	kinase insert domain receptor (a type III receptor tyrosine kinase) [Source:HG	1.032139183	0.090614919	0.243666196	-1.3664203	10
15	ENSG00000131409	LRRC4B	leucine rich repeat containing 4B [Source:HGNC Symbol;Acc:HGNC:25042]	1.436592661	-0.395730128	-0.175538686	-0.86532385	10
16	ENSG00000133101	CCNA1	cyclin A1 [Source:HGNC Symbol;Acc:HGNC:1577]	1.209198887	-0.52564589	0.375130783	-1.05868378	10
17	ENSG00000135547	HEY2	hes-related family bHLH transcription factor with YRPW motif 2 [Source:HGN(	1.413790905	-0.250755151	-0.21958484	-0.94345091	10
18	ENSG00000137203	TFAP2A	transcription factor AP-2 alpha (activating enhancer binding protein 2 alpha) [	1.267794716	-0.104210745	0.011871576	-1.17545555	10
19	ENSG00000137727	ARHGAP20	Rho GTPase activating protein 20 [Source:HGNC Symbol;Acc:HGNC:18357]	1.155166473	0.019583188	0.110904675	-1.28565434	10
20	ENSG00000144837	PLA1A	phospholipase A1 member A [Source:HGNC Symbol;Acc:HGNC:17661]	1.403250712	-0.434696018	-0.052491687	-0.91606301	10
21	ENSG00000149557	FEZ1	fasciculation and elongation protein zeta 1 (zygin I) [Source:HGNC Symbol;Acc	0.584342241	0.446812876	0.466069518	-1.49722463	10
22	ENSG00000150551	LYPD1	LY6/PLAUR domain containing 1 [Source:HGNC Symbol;Acc:HGNC:28431]	0.634765156	0.348163996	0.506728835	-1.48965799	10
23	ENSG00000152208	GRID2	glutamate receptor, ionotropic, delta 2 [Source:HGNC Symbol;Acc:HGNC:457(	0.913341658	-0.721830863	0.806028676	-0.99753947	10
24	ENSG00000158352	SHROOM4	shroom family member 4 [Source:HGNC Symbol;Acc:HGNC:29215]	0.570955195	0.433092926	0.493562291	-1.49761041	10
25	ENSG00000159261	CLDN14	claudin 14 [Source:HGNC Symbol;Acc:HGNC:2035]	1.042269224	-0.626752805	0.639156438	-1.05467286	10
26	ENSG00000162817	C1orf115	chromosome 1 open reading frame 115 [Source:HGNC Symbol;Acc:HGNC:258	0.956738894	0.139270589	0.307751064	-1.40376055	10
27	ENSG00000162892	IL24	interleukin 24 [Source:HGNC Symbol;Acc:HGNC:11346]	1.424426039	-0.292047079	-0.216491102	-0.91588786	10
28	ENSG00000164107	HAND2	heart and neural crest derivatives expressed 2 [Source:HGNC Symbol;Acc:HGI	0.531159375	0.450189794	0.517709215	-1.49905838	10
29	ENSG00000165507	C10orf10	chromosome 10 open reading frame 10 [Source:HGNC Symbol;Acc:HGNC:233	0.994637694	-0.045813925	0.408329208	-1.35715298	10
30	ENSG00000165566	AMER2	APC membrane recruitment protein 2 [Source:HGNC Symbol;Acc:HGNC:2636	1.160536436	-0.549153341	0.457424152	-1.06880725	10
31	ENSG00000165716	FAM69B	family with sequence similarity 69, member B [Source:HGNC Symbol;Acc:HGN	0.716450272	0.25492888	0.501711443	-1.4730906	10
32	ENSG00000171388	APLN	apelin [Source:HGNC Symbol;Acc:HGNC:16665]	1.159075015	-0.298520013	0.343399904	-1.20395491	10
33	ENSG00000175746	C15orf54	chromosome 15 open reading frame 54 [Source:HGNC Symbol;Acc:HGNC:337	0.742526846	0.166572247	0.547367862	-1.45646696	10
34	ENSG00000176105	YES1	YES proto-oncogene 1, Src family tyrosine kinase [Source:HGNC Symbol;Acc:H	0.801458001	0.126525288	0.513525601	-1.44150889	10
35	ENSG00000176771	NCKAP5	NCK-associated protein 5 [Source:HGNC Symbol;Acc:HGNC:29847]	1.080788179	-0.230579402	0.416709893	-1.26691867	10
36	ENSG00000177464	GPR4	G protein-coupled receptor 4 [Source:HGNC Symbol;Acc:HGNC:4497]	1.284032882	-0.180106898	0.043643746	-1.14756973	10
37	ENSG00000178773	CPNE7	copine VII [Source:HGNC Symbol;Acc:HGNC:2320]	0.806470395	0.040043007	0.574167379	-1.42068078	10
38	ENSG00000179776	CDH5	cadherin 5, type 2 (vascular endothelium) [Source:HGNC Symbol;Acc:HGNC:1	0.692108273	0.308132361	0.481154345	-1.48139498	10
39	ENSG00000182674	KCNB2	potassium voltage-gated channel, Shab-related subfamily, member 2 [Source	1.392265457	-0.392017159	-0.048642636	-0.95160566	10
40	ENSG00000183072	NKX2-5	NK2 homeobox 5 [Source:HGNC Symbol;Acc:HGNC:2488]	1.16313275	-0.755639646	0.500918481	-0.90841158	10
41	ENSG00000183783	KCTD8	potassium channel tetramerization domain containing 8 [Source:HGNC Symb(	1.189700908	-0.58579786	0.425751428	-1.02965448	10
42	ENSG00000183840	GPR39	G protein-coupled receptor 39 [Source:HGNC Symbol;Acc:HGNC:4496]	0.634357964	0.324949933	0.528280001	-1.4875879	10
43	ENSG00000196104	SPOCK3	sparc/osteonectin, cwcv and kazal-like domains proteoglycan (testican) 3 [Sou	1.107303904	-0.594974402	0.546531569	-1.05886107	10
44	ENSG00000198113	TOR4A	torsin family 4, member A [Source:HGNC Symbol;Acc:HGNC:25981]	0.704193987	0.244411919	0.524307847	-1.47291375	10
45	ENSG00000198523	PLN	phospholamban [Source:HGNC Symbol;Acc:HGNC:9080]	1.434086384	-0.296077493	-0.246357392	-0.8916515	10
46	ENSG00000213145	CRIP1	cysteine-rich protein 1 (intestinal) [Source:HGNC Symbol;Acc:HGNC:2360]	0.794215657	-0.219277459	0.750765441	-1.32570364	10
47	ENSG00000213316	LTC4S	leukotriene C4 synthase [Source:HGNC Symbol;Acc:HGNC:6719]	1.353444868	-0.247410617	-0.055363083	-1.05067117	10
1	ENSG00000126733	DACH2	dachshund family transcription factor 2 [Source:HGNC Symbol;Acc:HGNC:168	-0.50853682	-0.508536816	1.499889737	-0.48281611	11
2	ENSG00000120068	HOXB8	homeobox B8 [Source:HGNC Symbol;Acc:HGNC:5119]	-0.53137851	-0.531378508	1.498474037	-0.43571702	11
3	ENSG00000129152	MYOD1	myogenic differentiation 1 [Source:HGNC Symbol;Acc:HGNC:7611]	-0.58170436	-0.581704365	1.489030141	-0.32562141	11
4	ENSG00000176204	LRRTM4	leucine rich repeat transmembrane neuronal 4 [Source:HGNC Symbol;Acc:HG	-0.52447617	-0.524476169	1.499078394	-0.45012606	11
5	ENSG00000180053	NKX2-6	NK2 homeobox 6 [Source:HGNC Symbol;Acc:HGNC:32940]	-0.53417793	-0.534177927	1.498184091	-0.42982824	11
6	ENSG00000211765	TRBJ2-2	T cell receptor beta joining 2-2 [Source:HGNC Symbol;Acc:HGNC:12169]	-0.58982983	-0.589829827	1.48660268	-0.30694303	11
7	ENSG00000005249	PRKAR2B	protein kinase, cAMP-dependent, regulatory, type II, beta [Source:HGNC Sym	-0.64253725	-0.895485127	1.31787176	0.220150616	11
8	ENSG00000001617	SEMA3F	sema domain, immunoglobulin domain (Ig), short basic domain, secreted, (se	-0.37465288	-1.061160933	1.310693561	0.125120257	11
9	ENSG00000009709	PAX7	paired box 7 [Source:HGNC Symbol;Acc:HGNC:8621]	-0.5207349	-0.56146811	1.49713682	-0.41493381	11
10	ENSG00000016082	ISL1	ISL LIM homeobox 1 [Source:HGNC Symbol;Acc:HGNC:6132]	-0.58435922	-0.597051834	1.486302359	-0.3048913	11
11	ENSG00000026559	KCNG1	potassium voltage-gated channel, subfamily G, member 1 [Source:HGNC Sym	-0.57303626	-1.098106478	1.02189445	0.649248289	11

12	ENSG00000072657	TRHDE	thyrotropin-releasing hormone degrading enzyme [Source:HGNC Symbol;Acc:	-0.58077576	-0.596308967	1.4869718	-0.30988707	11
13	ENSG00000077063	CTTNBP2	cortactin binding protein 2 [Source:HGNC Symbol;Acc:HGNC:15679]	-0.65599452	-0.837378534	1.360440081	0.132932969	11
14	ENSG00000078295	ADCY2	adenylate cyclase 2 (brain) [Source:HGNC Symbol;Acc:HGNC:233]	-0.46189583	-1.118471568	1.168927039	0.41144036	11
15	ENSG00000081059	TCF7	transcription factor 7 (T-cell specific, HMG-box) [Source:HGNC Symbol;Acc:HG	-0.82399779	-0.883011541	1.058904072	0.64810526	11
16	ENSG00000091409	ITGA6	integrin, alpha 6 [Source:HGNC Symbol;Acc:HGNC:6142]	0.108939662	-1.429138542	0.852755396	0.467443485	11
17	ENSG00000101096	NFATC2	nuclear factor of activated T-cells, cytoplasmic, calcineurin-dependent 2 [Sour	-0.59295012	-1.053679501	1.125370366	0.521259251	11
18	ENSG00000101542	CDH20	cadherin 20, type 2 [Source:HGNC Symbol;Acc:HGNC:1760]	-0.61849368	-1.031809046	1.134251766	0.51605096	11
19	ENSG00000102452	NALCN	sodium leak channel, non-selective [Source:HGNC Symbol;Acc:HGNC:19082]	-0.7169482	-0.775480634	1.367077986	0.125350853	11
20	ENSG00000102466	FGF14	fibroblast growth factor 14 [Source:HGNC Symbol;Acc:HGNC:3671]	-0.62908324	-0.766865272	1.419720681	-0.02377216	11
21	ENSG00000103241	FOXF1	forkhead box F1 [Source:HGNC Symbol;Acc:HGNC:3809]	-0.62476077	-0.817050976	1.392728439	0.049083311	11
22	ENSG00000103522	IL21R	interleukin 21 receptor [Source:HGNC Symbol;Acc:HGNC:6006]	-0.66527784	-0.965105511	1.20048495	0.429898399	11
23	ENSG00000104332	SFRP1	secreted frizzled-related protein 1 [Source:HGNC Symbol;Acc:HGNC:10776]	-0.64386957	-0.713871671	1.438504934	-0.0807637	11
24	ENSG00000104783	KCNN4	potassium intermediate/small conductance calcium-activated channel, subfai	-0.83622415	-0.890298767	0.957220413	0.769302509	11
25	ENSG00000105784	RUNDC3B	RUN domain containing 3B [Source:HGNC Symbol;Acc:HGNC:30286]	0.136042437	-1.405975944	0.94989416	0.320039347	11
26	ENSG00000105991	HOXA1	homeobox A1 [Source:HGNC Symbol;Acc:HGNC:5099]	-0.78565739	-0.808704479	1.275586955	0.318774917	11
27	ENSG00000105996	HOXA2	homeobox A2 [Source:HGNC Symbol;Acc:HGNC:5103]	-0.53599132	-0.554640065	1.49672236	-0.40609097	11
28	ENSG00000105997	HOXA3	homeobox A3 [Source:HGNC Symbol;Acc:HGNC:5104]	-0.57229898	-0.587590186	1.489488483	-0.32959932	11
29	ENSG00000106004	HOXA5	homeobox A5 [Source:HGNC Symbol;Acc:HGNC:5106]	-0.83495279	-0.841205186	1.146684643	0.52947333	11
30	ENSG00000108511	HOXB6	homeobox B6 [Source:HGNC Symbol;Acc:HGNC:5117]	-0.56511486	-0.575682525	1.491953219	-0.35115584	11
31	ENSG00000114279	FGF12	fibroblast growth factor 12 [Source:HGNC Symbol;Acc:HGNC:3668]	-0.72761832	-0.772845167	1.361618113	0.138845377	11
32	ENSG00000120075	HOXB5	homeobox B5 [Source:HGNC Symbol;Acc:HGNC:5116]	-0.50906875	-0.523288906	1.499575127	-0.46721747	11
33	ENSG00000123689	G0S2	G0/G1 switch 2 [Source:HGNC Symbol;Acc:HGNC:30229]	-0.5372863	-1.136663245	0.932542998	0.741406542	11
34	ENSG00000129682	FGF13	fibroblast growth factor 13 [Source:HGNC Symbol;Acc:HGNC:3670]	-0.73432532	-0.983832874	0.950745374	0.767412819	11
35	ENSG00000130720	FIBCD1	fibrinogen C domain containing 1 [Source:HGNC Symbol;Acc:HGNC:25922]	-0.48972631	-1.024045515	1.28874414	0.225027689	11
36	ENSG00000135549	PKIB	protein kinase (cAMP-dependent, catalytic) inhibitor beta [Source:HGNC Sym	-0.6504801	-1.021703254	1.095802585	0.576380771	11
37	ENSG00000138741	TRPC3	transient receptor potential cation channel, subfamily C, member 3 [Source:H	-0.72898829	-0.9658741	1.07034825	0.624514144	11
38	ENSG00000139874	SSTR1	somatostatin receptor 1 [Source:HGNC Symbol;Acc:HGNC:11330]	-0.73099107	-0.762233874	1.367012022	0.126212927	11
39	ENSG00000139970	RTN1	reticulon 1 [Source:HGNC Symbol;Acc:HGNC:10467]	-0.42276311	-0.743220868	1.474377085	-0.30839311	11
40	ENSG00000139973	SYT16	synaptotagmin XVI [Source:HGNC Symbol;Acc:HGNC:23142]	-0.57618455	-0.683073836	1.468841817	-0.20958343	11
41	ENSG00000145536	ADAMTS16	ADAM metallopeptidase with thrombospondin type 1 motif, 16 [Source:HGN	-0.65432544	-0.841404309	1.35831277	0.137416979	11
42	ENSG00000149256	TENM4	teneurin transmembrane protein 4 [Source:HGNC Symbol;Acc:HGNC:29945]	-0.79320602	-0.919159113	1.02920507	0.683160059	11
43	ENSG00000149571	KIRREL3	kin of IRRE like 3 (Drosophila) [Source:HGNC Symbol;Acc:HGNC:23204]	-0.72741884	-0.761615103	1.369894145	0.119139803	11
44	ENSG00000151136	BTBD11	BTB (POZ) domain containing 11 [Source:HGNC Symbol;Acc:HGNC:23844]	-0.29530854	-1.279624386	0.920032349	0.654900581	11
45	ENSG00000156395	SORCS3	sortilin-related VPS10 domain containing receptor 3 [Source:HGNC Symbol;Ac	-0.487873	-0.526077546	1.499742742	-0.4857922	11
46	ENSG00000159556	ISL2	ISL LIM homeobox 2 [Source:HGNC Symbol;Acc:HGNC:18524]	-0.63565053	-0.717029532	1.440193872	-0.08751381	11
47	ENSG00000162490	DRAXIN	dorsal inhibitory axon guidance protein [Source:HGNC Symbol;Acc:HGNC:250	-0.67749241	-0.686830955	1.436674159	-0.0723508	11
48	ENSG00000162670	BRINP3	bone morphogenetic protein/retinoic acid inducible neural-specific 3 [Source	-0.58040199	-0.976181318	1.277655137	0.278928166	11
49	ENSG00000162981	FAM84A	family with sequence similarity 84, member A [Source:HGNC Symbol;Acc:HGN	-0.85329546	-0.876719884	0.923199435	0.806815907	11
50	ENSG00000163017	ACTG2	actin, gamma 2, smooth muscle, enteric [Source:HGNC Symbol;Acc:HGNC:14	-0.76494626	-0.961518162	0.863654688	0.86280973	11
51	ENSG00000163064	EN1	engrailed homeobox 1 [Source:HGNC Symbol;Acc:HGNC:3342]	-0.77934538	-0.883389188	1.170410946	0.492323617	11
52	ENSG00000163623	NKX6-1	NK6 homeobox 1 [Source:HGNC Symbol;Acc:HGNC:7839]	-0.82353995	-0.870783924	1.100292941	0.594030934	11
53	ENSG00000164100	NDST3	N-deacetylase/N-sulfotransferase (heparan glucosaminyl) 3 [Source:HGNC Sy	-0.6222527	-0.795649559	1.406991226	0.010911031	11
54	ENSG00000164283	ESM1	endothelial cell-specific molecule 1 [Source:HGNC Symbol;Acc:HGNC:3466]	0.15298961	-1.445378461	0.807967126	0.484421724	11
55	ENSG00000165078	CPA6	carboxypeptidase A6 [Source:HGNC Symbol;Acc:HGNC:17245]	-0.54304479	-0.587340009	1.492890942	-0.36250614	11
56	ENSG00000165449	SLC16A9	solute carrier family 16, member 9 [Source:HGNC Symbol;Acc:HGNC:23520]	-0.34970703	-1.258673695	0.804978488	0.803402238	11
57	ENSG00000166342	NETO1	neuropilin (NRP) and tolloid (TLL)-like 1 [Source:HGNC Symbol;Acc:HGNC:138	-0.75767324	-0.766751552	1.343627845	0.180796945	11
58	ENSG00000166446	CDYL2	chromodomain protein, Y-like 2 [Source:HGNC Symbol;Acc:HGNC:23030]	-0.20423485	-1.237968422	1.160275387	0.281927889	11
59	ENSG00000170323	FABP4	fatty acid binding protein 4, adipocyte [Source:HGNC Symbol;Acc:HGNC:3559	-0.77025112	-0.797797376	1.304045652	0.264002842	11
60	ENSG00000174473	GALNTL6	polypeptide N-acetylgalactosaminyltransferase-like 6 [Source:HGNC Symbol;A	-0.58930441	-0.911100371	1.340545164	0.159859612	11
61	ENSG00000175161	CADM2	cell adhesion molecule 2 [Source:HGNC Symbol;Acc:HGNC:29849]	-0.61836589	-1.077391974	0.945482201	0.750275662	11
62	ENSG00000175928	LRRN1	leucine rich repeat neuronal 1 [Source:HGNC Symbol;Acc:HGNC:20980]	-0.55928943	-0.666176861	1.476596534	-0.25113024	11
63	ENSG00000176194	CIDEA	cell death-inducing DFFA-like effector a [Source:HGNC Symbol;Acc:HGNC:197	-0.7356411	-0.838261189	1.295658396	0.278243896	11
64	ENSG00000179242	CDH4	cadherin 4, type 1, R-cadherin (retinal) [Source:HGNC Symbol;Acc:HGNC:176	-0.82495961	-0.905303212	0.903621877	0.826640941	11
65	ENSG00000180287	PLD5	phospholipase D family, member 5 [Source:HGNC Symbol;Acc:HGNC:26879]	-0.56418732	-0.577179291	1.491877602	-0.35051099	11
66	ENSG00000182742	HOXB4	homeobox B4 [Source:HGNC Symbol;Acc:HGNC:5115]	-0.54993377	-0.579499505	1.49314095	-0.36370767	11
67	ENSG00000183032	SLC25A21	solute carrier family 25 (mitochondrial oxoadipate carrier), member 21 [Sourc	-0.56270042	-0.96127372	1.308829135	0.215145001	11
68	ENSG00000183691	NOG	noggin [Source:HGNC Symbol;Acc:HGNC:7866]	-0.70686904	-0.86959351	1.288993793	0.287468759	11
69	ENSG00000185052	SLC24A3	solute carrier family 24 (sodium/potassium/calcium exchanger), member 3 [S	-0.4893182	-0.556288463	1.498627137	-0.45302048	11
70	ENSG00000185985	SLITRK2	SLIT and NTRK-like family, member 2 [Source:HGNC Symbol;Acc:HGNC:13449	-0.60230403	-0.628938107	1.476924765	-0.24568263	11
71	ENSG00000186684	CYP27C1	cytochrome P450, family 27, subfamily C, polypeptide 1 [Source:HGNC Symbo	-0.79030818	-0.834216434	1.236500331	0.388024287	11
72	ENSG00000189056	RELN	reelin [Source:HGNC Symbol;Acc:HGNC:9957]	-0.5648571	-0.69355341	1.468288571	-0.20987807	11
73	ENSG00000189320	FAM180A	family with sequence similarity 180, member A [Source:HGNC Symbol;Acc:HG	0.328853076	-1.488939585	0.611778188	0.548308321	11
74	ENSG00000197046	SIGLEC15	sialic acid binding Ig-like lectin 15 [Source:HGNC Symbol;Acc:HGNC:27596]	-0.81897436	-0.83039744	1.198522373	0.450849429	11
75	ENSG00000197576	HOXA4	homeobox A4 [Source:HGNC Symbol;Acc:HGNC:5105]	-0.61105138	-0.617619214	1.477569623	-0.24889903	11
76	ENSG00000197632	SERPINB2	serpin peptidase inhibitor, clade B (ovalbumin), member 2 [Source:HGNC Sym	-0.57953025	-0.631945656	1.480652932	-0.26917702	11
77	ENSG00000198542	ITGBL1	integrin, beta-like 1 (with EGF-like repeat domains) [Source:HGNC Symbol;Ac	-0.60501653	-0.801611668	1.411153166	-0.00452497	11
78	ENSG00000203805	PPAPDC1A	phosphatidic acid phosphatase type 2 domain containing 1A [Source:HGNC Sy	-0.44979421	-0.686577032	1.484871867	-0.34850062	11
79	ENSG00000206432	TMEM200C	transmembrane protein 200C [Source:HGNC Symbol;Acc:HGNC:37208]	-0.45815457	-0.917774577	1.395493268	-0.01956412	11
80	ENSG00000211767	TRBJ2-3	T cell receptor beta joining 2-3 [Source:HGNC Symbol;Acc:HGNC:12171]	-0.60972751	-0.616458496	1.478091622	-0.25190561	11
81	ENSG00000242550	SERPINB10	serpin peptidase inhibitor, clade B (ovalbumin), member 10 [Source:HGNC Sy	-0.582319	-0.626167261	1.481340509	-0.27285425	11
82	ENSG00000260027	HOXB7	homeobox B7 [Source:HGNC Symbol;Acc:HGNC:5118]	-0.60696103	-0.637992714	1.473831292	-0.22887755	11
83	ENSG00000186960	C14orf23	long intergenic non-protein coding RNA 1551 [Source:HGNC Symbol;Acc:HGN	-0.5	-0.5	1.5	-0.5	11
84	ENSG00000008118	CAMK1G	calcium/calmodulin-dependent protein kinase IG [Source:HGNC Symbol;Acc:H	-0.88129164	-0.850620704	0.868109165	0.863803181	11
85	ENSG00000005020	SKAP2	src kinase associated phosphoprotein 2 [Source:HGNC Symbol;Acc:HGNC:156	-0.74362937	-0.607153911	1.438959502	-0.08817622	11
86	ENSG00000114948	ADAM23	ADAM metallopeptidase domain 23 [Source:HGNC Symbol;Acc:HGNC:202]	-0.59222397	-0.456948504	1.496719817	-0.44754735	11
87	ENSG00000117600	LPPR4	Lipid phosphate phosphatase-related protein type 4 [Source:UniProtKB/Swis:	-0.88626533	-0.845444123	0.87906804	0.852641413	11
88	ENSG00000120093	HOXB3	homeobox B3 [Source:HGNC Symbol;Acc:HGNC:5114]	-0.54565001	-0.491651558	1.499096484	-0.46179491	11
89	ENSG00000131634	TMEM204	transmembrane protein 204 [Source:HGNC Symbol;Acc:HGNC:14158]	-1.08664622	-0.59531349	0.999579357	0.682380355	11
90	ENSG00000137672	TRPC6	transient receptor potential cation channel, subfamily C, member 6 [Source:H	-0.85695434	-0.85310612	1.049562756	0.660497707	11
91	ENSG00000138435	CHRNA1	cholinergic receptor, nicotinic, alpha 1 (muscle) [Source:HGNC Symbol;Acc:HG	-0.89751368	-0.828457107	0.959526699	0.766444085	11
92	ENSG00000143127	ITGA10	integrin, alpha 10 [Source:HGNC Symbol;Acc:HGNC:6135]	-0.90140043	-0.76137567	1.167075527	0.495700576	11
93	ENSG00000143195	ILDR2	immunoglobulin-like domain containing receptor 2 [Source:HGNC Symbol;Acc	-0.86867214	-0.659436528	1.331061445	0.197047222	11
94	ENSG00000143995	MEIS1	Meis homeobox 1 [Source:HGNC Symbol;Acc:HGNC:7000]	-0.82798141	-0.385446664	1.452194317	-0.23876624	11
95	ENSG00000144355	DLX1	distal-less homeobox 1 [Source:HGNC Symbol;Acc:HGNC:2914]	-0.87817541	-0.842786946	0.997707995	0.723254361	11

96	ENSG00000156475	PPP2R2B	protein phosphatase 2, regulatory subunit B, beta [Source:HGNC Symbol;Acc:	-0.58780748	-0.491357085	1.496335255	-0.41717069	11
97	ENSG00000158164	TMSB15A	thymosin beta 15a [Source:HGNC Symbol;Acc:HGNC:30744]	-1.02005436	-0.680308073	1.009952642	0.690409794	11
98	ENSG00000162576	MXRA8	matrix-remodelling associated 8 [Source:HGNC Symbol;Acc:HGNC:7542]	-0.88473463	-0.451446972	1.416672671	-0.08049107	11
99	ENSG00000164099	PRSS12	protease, serine, 12 (neurotrypsin, motopsin) [Source:HGNC Symbol;Acc:HGN	-0.76975699	-0.553726315	1.444383752	-0.12090045	11
100	ENSG00000165300	SLITRK5	SLIT and NTRK-like family, member 5 [Source:HGNC Symbol;Acc:HGNC:20295	-0.64422758	-0.643936585	1.462810682	-0.17464651	11
101	ENSG00000167371	PRRT2	proline-rich transmembrane protein 2 [Source:HGNC Symbol;Acc:HGNC:3050	-0.87767508	-0.771023429	1.195904057	0.452794451	11
102	ENSG00000168772	CXXC4	CXXC finger protein 4 [Source:HGNC Symbol;Acc:HGNC:24593]	-0.68273783	-0.600710381	1.463124265	-0.17967606	11
103	ENSG00000170549	IRX1	iroquois homeobox 1 [Source:HGNC Symbol;Acc:HGNC:14358]	-0.5648871	-0.51369733	1.497244571	-0.41866014	11
104	ENSG00000170561	IRX2	iroquois homeobox 2 [Source:HGNC Symbol;Acc:HGNC:14359]	-0.95735392	-0.692414824	1.173572432	0.476196314	11
105	ENSG00000171724	VAT1L	vesicle amine transport 1-like [Source:HGNC Symbol;Acc:HGNC:29315]	-0.54641879	-0.539575652	1.497091728	-0.41109728	11
106	ENSG00000171873	ADRA1D	adrenoceptor alpha 1D [Source:HGNC Symbol;Acc:HGNC:280]	-0.78084581	-0.751252412	1.337117039	0.194981182	11
107	ENSG00000176399	DMRTA1	DMRT-like family A1 [Source:HGNC Symbol;Acc:HGNC:13826]	-0.7700747	-0.707485857	1.377081192	0.100479369	11
108	ENSG00000179455	MKRN3	makorin ring finger protein 3 [Source:HGNC Symbol;Acc:HGNC:7114]	-0.93679215	-0.775076358	1.023907797	0.687960708	11
109	ENSG00000180353	HCLS1	hematopoietic cell-specific Lyn substrate 1 [Source:HGNC Symbol;Acc:HGNC:4	-0.8307674	-0.755048989	1.283988502	0.301827889	11
110	ENSG00000184058	TBX1	T-box 1 [Source:HGNC Symbol;Acc:HGNC:11592]	-0.54679676	-0.532233137	1.497530719	-0.41850082	11
111	ENSG00000184524	CEND1	cell cycle exit and neuronal differentiation 1 [Source:HGNC Symbol;Acc:HGNC	-0.71622249	-0.503096458	1.472952573	-0.25363362	11
112	ENSG00000184845	DRD1	dopamine receptor D1 [Source:HGNC Symbol;Acc:HGNC:3020]	-0.5492522	-0.542503788	1.49667926	-0.40492328	11
113	ENSG00000186493	C5orf38	chromosome 5 open reading frame 38 [Source:HGNC Symbol;Acc:HGNC:2422	-0.86934932	-0.536667015	1.398629943	0.007386391	11
114	ENSG00000187323	DCC	DCC netrin 1 receptor [Source:HGNC Symbol;Acc:HGNC:2701]	-0.71504668	-0.708024757	1.409691514	0.013379924	11
115	ENSG00000197442	MAP3K5	mitogen-activated protein kinase kinase kinase 5 [Source:HGNC Symbol;Acc:H	-0.95637472	-0.725700651	1.109381859	0.57269351	11
116	ENSG00000211764	TRBJ2-1	T cell receptor beta joining 2-1 [Source:HGNC Symbol;Acc:HGNC:12168]	-0.55811042	-0.535533088	1.496476694	-0.40283318	11
117	ENSG00000211766	TRBJ2-2P	T cell receptor beta joining 2-2P (non-functional) [Source:HGNC Symbol;Acc:H	-0.60346019	-0.60123454	1.482321265	-0.27762653	11
118	ENSG00000211768	TRBJ2-4	T cell receptor beta joining 2-4 [Source:HGNC Symbol;Acc:HGNC:12172]	-0.56724363	-0.559815557	1.493505403	-0.36644624	11
119	ENSG00000211772	TRBC2	T cell receptor beta constant 2 [Source:HGNC Symbol;Acc:HGNC:12157]	-0.58744267	-0.564332215	1.490531518	-0.33875663	11
120	ENSG00000142698	C1orf94	chromosome 1 open reading frame 94 [Source:HGNC Symbol;Acc:HGNC:2825	-0.58048686	-0.370219781	1.493697752	-0.54299111	11
121	ENSG00000131620	ANO1	anoctamin 1, calcium activated chloride channel [Source:HGNC Symbol;Acc:H	-0.5298694	-0.47965934	1.499649086	-0.49012034	11
122	ENSG00000155816	FMN2	formin 2 [Source:HGNC Symbol;Acc:HGNC:14074]	-0.72378353	-0.337672166	1.479087171	-0.41763147	11
1	ENSG00000104435	STMN2	stathmin 2 [Source:HGNC Symbol;Acc:HGNC:10577]	-0.51798823	-0.517988229	-0.463529162	1.49950562	12
2	ENSG00000002933	TMEM176A	transmembrane protein 176A [Source:HGNC Symbol;Acc:HGNC:24930]	-0.51214643	-0.512146428	-0.475483094	1.49977595	12
3	ENSG00000104879	CKM	creatine kinase, muscle [Source:HGNC Symbol;Acc:HGNC:1994]	-0.52697783	-0.526977829	-0.44492172	1.498877379	12
4	ENSG00000105880	DLX5	distal-less homeobox 5 [Source:HGNC Symbol;Acc:HGNC:2918]	-0.54362368	-0.54362368	-0.409763223	1.497010584	12
5	ENSG00000106006	HOXA6	homeobox A6 [Source:HGNC Symbol;Acc:HGNC:5107]	-0.5173276	-0.517327596	-0.464886392	1.499541583	12
6	ENSG00000117501	MROH9	maestro heat-like repeat family member 9 [Source:HGNC Symbol;Acc:HGNC:2	-0.60039173	-0.600391735	-0.282251754	1.483035224	12
7	ENSG00000118729	CASQ2	calsequestrin 2 (cardiac muscle) [Source:HGNC Symbol;Acc:HGNC:1513]	-0.58936531	-0.589365311	-0.308018141	1.486748763	12
8	ENSG00000122180	MYOG	myogenin (myogenic factor 4) [Source:HGNC Symbol;Acc:HGNC:7612]	-0.58381423	-0.583814228	-0.320797231	1.488425688	12
9	ENSG00000122592	HOXA7	homeobox A7 [Source:HGNC Symbol;Acc:HGNC:5108]	-0.51443642	-0.514436424	-0.470809907	1.499682754	12
10	ENSG00000124491	F13A1	coagulation factor XIII, A1 polypeptide [Source:HGNC Symbol;Acc:HGNC:3531	-0.515834	-0.515834001	-0.4679498	1.499617802	12
11	ENSG00000124785	NRN1	neuritin 1 [Source:HGNC Symbol;Acc:HGNC:17972]	-0.50921283	-0.509212833	-0.481445827	1.499871493	12
12	ENSG00000125726	CD70	CD70 molecule [Source:HGNC Symbol;Acc:HGNC:11937]	-0.557148	-0.557148003	-0.38049383	1.494789835	12
13	ENSG00000125813	PAX1	paired box 1 [Source:HGNC Symbol;Acc:HGNC:8615]	-0.51225184	-0.512251842	-0.475268335	1.499772019	12
14	ENSG00000125869	LAMP5	lysosomal-associated membrane protein family, member 5 [Source:HGNC Syr	-0.60260213	-0.602602125	-0.277023566	1.482227817	12
15	ENSG00000128709	HOXD9	homeobox D9 [Source:HGNC Symbol;Acc:HGNC:5140]	-0.51215056	-0.512150557	-0.475474682	1.499775797	12
16	ENSG00000133937	GSC	goosecoid homeobox [Source:HGNC Symbol;Acc:HGNC:4612]	-0.85989442	-0.859894422	0.714418768	1.005370076	12
17	ENSG00000136944	LMX1B	LIM homeobox transcription factor 1, beta [Source:HGNC Symbol;Acc:HGNC:6	-0.50216191	-0.502161912	-0.49566915	1.499992974	12
18	ENSG00000145428	RNF175	ring finger protein 175 [Source:HGNC Symbol;Acc:HGNC:27735]	-0.57732923	-0.577329234	-0.335568303	1.49022677	12
19	ENSG00000147655	RSPO2	R-spondin 2 [Source:HGNC Symbol;Acc:HGNC:28583]	-0.6651094	-0.665109403	-0.119275047	1.449493852	12
20	ENSG00000151952	TMEM132D	transmembrane protein 132D [Source:HGNC Symbol;Acc:HGNC:29411]	-0.56869521	-0.56869521	-0.354977631	1.492368051	12
21	ENSG00000155511	GRIA1	glutamate receptor, ionotropic, AMPA 1 [Source:HGNC Symbol;Acc:HGNC:457	-0.57716585	-0.577165853	-0.335938279	1.490269985	12
22	ENSG00000156076	WIF1	WNT inhibitory factor 1 [Source:HGNC Symbol;Acc:HGNC:18081]	-0.50227135	-0.502271347	-0.495449549	1.499992244	12
23	ENSG00000164749	HNF4G	hepatocyte nuclear factor 4, gamma [Source:HGNC Symbol;Acc:HGNC:5026]	-0.7235487	-0.723548697	0.050529702	1.396567691	12
24	ENSG00000164778	EN2	engrailed homeobox 2 [Source:HGNC Symbol;Acc:HGNC:3343]	-0.59067521	-0.590675211	-0.304984095	1.486334516	12
25	ENSG00000166415	WDR72	WD repeat domain 72 [Source:HGNC Symbol;Acc:HGNC:26790]	-0.53792775	-0.537927752	-0.421899038	1.497754542	12
26	ENSG00000168530	MYL1	myosin, light chain 1, alkali; skeletal, fast [Source:HGNC Symbol;Acc:HGNC:75	-0.52748253	-0.527482534	-0.443869282	1.498834351	12
27	ENSG00000171435	KSR2	kinase suppressor of ras 2 [Source:HGNC Symbol;Acc:HGNC:18610]	-0.53533321	-0.535333213	-0.427390372	1.498056799	12
28	ENSG00000173702	MUC13	mucin 13, cell surface associated [Source:HGNC Symbol;Acc:HGNC:7511]	-0.541895	-0.541894997	-0.413458145	1.497248138	12
29	ENSG00000175879	HOXD8	homeobox D8 [Source:HGNC Symbol;Acc:HGNC:5139]	-0.51653963	-0.516539632	-0.466503407	1.499582671	12
30	ENSG00000180806	HOXC9	homeobox C9 [Source:HGNC Symbol;Acc:HGNC:5130]	-0.50310746	-0.503107464	-0.493770543	1.49998547	12
31	ENSG00000182107	TMEM30B	transmembrane protein 30B [Source:HGNC Symbol;Acc:HGNC:27254]	-0.66443796	-0.664437962	-0.12108377	1.449959693	12
32	ENSG00000184809	C21orf88	B3GALT5 antisense RNA 1 [Source:HGNC Symbol;Acc:HGNC:16424]	-0.72816683	-0.728166825	0.065184058	1.391149592	12
33	ENSG00000197410	DCHS2	dachsous cadherin-related 2 [Source:HGNC Symbol;Acc:HGNC:23111]	-0.54401509	-0.544015093	-0.408925177	1.496955363	12
34	ENSG00000198597	ZNF536	zinc finger protein 536 [Source:HGNC Symbol;Acc:HGNC:29025]	-0.52351194	-0.523511939	-0.45212657	1.499150448	12
35	ENSG00000204065	TCEAL5	transcription elongation factor A (SII)-like 5 [Source:HGNC Symbol;Acc:HGNC:	-0.53734163	-0.537341632	-0.423141548	1.497824813	12
36	ENSG00000214216	IQCJ	IQ motif containing J [Source:HGNC Symbol;Acc:HGNC:32406]	-0.5635396	-0.563539597	-0.366431503	1.493510696	12
37	ENSG00000215018	COL28A1	collagen, type XXVIII, alpha 1 [Source:HGNC Symbol;Acc:HGNC:22442]	-0.57151718	-0.571517183	-0.348665526	1.491699893	12
38	ENSG00000257184	HOXA10-HOXA9	Uncharacterized protein {ECO:0000313|Ensembl:ENSP00000421799} [Source	-0.50450371	-0.50450371	-0.490962017	1.499969437	12
39	ENSG00000262406	MMP12	matrix metallopeptidase 12 (macrophage elastase) [Source:HGNC Symbol;Acc	-0.50696104	-0.506961038	-0.486004727	1.499926804	12
40	ENSG00000005073	HOXA11	homeobox A11 [Source:HGNC Symbol;Acc:HGNC:5101]	-0.5041473	-0.505883345	-0.489931057	1.4999617	12
41	ENSG00000002746	HECW1	HECT, C2 and WW domain containing E3 ubiquitin protein ligase 1 [Source:HG	-0.5422896	-0.569926504	-0.382665998	1.494882103	12
42	ENSG00000005379	BZRAP1	benzodiazepine receptor (peripheral) associated protein 1 [Source:HGNC Sym	-0.62464647	-0.848566356	0.102345467	1.370867363	12
43	ENSG00000005471	ABCB4	ATP-binding cassette, sub-family B (MDR/TAP), member 4 [Source:HGNC Sym	-0.41578858	-0.865149318	-0.1527005	1.433638402	12
44	ENSG00000009694	TENM1	teneurin transmembrane protein 1 [Source:HGNC Symbol;Acc:HGNC:8117]	-0.48530566	-0.589626494	-0.421460259	1.496392416	12
45	ENSG00000011422	PLAUR	plasminogen activator, urokinase receptor [Source:HGNC Symbol;Acc:HGNC:9	-0.30215179	-1.047836085	0.0043561	1.345631771	12
46	ENSG00000011677	GABRA3	gamma-aminobutyric acid (GABA) A receptor, alpha 3 [Source:HGNC Symbol;A	-0.50679423	-0.516244687	-0.476748396	1.499787317	12
47	ENSG00000023171	GRAMD1B	GRAM domain containing 1B [Source:HGNC Symbol;Acc:HGNC:29214]	-0.79574738	-0.826466868	0.382184587	1.240029662	12
48	ENSG00000028277	POU2F2	POU class 2 homeobox 2 [Source:HGNC Symbol;Acc:HGNC:9213]	-0.51108965	-0.556662842	-0.430194852	1.497947343	12
49	ENSG00000037965	HOXC8	homeobox C8 [Source:HGNC Symbol;Acc:HGNC:5129]	-0.50306524	-0.504393411	-0.492520189	1.499978839	12
50	ENSG00000049130	KITLG	KIT ligand [Source:HGNC Symbol;Acc:HGNC:6343]	-0.60551847	-0.829732256	0.041267678	1.393983049	12
51	ENSG00000050438	SLC4A8	solute carrier family 4, sodium bicarbonate cotransporter, member 8 [Source	-0.7297827	-0.896738898	0.400653064	1.225868539	12
52	ENSG00000050628	PTGER3	prostaglandin E receptor 3 (subtype EP3) [Source:HGNC Symbol;Acc:HGNC:95	-0.65504481	-0.70537341	-0.077417377	1.437835596	12
53	ENSG00000054356	PTPRN	protein tyrosine phosphatase, receptor type, N [Source:HGNC Symbol;Acc:HG	-0.60427794	-0.618876295	-0.255540854	1.478695091	12
54	ENSG00000057657	PRDM1	PR domain containing 1, with ZNF domain [Source:HGNC Symbol;Acc:HGNC:9	-0.67624987	-0.811551512	0.120485183	1.367316197	12
55	ENSG00000060656	PTPRU	protein tyrosine phosphatase, receptor type, U [Source:HGNC Symbol;Acc:HG	-0.59942307	-0.743066832	-0.099275689	1.441765587	12
56	ENSG00000060718	COL11A1	collagen, type XI, alpha 1 [Source:HGNC Symbol;Acc:HGNC:2186]	-0.50603218	-0.58074134	-0.409538366	1.496311885	12
57	ENSG00000064300	NGFR	nerve growth factor receptor [Source:HGNC Symbol;Acc:HGNC:7809]	-0.68609845	-0.697829014	-0.044472277	1.428399736	12

58	ENSG00000065618	COL17A1	collagen, type XVII, alpha 1 [Source:HGNC Symbol;Acc:HGNC:2194]	-0.57235838	-0.727864819	-0.155285354	1.455508553	12
59	ENSG00000069431	ABCC9	ATP-binding cassette, sub-family C (CFTR/MRP), member 9 [Source:HGNC Syn	-0.50541275	-0.812726455	-0.120445693	1.438584895	12
60	ENSG00000072041	SLC6A15	solute carrier family 6 (neutral amino acid transporter), member 15 [Source:H	-0.6281356	-1.027490146	0.528512549	1.127113196	12
61	ENSG00000072133	RPS6KA6	ribosomal protein S6 kinase, 90kDa, polypeptide 6 [Source:HGNC Symbol;Acc	-0.62979624	-0.652019359	-0.182712815	1.464528415	12
62	ENSG00000074317	SNCB	synuclein, beta [Source:HGNC Symbol;Acc:HGNC:11140]	-0.53325789	-0.605482137	-0.352764189	1.491504217	12
63	ENSG00000078399	HOXA9	homeobox A9 [Source:HGNC Symbol;Acc:HGNC:5109]	-0.50376859	-0.504987904	-0.491214438	1.499970933	12
64	ENSG00000078725	BRINP1	bone morphogenetic protein/retinoic acid inducible neural-specific 1 [Source	-0.50032766	-0.500376859	-0.499295295	1.499999814	12
65	ENSG00000082397	EPB41L3	erythrocyte membrane protein band 4.1-like 3 [Source:HGNC Symbol;Acc:HG	-0.59056869	-0.72415067	-0.137181358	1.45190072	12
66	ENSG00000082482	KCNK2	potassium channel, subfamily K, member 2 [Source:HGNC Symbol;Acc:HGNC:	-0.59082591	-0.648951655	-0.234880866	1.474658427	12
67	ENSG00000084070	SMAP2	small ArfGAP2 [Source:HGNC Symbol;Acc:HGNC:25082]	-0.42795899	-0.832321561	-0.185316163	1.445596709	12
68	ENSG00000087494	PTHLH	parathyroid hormone-like hormone [Source:HGNC Symbol;Acc:HGNC:9607]	-0.49814245	-0.806277193	-0.138680783	1.443100423	12
69	ENSG00000088882	CPXM1	carboxypeptidase X (M14 family), member 1 [Source:HGNC Symbol;Acc:HGN(	-0.62327785	-0.777765795	-0.015419327	1.416462968	12
70	ENSG00000089472	HEPH	hephaestin [Source:HGNC Symbol;Acc:HGNC:4866]	-0.57755877	-1.02874457	0.404400325	1.201903014	12
71	ENSG00000092607	TBX15	T-box 15 [Source:HGNC Symbol;Acc:HGNC:11594]	-0.50036211	-0.50207789	-0.497557394	1.499997396	12
72	ENSG00000100292	HMOX1	heme oxygenase (decycling) 1 [Source:HGNC Symbol;Acc:HGNC:5013]	-0.50226204	-0.53840484	-0.458533068	1.499199951	12
73	ENSG00000100739	BDKRB1	bradykinin receptor B1 [Source:HGNC Symbol;Acc:HGNC:1029]	-0.5039536	-0.576078217	-0.41678352	1.496815337	12
74	ENSG00000101188	NTSR1	neurotensin receptor 1 (high affinity) [Source:HGNC Symbol;Acc:HGNC:8039]	-0.59160185	-0.62982646	-0.257439722	1.478868036	12
75	ENSG00000101463	SYNDIG1	synapse differentiation inducing 1 [Source:HGNC Symbol;Acc:HGNC:15885]	-0.61134061	-0.678590109	-0.171679455	1.461610171	12
76	ENSG00000101938	CHRDL1	chordin-like 1 [Source:HGNC Symbol;Acc:HGNC:29861]	-0.54724291	-0.548936975	-0.400168521	1.496348405	12
77	ENSG00000103196	CRISPLD2	cysteine-rich secretory protein LCCL domain containing 2 [Source:HGNC Symt	-0.5009545	-0.524120197	-0.474618555	1.499693248	12
78	ENSG00000103489	XYLT1	xylosyltransferase I [Source:HGNC Symbol;Acc:HGNC:15516]	-0.59602991	-0.702627035	-0.159315036	1.457971985	12
79	ENSG00000103647	CORO2B	coronin, actin binding protein, 2B [Source:HGNC Symbol;Acc:HGNC:2256]	-0.56153881	-0.891872144	0.081323211	1.372087744	12
80	ENSG00000103942	HOMER2	homer homolog 2 (Drosophila) [Source:HGNC Symbol;Acc:HGNC:17513]	-0.57066289	-0.615136866	-0.299537788	1.485337543	12
81	ENSG00000104419	NDRG1	N-myc downstream regulated 1 [Source:HGNC Symbol;Acc:HGNC:7679]	-0.60055695	-1.061121008	0.573189265	1.08848869	12
82	ENSG00000104490	NCALD	neurocalcin delta [Source:HGNC Symbol;Acc:HGNC:7655]	-0.6889789	-0.703606797	-0.031923492	1.424509192	12
83	ENSG00000105664	COMP	cartilage oligomeric matrix protein [Source:HGNC Symbol;Acc:HGNC:2227]	-0.50002045	-0.501172953	-0.4988059	1.499999299	12
84	ENSG00000105889	STEAP1B	STEAP family member 1B [Source:HGNC Symbol;Acc:HGNC:41907]	-0.82206033	-0.874188215	0.601768618	1.094479926	12
85	ENSG00000105976	MET	MET proto-oncogene, receptor tyrosine kinase [Source:HGNC Symbol;Acc:HG	-0.56986565	-1.100674671	0.650316188	1.020224128	12
86	ENSG00000106809	OGN	osteoglycin [Source:HGNC Symbol;Acc:HGNC:8126]	-0.49791819	-0.507772527	-0.494284935	1.499975648	12
87	ENSG00000107105	ELAVL2	ELAV like neuron-specific RNA binding protein 2 [Source:HGNC Symbol;Acc:H(	-0.82903973	-0.875355736	0.635444969	1.068950494	12
88	ENSG00000108551	RASD1	RAS, dexamethasone-induced 1 [Source:HGNC Symbol;Acc:HGNC:15828]	-0.54021271	-0.560436243	-0.395288386	1.495937343	12
89	ENSG00000109321	AREG	amphiregulin [Source:HGNC Symbol;Acc:HGNC:651]	-0.586914	-0.663790189	-0.22104736	1.471751545	12
90	ENSG00000110076	NRXN2	neurexin 2 [Source:HGNC Symbol;Acc:HGNC:8009]	-0.51604697	-0.537498625	-0.44528985	1.498835448	12
91	ENSG00000111262	KCNA1	potassium voltage-gated channel, shaker-related subfamily, member 1 (episo	-0.60572925	-0.626883715	-0.244054347	1.476667314	12
92	ENSG00000112320	SOBP	sine oculis binding protein homolog (Drosophila) [Source:HGNC Symbol;Acc:H	-0.58383291	-0.598880629	-0.303370974	1.486084512	12
93	ENSG00000112379	KIAA1244	KIAA1244 [Source:HGNC Symbol;Acc:HGNC:21213]	-0.50875453	-0.578189957	-0.409455097	1.496399587	12
94	ENSG00000112619	PRPH2	peripherin 2 (retinal degeneration, slow) [Source:HGNC Symbol;Acc:HGNC:99	-0.558316	-0.718054734	-0.185865452	1.462236189	12
95	ENSG00000112769	LAMA4	laminin, alpha 4 [Source:HGNC Symbol;Acc:HGNC:6484]	-0.19744465	-1.10126898	-0.023284877	1.321998509	12
96	ENSG00000113389	NPR3	natriuretic peptide receptor 3 [Source:HGNC Symbol;Acc:HGNC:7945]	-0.83926274	-0.868115206	0.648208677	1.059169272	12
97	ENSG00000113448	PDE4D	phosphodiesterase 4D, cAMP-specific [Source:HGNC Symbol;Acc:HGNC:8783]	-0.54660909	-0.568485677	-0.379555695	1.494650464	12
98	ENSG00000113645	WWC1	WW and C2 domain containing 1 [Source:HGNC Symbol;Acc:HGNC:29435]	-0.42830803	-0.663940524	-0.39707023	1.489318785	12
99	ENSG00000114757	PEX5L	peroxisomal biogenesis factor 5-like [Source:HGNC Symbol;Acc:HGNC:30024]	-0.77932001	-0.846647008	0.392290752	1.233676267	12
100	ENSG00000115252	PDE1A	phosphodiesterase 1A, calmodulin-dependent [Source:HGNC Symbol;Acc:HGI	-0.50956028	-0.51362179	-0.476611933	1.499794001	12
101	ENSG00000115844	DLX2	distal-less homeobox 2 [Source:HGNC Symbol;Acc:HGNC:2915]	-0.61299163	-0.648954954	-0.207729314	1.469675902	12
102	ENSG00000115884	SDC1	syndecan 1 [Source:HGNC Symbol;Acc:HGNC:10658]	-0.47562252	-0.753037246	-0.237701719	1.466361488	12
103	ENSG00000116176	TPSG1	tryptase gamma 1 [Source:HGNC Symbol;Acc:HGNC:14134]	-0.74295253	-0.960727834	0.659798301	1.043882065	12
104	ENSG00000116774	OLFML3	olfactomedin-like 3 [Source:HGNC Symbol;Acc:HGNC:24956]	-0.29563337	-0.965448834	-0.139311255	1.400393457	12
105	ENSG00000116852	KIF21B	kinesin family member 21B [Source:HGNC Symbol;Acc:HGNC:29442]	-0.2968222	-0.94065762	-0.175420549	1.41290037	12
106	ENSG00000118513	MYB	v-myb avian myeloblastosis viral oncogene homolog [Source:HGNC Symbol;A(	-0.64778491	-0.666430692	-0.140596248	1.454811855	12
107	ENSG00000118596	SLC16A7	solute carrier family 16 (monocarboxylate transporter), member 7 [Source:H(	-0.28838061	-0.889926951	-0.256618734	1.434926295	12
108	ENSG00000118785	SPP1	secreted phosphoprotein 1 [Source:HGNC Symbol;Acc:HGNC:11255]	-0.83903933	-0.849515905	0.571581498	1.116973735	12
109	ENSG00000118985	ELL2	elongation factor, RNA polymerase II, 2 [Source:HGNC Symbol;Acc:HGNC:170	-0.56063713	-0.650485629	-0.268837072	1.479959827	12
110	ENSG00000119630	PGF	placental growth factor [Source:HGNC Symbol;Acc:HGNC:8893]	-0.54838604	-0.570661194	-0.37521838	1.494265613	12
111	ENSG00000119714	GPR68	G protein-coupled receptor 68 [Source:HGNC Symbol;Acc:HGNC:4519]	-0.51625698	-0.542906927	-0.43939088	1.498554788	12
112	ENSG00000120051	CCDC147	cilia and flagella associated protein 58 [Source:HGNC Symbol;Acc:HGNC:2667	-0.4683068	-0.851037307	-0.110422034	1.429766141	12
113	ENSG00000120341	SEC16B	SEC16 homolog B (S. cerevisiae) [Source:HGNC Symbol;Acc:HGNC:30301]	-0.51792633	-0.587209679	-0.389842282	1.494978295	12
114	ENSG00000120549	KIAA1217	KIAA1217 [Source:HGNC Symbol;Acc:HGNC:25428]	-0.62348718	-0.733982643	-0.079938625	1.437408445	12
115	ENSG00000120693	SMAD9	SMAD family member 9 [Source:HGNC Symbol;Acc:HGNC:6774]	-0.67884394	-0.906488231	0.312660393	1.272671782	12
116	ENSG00000120738	EGR1	early growth response 1 [Source:HGNC Symbol;Acc:HGNC:3238]	-0.48356177	-0.551115841	-0.464282422	1.498960032	12
117	ENSG00000120885	CLU	clusterin [Source:HGNC Symbol;Acc:HGNC:2095]	-0.48813757	-0.536710535	-0.474618674	1.499466781	12
118	ENSG00000122420	PTGFR	prostaglandin F receptor (FP) [Source:HGNC Symbol;Acc:HGNC:9600]	-0.54434718	-0.590358934	-0.357683017	1.492389135	12
119	ENSG00000122641	INHBA	inhibin, beta A [Source:HGNC Symbol;Acc:HGNC:6066]	-0.46081603	-0.79927868	-0.19302094	1.453115651	12
120	ENSG00000123496	IL13RA2	interleukin 13 receptor, alpha 2 [Source:HGNC Symbol;Acc:HGNC:5975]	-0.56582256	-0.635658233	-0.280737163	1.48221796	12
121	ENSG00000124882	EREG	epiregulin [Source:HGNC Symbol;Acc:HGNC:3443]	-0.57937975	-0.616762176	-0.287520863	1.48366279	12
122	ENSG00000125148	MT2A	metallothionein 2A [Source:HGNC Symbol;Acc:HGNC:7406]	-0.60287181	-0.729770619	-0.113426346	1.446068772	12
123	ENSG00000125430	HS3ST3B1	heparan sulfate (glucosamine) 3-O-sulfotransferase 3B1 [Source:HGNC Symbo	-0.54495826	-0.575900758	-0.373164712	1.494023731	12
124	ENSG00000126778	SIX1	SIX homeobox 1 [Source:HGNC Symbol;Acc:HGNC:10887]	-0.58584483	-0.586743567	-0.315103623	1.487692016	12
125	ENSG00000127152	BCL11B	B-cell CLL/lymphoma 11B (zinc finger protein) [Source:HGNC Symbol;Acc:HGN	-0.5259409	-0.607519147	-0.358456661	1.491916712	12
126	ENSG00000127507	EMR2	egf-like module containing, mucin-like, hormone receptor-like 2 [Source:HGN	-0.50102072	-0.503244192	-0.49572763	1.499992545	12
127	ENSG00000127863	TNFRSF19	tumor necrosis factor receptor superfamily, member 19 [Source:HGNC Symbo	-0.79576029	-0.883219266	0.541829637	1.137149918	12
128	ENSG00000127946	HIP1	huntingtin interacting protein 1 [Source:HGNC Symbol;Acc:HGNC:4913]	-0.68177709	-0.684581512	-0.069502644	1.43586125	12
129	ENSG00000128564	VGF	VGF nerve growth factor inducible [Source:HGNC Symbol;Acc:HGNC:12684]	-0.5390947	-0.540126679	-0.418324767	1.497546147	12
130	ENSG00000128594	LRRC4	leucine rich repeat containing 4 [Source:HGNC Symbol;Acc:HGNC:15586]	-0.58070491	-0.652124981	-0.243114007	1.4759439	12
131	ENSG00000129009	ISLR	immunoglobulin superfamily containing leucine-rich repeat [Source:HGNC Syı	-0.47382028	-0.56177856	-0.462931615	1.498530452	12
132	ENSG00000129910	CDH15	cadherin 15, type 1, M-cadherin (myotubule) [Source:HGNC Symbol;Acc:HGN	-0.51183608	-0.516566236	-0.471287934	1.499690247	12
133	ENSG00000131016	AKAP12	A kinase (PRKA) anchor protein 12 [Source:HGNC Symbol;Acc:HGNC:370]	-0.63751831	-0.819618718	0.072759322	1.384377708	12
134	ENSG00000131737	KRT34	keratin 34 [Source:HGNC Symbol;Acc:HGNC:6452]	-0.50045218	-0.509879452	-0.489616967	1.499948595	12
135	ENSG00000131831	RAI2	retinoic acid induced 2 [Source:HGNC Symbol;Acc:HGNC:9835]	-0.58904305	-0.655567802	-0.228791238	1.473402088	12
136	ENSG00000132359	RAP1GAP2	RAP1 GTPase activating protein 2 [Source:HGNC Symbol;Acc:HGNC:29176]	-0.63806181	-1.020347876	0.532046629	1.126363053	12
137	ENSG00000132429	POPDC3	popeye domain containing 3 [Source:HGNC Symbol;Acc:HGNC:17649]	-0.76442973	-0.923502635	0.581066725	1.106865636	12
138	ENSG00000132623	ANKEF1	ankyrin repeat and EF-hand domain containing 1 [Source:HGNC Symbol;Acc:H	-0.5606585	-1.101239989	0.61649736	1.045401134	12
139	ENSG00000132639	SNAP25	synaptosomal-associated protein, 25kDa [Source:HGNC Symbol;Acc:HGNC:11	-0.73974572	-0.762683277	0.14193457	1.360494422	12
140	ENSG00000134531	EMP1	epithelial membrane protein 1 [Source:HGNC Symbol;Acc:HGNC:3333]	-0.59494973	-0.769240628	-0.067503384	1.431693741	12
141	ENSG00000134532	SOX5	SRY (sex determining region Y)-box 5 [Source:HGNC Symbol;Acc:HGNC:11201	-0.48746283	-0.554398871	-0.456894287	1.498755983	12

142	ENSG00000135111	TBX3	T-box 3 [Source:HGNC Symbol;Acc:HGNC:11602]	-0.41860154	-0.773417385	-0.274663768	1.466682691	12
143	ENSG00000135750	KCNK1	potassium channel, subfamily K, member 1 [Source:HGNC Symbol;Acc:HGNC:	-0.57390359	-0.677228113	-0.219813199	1.470944903	12
144	ENSG00000136160	EDNRB	endothelin receptor type B [Source:HGNC Symbol;Acc:HGNC:3180]	-0.58885091	-0.679589455	-0.198528965	1.466969333	12
145	ENSG00000136531	SCN2A	sodium channel, voltage-gated, type II, alpha subunit [Source:HGNC Symbol;A	-0.63010593	-1.068285401	0.750539048	0.947852285	12
146	ENSG00000136630	HLX	H2.0-like homeobox [Source:HGNC Symbol;Acc:HGNC:4978]	-0.51307809	-0.779298179	-0.158276594	1.450652866	12
147	ENSG00000136928	GABBR2	gamma-aminobutyric acid (GABA) B receptor, 2 [Source:HGNC Symbol;Acc:HG	-0.1574384	-1.0519102	-0.149459664	1.358808261	12
148	ENSG00000136960	ENPP2	ectonucleotide pyrophosphatase/phosphodiesterase 2 [Source:HGNC Symbol	-0.52307791	-0.523362238	-0.452731501	1.499171655	12
149	ENSG00000137265	IRF4	interferon regulatory factor 4 [Source:HGNC Symbol;Acc:HGNC:6119]	-0.54208533	-0.549296729	-0.405324007	1.496706066	12
150	ENSG00000137273	FOXF2	forkhead box F2 [Source:HGNC Symbol;Acc:HGNC:3810]	-0.67792819	-0.689832562	-0.067504906	1.435265654	12
151	ENSG00000137726	FXYD6	FXYD domain containing ion transport regulator 6 [Source:HGNC Symbol;Acc:	-0.48904485	-0.637969389	-0.363517593	1.490531835	12
152	ENSG00000137745	MMP13	matrix metallopeptidase 13 (collagenase 3) [Source:HGNC Symbol;Acc:HGNC:	-0.53743817	-0.568668639	-0.389294745	1.495401551	12
153	ENSG00000137819	PAQR5	progestin and adipoQ receptor family member V [Source:HGNC Symbol;Acc:H	-0.67677785	-0.733147395	-0.005854526	1.415779772	12
154	ENSG00000138166	DUSP5	dual specificity phosphatase 5 [Source:HGNC Symbol;Acc:HGNC:3071]	-0.52413867	-0.708351902	-0.239324626	1.471815194	12
155	ENSG00000138623	SEMA7A	semaphorin 7A, GPI membrane anchor (John Milton Hagen blood group) [Sou	-0.72355524	-0.749067589	0.091696585	1.380926246	12
156	ENSG00000138678	AGPAT9	1-acylglycerol-3-phosphate O-acyltransferase 9 [Source:HGNC Symbol;Acc:HG	-0.62288358	-0.707339945	-0.11813116	1.448354681	12
157	ENSG00000138696	BMPR1B	bone morphogenetic protein receptor, type IB [Source:HGNC Symbol;Acc:HGI	-0.59576523	-0.61080103	-0.275376313	1.48194257	12
158	ENSG00000139364	TMEM132B	transmembrane protein 132B [Source:HGNC Symbol;Acc:HGNC:29397]	-0.79180428	-0.935943809	0.816448096	0.911299992	12
159	ENSG00000139668	WDFY2	WD repeat and FYVE domain containing 2 [Source:HGNC Symbol;Acc:HGNC:2	-0.64316498	-0.788286337	0.030008146	1.401443173	12
160	ENSG00000140557	ST8SIA2	ST8 alpha-N-acetyl-neuraminide alpha-2,8-sialyltransferase 2 [Source:HGNC S	-0.77491828	-0.775020937	0.228303148	1.321636064	12
161	ENSG00000141750	STAC2	SH3 and cysteine rich domain 2 [Source:HGNC Symbol;Acc:HGNC:23990]	-0.43737411	-0.655917148	-0.396973385	1.490264645	12
162	ENSG00000141837	CACNA1A	calcium channel, voltage-dependent, P/Q type, alpha 1A subunit [Source:HGN	-0.44838452	-0.639734937	-0.404014252	1.492133712	12
163	ENSG00000142156	COL6A1	collagen, type VI, alpha 1 [Source:HGNC Symbol;Acc:HGNC:2211]	-0.56225677	-0.639138965	-0.280691054	1.482086787	12
164	ENSG00000142173	COL6A2	collagen, type VI, alpha 2 [Source:HGNC Symbol;Acc:HGNC:2212]	-0.59268878	-0.680164544	-0.193036545	1.465889866	12
165	ENSG00000142512	SIGLEC10	sialic acid binding Ig-like lectin 10 [Source:HGNC Symbol;Acc:HGNC:15620]	-0.50813518	-0.568768705	-0.420307625	1.497211508	12
166	ENSG00000142611	PRDM16	PR domain containing 16 [Source:HGNC Symbol;Acc:HGNC:14000]	-0.81331815	-0.909242111	0.742567393	0.979992873	12
167	ENSG00000143067	ZNF697	zinc finger protein 697 [Source:HGNC Symbol;Acc:HGNC:32034]	-0.45776997	-0.628753917	-0.406698082	1.493221966	12
168	ENSG00000143469	SYT14	synaptotagmin XIV [Source:HGNC Symbol;Acc:HGNC:23143]	-0.64222197	-0.989332393	0.443092636	1.188461726	12
169	ENSG00000143473	KCNH1	potassium voltage-gated channel, subfamily H (eag-related), member 1 [Sour	-0.47734146	-0.585033801	-0.434617727	1.49699299	12
170	ENSG00000144218	AFF3	AF4/FMR2 family, member 3 [Source:HGNC Symbol;Acc:HGNC:6473]	-0.74017801	-0.750866565	0.122333331	1.368711244	12
171	ENSG00000144278	GALNT13	polypeptide N-acetylgalactosaminyltransferase 13 [Source:HGNC Symbol;Acc	-0.58406253	-0.743403305	-0.118935507	1.44640134	12
172	ENSG00000144339	TMEFF2	transmembrane protein with EGF-like and two follistatin-like domains 2 [Sour	-0.649587	-0.76524186	0.003293958	1.411534901	12
173	ENSG00000146938	NLGN4X	neuroligin 4, X-linked [Source:HGNC Symbol;Acc:HGNC:14287]	-0.55662189	-0.563123066	-0.374512372	1.494257329	12
174	ENSG00000147100	SLC16A2	solute carrier family 16, member 2 (thyroid hormone transporter) [Source:HG	-0.49377364	-0.647669893	-0.347228297	1.488671829	12
175	ENSG00000147255	IGSF1	immunoglobulin superfamily, member 1 [Source:HGNC Symbol;Acc:HGNC:59	-0.4619304	-0.867971548	-0.092982435	1.422884382	12
176	ENSG00000148841	ITPRIP	inositol 1,4,5-trisphosphate receptor interacting protein [Source:HGNC Symb	-0.51656345	-0.669723607	-0.295955456	1.482242511	12
177	ENSG00000149090	PAMR1	peptidase domain containing associated with muscle regeneration 1 [Source:	-0.59320938	-0.79954206	-0.024372791	1.417124233	12
178	ENSG00000150471	LPHN3	latrophilin 3 [Source:HGNC Symbol;Acc:HGNC:20974]	-0.66501661	-1.013156712	0.59096016	1.087213166	12
179	ENSG00000151617	EDNRA	endothelin receptor type A [Source:HGNC Symbol;Acc:HGNC:3179]	-0.51753815	-0.559647463	-0.420256653	1.497442263	12
180	ENSG00000151692	RNF144A	ring finger protein 144A [Source:HGNC Symbol;Acc:HGNC:20457]	-0.61433423	-0.675537149	-0.17187485	1.461746232	12
181	ENSG00000152049	KCNE4	potassium voltage-gated channel, Isk-related family, member 4 [Source:HGN	-0.63472622	-0.646016842	-0.184138468	1.464881528	12
182	ENSG00000152217	SETBP1	SET binding protein 1 [Source:HGNC Symbol;Acc:HGNC:15573]	-0.58871819	-0.65973488	-0.223960724	1.472413793	12
183	ENSG00000152270	PDE3B	phosphodiesterase 3B, cGMP-inhibited [Source:HGNC Symbol;Acc:HGNC:877	-0.53026902	-0.587808903	-0.37590658	1.493984505	12
184	ENSG00000153132	CLGN	calmegin [Source:HGNC Symbol;Acc:HGNC:2060]	-0.60254532	-0.869898901	0.105282718	1.367161506	12
185	ENSG00000153714	LURAP1L	leucine rich adaptor protein 1-like [Source:HGNC Symbol;Acc:HGNC:31452]	-0.80541873	-0.888550479	0.594979774	1.098989436	12
186	ENSG00000153976	HS3ST3A1	heparan sulfate (glucosamine) 3-O-sulfotransferase 3A1 [Source:HGNC Symb	-0.54253004	-0.662666475	-0.274979369	1.480175883	12
187	ENSG00000154188	ANGPT1	angiopoietin 1 [Source:HGNC Symbol;Acc:HGNC:484]	-0.83646826	-0.8946089	0.836541024	0.894536131	12
188	ENSG00000154217	PITPNC1	phosphatidylinositol transfer protein, cytoplasmic 1 [Source:HGNC Symbol;Ac	-0.66700196	-0.80172795	0.088703707	1.380026206	12
189	ENSG00000154928	EPHB1	EPH receptor B1 [Source:HGNC Symbol;Acc:HGNC:3392]	-0.63418946	-0.643844323	-0.187597606	1.465631386	12
190	ENSG00000155961	RAB39B	RAB39B, member RAS oncogene family [Source:HGNC Symbol;Acc:HGNC:164	-0.50974958	-0.533684234	-0.455769524	1.499203337	12
191	ENSG00000155966	AFF2	AF4/FMR2 family, member 2 [Source:HGNC Symbol;Acc:HGNC:3776]	-0.63401771	-0.982870917	0.405441904	1.211446724	12
192	ENSG00000156298	TSPAN7	tetraspanin 7 [Source:HGNC Symbol;Acc:HGNC:11854]	-0.3711911	-0.774841669	-0.32284689	1.468879656	12
193	ENSG00000157214	STEAP2	STEAP family member 2, metalloreductase [Source:HGNC Symbol;Acc:HGNC:	-0.70335276	-0.828211212	0.19722373	1.334340243	12
194	ENSG00000157240	FZD1	frizzled class receptor 1 [Source:HGNC Symbol;Acc:HGNC:4038]	-0.61865171	-0.634321845	-0.219070576	1.472044135	12
195	ENSG00000157551	KCNJ15	potassium inwardly-rectifying channel, subfamily J, member 15 [Source:HGN	-0.5010936	-0.502198003	-0.496704177	1.499995778	12
196	ENSG00000157617	C2CD2	C2 calcium-dependent domain containing 2 [Source:HGNC Symbol;Acc:HGNC	-0.53220067	-0.53569275	-0.430314027	1.498207449	12
197	ENSG00000157766	ACAN	aggrecan [Source:HGNC Symbol;Acc:HGNC:319]	-0.57294713	-0.581869897	-0.335377831	1.490194859	12
198	ENSG00000158321	AUTS2	autism susceptibility candidate 2 [Source:HGNC Symbol;Acc:HGNC:14262]	-0.70101905	-0.703934104	-0.013799339	1.418752495	12
199	ENSG00000159167	STC1	stanniocalcin 1 [Source:HGNC Symbol;Acc:HGNC:11373]	-0.69109979	-0.71871411	-0.006445104	1.416259004	12
200	ENSG00000159173	TNNI1	troponin I type 1 (skeletal, slow) [Source:HGNC Symbol;Acc:HGNC:11945]	-0.57615385	-0.59970044	-0.311264009	1.4871183	12
201	ENSG00000159753	RLTPR	RGD motif, leucine rich repeats, tropomodulin domain and proline-rich conta	-0.57951001	-0.592616362	-0.315611335	1.487737706	12
202	ENSG00000160801	PTH1R	parathyroid hormone 1 receptor [Source:HGNC Symbol;Acc:HGNC:9608]	-0.51580312	-0.545960199	-0.436641966	1.498405284	12
203	ENSG00000162373	BEND5	BEN domain containing 5 [Source:HGNC Symbol;Acc:HGNC:25668]	-0.59706223	-0.630232722	-0.250411829	1.47770678	12
204	ENSG00000162407	PPAP2B	phosphatidic acid phosphatase type 2B [Source:HGNC Symbol;Acc:HGNC:922	-0.49876463	-0.585177111	-0.412318586	1.49626033	12
205	ENSG00000162595	DIRAS3	DIRAS family, GTP-binding RAS-like 3 [Source:HGNC Symbol;Acc:HGNC:687]	-0.41116881	-0.742758742	-0.321221969	1.47514952	12
206	ENSG00000162631	NTNG1	netrin G1 [Source:HGNC Symbol;Acc:HGNC:23319]	-0.68373802	-0.801941728	0.116034635	1.369645116	12
207	ENSG00000162804	SNED1	sushi, nidogen and EGF-like domains 1 [Source:HGNC Symbol;Acc:HGNC:2469	-0.61367219	-0.67605194	-0.172042473	1.461766604	12
208	ENSG00000163012	ZSWIM2	zinc finger, SWIM-type containing 2 [Source:HGNC Symbol;Acc:HGNC:30990]	-0.5264846	-0.601841589	-0.364287403	1.492613597	12
209	ENSG00000163395	IGFN1	immunoglobulin-like and fibronectin type III domain containing 1 [Source:HGI	-0.41509694	-0.690563719	-0.379793269	1.485453923	12
210	ENSG00000163618	CADPS	Ca++-dependent secretion activator [Source:HGNC Symbol;Acc:HGNC:1426]	-0.5872696	-0.591175586	-0.308346157	1.486791341	12
211	ENSG00000164106	SCRG1	stimulator of chondrogenesis 1 [Source:HGNC Symbol;Acc:HGNC:17036]	-0.50435195	-0.518745105	-0.476674336	1.499771386	12
212	ENSG00000164483	SAMD3	sterile alpha motif domain containing 3 [Source:HGNC Symbol;Acc:HGNC:215	-0.85970923	-0.87168434	0.832495794	0.898897774	12
213	ENSG00000164484	TMEM200A	transmembrane protein 200A [Source:HGNC Symbol;Acc:HGNC:21075]	-0.80074271	-0.927328262	0.810375653	0.917695322	12
214	ENSG00000164647	STEAP1	six transmembrane epithelial antigen of the prostate 1 [Source:HGNC Symbol	-0.72083959	-0.884115526	0.349272554	1.255682564	12
215	ENSG00000164920	OSR2	odd-skipped related transciption factor 2 [Source:HGNC Symbol;Acc:HGNC:1	-0.82727585	-0.848063526	0.527007562	1.148331813	12
216	ENSG00000165194	PCDH19	protocadherin 19 [Source:HGNC Symbol;Acc:HGNC:14270]	-0.49384692	-0.513304149	-0.492782194	1.499933259	12
217	ENSG00000165323	FAT3	FAT atypical cadherin 3 [Source:HGNC Symbol;Acc:HGNC:23112]	-0.50694152	-0.660628028	-0.317595162	1.48516471	12
218	ENSG00000165379	LRFN5	leucine rich repeat and fibronectin type III domain containing 5 [Source:HGN	-0.72898537	-0.84127457	0.271330749	1.298929193	12
219	ENSG00000165633	VSTM4	V-set and transmembrane domain containing 4 [Source:HGNC Symbol;Acc:HG	-0.53048406	-0.689424005	-0.255838486	1.475746548	12
220	ENSG00000166033	HTRA1	HtrA serine peptidase 1 [Source:HGNC Symbol;Acc:HGNC:9476]	-0.7464902	-0.864111688	0.358621999	1.251979887	12
221	ENSG00000166250	CLMP	CXADR-like membrane protein [Source:HGNC Symbol;Acc:HGNC:24039]	-0.60518726	-0.808033634	0.005813262	1.40740763	12
222	ENSG00000166341	DCHS1	dachsous cadherin-related 1 [Source:HGNC Symbol;Acc:HGNC:13681]	-0.68807531	-0.995058398	0.596677463	1.086456241	12
223	ENSG00000166396	SERPINB7	serpin peptidase inhibitor, clade B (ovalbumin), member 7 [Source:HGNC Sym	-0.67346082	-0.67347337	-0.09652261	1.443456799	12
224	ENSG00000166825	ANPEP	alanyl (membrane) aminopeptidase [Source:HGNC Symbol;Acc:HGNC:500]	-0.64346851	-0.746233619	-0.034342888	1.424045021	12
225	ENSG00000166923	GREM1	gremlin 1, DAN family BMP antagonist [Source:HGNC Symbol;Acc:HGNC:2001	-0.68671878	-0.805790222	0.127656136	1.364852866	12

226	ENSG00000167103	PIP5KL1	phosphatidylinositol-4-phosphate 5-kinase-like 1 [Source:HGNC Symbol;Acc:H	-0.4804458	-0.575669455	-0.441500725	1.49761598	12
227	ENSG00000167157	PRRX2	paired related homeobox 2 [Source:HGNC Symbol;Acc:HGNC:21338]	-0.82626483	-0.830691252	0.471766143	1.185189936	12
228	ENSG00000167693	NXN	nucleoredoxin [Source:HGNC Symbol;Acc:HGNC:18008]	-0.66051987	-0.667418091	-0.122337885	1.450275847	12
229	ENSG00000168077	SCARA3	scavenger receptor class A, member 3 [Source:HGNC Symbol;Acc:HGNC:1900	-0.62773726	-0.674105502	-0.156584172	1.458426929	12
230	ENSG00000168398	BDKRB2	bradykinin receptor B2 [Source:HGNC Symbol;Acc:HGNC:1030]	-0.52486805	-0.537760187	-0.435830686	1.498458918	12
231	ENSG00000168685	IL7R	interleukin 7 receptor [Source:HGNC Symbol;Acc:HGNC:6024]	-0.82924857	-0.83447332	0.491261604	1.172460287	12
232	ENSG00000168779	SHOX2	short stature homeobox 2 [Source:HGNC Symbol;Acc:HGNC:10854]	-0.47983099	-0.638426662	-0.372784134	1.491041786	12
233	ENSG00000169047	IRS1	insulin receptor substrate 1 [Source:HGNC Symbol;Acc:HGNC:6125]	-0.63648445	-0.709070686	-0.097562286	1.443117425	12
234	ENSG00000169436	COL22A1	collagen, type XXII, alpha 1 [Source:HGNC Symbol;Acc:HGNC:22989]	-0.54250581	-0.916274499	0.096101715	1.362678594	12
235	ENSG00000169884	WNT10B	wingless-type MMTV integration site family, member 10B [Source:HGNC Sym	-0.46825079	-0.698989691	-0.313856627	1.481097104	12
236	ENSG00000170166	HOXD4	homeobox D4 [Source:HGNC Symbol;Acc:HGNC:5138]	-0.58124332	-0.622360439	-0.27867886	1.482282623	12
237	ENSG00000170370	EMX2	empty spiracles homeobox 2 [Source:HGNC Symbol;Acc:HGNC:3341]	-0.50914918	-0.524284946	-0.466110328	1.499544454	12
238	ENSG00000170396	ZNF804A	zinc finger protein 804A [Source:HGNC Symbol;Acc:HGNC:21711]	-0.28716249	-1.171007657	0.237582941	1.220587203	12
239	ENSG00000170961	HAS2	hyaluronan synthase 2 [Source:HGNC Symbol;Acc:HGNC:4819]	-0.52671286	-0.563968014	-0.405901192	1.496582062	12
240	ENSG00000171051	FPR1	formyl peptide receptor 1 [Source:HGNC Symbol;Acc:HGNC:3826]	-0.51720498	-0.522522199	-0.459665188	1.499392368	12
241	ENSG00000171094	ALK	anaplastic lymphoma receptor tyrosine kinase [Source:HGNC Symbol;Acc:HGI	-0.5040367	-0.507635158	-0.48827512	1.499946983	12
242	ENSG00000171246	NPTX1	neuronal pentraxin I [Source:HGNC Symbol;Acc:HGNC:7952]	-0.80233471	-0.815063514	0.370554164	1.246844056	12
243	ENSG00000171310	CHST11	carbohydrate (chondroitin 4) sulfotransferase 11 [Source:HGNC Symbol;Acc:H	-0.30281132	-1.159270794	0.233268783	1.228813332	12
244	ENSG00000171587	DSCAM	Down syndrome cell adhesion molecule [Source:HGNC Symbol;Acc:HGNC:303	-0.8405904	-0.883692816	0.748302918	0.975980295	12
245	ENSG00000171812	COL8A2	collagen, type VIII, alpha 2 [Source:HGNC Symbol;Acc:HGNC:2216]	-0.49806675	-0.603263324	-0.393140793	1.494470872	12
246	ENSG00000172164	SNTB1	syntrophin, beta 1 (dystrophin-associated protein A1, 59kDa, basic componer	-0.57159104	-0.574033917	-0.345757185	1.491382147	12
247	ENSG00000172348	RCAN2	regulator of calcineurin 2 [Source:HGNC Symbol;Acc:HGNC:3041]	-0.73537819	-0.799147399	0.200165827	1.334359767	12
248	ENSG00000172789	HOXC5	homeobox C5 [Source:HGNC Symbol;Acc:HGNC:5127]	-0.5496986	-0.604011443	-0.336238124	1.489948169	12
249	ENSG00000172935	MRGPRF	MAS-related GPR, member F [Source:HGNC Symbol;Acc:HGNC:24828]	-0.6458133	-0.841038261	0.122855055	1.363996504	12
250	ENSG00000173376	NDNF	neuron-derived neurotrophic factor [Source:HGNC Symbol;Acc:HGNC:26256]	-0.59777198	-0.603030478	-0.282225426	1.483027886	12
251	ENSG00000173848	NET1	neuroepithelial cell transforming 1 [Source:HGNC Symbol;Acc:HGNC:14592]	-0.71443289	-0.805849344	0.175092646	1.345189592	12
252	ENSG00000174136	RGMB	repulsive guidance molecule family member b [Source:HGNC Symbol;Acc:HGI	-0.45650146	-0.619449165	-0.418333809	1.494284435	12
253	ENSG00000174332	GLIS1	GLIS family zinc finger 1 [Source:HGNC Symbol;Acc:HGNC:29525]	-0.52573613	-0.682496154	-0.269922726	1.478155012	12
254	ENSG00000174469	CNTNAP2	contactin associated protein-like 2 [Source:HGNC Symbol;Acc:HGNC:13830]	-0.53420921	-0.654091269	-0.294881856	1.483182331	12
255	ENSG00000174607	UGT8	UDP glycosyltransferase 8 [Source:HGNC Symbol;Acc:HGNC:12555]	-0.77014687	-0.93846186	0.671970576	1.036638159	12
256	ENSG00000175471	MCTP1	multiple C2 domains, transmembrane 1 [Source:HGNC Symbol;Acc:HGNC:261	-0.53691061	-0.592973831	-0.362901953	1.492786391	12
257	ENSG00000175513	TSGA10IP	testis specific, 10 interacting protein [Source:HGNC Symbol;Acc:HGNC:26555]	-0.55718568	-0.907838074	0.103136455	1.3618873	12
258	ENSG00000176049	JAKMIP2	janus kinase and microtubule interacting protein 2 [Source:HGNC Symbol;Acc	-0.60420574	-0.643032624	-0.225981521	1.473219883	12
259	ENSG00000176842	IRX5	iroquois homeobox 5 [Source:HGNC Symbol;Acc:HGNC:14361]	-0.5239098	-0.560516812	-0.412603134	1.497029751	12
260	ENSG00000176971	FIBIN	fin bud initiation factor homolog (zebrafish) [Source:HGNC Symbol;Acc:HGNC	-0.48879148	-0.530038712	-0.480820832	1.499651021	12
261	ENSG00000177453	NIM1K	NIM1 serine/threonine protein kinase [Source:HGNC Symbol;Acc:HGNC:2864	-0.4889097	-0.632731813	-0.369657052	1.491298568	12
262	ENSG00000179046	TRIML2	tripartite motif family-like 2 [Source:HGNC Symbol;Acc:HGNC:26378]	-0.46772569	-0.568607925	-0.461864836	1.498198446	12
263	ENSG00000179314	WSCD1	WSC domain containing 1 [Source:HGNC Symbol;Acc:HGNC:29060]	-0.67398298	-0.736672406	-0.004632306	1.415287692	12
264	ENSG00000179388	EGR3	early growth response 3 [Source:HGNC Symbol;Acc:HGNC:3240]	-0.60275749	-0.648207951	-0.221291329	1.472256773	12
265	ENSG00000179981	TSHZ1	teashirt zinc finger homeobox 1 [Source:HGNC Symbol;Acc:HGNC:10669]	-0.6766327	-0.757138374	0.030933668	1.402837404	12
266	ENSG00000180537	RNF182	ring finger protein 182 [Source:HGNC Symbol;Acc:HGNC:28522]	-0.6261898	-0.666860981	-0.16808882	1.461139596	12
267	ENSG00000180660	MAB21L1	mab-21-like 1 (C. elegans) [Source:HGNC Symbol;Acc:HGNC:6757]	-0.57258161	-0.5761227	-0.342291765	1.490996072	12
268	ENSG00000180875	GREM2	gremlin 2, DAN family BMP antagonist [Source:HGNC Symbol;Acc:HGNC:1765	-0.58149102	-0.634943955	-0.26323456	1.479669535	12
269	ENSG00000182379	NXPH4	neurexophilin 4 [Source:HGNC Symbol;Acc:HGNC:8078]	-0.55746017	-0.56319384	-0.373514026	1.494168038	12
270	ENSG00000182771	GRID1	glutamate receptor, ionotropic, delta 1 [Source:HGNC Symbol;Acc:HGNC:457	-0.51670588	-0.516796049	-0.466069916	1.499571843	12
271	ENSG00000183837	PNMA3	paraneoplastic Ma antigen 3 [Source:HGNC Symbol;Acc:HGNC:18742]	-0.4770508	-0.557814283	-0.463840326	1.498705411	12
272	ENSG00000183876	ARSI	arylsulfatase family, member I [Source:HGNC Symbol;Acc:HGNC:32521]	-0.61100759	-0.707670921	-0.133256194	1.451934703	12
273	ENSG00000184156	KCNQ3	potassium voltage-gated channel, KQT-like subfamily, member 3 [Source:HGN	-0.40131751	-0.907571419	-0.106693918	1.415582843	12
274	ENSG00000184160	ADRA2C	adrenoceptor alpha 2C [Source:HGNC Symbol;Acc:HGNC:283]	-0.5036132	-0.504245879	-0.49211762	1.499976696	12
275	ENSG00000184557	SOCS3	suppressor of cytokine signaling 3 [Source:HGNC Symbol;Acc:HGNC:19391]	-0.54260739	-0.599005223	-0.349826707	1.491439318	12
276	ENSG00000184588	PDE4B	phosphodiesterase 4B, cAMP-specific [Source:HGNC Symbol;Acc:HGNC:8781]	-0.66550665	-0.676103652	-0.103799797	1.445410099	12
277	ENSG00000184985	SORCS2	sortilin-related VPS10 domain containing receptor 2 [Source:HGNC Symbol;Ac	-0.60099648	-0.678886385	-0.184335958	1.464218823	12
278	ENSG00000185274	WBSCR17	Williams-Beuren syndrome chromosome region 17 [Source:HGNC Symbol;Acc	-0.54606393	-0.56530673	-0.383641585	1.495012247	12
279	ENSG00000185559	DLK1	delta-like 1 homolog (Drosophila) [Source:HGNC Symbol;Acc:HGNC:2907]	-0.49995699	-0.50125692	-0.498785321	1.499999236	12
280	ENSG00000185585	OLFML2A	olfactomedin-like 2A [Source:HGNC Symbol;Acc:HGNC:27270]	-0.52810585	-0.598346409	-0.36646157	1.49291383	12
281	ENSG00000185862	EVI2B	ecotropic viral integration site 2B [Source:HGNC Symbol;Acc:HGNC:3500]	-0.67356538	-0.75952764	0.030051917	1.4030411	12
282	ENSG00000185960	SHOX	short stature homeobox [Source:HGNC Symbol;Acc:HGNC:10853]	-0.50001252	-0.50028717	-0.499700265	1.499999957	12
283	ENSG00000186297	GABRA5	gamma-aminobutyric acid (GABA) A receptor, alpha 5 [Source:HGNC Symbol;/	-0.58696485	-0.604741208	-0.292876025	1.484582085	12
284	ENSG00000186854	TRABD2A	TraB domain containing 2A [Source:HGNC Symbol;Acc:HGNC:27013]	-0.51063164	-0.608635751	-0.37375746	1.493024853	12
285	ENSG00000187634	SAMD11	sterile alpha motif domain containing 11 [Source:HGNC Symbol;Acc:HGNC:28	-0.64908511	-0.756809269	-0.010375585	1.416269961	12
286	ENSG00000187772	LIN28B	lin-28 homolog B (C. elegans) [Source:HGNC Symbol;Acc:HGNC:32207]	-0.49689674	-0.507883179	-0.495196368	1.499976287	12
287	ENSG00000188064	WNT7B	wingless-type MMTV integration site family, member 7B [Source:HGNC Symb	-0.68971996	-0.69413858	-0.044591528	1.42845007	12
288	ENSG00000188487	INSC	inscuteable homolog (Drosophila) [Source:HGNC Symbol;Acc:HGNC:33116]	-0.3204668	-0.89531978	-0.215707041	1.431493623	12
289	ENSG00000188916	FAM196A	family with sequence similarity 196, member A [Source:HGNC Symbol;Acc:HG	-0.58655415	-0.62932962	-0.264042004	1.47992577	12
290	ENSG00000189129	PLAC9	placenta-specific 9 [Source:HGNC Symbol;Acc:HGNC:19255]	-0.5135526	-1.073474517	0.390655852	1.196371262	12
291	ENSG00000189143	CLDN4	claudin 4 [Source:HGNC Symbol;Acc:HGNC:2046]	-0.48813809	-0.531793186	-0.479677552	1.499608833	12
292	ENSG00000189367	KIAA0408	KIAA0408 [Source:HGNC Symbol;Acc:HGNC:21636]	-0.34090546	-1.149787944	0.270642718	1.220050684	12
293	ENSG00000196460	RFX8	RFX family member 8, lacking RFX DNA binding domain [Source:HGNC Symbo	-0.75488346	-0.773272983	0.187637075	1.34051937	12
294	ENSG00000196549	MME	membrane metallo-endopeptidase [Source:HGNC Symbol;Acc:HGNC:7154]	-0.56065224	-0.689006102	-0.220782043	1.47044039	12
295	ENSG00000196569	LAMA2	laminin, alpha 2 [Source:HGNC Symbol;Acc:HGNC:6482]	-0.39707934	-0.729465625	-0.352056999	1.478601964	12
296	ENSG00000196581	AJAP1	adherens junctions associated protein 1 [Source:HGNC Symbol;Acc:HGNC:308	-0.50302713	-0.508101934	-0.488820991	1.499950056	12
297	ENSG00000196876	SCN8A	sodium channel, voltage gated, type VIII, alpha subunit [Source:HGNC Symbol	-0.68887965	-0.794480525	0.111574218	1.371785958	12
298	ENSG00000197106	SLC6A17	solute carrier family 6 (neutral amino acid transporter), member 17 [Source:H	-0.75629681	-0.760167633	0.166485605	1.349978835	12
299	ENSG00000197457	STMN3	stathmin-like 3 [Source:HGNC Symbol;Acc:HGNC:15926]	-0.65244165	-0.72095435	-0.0587671	1.4321631	12
300	ENSG00000197757	HOXC6	homeobox C6 [Source:HGNC Symbol;Acc:HGNC:5128]	-0.50819757	-0.510599154	-0.481068783	1.499865512	12
301	ENSG00000198353	HOXC4	homeobox C4 [Source:HGNC Symbol;Acc:HGNC:5126]	-0.61895344	-0.685049703	-0.153414913	1.45741806	12
302	ENSG00000198576	ARC	activity-regulated cytoskeleton-associated protein [Source:HGNC Symbol;Acc	-0.46512934	-0.57768106	-0.454866412	1.497676813	12
303	ENSG00000198732	SMOC1	SPARC related modular calcium binding 1 [Source:HGNC Symbol;Acc:HGNC:2(	-0.50190878	-0.5223301	-0.475485289	1.499724169	12
304	ENSG00000198846	TOX	thymocyte selection-associated high mobility group box [Source:HGNC Symbo	-0.84423844	-0.865114607	0.657326259	1.052026788	12
305	ENSG00000198885	ITPRIPL1	inositol 1,4,5-trisphosphate receptor interacting protein-like 1 [Source:HGNC	-0.54908072	-0.797203321	-0.087363363	1.433647402	12
306	ENSG00000204442	FAM155A	family with sequence similarity 155, member A [Source:HGNC Symbol;Acc:HG	-0.76139548	-0.773267605	0.19955573	1.335107354	12
307	ENSG00000205835	GMNC	geminin coiled-coil domain containing [Source:HGNC Symbol;Acc:HGNC:4004	-0.61657377	-1.083686728	0.847217133	0.853043368	12
308	ENSG00000206052	DOK6	docking protein 6 [Source:HGNC Symbol;Acc:HGNC:28301]	-0.63048684	-0.710228259	-0.104014347	1.444729448	12
309	ENSG00000212724	KRTAP2-3	keratin associated protein 2-3 [Source:HGNC Symbol;Acc:HGNC:18906]	-0.52898196	-0.552349162	-0.416008366	1.497339485	12

310	ENSG00000213853	EMP2	epithelial membrane protein 2 [Source:HGNC Symbol;Acc:HGNC:3334]	-0.65362883	-0.800001822	0.06490695	1.388723703	12
311	ENSG00000214338	SOGA3	SOGA family member 3 [Source:HGNC Symbol;Acc:HGNC:21494]	-0.470101	-0.935961276	0.02683739	1.379224888	12
312	ENSG00000214575	CPEB1	cytoplasmic polyadenylation element binding protein 1 [Source:HGNC Symbo	-0.55598286	-0.576565904	-0.36031114	1.492859906	12
313	ENSG00000218336	TENM3	teneurin transmembrane protein 3 [Source:HGNC Symbol;Acc:HGNC:29944]	-0.67365338	-0.807377509	0.109006762	1.372024128	12
314	ENSG00000222022	AC112721.1		-0.56487532	-0.608061863	-0.314443672	1.487380858	12
315	ENSG00000225968	ELFN1	extracellular leucine-rich repeat and fibronectin type III domain containing 1 [	-0.54453717	-0.553338241	-0.398329637	1.496205052	12
316	ENSG00000227051	C14orf132	chromosome 14 open reading frame 132 [Source:HGNC Symbol;Acc:HGNC:20	-0.6789551	-0.778957248	0.069603702	1.388308647	12
317	ENSG00000244242	IFITM10	interferon induced transmembrane protein 10 [Source:HGNC Symbol;Acc:HG	-0.54846385	-0.557785281	-0.389257895	1.495507029	12
318	ENSG00000249992	TMEM158	transmembrane protein 158 (gene/pseudogene) [Source:HGNC Symbol;Acc:H	-0.51428172	-0.909329564	0.042739314	1.380871973	12
319	ENSG00000253293	HOXA10	homeobox A10 [Source:HGNC Symbol;Acc:HGNC:5100]	-0.50188013	-0.502977764	-0.495133084	1.499990978	12
320	ENSG00000255330	C6ORF174	SOGA family member 3 [Source:HGNC Symbol;Acc:HGNC:21494]	-0.3909476	-1.071958124	0.171087277	1.291818446	12
321	ENSG00000258691	RP11-404P21.8	Uncharacterized protein {ECO:0000313|Ensembl:ENSP00000450984} [Source	-0.50939345	-0.579776459	-0.407053642	1.496223548	12
322	ENSG00000258875	CTD-2547L24.3		-0.49528412	-0.545934051	-0.457803404	1.499021578	12
323	ENSG00000266265	KLF14	Kruppel-like factor 14 [Source:HGNC Symbol;Acc:HGNC:23025]	-0.55942556	-0.588024189	-0.343594621	1.491044375	12
324	ENSG00000268089	GABRQ	gamma-aminobutyric acid (GABA) A receptor, theta [Source:HGNC Symbol;Ac	-0.80322057	-0.911207885	0.69152712	1.022901337	12
325	ENSG00000272398	CD24	CD24 molecule [Source:HGNC Symbol;Acc:HGNC:1645]	-0.53300046	-0.540499143	-0.424387671	1.497887275	12
326	ENSG00000273049	RP11-834C11.12	Uncharacterized protein {ECO:0000313|Ensembl:ENSP00000476742} [Source	-0.50885666	-0.51880987	-0.472029799	1.499696331	12
327	ENSG00000278500	HOXD3		-0.59761453	-0.738935641	-0.107498014	1.444048187	12
328	ENSG00000081248	CACNA1S	calcium channel, voltage-dependent, L type, alpha 1S subunit [Source:HGNC S	-0.5	-0.5	-0.5	1.5	12
329	ENSG00000057149	SERPINB3	serpin peptidase inhibitor, clade B (ovalbumin), member 3 [Source:HGNC Sym	-0.5	-0.5	-0.5	1.5	12
330	ENSG00000101282	RSPO4	R-spondin 4 [Source:HGNC Symbol;Acc:HGNC:16175]	-0.5	-0.5	-0.5	1.5	12
331	ENSG00000123364	HOXC13	homeobox C13 [Source:HGNC Symbol;Acc:HGNC:5125]	-0.5	-0.5	-0.5	1.5	12
332	ENSG00000123407	HOXC12	homeobox C12 [Source:HGNC Symbol;Acc:HGNC:5124]	-0.5	-0.5	-0.5	1.5	12
333	ENSG00000128710	HOXD10	homeobox D10 [Source:HGNC Symbol;Acc:HGNC:5133]	-0.5	-0.5	-0.5	1.5	12
334	ENSG00000128713	HOXD11	homeobox D11 [Source:HGNC Symbol;Acc:HGNC:5134]	-0.5	-0.5	-0.5	1.5	12
335	ENSG00000147256	ARHGAP36	Rho GTPase activating protein 36 [Source:HGNC Symbol;Acc:HGNC:26388]	-0.5	-0.5	-0.5	1.5	12
336	ENSG00000149575	SCN2B	sodium channel, voltage-gated, type II, beta subunit [Source:HGNC Symbol;Ac	-0.5	-0.5	-0.5	1.5	12
337	ENSG00000163568	AIM2	absent in melanoma 2 [Source:HGNC Symbol;Acc:HGNC:357]	-0.5	-0.5	-0.5	1.5	12
338	ENSG00000165409	TSHR	thyroid stimulating hormone receptor [Source:HGNC Symbol;Acc:HGNC:1237	-0.5	-0.5	-0.5	1.5	12
339	ENSG00000166426	CRABP1	cellular retinoic acid binding protein 1 [Source:HGNC Symbol;Acc:HGNC:2338	-0.5	-0.5	-0.5	1.5	12
340	ENSG00000171049	FPR2	formyl peptide receptor 2 [Source:HGNC Symbol;Acc:HGNC:3827]	-0.5	-0.5	-0.5	1.5	12
341	ENSG00000172482	AGXT	alanine-glyoxylate aminotransferase [Source:HGNC Symbol;Acc:HGNC:341]	-0.5	-0.5	-0.5	1.5	12
342	ENSG00000197406	DIO3	deiodinase, iodothyronine, type III [Source:HGNC Symbol;Acc:HGNC:2885]	-0.5	-0.5	-0.5	1.5	12
343	ENSG00000197587	DMBX1	diencephalon/mesencephalon homeobox 1 [Source:HGNC Symbol;Acc:HGNC	-0.5	-0.5	-0.5	1.5	12
344	ENSG00000198125	MB	myoglobin [Source:HGNC Symbol;Acc:HGNC:6915]	-0.5	-0.5	-0.5	1.5	12
345	ENSG00000205755	CRLF2	cytokine receptor-like factor 2 [Source:HGNC Symbol;Acc:HGNC:14281]	-0.5	-0.5	-0.5	1.5	12
346	ENSG00000206073	SERPINB4	serpin peptidase inhibitor, clade B (ovalbumin), member 4 [Source:HGNC Sym	-0.5	-0.5	-0.5	1.5	12
347	ENSG00000019549	SNAI2	snail family zinc finger 2 [Source:HGNC Symbol;Acc:HGNC:11094]	-0.82031299	-0.758066576	0.285810872	1.29256869	12
348	ENSG00000006283	CACNA1G	calcium channel, voltage-dependent, T type, alpha 1G subunit [Source:HGNC	-0.50654047	-0.498077173	-0.495365365	1.499983011	12
349	ENSG00000041982	TNC	tenascin C [Source:HGNC Symbol;Acc:HGNC:5318]	-0.57113929	-0.543097405	-0.380453892	1.494690588	12
350	ENSG00000044524	EPHA3	EPH receptor A3 [Source:HGNC Symbol;Acc:HGNC:3387]	-0.52561096	-0.505069661	-0.468907242	1.499587862	12
351	ENSG00000066382	MPPED2	metallophosphoesterase domain containing 2 [Source:HGNC Symbol;Acc:HGN	-0.56470555	-0.531620663	-0.39986683	1.496193045	12
352	ENSG00000069011	PITX1	paired-like homeodomain 1 [Source:HGNC Symbol;Acc:HGNC:9004]	-0.5040455	-0.503387411	-0.492546241	1.49997915	12
353	ENSG00000069702	TGFBR3	transforming growth factor, beta receptor III [Source:HGNC Symbol;Acc:HGN	-0.87623775	-0.3356665	-0.226267637	1.438171884	12
354	ENSG00000070808	CAMK2A	calcium/calmodulin-dependent protein kinase II alpha [Source:HGNC Symbol;	-0.68323648	-0.579158371	-0.205744359	1.468139213	12
355	ENSG00000073711	PPP2R3A	protein phosphatase 2, regulatory subunit B'', alpha [Source:HGNC Symbol;Ac	-0.82347178	-0.522665141	-0.082799607	1.428936528	12
356	ENSG00000091879	ANGPT2	angiopoietin 2 [Source:HGNC Symbol;Acc:HGNC:485]	-0.96418688	-0.538499337	0.180672806	1.322013412	12
357	ENSG00000099994	SUSD2	sushi domain containing 2 [Source:HGNC Symbol;Acc:HGNC:30667]	-0.54004017	-0.53909178	-0.418419876	1.497551827	12
358	ENSG00000099998	GGT5	gamma-glutamyltransferase 5 [Source:HGNC Symbol;Acc:HGNC:4260]	-0.64863413	-0.636649492	-0.178325362	1.463608983	12
359	ENSG00000100302	RASD2	RASD family, member 2 [Source:HGNC Symbol;Acc:HGNC:18229]	-0.5119391	-0.510522691	-0.47734659	1.499808379	12
360	ENSG00000100625	SIX4	SIX homeobox 4 [Source:HGNC Symbol;Acc:HGNC:10890]	-0.79740987	-0.516451646	-0.12865792	1.442519439	12
361	ENSG00000102445	KIAA0226L	KIAA0226-like [Source:HGNC Symbol;Acc:HGNC:20420]	-0.52009466	-0.501738757	-0.477943235	1.499776658	12
362	ENSG00000104722	NEFM	neurofilament, medium polypeptide [Source:HGNC Symbol;Acc:HGNC:7734]	-0.83361029	-0.562197296	-0.014447484	1.410255068	12
363	ENSG00000105251	SHD	Src homology 2 domain containing transforming protein D [Source:HGNC Sym	-0.89724569	-0.832876332	0.81707098	0.91305104	12
364	ENSG00000105509	HAS1	hyaluronan synthase 1 [Source:HGNC Symbol;Acc:HGNC:4818]	-0.50041157	-0.500130556	-0.499457758	1.49999988	12
365	ENSG00000105696	TMEM59L	transmembrane protein 59-like [Source:HGNC Symbol;Acc:HGNC:13237]	-0.5382792	-0.485145251	-0.476010105	1.499434551	12
366	ENSG00000105974	CAV1	caveolin 1, caveolae protein, 22kDa [Source:HGNC Symbol;Acc:HGNC:1527]	-0.90638017	-0.624700862	0.210509319	1.320571712	12
367	ENSG00000106511	MEOX2	mesenchyme homeobox 2 [Source:HGNC Symbol;Acc:HGNC:7014]	-0.51260755	-0.50461647	-0.482655704	1.499879725	12
368	ENSG00000107282	APBA1	amyloid beta (A4) precursor protein-binding, family A, member 1 [Source:HGI	-0.80245972	-0.627264089	0.029098427	1.400625378	12
369	ENSG00000107821	KAZALD1	Kazal-type serine peptidase inhibitor domain 1 [Source:HGNC Symbol;Acc:HG	-0.6610032	-0.557581846	-0.259529234	1.478114281	12
370	ENSG00000108001	EBF3	early B-cell factor 3 [Source:HGNC Symbol;Acc:HGNC:19087]	-0.70591148	-0.475578123	-0.297386828	1.478876433	12
371	ENSG00000109705	NKX3-2	NK3 homeobox 2 [Source:HGNC Symbol;Acc:HGNC:951]	-0.62356312	-0.590273554	-0.266593664	1.480430338	12
372	ENSG00000110436	SLC1A2	solute carrier family 1 (glial high affinity glutamate transporter), member 2 [S	-0.55239005	-0.53074501	-0.414095635	1.497230699	12
373	ENSG00000111186	WNT5B	wingless-type MMTV integration site family, member 5B [Source:HGNC Symb	-0.69251082	-0.672884449	-0.070795281	1.436190553	12
374	ENSG00000111728	ST8SIA1	ST8 alpha-N-acetyl-neuraminide alpha-2,8-sialyltransferase 1 [Source:HGNC S	-0.77061859	-0.439487413	-0.255417667	1.465523668	12
375	ENSG00000112246	SIM1	single-minded family bHLH transcription factor 1 [Source:HGNC Symbol;Acc:H	-0.80923875	-0.642642069	0.063307456	1.388573362	12
376	ENSG00000112297	AIM1	absent in melanoma 1 [Source:HGNC Symbol;Acc:HGNC:356]	-0.86619483	-0.628449811	0.139960795	1.354683847	12
377	ENSG00000113721	PDGFRB	platelet-derived growth factor receptor, beta polypeptide [Source:HGNC Sym	-0.99069096	-0.713223003	0.681394737	1.02251923	12
378	ENSG00000114251	WNT5A	wingless-type MMTV integration site family, member 5A [Source:HGNC Symb	-0.61680202	-0.503785355	-0.371880534	1.492467907	12
379	ENSG00000115648	MLPH	melanophilin [Source:HGNC Symbol;Acc:HGNC:29643]	-0.85993971	-0.849458013	0.657335635	1.05206209	12
380	ENSG00000115919	KYNU	kynureninase [Source:HGNC Symbol;Acc:HGNC:6469]	-0.61125712	-0.54553058	-0.332562305	1.489350004	12
381	ENSG00000117598	LPPR5	Lipid phosphate phosphatase-related protein type 5 [Source:UniProtKB/Swis	-0.60656237	-0.505293599	-0.381794959	1.493650924	12
382	ENSG00000118473	SGIP1	SH3-domain GRB2-like (endophilin) interacting protein 1 [Source:HGNC Symb	-0.88103013	-0.382322071	-0.168194619	1.431546824	12
383	ENSG00000121068	TBX2	T-box 2 [Source:HGNC Symbol;Acc:HGNC:11597]	-0.74088275	-0.717802784	0.069051101	1.389634433	12
384	ENSG00000121297	TSHZ3	teashirt zinc finger homeobox 3 [Source:HGNC Symbol;Acc:HGNC:30700]	-0.85743834	-0.606867297	0.090118526	1.374187114	12
385	ENSG00000121440	PDZRN3	PDZ domain containing ring finger 3 [Source:HGNC Symbol;Acc:HGNC:17704]	-0.7540533	-0.494082016	-0.215088378	1.463223692	12
386	ENSG00000122042	UBL3	ubiquitin-like 3 [Source:HGNC Symbol;Acc:HGNC:12504]	-0.71084975	-0.643735275	-0.085218871	1.439803899	12
387	ENSG00000123388	HOXC11	homeobox C11 [Source:HGNC Symbol;Acc:HGNC:5123]	-0.50193221	-0.500508408	-0.497556892	1.49999751	12
388	ENSG00000123572	NRK	Nik related kinase [Source:HGNC Symbol;Acc:HGNC:25391]	-1.02255075	-0.265899943	-0.081596457	1.370047152	12
389	ENSG00000123700	KCNJ2	potassium inwardly-rectifying channel, subfamily J, member 2 [Source:HGNC	-1.04803341	-0.662389676	0.848417708	0.86200538	12
390	ENSG00000124343	XG	Xg blood group [Source:HGNC Symbol;Acc:HGNC:12806]	-0.60097457	-0.595485935	-0.2873394	1.4837999	12
391	ENSG00000125355	TMEM255A	transmembrane protein 255A [Source:HGNC Symbol;Acc:HGNC:26086]	-0.55514565	-0.534760193	-0.406860474	1.496766315	12
392	ENSG00000126010	GRPR	gastrin-releasing peptide receptor [Source:HGNC Symbol;Acc:HGNC:4609]	-0.64403885	-0.579871297	-0.254066066	1.47797621	12
393	ENSG00000126860	EVI2A	ecotropic viral integration site 2A [Source:HGNC Symbol;Acc:HGNC:3499]	-0.70773561	-0.666083711	-0.058661601	1.432480927	12

394	ENSG00000128285	MCHR1	melanin-concentrating hormone receptor 1 [Source:HGNC Symbol;Acc:HGNC	-0.60166352	-0.468409453	-0.425709447	1.495782423	12
395	ENSG00000130592	LSP1	lymphocyte-specific protein 1 [Source:HGNC Symbol;Acc:HGNC:6707]	-0.66452314	-0.595148637	-0.210051188	1.469722965	12
396	ENSG00000131386	GALNT15	polypeptide N-acetylgalactosaminyltransferase 15 [Source:HGNC Symbol;Acc	-0.53090453	-0.522086512	-0.445916684	1.498907724	12
397	ENSG00000132854	KANK4	KN motif and ankyrin repeat domains 4 [Source:HGNC Symbol;Acc:HGNC:272	-0.51404813	-0.511530873	-0.474171631	1.499750631	12
398	ENSG00000133019	CHRM3	cholinergic receptor, muscarinic 3 [Source:HGNC Symbol;Acc:HGNC:1952]	-0.5730038	-0.466314549	-0.458636814	1.49795516	12
399	ENSG00000133055	MYBPH	myosin binding protein H [Source:HGNC Symbol;Acc:HGNC:7552]	-0.58813718	-0.570228695	-0.331319158	1.489685036	12
400	ENSG00000133105	RXFP2	relaxin/insulin-like family peptide receptor 2 [Source:HGNC Symbol;Acc:HGN(	-0.5146387	-0.506355527	-0.478830053	1.49982428	12
401	ENSG00000133636	NTS	neurotensin [Source:HGNC Symbol;Acc:HGNC:8038]	-0.60353576	-0.54876163	-0.337829048	1.490126436	12
402	ENSG00000134207	SYT6	synaptotagmin VI [Source:HGNC Symbol;Acc:HGNC:18638]	-0.54594499	-0.531363481	-0.42032964	1.497638111	12
403	ENSG00000134242	PTPN22	protein tyrosine phosphatase, non-receptor type 22 (lymphoid) [Source:HGN(	-0.54422528	-0.530380434	-0.423198757	1.497804476	12
404	ENSG00000135905	DOCK10	dedicator of cytokinesis 10 [Source:HGNC Symbol;Acc:HGNC:23479]	-0.70535271	-0.676751219	-0.046979097	1.429083029	12
405	ENSG00000136866	ZFP37	ZFP37 zinc finger protein [Source:HGNC Symbol;Acc:HGNC:12863]	-0.99720817	-0.280249848	-0.1066187	1.384076714	12
406	ENSG00000137449	CPEB2	cytoplasmic polyadenylation element binding protein 2 [Source:HGNC Symbo	-1.03669715	-0.401213955	0.114599616	1.323311492	12
407	ENSG00000137501	SYTL2	synaptotagmin-like 2 [Source:HGNC Symbol;Acc:HGNC:15585]	-0.60206215	-0.553658081	-0.334044287	1.489764516	12
408	ENSG00000137507	LRRC32	leucine rich repeat containing 32 [Source:HGNC Symbol;Acc:HGNC:4161]	-0.7574672	-0.634023646	-0.030936795	1.422427636	12
409	ENSG00000138134	STAMBPL1	STAM binding protein-like 1 [Source:HGNC Symbol;Acc:HGNC:24105]	-0.598894	-0.556755453	-0.334202282	1.489851731	12
410	ENSG00000138347	MYPN	myopalladin [Source:HGNC Symbol;Acc:HGNC:23246]	-0.73085691	-0.66745535	-0.02289406	1.421206318	12
411	ENSG00000138395	CDK15	cyclin-dependent kinase 15 [Source:HGNC Symbol;Acc:HGNC:14434]	-0.85286875	-0.85181118	0.635469285	1.069210644	12
412	ENSG00000139263	LRIG3	leucine-rich repeats and immunoglobulin-like domains 3 [Source:HGNC Symb	-1.26899114	-0.297371289	0.590196535	0.976165894	12
413	ENSG00000141314	RHBDL3	rhomboid, veinlet-like 3 (Drosophila) [Source:HGNC Symbol;Acc:HGNC:16502	-0.64751308	-0.612793355	-0.209794689	1.470101121	12
414	ENSG00000142661	MYOM3	myomesin 3 [Source:HGNC Symbol;Acc:HGNC:26679]	-0.7158058	-0.645157811	-0.076248815	1.43721243	12
415	ENSG00000143013	LMO4	LIM domain only 4 [Source:HGNC Symbol;Acc:HGNC:6644]	-0.52308721	-0.513380962	-0.463011708	1.499479882	12
416	ENSG00000143816	WNT9A	wingless-type MMTV integration site family, member 9A [Source:HGNC Symb	-0.82449181	-0.801512223	0.39119232	1.234811716	12
417	ENSG00000144369	FAM171B	family with sequence similarity 171, member B [Source:HGNC Symbol;Acc:HG	-0.89686226	-0.483663328	-0.019942259	1.400467844	12
418	ENSG00000144891	AGTR1	angiotensin II receptor, type 1 [Source:HGNC Symbol;Acc:HGNC:336]	-0.76625147	-0.761240445	0.186357044	1.341134866	12
419	ENSG00000145423	SFRP2	secreted frizzled-related protein 2 [Source:HGNC Symbol;Acc:HGNC:10777]	-0.5006644	-0.50059358	-0.498741427	1.499999406	12
420	ENSG00000146197	SCUBE3	signal peptide, CUB domain, EGF-like 3 [Source:HGNC Symbol;Acc:HGNC:1365	-0.68218627	-0.667024536	-0.093351389	1.442562193	12
421	ENSG00000148344	PTGES	prostaglandin E synthase [Source:HGNC Symbol;Acc:HGNC:9599]	-0.52116656	-0.518362162	-0.459872329	1.499401055	12
422	ENSG00000148926	ADM	adrenomedullin [Source:HGNC Symbol;Acc:HGNC:259]	-0.61784646	-0.47369423	-0.402425039	1.493965733	12
423	ENSG00000150594	ADRA2A	adrenoceptor alpha 2A [Source:HGNC Symbol;Acc:HGNC:281]	-0.68925551	-0.462141239	-0.332182424	1.483579171	12
424	ENSG00000151632	AKR1C2	aldo-keto reductase family 1, member C2 [Source:HGNC Symbol;Acc:HGNC:3ŧ	-0.64942847	-0.447065571	-0.394415546	1.490909591	12
425	ENSG00000152784	PRDM8	PR domain containing 8 [Source:HGNC Symbol;Acc:HGNC:13993]	-0.79953668	-0.748250564	0.22478547	1.323001771	12
426	ENSG00000153404	PLEKHG4B	pleckstrin homology domain containing, family G (with RhoGef domain) mem	-0.5907089	-0.588435543	-0.307538642	1.486683084	12
427	ENSG00000156427	FGF18	fibroblast growth factor 18 [Source:HGNC Symbol;Acc:HGNC:3674]	-0.50655289	-0.502422641	-0.490991977	1.499967511	12
428	ENSG00000159200	RCAN1	regulator of calcineurin 1 [Source:HGNC Symbol;Acc:HGNC:3040]	-0.6926148	-0.401770825	-0.390896998	1.485282625	12
429	ENSG00000159251	ACTC1	actin, alpha, cardiac muscle 1 [Source:HGNC Symbol;Acc:HGNC:143]	-0.69919856	-0.674817637	-0.058572975	1.432589172	12
430	ENSG00000160886	LY6K	lymphocyte antigen 6 complex, locus K [Source:HGNC Symbol;Acc:HGNC:242	-0.71226559	-0.644070497	-0.082761367	1.439097454	12
431	ENSG00000161544	CYGB	cytoglobin [Source:HGNC Symbol;Acc:HGNC:16505]	-0.88619903	-0.701149451	0.312424246	1.274924238	12
432	ENSG00000162745	OLFML2B	olfactomedin-like 2B [Source:HGNC Symbol;Acc:HGNC:24558]	-0.5944078	-0.586591574	-0.305382543	1.486381915	12
433	ENSG00000163359	COL6A3	collagen, type VI, alpha 3 [Source:HGNC Symbol;Acc:HGNC:2213]	-0.60053564	-0.58825985	-0.296301402	1.485096891	12
434	ENSG00000164220	F2RL2	coagulation factor II (thrombin) receptor-like 2 [Source:HGNC Symbol;Acc:HG	-0.66374066	-0.629522152	-0.167888656	1.461151464	12
435	ENSG00000164604	GPR85	G protein-coupled receptor 85 [Source:HGNC Symbol;Acc:HGNC:4536]	-0.87431588	-0.8418976	0.693659442	1.022554036	12
436	ENSG00000164929	BAALC	brain and acute leukemia, cytoplasmic [Source:HGNC Symbol;Acc:HGNC:1433	-0.57586471	-0.568786645	-0.346844667	1.491496017	12
437	ENSG00000165757	KIAA1462	KIAA1462 [Source:HGNC Symbol;Acc:HGNC:29283]	-0.6604635	-0.584702136	-0.227914876	1.47308051	12
438	ENSG00000166897	ELFN2	extracellular leucine-rich repeat and fibronectin type III domain containing 2 [	-0.50825181	-0.499398478	-0.492317844	1.499968132	12
439	ENSG00000168229	PTGDR	prostaglandin D2 receptor (DP) [Source:HGNC Symbol;Acc:HGNC:9591]	-0.63345617	-0.554780431	-0.296106061	1.484342666	12
440	ENSG00000168621	GDNF	glial cell derived neurotrophic factor [Source:HGNC Symbol;Acc:HGNC:4232]	-0.68406867	-0.671019538	-0.085230697	1.440318909	12
441	ENSG00000168675	LDLRAD4	low density lipoprotein receptor class A domain containing 4 [Source:HGNC S	-0.64870656	-0.618957474	-0.200609527	1.468273564	12
442	ENSG00000169126	ARMC4	armadillo repeat containing 4 [Source:HGNC Symbol;Acc:HGNC:25583]	-0.67869914	-0.677913162	-0.08314075	1.439753049	12
443	ENSG00000169744	LDB2	LIM domain binding 2 [Source:HGNC Symbol;Acc:HGNC:6533]	-0.98567248	-0.309371461	-0.09212986	1.3871738	12
444	ENSG00000169851	PCDH7	protocadherin 7 [Source:HGNC Symbol;Acc:HGNC:8659]	-0.83300671	-0.751479177	0.299251512	1.285234375	12
445	ENSG00000169862	CTNND2	catenin (cadherin-associated protein), delta 2 [Source:HGNC Symbol;Acc:HGN	-0.60914854	-0.598523718	-0.274079382	1.481751636	12
446	ENSG00000170577	SIX2	SIX homeobox 2 [Source:HGNC Symbol;Acc:HGNC:10888]	-0.82392042	-0.453212486	-0.167727339	1.444860244	12
447	ENSG00000171408	PDE7B	phosphodiesterase 7B [Source:HGNC Symbol;Acc:HGNC:8792]	-0.87261643	-0.720136637	0.321238322	1.271514741	12
448	ENSG00000171604	CXXC5	CXXC finger protein 5 [Source:HGNC Symbol;Acc:HGNC:26943]	-0.93582472	-0.409022269	-0.053050886	1.397897877	12
449	ENSG00000172554	SNTG2	syntrophin, gamma 2 [Source:HGNC Symbol;Acc:HGNC:13741]	-0.66478655	-0.557812393	-0.254590271	1.477189213	12
450	ENSG00000173210	ABLIM3	actin binding LIM protein family, member 3 [Source:HGNC Symbol;Acc:HGNC:	-0.86910996	-0.861213099	0.810613619	0.919709435	12
451	ENSG00000174482	LINGO2	leucine rich repeat and Ig domain containing 2 [Source:HGNC Symbol;Acc:HG	-0.69742557	-0.602583812	-0.157917998	1.457927376	12
452	ENSG00000174669	SLC29A2	solute carrier family 29 (equilibrative nucleoside transporter), member 2 [Sou	-0.75314849	-0.61405371	-0.065092586	1.432294785	12
453	ENSG00000175084	DES	desmin [Source:HGNC Symbol;Acc:HGNC:2770]	-0.56355611	-0.495297831	-0.439205764	1.498059702	12
454	ENSG00000176887	SOX11	SRY (sex determining region Y)-box 11 [Source:HGNC Symbol;Acc:HGNC:1119	-0.77674543	-0.707406432	0.111643814	1.372508047	12
455	ENSG00000177363	LRRN4CL	LRRN4 C-terminal like [Source:HGNC Symbol;Acc:HGNC:33724]	-0.55225888	-0.514367716	-0.431463839	1.498090432	12
456	ENSG00000178031	ADAMTSL1	ADAMTS-like 1 [Source:HGNC Symbol;Acc:HGNC:14632]	-0.92467181	-0.804473638	0.826941413	0.902204031	12
457	ENSG00000179399	GPC5	glypican 5 [Source:HGNC Symbol;Acc:HGNC:4453]	-0.5557494	-0.531793586	-0.409371553	1.496914534	12
458	ENSG00000180818	HOXC10	homeobox C10 [Source:HGNC Symbol;Acc:HGNC:5122]	-0.50249382	-0.50191786	-0.495580965	1.499992644	12
459	ENSG00000181541	MAB21L2	mab-21-like 2 (C. elegans) [Source:HGNC Symbol;Acc:HGNC:6758]	-0.83756133	-0.512921286	-0.0742589	1.424741516	12
460	ENSG00000181634	TNFSF15	tumor necrosis factor (ligand) superfamily, member 15 [Source:HGNC Symbol	-0.66820244	-0.496202706	-0.320396095	1.484801239	12
461	ENSG00000182175	RGMA	repulsive guidance molecule family member a [Source:HGNC Symbol;Acc:HGI	-0.80395214	-0.768584228	0.273187337	1.299349035	12
462	ENSG00000182463	TSHZ2	teashirt zinc finger homeobox 2 [Source:HGNC Symbol;Acc:HGNC:13010]	-0.5204099	-0.514798648	-0.464314132	1.499522682	12
463	ENSG00000183160	TMEM119	transmembrane protein 119 [Source:HGNC Symbol;Acc:HGNC:27884]	-0.57881639	-0.577100917	-0.334141594	1.490058897	12
464	ENSG00000183778	B3GALT5	UDP-Gal:betaGlcNAc beta 1,3-galactosyltransferase, polypeptide 5 [Source:H(	-0.83802948	-0.707687775	0.224103819	1.321613438	12
465	ENSG00000183780	SLC35F3	solute carrier family 35, member F3 [Source:HGNC Symbol;Acc:HGNC:23616]	-0.5224271	-0.513773348	-0.463289398	1.499489848	12
466	ENSG00000185269	NOTUM	notum pectinacetylesterase homolog (Drosophila) [Source:HGNC Symbol;Acc	-0.53930964	-0.488872837	-0.471192694	1.499375172	12
467	ENSG00000185306	C12orf56	chromosome 12 open reading frame 56 [Source:HGNC Symbol;Acc:HGNC:269	-0.70759847	-0.625162355	-0.114756813	1.447517637	12
468	ENSG00000185739	SRL	sarcalumenin [Source:HGNC Symbol;Acc:HGNC:11295]	-0.57060582	-0.570391774	-0.35094693	1.491944527	12
469	ENSG00000185924	RTN4RL1	reticulon 4 receptor-like 1 [Source:HGNC Symbol;Acc:HGNC:21329]	-0.57817533	-0.517014657	-0.400742654	1.495932639	12
470	ENSG00000186480	INSIG1	insulin induced gene 1 [Source:HGNC Symbol;Acc:HGNC:6083]	-0.62805356	-0.578112535	-0.275528448	1.481694538	12
471	ENSG00000187546	AGMO	alkylglycerol monooxygenase [Source:HGNC Symbol;Acc:HGNC:33784]	-0.55714843	-0.50798591	-0.432906905	1.498041241	12
472	ENSG00000187699	C2orf88	chromosome 2 open reading frame 88 [Source:HGNC Symbol;Acc:HGNC:2819	-0.55589166	-0.552448422	-0.386993154	1.49533324	12
473	ENSG00000187867	PALM3	paralemmin 3 [Source:HGNC Symbol;Acc:HGNC:33274]	-0.69045623	-0.499361101	-0.289985693	1.479803022	12
474	ENSG00000189184	PCDH18	protocadherin 18 [Source:HGNC Symbol;Acc:HGNC:14268]	-1.08738953	-0.402951553	0.223320103	1.267020985	12
475	ENSG00000196557	CACNA1H	calcium channel, voltage-dependent, T type, alpha 1H subunit [Source:HGNC	-0.8618158	-0.84445107	0.642773489	1.063493378	12
476	ENSG00000197594	ENPP1	ectonucleotide pyrophosphatase/phosphodiesterase 1 [Source:HGNC Symbol	-0.68050488	-0.660336827	-0.104801745	1.445643449	12
477	ENSG00000198743	SLC5A3	solute carrier family 5 (sodium/myo-inositol cotransporter), member 3 [Sourc	-1.02600059	-0.5345528	0.309154282	1.251399107	12

478	ENSG00000198768	APCDD1L	adenomatosis polyposis coli down-regulated 1-like [Source:HGNC Symbol;Acc	-1.04841436	-0.593181746	0.5033521	1.138244004	12
479	ENSG00000198795	ZNF521	zinc finger protein 521 [Source:HGNC Symbol;Acc:HGNC:24605]	-0.7004124	-0.685618146	-0.041426656	1.427457201	12
480	ENSG00000205336	GPR56	G protein-coupled receptor 56 [Source:HGNC Symbol;Acc:HGNC:4512]	-0.86333934	-0.796119135	0.480488423	1.178970051	12
481	ENSG00000206190	ATP10A	ATPase, class V, type 10A [Source:HGNC Symbol;Acc:HGNC:13542]	-0.75903292	-0.630706643	-0.033275167	1.42301473	12
482	ENSG00000216490	IFI30	interferon, gamma-inducible protein 30 [Source:HGNC Symbol;Acc:HGNC:539	-0.60675948	-0.446800995	-0.442037849	1.495598323	12
483	ENSG00000221818	EBF2	early B-cell factor 2 [Source:HGNC Symbol;Acc:HGNC:19090]	-0.57127765	-0.533964168	-0.39017928	1.495421094	12
484	ENSG00000221852	KRTAP1-5	keratin associated protein 1-5 [Source:HGNC Symbol;Acc:HGNC:16777]	-0.58922284	-0.565541844	-0.335371223	1.490135904	12
485	ENSG00000221866	PLXNA4	plexin A4 [Source:HGNC Symbol;Acc:HGNC:9102]	-0.78008266	-0.490398983	-0.184490258	1.454971899	12
486	ENSG00000245848	CEBPA	CCAAT/enhancer binding protein (C/EBP), alpha [Source:HGNC Symbol;Acc:H	-0.50996353	-0.496301414	-0.493696884	1.499961829	12
487	ENSG00000248905	FMN1	formin 1 [Source:HGNC Symbol;Acc:HGNC:3768]	-0.70703608	-0.605446271	-0.141326951	1.453809302	12
488	ENSG00000256235	SMIM3	small integral membrane protein 3 [Source:HGNC Symbol;Acc:HGNC:30248]	-0.62030317	-0.575514069	-0.287816531	1.483633773	12
489	ENSG00000262003	RP11-676J12.7		-0.61128882	-0.590559404	-0.280933582	1.482781808	12
490	ENSG00000265118	CTD-2370N5.3		-0.67560886	-0.656591614	-0.116543055	1.448743535	12
491	ENSG00000277586	NEFL	neurofilament, light polypeptide [Source:HGNC Symbol;Acc:HGNC:7739]	-0.77142096	-0.730427169	0.14116512	1.360683012	12
492	ENSG00000137252	HCRTR2	hypocretin (orexin) receptor 2 [Source:HGNC Symbol;Acc:HGNC:4849]	-0.5560065	-0.382989526	-0.556006502	1.495002529	13
493	ENSG00000089250	NOS1	nitric oxide synthase 1 (neuronal) [Source:HGNC Symbol;Acc:HGNC:7872]	-0.50346003	-0.493061914	-0.503460033	1.49998198	13
494	ENSG00000171551	ECEL1	endothelin converting enzyme-like 1 [Source:HGNC Symbol;Acc:HGNC:3147]	-0.53187878	-0.43466658	-0.531878779	1.498424137	13
495	ENSG00000185610	DBX2	developing brain homeobox 2 [Source:HGNC Symbol;Acc:HGNC:33186]	-0.55024196	-0.395521003	-0.550241957	1.496004918	13
496	ENSG00000052850	ALX4	ALX homeobox 4 [Source:HGNC Symbol;Acc:HGNC:450]	-0.49982147	-0.491646347	-0.508496684	1.499964497	13
497	ENSG00000007062	PROM1	prominin 1 [Source:HGNC Symbol;Acc:HGNC:9454]	-0.50043599	-0.489660444	-0.509852527	1.499948957	13
498	ENSG00000056998	GYG2	glycogenin 2 [Source:HGNC Symbol;Acc:HGNC:4700]	-0.49775046	-0.305125049	-0.679496711	1.482372221	13
499	ENSG00000062038	CDH3	cadherin 3, type 1, P-cadherin (placental) [Source:HGNC Symbol;Acc:HGNC:1	0.01269301	0.181491998	-1.311185917	1.11700091	13
500	ENSG00000085117	CD82	CD82 molecule [Source:HGNC Symbol;Acc:HGNC:6210]	-0.4725043	-0.40121691	-0.620038228	1.493759442	13
501	ENSG00000091622	PITPNM3	PITPNM family member 3 [Source:HGNC Symbol;Acc:HGNC:21043]	-0.49160828	-0.08270509	-0.85006872	1.42438209	13
502	ENSG00000099282	TSPAN15	tetraspanin 15 [Source:HGNC Symbol;Acc:HGNC:23298]	0.220483547	0.330974793	-1.435425152	0.883966812	13
503	ENSG00000100985	MMP9	matrix metallopeptidase 9 (gelatinase B, 92kDa gelatinase, 92kDa type IV coll	-0.49995213	-0.499906541	-0.500141326	1.499999992	13
504	ENSG00000105642	KCNN1	potassium intermediate/small conductance calcium-activated channel, subfa	-0.49237547	-0.175107622	-0.785217092	1.452700187	13
505	ENSG00000106066	CPVL	carboxypeptidase, vitellogenic-like [Source:HGNC Symbol;Acc:HGNC:14399]	-0.42125205	-0.390920506	-0.675559629	1.487732185	13
506	ENSG00000108176	DNAJC12	DnaJ (Hsp40) homolog, subfamily C, member 12 [Source:HGNC Symbol;Acc:H	-0.43431732	-0.416969874	-0.640930274	1.492217468	13
507	ENSG00000110328	GALNT18	polypeptide N-acetylgalactosaminyltransferase 18 [Source:HGNC Symbol;Acc	0.178692917	0.267937358	-1.405799622	0.959169347	13
508	ENSG00000120645	IQSEC3	IQ motif and Sec7 domain 3 [Source:HGNC Symbol;Acc:HGNC:29193]	-0.54529892	-0.344823721	-0.600784402	1.490907038	13
509	ENSG00000121570	DPPA4	developmental pluripotency associated 4 [Source:HGNC Symbol;Acc:HGNC:1	-0.43957509	-0.248066957	-0.776181373	1.463823419	13
510	ENSG00000122877	EGR2	early growth response 2 [Source:HGNC Symbol;Acc:HGNC:3239]	-0.41881257	-0.22943105	-0.807657217	1.455900836	13
511	ENSG00000123500	COL10A1	collagen, type X, alpha 1 [Source:HGNC Symbol;Acc:HGNC:2185]	-0.45551072	-0.455417681	-0.586219398	1.4971478	13
512	ENSG00000124493	GRM4	glutamate receptor, metabotropic 4 [Source:HGNC Symbol;Acc:HGNC:4596]	-0.53359565	-0.30778518	-0.643871258	1.485252089	13
513	ENSG00000125657	TNFSF9	tumor necrosis factor (ligand) superfamily, member 9 [Source:HGNC Symbol;	-0.31239258	-0.275129015	-0.85822099	1.445742588	13
514	ENSG00000127241	MASP1	mannan-binding lectin serine peptidase 1 (C4/C2 activating component of Ra	-0.45973166	-0.267773661	-0.743566637	1.471071963	13
515	ENSG00000127903	ZNF835	zinc finger protein 835 [Source:HGNC Symbol;Acc:HGNC:34332]	-0.42184715	0.345106548	-1.123525107	1.200265714	13
516	ENSG00000129965	INS-IGF2	INS-IGF2 readthrough [Source:HGNC Symbol;Acc:HGNC:33527]	-0.52924317	0.297613424	-1.025347198	1.256976945	13
517	ENSG00000132205	EMILIN2	elastin microfibril interfacer 2 [Source:HGNC Symbol;Acc:HGNC:19881]	-0.4471395	-0.413171435	-0.632691697	1.493002629	13
518	ENSG00000133106	EPSTI1	epithelial stromal interaction 1 (breast) [Source:HGNC Symbol;Acc:HGNC:164	-0.4338166	-0.188896434	-0.824917114	1.447630149	13
519	ENSG00000137142	IGFBPL1	insulin-like growth factor binding protein-like 1 [Source:HGNC Symbol;Acc:HG	-0.50622613	0.801395813	-1.161965757	0.866796074	13
520	ENSG00000137869	CYP19A1	cytochrome P450, family 19, subfamily A, polypeptide 1 [Source:HGNC Symbo	-0.18831857	-0.143301214	-1.036066776	1.367686556	13
521	ENSG00000137959	IFI44L	interferon-induced protein 44-like [Source:HGNC Symbol;Acc:HGNC:17817]	-0.59923294	-0.233308434	-0.642011998	1.474553373	13
522	ENSG00000138316	ADAMTS14	ADAM metallopeptidase with thrombospondin type 1 motif, 14 [Source:HGN	-0.47119167	-0.463765283	-0.56349871	1.498455664	13
523	ENSG00000139044	B4GALNT3	beta-1,4-N-acetyl-galactosaminyl transferase 3 [Source:HGNC Symbol;Acc:HG	-0.18041987	0.31725601	-1.263707831	1.126871693	13
524	ENSG00000139626	ITGB7	integrin, beta 7 [Source:HGNC Symbol;Acc:HGNC:6162]	-0.45982419	-0.406295845	-0.627223773	1.493343811	13
525	ENSG00000140285	FGF7	fibroblast growth factor 7 [Source:HGNC Symbol;Acc:HGNC:3685]	-0.46153947	-0.275648669	-0.735778142	1.472966278	13
526	ENSG00000141574	SECTM1	secreted and transmembrane 1 [Source:HGNC Symbol;Acc:HGNC:10707]	-0.55440998	-0.365066653	-0.573856688	1.493333323	13
527	ENSG00000143333	RGS16	regulator of G-protein signaling 16 [Source:HGNC Symbol;Acc:HGNC:9997]	-0.47073258	-0.456264374	-0.571047796	1.49804475	13
528	ENSG00000144668	ITGA9	integrin, alpha 9 [Source:HGNC Symbol;Acc:HGNC:6145]	-0.54230031	-0.364421118	-0.586359377	1.493080808	13
529	ENSG00000145824	CXCL14	chemokine (C-X-C motif) ligand 14 [Source:HGNC Symbol;Acc:HGNC:10640]	-0.50095524	-0.4781386	-0.520679529	1.499773368	13
530	ENSG00000147234	FRMPD3	FERM and PDZ domain containing 3 [Source:HGNC Symbol;Acc:HGNC:29382]	-0.56537775	-0.207608119	-0.694824153	1.467810023	13
531	ENSG00000150540	HNMT	histamine N-methyltransferase [Source:HGNC Symbol;Acc:HGNC:5028]	-0.50311436	-0.283490886	-0.692315851	1.478921099	13
532	ENSG00000154856	APCDD1	adenomatosis polyposis coli down-regulated 1 [Source:HGNC Symbol;Acc:HG	-0.66457518	0.197817336	-0.864409009	1.331166855	13
533	ENSG00000157168	NRG1	neuregulin 1 [Source:HGNC Symbol;Acc:HGNC:7997]	-0.48726697	0.090052758	-0.958019848	1.355234064	13
534	ENSG00000157303	SUSD3	sushi domain containing 3 [Source:HGNC Symbol;Acc:HGNC:28391]	-0.49161369	-0.237499888	-0.739097701	1.468211282	13
535	ENSG00000157542	KCNJ6	potassium inwardly-rectifying channel, subfamily J, member 6 [Source:HGNC	-0.50001471	-0.495022239	-0.50495073	1.499987678	13
536	ENSG00000160161	CILP2	cartilage intermediate layer protein 2 [Source:HGNC Symbol;Acc:HGNC:2421	-0.31120694	-0.231295924	-0.891180882	1.433683742	13
537	ENSG00000162105	SHANK2	SH3 and multiple ankyrin repeat domains 2 [Source:HGNC Symbol;Acc:HGNC:	-0.46301854	-0.446615835	-0.587399383	1.497033763	13
538	ENSG00000162551	ALPL	alkaline phosphatase, liver/bone/kidney [Source:HGNC Symbol;Acc:HGNC:43	-0.50001095	-0.495667875	-0.504311836	1.49999066	13
539	ENSG00000163083	INHBB	inhibin, beta B [Source:HGNC Symbol;Acc:HGNC:6067]	-0.54128308	-0.414059946	-0.541942832	1.497285862	13
540	ENSG00000163531	NFASC	neurofascin [Source:HGNC Symbol;Acc:HGNC:29866]	-0.50465329	0.250872157	-1.024236224	1.27801736	13
541	ENSG00000164742	ADCY1	adenylate cyclase 1 (brain) [Source:HGNC Symbol;Acc:HGNC:232]	-0.47166879	-0.467580892	-0.559404239	1.498653917	13
542	ENSG00000165731	RET	ret proto-oncogene [Source:HGNC Symbol;Acc:HGNC:9967]	-0.49301921	-0.316816752	-0.674119106	1.483955066	13
543	ENSG00000167244	IGF2	insulin-like growth factor 2 [Source:HGNC Symbol;Acc:HGNC:5466]	-0.51213307	0.052529981	-0.91678967	1.376392757	13
544	ENSG00000168334	XIRP1	xin actin-binding repeat containing 1 [Source:HGNC Symbol;Acc:HGNC:14301	-0.52916991	-0.237931951	-0.704829735	1.4719316	13
545	ENSG00000172216	CEBPB	CCAAT/enhancer binding protein (C/EBP), beta [Source:HGNC Symbol;Acc:HG	-0.43620387	-0.406919052	-0.648184992	1.491307912	13
546	ENSG00000172575	RASGRP1	RAS guanyl releasing protein 1 (calcium and DAG-regulated) [Source:HGNC Sy	-0.41037956	0.03425464	-0.986603503	1.362728426	13
547	ENSG00000180044	C3orf80	chromosome 3 open reading frame 80 [Source:HGNC Symbol;Acc:HGNC:4004	-0.50098402	-0.452239841	-0.545683588	1.498907454	13
548	ENSG00000180447	GAS1	growth arrest-specific 1 [Source:HGNC Symbol;Acc:HGNC:4165]	-0.5276581	-0.322662087	-0.636900143	1.487220331	13
549	ENSG00000183486	MX2	MX dynamin-like GTPase 2 [Source:HGNC Symbol;Acc:HGNC:7533]	-0.64789909	0.809833555	-1.058536924	0.896602457	13
550	ENSG00000183570	PCBP3	poly(rC) binding protein 3 [Source:HGNC Symbol;Acc:HGNC:8651]	-0.44457731	-0.263746512	-0.759823609	1.46814743	13
551	ENSG00000184922	FMNL1	formin-like 1 [Source:HGNC Symbol;Acc:HGNC:1212]	-0.58730797	0.10112358	-0.880886397	1.367070784	13
552	ENSG00000185070	FLRT2	fibronectin leucine rich transmembrane protein 2 [Source:HGNC Symbol;Acc:	-0.50793386	-0.373062116	-0.611821089	1.492817069	13
553	ENSG00000187094	CCK	cholecystokinin [Source:HGNC Symbol;Acc:HGNC:1569]	-0.48850702	-0.485844518	-0.525403989	1.499755528	13
554	ENSG00000188517	COL25A1	collagen, type XXV, alpha 1 [Source:HGNC Symbol;Acc:HGNC:18603]	-0.3917899	0.064846456	-1.017385935	1.344329377	13
555	ENSG00000188573	FBLL1	fibrillarin-like 1 [Source:HGNC Symbol;Acc:HGNC:35458]	-0.56580776	0.022140687	-0.853240782	1.39690785	13
556	ENSG00000197134	ZNF257	zinc finger protein 257 [Source:HGNC Symbol;Acc:HGNC:13498]	-0.39289092	0.044709079	-1.005532433	1.353714278	13
557	ENSG00000198300	PEG3	paternally expressed 3 [Source:HGNC Symbol;Acc:HGNC:8826]	-0.31904226	0.218186369	-1.142367257	1.243223148	13
558	ENSG00000198354	DCAF12L2	DDB1 and CUL4 associated factor 12-like 2 [Source:HGNC Symbol;Acc:HGNC:3	-0.49834907	-0.467264457	-0.53383167	1.499445192	13
559	ENSG00000211445	GPX3	glutathione peroxidase 3 (plasma) [Source:HGNC Symbol;Acc:HGNC:4555]	-0.3291629	-0.133348998	-0.944947944	1.407459844	13
560	ENSG00000234719	RP11-166B2.1	Putative NPIP-like protein LOC729978 [Source:UniProtKB/Swiss-Prot;Acc:A6N	-0.4449013	-0.286417022	-0.7417285	1.473046826	13
561	ENSG00000257335	MGAM	maltase-glucoamylase (alpha-glucosidase) [Source:HGNC Symbol;Acc:HGNC:7	-0.49511387	-0.478289363	-0.526300014	1.499703249	13

562	ENSG00000259803	SLC22A31	solute carrier family 22, member 31 [Source:HGNC Symbol;Acc:HGNC:27091]	-0.52630462	-0.376761364	-0.590889722	1.493955708	13
563	ENSG00000269699	ZIM2	zinc finger, imprinted 2 [Source:HGNC Symbol;Acc:HGNC:12875]	-0.20396773	0.223557365	-1.215661188	1.196071548	13
564	ENSG00000064655	EYA2	EYA transcriptional coactivator and phosphatase 2 [Source:HGNC Symbol;Acc	-0.52944446	-0.479883212	-0.490331015	1.499658683	13
565	ENSG00000041353	RAB27B	RAB27B, member RAS oncogene family [Source:HGNC Symbol;Acc:HGNC:976	-0.53521582	-0.445012316	-0.518618913	1.498847052	13
566	ENSG00000077274	CAPN6	calpain 6 [Source:HGNC Symbol;Acc:HGNC:1483]	-0.50255324	-0.497569796	-0.499873859	1.49999689	13
567	ENSG00000079215	SLC1A3	solute carrier family 1 (glial high affinity glutamate transporter), member 3 [S	-0.53486194	-0.429005968	-0.53427368	1.498141588	13
568	ENSG00000082293	COL19A1	collagen, type XIX, alpha 1 [Source:HGNC Symbol;Acc:HGNC:2196]	-0.69900033	-0.373745899	-0.411340006	1.484086232	13
569	ENSG00000085741	WNT11	wingless-type MMTV integration site family, member 11 [Source:HGNC Symb	-0.56830158	-0.418062657	-0.510760037	1.497124279	13
570	ENSG00000092068	SLC7A8	solute carrier family 7 (amino acid transporter light chain, L system), member	-0.50746813	-0.493509176	-0.49899797	1.499975273	13
571	ENSG00000095713	CRTAC1	cartilage acidic protein 1 [Source:HGNC Symbol;Acc:HGNC:14882]	-0.58234135	-0.419348325	-0.494980059	1.49666973	13
572	ENSG00000103742	IGDCC4	immunoglobulin superfamily, DCC subclass, member 4 [Source:HGNC Symbol	-0.5361692	-0.435728503	-0.526565517	1.498463219	13
573	ENSG00000109063	MYH3	myosin, heavy chain 3, skeletal muscle, embryonic [Source:HGNC Symbol;Acc	-0.51904345	-0.463308357	-0.517147389	1.499499197	13
574	ENSG00000113296	THBS4	thrombospondin 4 [Source:HGNC Symbol;Acc:HGNC:11788]	-0.50133415	-0.499041232	-0.499623909	1.49999929	13
575	ENSG00000114423	CBLB	Cbl proto-oncogene B, E3 ubiquitin protein ligase [Source:HGNC Symbol;Acc:H	-0.66020688	-0.278734348	-0.541872237	1.480813465	13
576	ENSG00000134326	CMPK2	cytidine monophosphate (UMP-CMP) kinase 2, mitochondrial [Source:HGNC S	-1.30502769	0.570846989	-0.225162043	0.959342743	13
577	ENSG00000141576	RNF157	ring finger protein 157 [Source:HGNC Symbol;Acc:HGNC:29402]	-0.55143934	-0.441252275	-0.505775136	1.498466749	13
578	ENSG00000156113	KCNMA1	potassium large conductance calcium-activated channel, subfamily M, alpha r	-0.66712131	-0.387025275	-0.434578433	1.488725018	13
579	ENSG00000158246	FAM46B	family with sequence similarity 46, member B [Source:HGNC Symbol;Acc:HGN	-0.63018838	-0.342545384	-0.516733261	1.489467021	13
580	ENSG00000161381	PLXDC1	plexin domain containing 1 [Source:HGNC Symbol;Acc:HGNC:20945]	-0.51603807	-0.479400413	-0.504386312	1.499824792	13
581	ENSG00000163673	DCLK3	doublecortin-like kinase 3 [Source:HGNC Symbol;Acc:HGNC:19005]	-0.50292414	-0.496084745	-0.5009849	1.499993788	13
582	ENSG00000163737	PF4	platelet factor 4 [Source:HGNC Symbol;Acc:HGNC:8861]	-0.51352643	-0.474542581	-0.511689057	1.499758066	13
583	ENSG00000164440	TXLNB	taxilin beta [Source:HGNC Symbol;Acc:HGNC:21617]	-0.90968227	-0.060556452	-0.436327735	1.406566455	13
584	ENSG00000171502	COL24A1	collagen, type XXIV, alpha 1 [Source:HGNC Symbol;Acc:HGNC:20821]	-0.53313819	-0.443623636	-0.522047189	1.498809017	13
585	ENSG00000183114	FAM43B	family with sequence similarity 43, member B [Source:HGNC Symbol;Acc:HGN	-0.5800415	-0.362957402	-0.550065608	1.493064505	13
586	ENSG00000204252	HLA-DOA	major histocompatibility complex, class II, DO alpha [Source:HGNC Symbol;Ac	-0.53312122	-0.480907222	-0.485553955	1.499582397	13
587	ENSG00000205426	KRT81	keratin 81 [Source:HGNC Symbol;Acc:HGNC:6458]	-0.55273712	-0.431986581	-0.51337889	1.498102594	13
1	ENSG00000169271	HSPB3	heat shock 27kDa protein 3 [Source:HGNC Symbol;Acc:HGNC:5248]	-0.36841352	0.879176265	-1.245553397	0.734790655	14
2	ENSG00000133687	TMTC1	transmembrane and tetratricopeptide repeat containing 1 [Source:HGNC Sym	0.218009318	0.867309706	-1.440236004	0.354916981	14
3	ENSG00000173253	DMRT2	doublesex and mab-3 related transcription factor 2 [Source:HGNC Symbol;Ac	-0.1351416	1.244667544	-1.193909098	0.084383158	14
1	ENSG00000104059	FAM189A1	family with sequence similarity 189, member A1 [Source:HGNC Symbol;Acc:H	0.624298365	0.290283419	-1.483876779	0.569294995	15
2	ENSG00000025708	TYMP	thymidine phosphorylase [Source:HGNC Symbol;Acc:HGNC:3148]	1.046325581	0.066157492	-1.356871498	0.244388425	15
3	ENSG00000120949	TNFRSF8	tumor necrosis factor receptor superfamily, member 8 [Source:HGNC Symbol	1.495059904	-0.486723352	-0.602973544	-0.40536301	15
4	ENSG00000122176	FMOD	fibromodulin [Source:HGNC Symbol;Acc:HGNC:3774]	1.196852586	-0.523445456	-1.067049429	0.393642299	15
5	ENSG00000135917	SLC19A3	solute carrier family 19 (thiamine transporter), member 3 [Source:HGNC Sym	1.215116128	-0.346440231	-1.150638533	0.281962636	15
6	ENSG00000147257	GPC3	glypican 3 [Source:HGNC Symbol;Acc:HGNC:4451]	1.432421369	-0.2725109	-0.897138685	-0.26277178	15
7	ENSG00000157445	CACNA2D3	calcium channel, voltage-dependent, alpha 2/delta subunit 3 [Source:HGNC S	0.889073475	-0.064728034	-1.377984797	0.553639356	15
8	ENSG00000159403	C1R	complement component 1, r subcomponent [Source:HGNC Symbol;Acc:HGNC	1.238393241	-0.554391631	-1.022112193	0.338110583	15
9	ENSG00000161249	DMKN	dermokine [Source:HGNC Symbol;Acc:HGNC:25063]	0.967953327	-0.706312135	-1.005452366	0.743811174	15
10	ENSG00000161905	ALOX15	arachidonate 15-lipoxygenase [Source:HGNC Symbol;Acc:HGNC:433]	1.491279579	-0.499387831	-0.627802598	-0.36408915	15
11	ENSG00000162433	AK4	adenylate kinase 4 [Source:HGNC Symbol;Acc:HGNC:363]	0.694997553	0.131405808	-1.452008606	0.625605245	15
12	ENSG00000162493	PDPN	podoplanin [Source:HGNC Symbol;Acc:HGNC:29602]	1.032574071	-0.146552553	-1.314732065	0.428710548	15
13	ENSG00000162494	LRRC38	leucine rich repeat containing 38 [Source:HGNC Symbol;Acc:HGNC:27005]	1.488223802	-0.538151151	-0.623804993	-0.32626766	15
14	ENSG00000169116	PARM1	prostate androgen-regulated mucin-like protein 1 [Source:HGNC Symbol;Acc:	1.387661009	-0.701177617	-0.759845315	0.073361923	15
15	ENSG00000169605	GKN1	gastrokine 1 [Source:HGNC Symbol;Acc:HGNC:23217]	1.499082246	-0.475664311	-0.549150574	-0.47426736	15
16	ENSG00000175899	A2M	alpha-2-macroglobulin [Source:HGNC Symbol;Acc:HGNC:7]	1.257662558	-0.721185615	-0.882352648	0.345875705	15
17	ENSG00000182326	C1S	complement component 1, s subcomponent [Source:HGNC Symbol;Acc:HGNC	1.238077802	-0.462792679	-1.077734143	0.30244902	15
18	ENSG00000196136	SERPINA3	serpin peptidase inhibitor, clade A (alpha-1 antiproteinase, antitrypsin), mem	1.472777301	-0.384171627	-0.757385009	-0.33122067	15
19	ENSG00000205403	CFI	complement factor I [Source:HGNC Symbol;Acc:HGNC:5394]	1.237472811	-0.714377032	-0.90245034	0.379354561	15
20	ENSG00000273259	GAA	serpin peptidase inhibitor, clade A (alpha-1 antiproteinase, antitrypsin), mem	1.473527299	-0.377054922	-0.754901436	-0.34157094	15
21	ENSG00000152910	CNTNAP4	contactin associated protein-like 4 [Source:HGNC Symbol;Acc:HGNC:18747]	1.497410202	-0.540670354	-0.540670354	-0.41606949	15
22	ENSG00000066248	NGEF	neuronal guanine nucleotide exchange factor [Source:HGNC Symbol;Acc:HGN	0.80348243	-1.462033397	0.325425537	0.33312543	15
23	ENSG00000038295	TLL1	tolloid-like 1 [Source:HGNC Symbol;Acc:HGNC:11843]	1.498257967	-0.563740161	-0.486731058	-0.44778675	15
24	ENSG00000069482	GAL	galanin/GMAP prepropeptide [Source:HGNC Symbol;Acc:HGNC:4114]	0.915147772	-0.88162772	-0.848828329	0.815308277	15
25	ENSG00000085563	ABCB1	ATP-binding cassette, sub-family B (MDR/TAP), member 1 [Source:HGNC Sym	1.289117552	-1.098599578	-0.333068106	0.142550131	15
26	ENSG00000154175	ABI3BP	ABI family, member 3 (NESH) binding protein [Source:HGNC Symbol;Acc:HGN	0.902558782	-1.024488621	-0.690159993	0.812089832	15
1	ENSG00000112175	BMP5	bone morphogenetic protein 5 [Source:HGNC Symbol;Acc:HGNC:1072]	-0.44476275	-0.527054092	-0.527054092	1.498870931	16
2	ENSG00000106565	TMEM176B	transmembrane protein 176B [Source:HGNC Symbol;Acc:HGNC:29596]	-0.49036698	-0.504799155	-0.504799155	1.499965285	16
3	ENSG00000121075	TBX4	T-box 4 [Source:HGNC Symbol;Acc:HGNC:11603]	-0.49865649	-0.500671418	-0.500671418	1.499999323	16
4	ENSG00000124731	TREM1	triggering receptor expressed on myeloid cells 1 [Source:HGNC Symbol;Acc:H	-0.42732034	-0.535366368	-0.535366368	1.498053079	16
5	ENSG00000130032	PRRG3	proline rich Gla (G-carboxyglutamic acid) 3 (transmembrane) [Source:HGNC S	-0.48025124	-0.509801609	-0.509801609	1.499854456	16
6	ENSG00000130226	DPP6	dipeptidyl-peptidase 6 [Source:HGNC Symbol;Acc:HGNC:3010]	-0.4497084	-0.524677292	-0.524677292	1.499062985	16
7	ENSG00000138615	CILP	cartilage intermediate layer protein, nucleotide pyrophosphohydrolase [Sour	-0.41995916	-0.538841738	-0.538841738	1.497642636	16
8	ENSG00000165023	DIRAS2	DIRAS family, GTP-binding RAS-like 2 [Source:HGNC Symbol;Acc:HGNC:19323	-0.48605433	-0.506936497	-0.506936497	1.499927321	16
9	ENSG00000165349	SLC7A3	solute carrier family 7 (cationic amino acid transporter, y+ system), member 3	-0.35295341	-0.569601669	-0.569601669	1.49215675	16
10	ENSG00000184012	TMPRSS2	transmembrane protease, serine 2 [Source:HGNC Symbol;Acc:HGNC:11876]	-0.40248065	-0.54701676	-0.54701676	1.496514168	16
11	ENSG00000198759	EGFL6	EGF-like-domain, multiple 6 [Source:HGNC Symbol;Acc:HGNC:3235]	-0.29185903	-0.596306287	-0.596306287	1.484471601	16
12	ENSG00000204334	ERICH2	glutamate-rich 2 [Source:HGNC Symbol;Acc:HGNC:44395]	-0.40070372	-0.547841831	-0.547841831	1.496387379	16
13	ENSG00000237515	SHISA9	shisa family member 9 [Source:HGNC Symbol;Acc:HGNC:37231]	-0.45249833	-0.523332592	-0.523332592	1.499163518	16
14	ENSG00000049540	ELN	elastin [Source:HGNC Symbol;Acc:HGNC:3327]	-0.47897233	-0.540993799	-0.479396989	1.499363114	16
15	ENSG00000006016	CRLF1	cytokine receptor-like factor 1 [Source:HGNC Symbol;Acc:HGNC:2364]	-0.48542015	-0.517821044	-0.496623415	1.499864605	16
16	ENSG00000050767	COL23A1	collagen, type XXIII, alpha 1 [Source:HGNC Symbol;Acc:HGNC:22990]	-0.49856519	-0.501504191	-0.499929534	1.499998918	16
17	ENSG00000058085	LAMC2	laminin, gamma 2 [Source:HGNC Symbol;Acc:HGNC:6493]	-0.16896179	-0.648309632	-0.644260352	1.461531776	16
18	ENSG00000059915	PSD	pleckstrin and Sec7 domain containing [Source:HGNC Symbol;Acc:HGNC:9507	-0.42390753	-0.554524669	-0.519282786	1.497714987	16
19	ENSG00000074416	MGLL	monoglyceride lipase [Source:HGNC Symbol;Acc:HGNC:17038]	-0.35054298	-0.730979094	-0.396774082	1.478296152	16
20	ENSG00000075340	ADD2	adducin 2 (beta) [Source:HGNC Symbol;Acc:HGNC:244]	0.438893686	-1.082063883	-0.523895869	1.167066066	16
21	ENSG00000077420	APBB1IP	amyloid beta (A4) precursor protein-binding, family B, member 1 interacting p	-0.46835542	-0.542446889	-0.488463507	1.499265813	16
22	ENSG00000078596	ITM2A	integral membrane protein 2A [Source:HGNC Symbol;Acc:HGNC:6173]	-0.37463279	-0.625787369	-0.491661936	1.492082096	16
23	ENSG00000087116	ADAMTS2	ADAM metallopeptidase with thrombospondin type 1 motif, 2 [Source:HGNC	-0.38870806	-0.615743329	-0.489062581	1.493513973	16
24	ENSG00000090530	LEPREL1	leprecan-like 1 [Source:HGNC Symbol;Acc:HGNC:19317]	-0.4680712	-0.554836314	-0.475940828	1.49884834	16
25	ENSG00000101230	ISM1	isthmin 1, angiogenesis inhibitor [Source:HGNC Symbol;Acc:HGNC:16213]	-0.39318243	-0.619679036	-0.480600498	1.493461962	16
26	ENSG00000101825	MXRA5	matrix-remodelling associated 5 [Source:HGNC Symbol;Acc:HGNC:7539]	-0.36383024	-0.583417862	-0.54583945	1.493087555	16
27	ENSG00000102265	TIMP1	TIMP metallopeptidase inhibitor 1 [Source:HGNC Symbol;Acc:HGNC:11820]	0.010165853	-0.892365348	-0.51138195	1.393581445	16
28	ENSG00000102383	ZDHHC15	zinc finger, DHHC-type containing 15 [Source:HGNC Symbol;Acc:HGNC:20342	0.147352241	-0.787633638	-0.717390129	1.357671526	16
29	ENSG00000102683	SGCG	sarcoglycan, gamma (35kDa dystrophin-associated glycoprotein) [Source:HGN	-0.20295889	-0.655009545	-0.610658818	1.468627249	16

30	ENSG00000105088	OLFM2	olfactomedin 2 [Source:HGNC Symbol;Acc:HGNC:17189]	-0.44301135	-0.545495861	-0.510137242	1.498644455	16
31	ENSG00000105825	TFPI2	tissue factor pathway inhibitor 2 [Source:HGNC Symbol;Acc:HGNC:11761]	0.077811819	-0.996224163	-0.429540733	1.347953077	16
32	ENSG00000107736	CDH23	cadherin-related 23 [Source:HGNC Symbol;Acc:HGNC:13733]	-0.16758636	-0.798428952	-0.483385957	1.449401269	16
33	ENSG00000108018	SORCS1	sortilin-related VPS10 domain containing receptor 1 [Source:HGNC Symbol;Ac	-0.43142524	-0.544770599	-0.522005569	1.498201404	16
34	ENSG00000108342	CSF3	colony stimulating factor 3 (granulocyte) [Source:HGNC Symbol;Acc:HGNC:24	-0.3742948	-0.569163485	-0.550743243	1.494201529	16
35	ENSG00000112562	SMOC2	SPARC related modular calcium binding 2 [Source:HGNC Symbol;Acc:HGNC:2(	-0.47470249	-0.512613552	-0.512445457	1.499761497	16
36	ENSG00000115461	IGFBP5	insulin-like growth factor binding protein 5 [Source:HGNC Symbol;Acc:HGNC:!	-0.23979728	-0.730379698	-0.499396423	1.469573399	16
37	ENSG00000115602	IL1RL1	interleukin 1 receptor-like 1 [Source:HGNC Symbol;Acc:HGNC:5998]	-0.35208113	-0.674889062	-0.459456253	1.486426447	16
38	ENSG00000116690	PRG4	proteoglycan 4 [Source:HGNC Symbol;Acc:HGNC:9364]	-0.48495483	-0.511088084	-0.503866025	1.499908936	16
39	ENSG00000122861	PLAU	plasminogen activator, urokinase [Source:HGNC Symbol;Acc:HGNC:9052]	-0.20624247	-0.742282482	-0.514835843	1.463360795	16
40	ENSG00000124749	COL21A1	collagen, type XXI, alpha 1 [Source:HGNC Symbol;Acc:HGNC:17025]	-0.49065373	-0.507831709	-0.501476842	1.499962282	16
41	ENSG00000130052	STARD8	StAR-related lipid transfer (START) domain containing 8 [Source:HGNC Symbo	-0.22968519	-0.633254176	-0.611099666	1.47403903	16
42	ENSG00000132185	FCRLA	Fc receptor-like A [Source:HGNC Symbol;Acc:HGNC:18504]	-0.21216434	-0.884214958	-0.338412406	1.434791707	16
43	ENSG00000132386	SERPINF1	serpin peptidase inhibitor, clade F (alpha-2 antiplasmin, pigment epithelium c	-0.26574333	-0.656308886	-0.557195135	1.479247347	16
44	ENSG00000135333	EPHA7	EPH receptor A7 [Source:HGNC Symbol;Acc:HGNC:3390]	-0.39664539	-0.556891544	-0.542527346	1.496064278	16
45	ENSG00000136052	SLC41A2	solute carrier family 41 (magnesium transporter), member 2 [Source:HGNC Sy	-0.31361206	-0.676184267	-0.493680094	1.483476417	16
46	ENSG00000136235	GPNMB	glycoprotein (transmembrane) nmb [Source:HGNC Symbol;Acc:HGNC:4462]	-0.46603484	-0.532740045	-0.500668536	1.499443425	16
47	ENSG00000137571	SLCO5A1	solute carrier organic anion transporter family, member 5A1 [Source:HGNC Sy	-0.22268458	-0.748584041	-0.493741037	1.465009659	16
48	ENSG00000137868	STRA6	stimulated by retinoic acid 6 [Source:HGNC Symbol;Acc:HGNC:30650]	-0.4466393	-0.555973667	-0.495887135	1.498500105	16
49	ENSG00000139269	INHBE	inhibin, beta E [Source:HGNC Symbol;Acc:HGNC:24029]	-0.31547927	-0.587072857	-0.585188631	1.487740756	16
50	ENSG00000140379	BCL2A1	BCL2-related protein A1 [Source:HGNC Symbol;Acc:HGNC:991]	-0.41470356	-0.591956557	-0.489374861	1.496034973	16
51	ENSG00000141433	ADCYAP1	adenylate cyclase activating polypeptide 1 (pituitary) [Source:HGNC Symbol;A	-0.3895071	-0.608347912	-0.496145287	1.494000298	16
52	ENSG00000143320	CRABP2	cellular retinoic acid binding protein 2 [Source:HGNC Symbol;Acc:HGNC:2339	0.311694606	-0.916510931	-0.667115636	1.271931961	16
53	ENSG00000144834	TAGLN3	transgelin 3 [Source:HGNC Symbol;Acc:HGNC:29868]	0.096604297	-1.164533626	-0.195518892	1.263448221	16
54	ENSG00000149968	MMP3	matrix metallopeptidase 3 (stromelysin 1, progelatinase) [Source:HGNC Symb	-0.49881737	-0.500814008	-0.500368076	1.499999451	16
55	ENSG00000154065	ANKRD29	ankyrin repeat domain 29 [Source:HGNC Symbol;Acc:HGNC:27110]	-0.3763178	-0.613936164	-0.502662038	1.492915998	16
56	ENSG00000158050	DUSP2	dual specificity phosphatase 2 [Source:HGNC Symbol;Acc:HGNC:3068]	-0.46020421	-0.540524543	-0.498464004	1.499192761	16
57	ENSG00000159674	SPON2	spondin 2, extracellular matrix protein [Source:HGNC Symbol;Acc:HGNC:1125	-0.36146167	-0.589572883	-0.541713254	1.49274781	16
58	ENSG00000162746	FCRLB	Fc receptor-like B [Source:HGNC Symbol;Acc:HGNC:26431]	-0.20609149	-0.932598181	-0.279030034	1.417719708	16
59	ENSG00000164626	KCNK5	potassium channel, subfamily K, member 5 [Source:HGNC Symbol;Acc:HGNC:	-0.45285406	-0.547249867	-0.498781572	1.498885496	16
60	ENSG00000165474	GJB2	gap junction protein, beta 2, 26kDa [Source:HGNC Symbol;Acc:HGNC:4284]	-0.10635555	-0.722475105	-0.615933664	1.44476432	16
61	ENSG00000169760	NLGN1	neuroligin 1 [Source:HGNC Symbol;Acc:HGNC:14291]	-0.0544361	-0.748226117	-0.627031927	1.429694146	16
62	ENSG00000170345	FOS	FBJ murine osteosarcoma viral oncogene homolog [Source:HGNC Symbol;Acc	-0.39992064	-0.557545701	-0.538821585	1.49628793	16
63	ENSG00000170893	TRH	thyrotropin-releasing hormone [Source:HGNC Symbol;Acc:HGNC:12298]	-0.49962955	-0.500474367	-0.499895994	1.499999907	16
64	ENSG00000170989	S1PR1	sphingosine-1-phosphate receptor 1 [Source:HGNC Symbol;Acc:HGNC:3165]	0.365460501	-1.16844401	-0.366227582	1.169211091	16
65	ENSG00000173406	DAB1	Dab, reelin signal transducer, homolog 1 (Drosophila) [Source:HGNC Symbol;A	-0.22089082	-0.744892328	-0.499445976	1.465229123	16
66	ENSG00000179603	GRM8	glutamate receptor, metabotropic 8 [Source:HGNC Symbol;Acc:HGNC:4600]	-0.35034418	-0.586031269	-0.555390999	1.491766451	16
67	ENSG00000180209	MYLPF	myosin light chain, phosphorylatable, fast skeletal muscle [Source:HGNC Sym	-0.49094068	-0.505542685	-0.503485399	1.499968765	16
68	ENSG00000181773	GPR3	G protein-coupled receptor 3 [Source:HGNC Symbol;Acc:HGNC:4484]	-0.09565151	-0.943171017	-0.364246784	1.403069315	16
69	ENSG00000182256	GABRG3	gamma-aminobutyric acid (GABA) A receptor, gamma 3 [Source:HGNC Symbo	-0.42609298	-0.552883685	-0.51886844	1.497845102	16
70	ENSG00000183715	OPCML	opioid binding protein/cell adhesion molecule-like [Source:HGNC Symbol;Acc	-0.22819142	-0.726101883	-0.514157257	1.46845056	16
71	ENSG00000185565	LSAMP	limbic system-associated membrane protein [Source:HGNC Symbol;Acc:HGNC	-0.16488149	-0.655983604	-0.639707881	1.46057298	16
72	ENSG00000186462	NAP1L2	nucleosome assembly protein 1-like 2 [Source:HGNC Symbol;Acc:HGNC:7638]	-0.4139419	-0.557516107	-0.525696368	1.497154375	16
73	ENSG00000187479	C11orf96	chromosome 11 open reading frame 96 [Source:HGNC Symbol;Acc:HGNC:386	-0.44920882	-0.551946057	-0.497523622	1.498678496	16
74	ENSG00000187955	COL14A1	collagen, type XIV, alpha 1 [Source:HGNC Symbol;Acc:HGNC:2191]	-0.41326753	-0.58167481	-0.501505612	1.496447955	16
75	ENSG00000196220	SRGAP3	SLIT-ROBO Rho GTPase activating protein 3 [Source:HGNC Symbol;Acc:HGNC:	-0.47422952	-0.517945739	-0.507563884	1.499739138	16
76	ENSG00000196935	SRGAP1	SLIT-ROBO Rho GTPase activating protein 1 [Source:HGNC Symbol;Acc:HGNC:	-0.38189976	-0.644635251	-0.4644111	1.490946109	16
77	ENSG00000197249	SERPINA1	serpin peptidase inhibitor, clade A (alpha-1 antiproteinase, antitrypsin), mem	-0.47211341	-0.525319071	-0.50221159	1.499644066	16
78	ENSG00000197261	C6orf141	chromosome 6 open reading frame 141 [Source:HGNC Symbol;Acc:HGNC:213	-0.20246699	-0.823937005	-0.423115886	1.449519878	16
79	ENSG00000198088	NUP62CL	nucleoporin 62kDa C-terminal like [Source:HGNC Symbol;Acc:HGNC:25960]	-0.07674555	-0.784021443	-0.57185415	1.43262114	16
80	ENSG00000222047	C10orf55	chromosome 10 open reading frame 55 [Source:HGNC Symbol;Acc:HGNC:310	-0.18636491	-0.740832004	-0.533043151	1.460240069	16
1	ENSG00000007237	GAS7	growth arrest-specific 7 [Source:HGNC Symbol;Acc:HGNC:4169]	-0.44681341	-0.477341249	-0.573652407	1.497807071	17
2	ENSG00000006128	TAC1	tachykinin, precursor 1 [Source:HGNC Symbol;Acc:HGNC:11517]	-0.48952515	-0.503321864	-0.50711016	1.499957172	17
3	ENSG00000008394	MGST1	microsomal glutathione S-transferase 1 [Source:HGNC Symbol;Acc:HGNC:706	0.143031027	0.026723244	-1.302337553	1.132583282	17
4	ENSG00000019582	CD74	CD74 molecule, major histocompatibility complex, class II invariant chain [Sou	-0.20383632	-0.526382278	-0.733699794	1.463918394	17
5	ENSG00000050555	LAMC3	laminin, gamma 3 [Source:HGNC Symbol;Acc:HGNC:6494]	-0.42938905	-0.500902133	-0.567327469	1.497618655	17
6	ENSG00000062282	DGAT2	diacylglycerol O-acyltransferase 2 [Source:HGNC Symbol;Acc:HGNC:16940]	-0.47886788	-0.488230557	-0.532491328	1.499589765	17
7	ENSG00000069667	RORA	RAR-related orphan receptor A [Source:HGNC Symbol;Acc:HGNC:10258]	-0.47791673	-0.493724954	-0.52803011	1.499671789	17
8	ENSG00000073756	PTGS2	prostaglandin-endoperoxide synthase 2 (prostaglandin G/H synthase and cycl	-0.19919664	-0.422653266	-0.826690151	1.448540061	17
9	ENSG00000076864	RAP1GAP	RAP1 GTPase activating protein [Source:HGNC Symbol;Acc:HGNC:9858]	-0.47945467	-0.480065151	-0.539877652	1.499397475	17
10	ENSG00000078081	LAMP3	lysosomal-associated membrane protein 3 [Source:HGNC Symbol;Acc:HGNC::	-0.26741868	-0.512895467	-0.696346583	1.476660734	17
11	ENSG00000080573	COL5A3	collagen, type V, alpha 3 [Source:HGNC Symbol;Acc:HGNC:14864]	-0.2672824	-0.580646881	-0.632424799	1.480354077	17
12	ENSG00000080854	IGSF9B	immunoglobulin superfamily, member 9B [Source:HGNC Symbol;Acc:HGNC:3:	0.175550427	-0.546899201	-0.954749516	1.32609829	17
13	ENSG00000081041	CXCL2	chemokine (C-X-C motif) ligand 2 [Source:HGNC Symbol;Acc:HGNC:4603]	-0.43688015	-0.509987635	-0.551448799	1.49831658	17
14	ENSG00000084628	NKAIN1	Na+/K+ transporting ATPase interacting 1 [Source:HGNC Symbol;Acc:HGNC:2!	-0.27799546	-0.428744402	-0.76223666	1.468976526	17
15	ENSG00000086619	ERO1LB	ERO1-like beta (S. cerevisiae) [Source:HGNC Symbol;Acc:HGNC:14355]	-0.3093248	-0.560614115	-0.61660867	1.486547587	17
16	ENSG00000092758	COL9A3	collagen, type IX, alpha 3 [Source:HGNC Symbol;Acc:HGNC:2219]	-0.13394203	-0.607494824	-0.710518194	1.45195505	17
17	ENSG00000095383	TBC1D2	TBC1 domain family, member 2 [Source:HGNC Symbol;Acc:HGNC:18026]	-0.30756013	-0.573190705	-0.605807682	1.48655852	17
18	ENSG00000099958	DERL3	derlin 3 [Source:HGNC Symbol;Acc:HGNC:14236]	-0.39650412	-0.5242395	-0.575020155	1.49576377	17
19	ENSG00000101680	LAMA1	laminin, alpha 1 [Source:HGNC Symbol;Acc:HGNC:6481]	0.81813092	-0.564393706	-1.12183852	0.868101306	17
20	ENSG00000102230	PCYT1B	phosphate cytidylyltransferase 1, choline, beta [Source:HGNC Symbol;Acc:HG	-0.38677071	-0.465238027	-0.639594649	1.491603391	17
21	ENSG00000103021	CCDC113	coiled-coil domain containing 113 [Source:HGNC Symbol;Acc:HGNC:25002]	-0.43022277	-0.430838035	-0.632148782	1.493209588	17
22	ENSG00000104313	EYA1	EYA transcriptional coactivator and phosphatase 1 [Source:HGNC Symbol;Acc	-0.31247712	-0.405619415	-0.754504522	1.472601055	17
23	ENSG00000105419	MEIS3	Meis homeobox 3 [Source:HGNC Symbol;Acc:HGNC:29537]	-0.38676029	-0.531467015	-0.576837262	1.495064568	17
24	ENSG00000106236	NPTX2	neuronal pentraxin II [Source:HGNC Symbol;Acc:HGNC:7953]	-0.38503719	-0.512234109	-0.597025549	1.494296849	17
25	ENSG00000106819	ASPN	asporin [Source:HGNC Symbol;Acc:HGNC:14872]	-0.28909179	-0.3619261	-0.808846221	1.459864112	17
26	ENSG00000106823	ECM2	extracellular matrix protein 2, female organ and adipocyte specific [Source:HG	-0.38206167	-0.422101624	-0.682473517	1.486636808	17
27	ENSG00000108846	ABCC3	ATP-binding cassette, sub-family C (CFTR/MRP), member 3 [Source:HGNC Syn	-0.1555208	-0.186229408	-1.02969461	1.371444821	17
28	ENSG00000108950	FAM20A	family with sequence similarity 20, member A [Source:HGNC Symbol;Acc:HGN	-0.36488041	-0.523958755	-0.603748336	1.492587503	17
29	ENSG00000109625	CPZ	carboxypeptidase Z [Source:HGNC Symbol;Acc:HGNC:2333]	0.091475803	-0.708169234	-0.764027548	1.380720978	17
30	ENSG00000110324	IL10RA	interleukin 10 receptor, alpha [Source:HGNC Symbol;Acc:HGNC:5964]	-0.44532712	-0.492244931	-0.560742507	1.498314561	17
31	ENSG00000110900	TSPAN11	tetraspanin 11 [Source:HGNC Symbol;Acc:HGNC:30795]	-0.34760211	-0.417211737	-0.715918397	1.480732247	17
32	ENSG00000111424	VDR	vitamin D (1,25- dihydroxyvitamin D3) receptor [Source:HGNC Symbol;Acc:HG	-0.44650921	-0.457665203	-0.592519514	1.496693927	17
33	ENSG00000112715	VEGFA	vascular endothelial growth factor A [Source:HGNC Symbol;Acc:HGNC:12680]	-0.46115534	-0.483415831	-0.554246815	1.498817986	17

34	ENSG00000115009	CCL20	chemokine (C-C motif) ligand 20 [Source:HGNC Symbol;Acc:HGNC:10619]	-0.18461947	-0.467288943	-0.799893119	1.451801533	17
35	ENSG00000116016	EPAS1	endothelial PAS domain protein 1 [Source:HGNC Symbol;Acc:HGNC:3374]	-0.13810493	-0.622682286	-0.692871318	1.453658535	17
36	ENSG00000116183	PAPPA2	pappalysin 2 [Source:HGNC Symbol;Acc:HGNC:14615]	-0.04622115	-0.65004199	-0.731895706	1.428158847	17
37	ENSG00000116194	ANGPTL1	angiopoietin-like 1 [Source:HGNC Symbol;Acc:HGNC:489]	-0.37307203	-0.510586866	-0.609287049	1.492945948	17
38	ENSG00000118113	MMP8	matrix metallopeptidase 8 (neutrophil collagenase) [Source:HGNC Symbol;Acc	-0.19147254	-0.622830115	-0.652064541	1.466367195	17
39	ENSG00000118194	TNNT2	troponin T type 2 (cardiac) [Source:HGNC Symbol;Acc:HGNC:11949]	0.061846959	-0.238246385	-1.120902275	1.297301701	17
40	ENSG00000122121	XPNPEP2	X-prolyl aminopeptidase (aminopeptidase P) 2, membrane-bound [Source:HG	0.790679436	-0.844459187	-0.88422718	0.938006932	17
41	ENSG00000124875	CXCL6	chemokine (C-X-C motif) ligand 6 [Source:HGNC Symbol;Acc:HGNC:10643]	-0.03930774	-0.29098289	-1.030521425	1.360812056	17
42	ENSG00000125740	FOSB	FBJ murine osteosarcoma viral oncogene homolog B [Source:HGNC Symbol;A	-0.41584268	-0.540421061	-0.541132517	1.497396255	17
43	ENSG00000125848	FLRT3	fibronectin leucine rich transmembrane protein 3 [Source:HGNC Symbol;Acc:	-0.38547762	-0.465078506	-0.640880022	1.491436148	17
44	ENSG00000128165	ADM2	adrenomedullin 2 [Source:HGNC Symbol;Acc:HGNC:28898]	-0.20103965	-0.425010786	-0.823412048	1.449462483	17
45	ENSG00000128274	A4GALT	alpha 1,4-galactosyltransferase [Source:HGNC Symbol;Acc:HGNC:18149]	-0.01554503	-0.372558486	-0.986127541	1.374231057	17
46	ENSG00000128590	DNAJB9	DnaJ (Hsp40) homolog, subfamily B, member 9 [Source:HGNC Symbol;Acc:HG	-0.24673967	-0.443505245	-0.773851095	1.464096007	17
47	ENSG00000130054	FAM155B	family with sequence similarity 155, member B [Source:HGNC Symbol;Acc:HG	-0.12506784	-0.381089489	-0.911187457	1.41734479	17
48	ENSG00000130487	KLHDC7B	kelch domain containing 7B [Source:HGNC Symbol;Acc:HGNC:25145]	-0.38668951	-0.521275178	-0.586821069	1.494785753	17
49	ENSG00000130595	TNNT3	troponin T type 3 (skeletal, fast) [Source:HGNC Symbol;Acc:HGNC:11950]	-0.39190863	-0.392123552	-0.700067588	1.484099768	17
50	ENSG00000132000	PODNL1	podocan-like 1 [Source:HGNC Symbol;Acc:HGNC:26275]	-0.40730073	-0.487189391	-0.600776005	1.49526613	17
51	ENSG00000133048	CHI3L1	chitinase 3-like 1 (cartilage glycoprotein-39) [Source:HGNC Symbol;Acc:HGNC	-0.37885491	-0.556212405	-0.559579109	1.494646425	17
52	ENSG00000133107	TRPC4	transient receptor potential cation channel, subfamily C, member 4 [Source:H	0.680111202	-0.629140655	-1.059973503	1.009002955	17
53	ENSG00000133134	BEX2	brain expressed X-linked 2 [Source:HGNC Symbol;Acc:HGNC:30933]	-0.35294053	-0.541699826	-0.597142266	1.491782623	17
54	ENSG00000133401	PDZD2	PDZ domain containing 2 [Source:HGNC Symbol;Acc:HGNC:18486]	0.213597037	0.135234937	-1.37342515	1.024593176	17
55	ENSG00000134259	NGF	nerve growth factor (beta polypeptide) [Source:HGNC Symbol;Acc:HGNC:780	-0.38300274	-0.393506901	-0.70650031	1.483009956	17
56	ENSG00000135097	MSI1	musashi RNA-binding protein 1 [Source:HGNC Symbol;Acc:HGNC:7330]	-0.48517302	-0.499879432	-0.514837547	1.499889995	17
57	ENSG00000135744	AGT	angiotensinogen (serpin peptidase inhibitor, clade A, member 8) [Source:HGN	-0.48455916	-0.500218814	-0.515105363	1.499883337	17
58	ENSG00000135842	FAM129A	family with sequence similarity 129, member A [Source:HGNC Symbol;Acc:HG	-0.13736176	-0.510180021	-0.796763116	1.444304895	17
59	ENSG00000136002	ARHGEF4	Rho guanine nucleotide exchange factor (GEF) 4 [Source:HGNC Symbol;Acc:H	-0.24711351	-0.261448909	-0.916458199	1.425020615	17
60	ENSG00000137440	FGFBP1	fibroblast growth factor binding protein 1 [Source:HGNC Symbol;Acc:HGNC:1	0.759100776	-0.497038682	-1.166014209	0.903952115	17
61	ENSG00000138207	RBP4	retinol binding protein 4, plasma [Source:HGNC Symbol;Acc:HGNC:9922]	-0.29852186	-0.521205211	-0.663275222	1.483002295	17
62	ENSG00000139329	LUM	lumican [Source:HGNC Symbol;Acc:HGNC:6724]	-0.45900152	-0.467027684	-0.571982432	1.498011632	17
63	ENSG00000142549	IGLON5	IgLON family member 5 [Source:HGNC Symbol;Acc:HGNC:34550]	-0.48007218	-0.499900634	-0.519829591	1.499802405	17
64	ENSG00000143162	CREG1	cellular repressor of E1A-stimulated genes 1 [Source:HGNC Symbol;Acc:HGNC	0.327153373	0.26375407	-1.446283565	0.855376122	17
65	ENSG00000143196	DPT	dermatopontin [Source:HGNC Symbol;Acc:HGNC:3011]	-0.04464795	-0.424206539	-0.929218557	1.398073047	17
66	ENSG00000144857	BOC	BOC cell adhesion associated, oncogene regulated [Source:HGNC Symbol;Acc	-0.38261258	-0.444900256	-0.661716181	1.489229019	17
67	ENSG00000147160	AWAT2	acyl-CoA wax alcohol acyltransferase 2 [Source:HGNC Symbol;Acc:HGNC:2325	-0.16849197	-0.202348762	-1.010803695	1.381644428	17
68	ENSG00000149131	SERPING1	serpin peptidase inhibitor, clade G (C1 inhibitor), member 1 [Source:HGNC Sy	0.716717359	-0.583517486	-1.100271177	0.967071304	17
69	ENSG00000150051	MKX	mohawk homeobox [Source:HGNC Symbol;Acc:HGNC:23729]	-0.46322671	-0.50735185	-0.528861533	1.499440093	17
70	ENSG00000151067	CACNA1C	calcium channel, voltage-dependent, L type, alpha 1C subunit [Source:HGNC S	-0.12009188	-0.531825837	-0.789890789	1.441808504	17
71	ENSG00000151090	THRB	thyroid hormone receptor, beta [Source:HGNC Symbol;Acc:HGNC:11799]	-0.15097235	-0.644534045	-0.661814642	1.457321034	17
72	ENSG00000151812	SLC35F4	solute carrier family 35, member F4 [Source:HGNC Symbol;Acc:HGNC:19845]	-0.38918976	-0.49084697	-0.613634392	1.493671121	17
73	ENSG00000151892	GFRA1	GDNF family receptor alpha 1 [Source:HGNC Symbol;Acc:HGNC:4243]	0.257702244	-0.245611807	-1.204625901	1.192535464	17
74	ENSG00000152583	SPARCL1	SPARC-like 1 (hevin) [Source:HGNC Symbol;Acc:HGNC:11220]	-0.41496738	-0.538195063	-0.54417556	1.497338006	17
75	ENSG00000152669	CCNO	cyclin O [Source:HGNC Symbol;Acc:HGNC:18576]	-0.14171321	-0.535573914	-0.771248107	1.448535228	17
76	ENSG00000154736	ADAMTS5	ADAM metallopeptidase with thrombospondin type 1 motif, 5 [Source:HGNC	-0.49655429	-0.497176162	-0.506254805	1.499985258	17
77	ENSG00000155269	GPR78	G protein-coupled receptor 78 [Source:HGNC Symbol;Acc:HGNC:4528]	0.078621237	-0.699147683	-0.765023874	1.38555032	17
78	ENSG00000157064	NMNAT2	nicotinamide nucleotide adenylyltransferase 2 [Source:HGNC Symbol;Acc:HGI	-0.14541521	-0.595651568	-0.713317069	1.45438385	17
79	ENSG00000159640	ACE	angiotensin I converting enzyme [Source:HGNC Symbol;Acc:HGNC:2707]	0.349383757	-0.654088766	-0.943931288	1.248636296	17
80	ENSG00000160588	MPZL3	myelin protein zero-like 3 [Source:HGNC Symbol;Acc:HGNC:27279]	-0.33094423	-0.505241444	-0.650926284	1.487111957	17
81	ENSG00000160963	COL26A1	collagen, type XXVI, alpha 1 [Source:HGNC Symbol;Acc:HGNC:18038]	-0.00720127	-0.554025406	-0.845334328	1.406561007	17
82	ENSG00000162706	CADM3	cell adhesion molecule 3 [Source:HGNC Symbol;Acc:HGNC:17601]	0.124544848	-0.717719436	-0.774262683	1.367437271	17
83	ENSG00000162849	KIF26B	kinesin family member 26B [Source:HGNC Symbol;Acc:HGNC:25484]	0.1346945	-0.655099578	-0.839181655	1.359586733	17
84	ENSG00000163235	TGFA	transforming growth factor, alpha [Source:HGNC Symbol;Acc:HGNC:11765]	-0.42592546	-0.529453842	-0.542577793	1.497957098	17
85	ENSG00000163393	SLC22A15	solute carrier family 22, member 15 [Source:HGNC Symbol;Acc:HGNC:20301]	-0.40598752	-0.487092265	-0.602059058	1.49513884	17
86	ENSG00000163734	CXCL3	chemokine (C-X-C motif) ligand 3 [Source:HGNC Symbol;Acc:HGNC:4604]	-0.46083833	-0.517788242	-0.520802648	1.499429216	17
87	ENSG00000163735	CXCL5	chemokine (C-X-C motif) ligand 5 [Source:HGNC Symbol;Acc:HGNC:10642]	-0.49070988	-0.503532242	-0.505724988	1.49996711	17
88	ENSG00000163739	CXCL1	chemokine (C-X-C motif) ligand 1 (melanoma growth stimulating activity, alph	0.348797389	-0.641210754	-0.95480112	1.247214485	17
89	ENSG00000164379	FOXQ1	forkhead box Q1 [Source:HGNC Symbol;Acc:HGNC:20951]	-0.49539342	-0.49548472	-0.509090798	1.499968937	17
90	ENSG00000164694	FNDC1	fibronectin type III domain containing 1 [Source:HGNC Symbol;Acc:HGNC:211	-0.10663882	-0.329087885	-0.962263854	1.397990558	17
91	ENSG00000166482	MFAP4	microfibrillar-associated protein 4 [Source:HGNC Symbol;Acc:HGNC:7035]	0.204351356	-0.705093417	-0.830302946	1.331045008	17
92	ENSG00000166863	TAC3	tachykinin 3 [Source:HGNC Symbol;Acc:HGNC:11521]	0.315902979	-0.749065779	-0.842968212	1.276131012	17
93	ENSG00000167311	ART5	ADP-ribosyltransferase 5 [Source:HGNC Symbol;Acc:HGNC:24049]	-0.44673779	-0.447699929	-0.60158539	1.496023108	17
94	ENSG00000167861	HID1	HID1 domain containing [Source:HGNC Symbol;Acc:HGNC:15736]	0.120212254	-0.205176413	-1.169920947	1.254885106	17
95	ENSG00000168477	TNXB	tenascin XB [Source:HGNC Symbol;Acc:HGNC:11976]	0.020093995	-0.583147119	-0.836735764	1.399788888	17
96	ENSG00000169071	ROR2	receptor tyrosine kinase-like orphan receptor 2 [Source:HGNC Symbol;Acc:HG	0.327599554	0.067072832	-1.382725712	0.988053326	17
97	ENSG00000169174	PCSK9	proprotein convertase subtilisin/kexin type 9 [Source:HGNC Symbol;Acc:HGN	-0.19488375	-0.624199499	-0.648037363	1.467120611	17
98	ENSG00000171872	KLF17	Kruppel-like factor 17 [Source:HGNC Symbol;Acc:HGNC:18830]	-0.40818641	-0.477869678	-0.608750623	1.494806711	17
99	ENSG00000171951	SCG2	secretogranin II [Source:HGNC Symbol;Acc:HGNC:10575]	-0.12505167	-0.62340857	-0.701787415	1.450247654	17
100	ENSG00000172817	CYP7B1	cytochrome P450, family 7, subfamily B, polypeptide 1 [Source:HGNC Symbol	0.33414102	-0.21267162	-1.251478828	1.130009427	17
101	ENSG00000173083	HPSE	heparanase [Source:HGNC Symbol;Acc:HGNC:5164]	0.438385701	-0.525221615	-1.080901836	1.16773775	17
102	ENSG00000173110	HSPA6	heat shock 70kDa protein 6 (HSP70B') [Source:HGNC Symbol;Acc:HGNC:5239]	-0.40485439	-0.537067639	-0.554719906	1.49664194	17
103	ENSG00000175426	PCSK1	proprotein convertase subtilisin/kexin type 1 [Source:HGNC Symbol;Acc:HGN	-0.48994999	-0.500746743	-0.509256453	1.499953189	17
104	ENSG00000177614	PGBD5	piggyBac transposable element derived 5 [Source:HGNC Symbol;Acc:HGNC:1	-0.48184768	-0.503990027	-0.514026744	1.499864451	17
105	ENSG00000178752	FAM132B	family with sequence similarity 132, member B [Source:HGNC Symbol;Acc:HG	0.599858263	-0.518992361	-1.128446587	1.047580685	17
106	ENSG00000179059	ZFP42	ZFP42 zinc finger protein [Source:HGNC Symbol;Acc:HGNC:30949]	-0.39493033	-0.470079122	-0.627904385	1.49291384	17
107	ENSG00000180535	BHLHA15	basic helix-loop-helix family, member a15 [Source:HGNC Symbol;Acc:HGNC:2	-0.12980552	-0.616412324	-0.705029715	1.451247558	17
108	ENSG00000181195	PENK	proenkephalin [Source:HGNC Symbol;Acc:HGNC:8831]	-0.48502024	-0.4965946	-0.518242958	1.499857796	17
109	ENSG00000182585	EPGN	epithelial mitogen [Source:HGNC Symbol;Acc:HGNC:17470]	-0.41240005	-0.50549292	-0.578632271	1.496525242	17
110	ENSG00000183196	CHST6	carbohydrate (N-acetylglucosamine 6-O) sulfotransferase 6 [Source:HGNC Syr	-0.38693811	-0.40501403	-0.693209048	1.485161187	17
111	ENSG00000183421	RIPK4	receptor-interacting serine-threonine kinase 4 [Source:HGNC Symbol;Acc:HGI	-0.28148586	-0.47588607	-0.71846763	1.475839565	17
112	ENSG00000183801	OLFML1	olfactomedin-like 1 [Source:HGNC Symbol;Acc:HGNC:24473]	0.205559271	-0.253116045	-1.178832695	1.226389469	17
113	ENSG00000186907	RTN4RL2	reticulon 4 receptor-like 2 [Source:HGNC Symbol;Acc:HGNC:23053]	-0.45334696	-0.522253038	-0.523592761	1.499192756	17
114	ENSG00000189001	SBSN	suprabasin [Source:HGNC Symbol;Acc:HGNC:24950]	-0.20358502	-0.6324061	-0.632998042	1.468989157	17
115	ENSG00000197181	PIWIL2	piwi-like RNA-mediated gene silencing 2 [Source:HGNC Symbol;Acc:HGNC:17	-0.18861979	-0.398181673	-0.854141417	1.440942876	17
116	ENSG00000197935	ZNF311	zinc finger protein 311 [Source:HGNC Symbol;Acc:HGNC:13847]	0.07992789	-0.222769723	-1.139732106	1.282573939	17
117	ENSG00000198729	PPP1R14C	protein phosphatase 1, regulatory (inhibitor) subunit 14C [Source:HGNC Symb	-0.33750371	-0.53794222	-0.614300474	1.489746402	17

118	ENSG00000198797	BRINP2	bone morphogenetic protein/retinoic acid inducible neural-specific 2 [Source	-0.37032778	-0.538078108	-0.585202816	1.493608701	17
119	ENSG00000204291	COL15A1	collagen, type XV, alpha 1 [Source:HGNC Symbol;Acc:HGNC:2192]	-0.11623039	-0.650478084	-0.681879969	1.448588439	17
120	ENSG00000205300	RP11-352D3.2		-0.36373832	-0.533289657	-0.595748186	1.492776164	17
121	ENSG00000206384	COL6A6	collagen, type VI, alpha 6 [Source:HGNC Symbol;Acc:HGNC:27023]	0.078481849	-0.208483205	-1.147831787	1.277833143	17
122	ENSG00000250038	RP11-180C1.1		-0.37607257	-0.53305347	-0.584948489	1.494074526	17
123	ENSG00000261594	TPBGL	trophoblast glycoprotein-like [Source:HGNC Symbol;Acc:HGNC:44159]	-0.41437986	-0.506758226	-0.575586771	1.496724858	17
124	ENSG00000262655	SPON1	spondin 1, extracellular matrix protein [Source:HGNC Symbol;Acc:HGNC:1125	-0.17430125	-0.602810935	-0.684823235	1.461935425	17
125	ENSG00000269113	TRABD2B	TraB domain containing 2B [Source:HGNC Symbol;Acc:HGNC:44200]	-0.3939096	-0.540745618	-0.561175968	1.495831186	17
