# Supplementary material for: Human fetal mesoangioblasts reveal tissue‐dependent transcriptional signatures
Source: Stem Cells Transl Med. 2020 Jan 23;9(5):575–89. doi: 10.1002/sctm.19-0209 (PMC7180296; doi:10.1002/sctm.19-0209)
Supplement: Supplementary file 8 — Table S2 List of transcriptionally enriched transcription factors within the different gene clusters (list related to Figure 4C). Z‐score were expressed by 1, 2, or 3 + symbols according to these values: +: 0.5 < z‐score < 1; ++: 1 < z‐score < 1.25; +++: z‐score > 1.25. [file SCT3-9-575-s008.pdf]

Supplementary Table 1. List of transcriptionally enriched transcription factors within the different gene clusters and superclusters.

Symbols: +: 0.5 < z-score < 1 ++: 1<zscore< 1.25 +++: z-score>1.25

Cluster	Super-cluster	List #	Abbrevation	Full name	Ensembl code	Degree of expression			
						Skeletal	Aorta	Ventricle	Atrium
1	1	1	**TAL1**	T-cell acute lymphocytic leukemia 1	ENSG00000162367				+++
		2	**ST18**	suppression of tumorigenicity 18, zinc finger	ENSG00000147488			+	++
2		1	**ZFP57**	ZFP57 zinc finger protein	ENSG00000204644			+	+
		2	**TFAP2D**	transcription factor AP-2 delta (activating enhancer binding protein 2 delta)	ENSG00000008197				+++
		3	**GATA4**	GATA binding protein 4	ENSG00000136574			+	+
		4	**ZHX2**	zinc fingers and homeoboxes 2	ENSG00000178764			+	+
		5	**WT1**	Wilms tumor 1	ENSG00000184937			+	+
		6	**GATA3**	GATA binding protein 3	ENSG00000107485				+++
		7	**SMAD6**	SMAD family member 6	ENSG00000137834			+	+
		8	**BCL6B**	B-cell CLL/lymphoma 6, member B	ENSG00000161940			+	+
		9	**GATA5**	GATA binding protein 5	ENSG00000130700				++
		10	**LYL1**	lymphoblastic leukemia-assoc. hematopoiesis regulator 1	ENSG00000104903				+
3		1	**LIN28A**	lin-28 homolog A (C. elegans)	ENSG00000131914			+++	
		2	**MYCL**	v-myc avian myelocytomatosis viral oncogene lung carcinoma derived homolog	ENSG00000116990			+	+
		3	**MYCN**	v-myc avian myelocytomatosis viral oncogene neuroblastoma derived homolog	ENSG00000134323			+++	
		4	**VAX2**	ventral anterior homeobox 2	ENSG00000116035			+++	
		5	**STAT1**	signal transducer and activator of transcription 1, 91kDa	ENSG00000115415			++	+
		6	**STAT4**	signal transducer and activator of transcription 4	ENSG00000138378			+	+
		7	**GBX2**	gastrulation brain homeobox 2	ENSG00000168505			+++	
		8	**RARB**	retinoic acid receptor, beta	ENSG00000077092			++	
		9	**MECOM**	MDS1 and EVI1 complex locus	ENSG00000085276			+	
		10	**MXD4**	MAX dimerization protein 4	ENSG00000123933			+	+
		11	**ETV7**	ets variant 7	ENSG00000010030			+	+
		12	**TCF21**	transcription factor 21	ENSG00000118526			+++	
		13	**ZNF117**	zinc finger protein 117	ENSG00000152926			++	+
		14	**MYRF**	myelin regulatory factor	ENSG00000124920			+	+
		15	**PGR**	progesterone receptor	ENSG00000082175			+++	
		16	**BHLHE41**	basic helix-loop-helix family, member e41	ENSG00000123095			++	
		17	**ZNF641**	zinc finger protein 641	ENSG00000167528			++	
		18	**FOXO1**	forkhead box O1	ENSG00000150907			+	+
		19	**NPAS3**	neuronal PAS domain protein 3	ENSG00000151322			+	+
		20	**ARNT2**	aryl-hydrocarbon receptor nuclear translocator 2	ENSG00000172379			+++	
		21	**STAT5A**	signal transducer and activator of transcription 5A	ENSG00000126561			+	+
		22	**GATA6**	GATA binding protein 6	ENSG00000141448			+	+
		23	**FOXS1**	forkhead box S1	ENSG00000179772			++	
		24	**L3MBTL1**	l(3)mbt-like 1 (Drosophila)	ENSG00000185513			++	
		25	**APOL2**	apolipoprotein L, 2	ENSG00000128335			++	+
4		1	**MSC**	musculin	ENSG00000178860			+	+
		2	**LHX2**	LIM homeobox 2	ENSG00000106689			+++	
		3	**MAF**	v-maf avian musculoaponeurotic fibrosarcoma oncogne homolog	ENSG00000178573			+	+
5	2	1	**OSR1**	odd-skipped related transciption factor 1	ENSG00000143867		+++		
		2	**ZBED2**	zinc finger, BED-type containing 2	ENSG00000177494		+++		
		3	**MEF2C**	myocyte enhancer factor 2C	ENSG00000081189		+++		
		4	**ZNF467**	zinc finger protein 467	ENSG00000181444		+		
		5	**AR**	androgen receptor	ENSG00000169083		+++		
6		1	**NR2F1**	nuclear receptor subfamily 2, group F, member 1	ENSG00000175745		+	+	
7		1	**MSX1**	msh homeobox 1	ENSG00000163132				++
		2	**SALL1**	spalt-like transcription factor 1	ENSG00000103449		+		++
8		1	**IRX4**	iroquois homeobox 4	ENSG00000113430		++		
		2	**FOXG1**	forkhead box G1	ENSG00000176165		+++		
9	3	1	**FOXD2**	forkhead box D2	ENSG00000186564		+++		
		2	**IRF6**	interferon regulatory factor 6	ENSG00000117595		+++		
		3	**NFE2L3**	nuclear factor, erythroid 2-like 3	ENSG00000050344		+		
		4	**FLI1**	Fli-1 proto-oncogene, ETS transcription factor	ENSG00000151702		+		
		5	**HHEX**	hematopoietically expressed homeobox	ENSG00000152804		+		
		6	**TBX5**	T-box 5	ENSG00000089225		+		

	3	7	**SIM2**	single-minded family bHLH transcription factor 2	ENSG00000159263		++	
**10**		1	**HAND2**	heart and neural crest derivatives expressed 2	ENSG00000164107		+	+
		2	**NKX2-5**	NK2 homeobox 5	ENSG00000183072		+	++
		3	**TFAP2A**	transcription factor AP-2 alpha (activating enhancer binding protein 2 alpha)	ENSG00000137203			+++
		4	**HEY2**	hes-related family bHLH transcription factor with YRPW motif 2	ENSG00000135547			+++
**11**		1	**PAX7**	paired box 7	ENSG00000009709		+++	
		2	**MEIS1**	Meis homeobox 1	ENSG00000143995		+++	
		3	**EN1**	engrailed homeobox 1	ENSG00000163064		++	
		4	**DLX1**	distal-less homeobox 1	ENSG00000144355	+++		
		5	**NKX6-1**	NK6 homeobox 1	ENSG00000163623	+	++	
		6	**IRX2**	iroquois homeobox 2	ENSG00000170561		++	
		7	**IRX1**	iroquois homeobox 1	ENSG00000170549		+++	
		8	**ISL1**	ISL LIM homeobox 1	ENSG00000016082		+++	
		9	**HOXA1**	homeobox A1	ENSG00000105991		+++	
		10	**HOXA2**	homeobox A2	ENSG00000105996		+++	
		11	**HOXA3**	homeobox A3	ENSG00000105997		+++	
		12	**HOXA4**	homeobox A4	ENSG00000197576		+++	
		13	**HOXA5**	homeobox A5	ENSG00000106004		++	
		14	**NKX2-6**	NK2 homeobox 6	ENSG00000180053		+++	
		15	**DMRTA1**	DMRT-like family A1	ENSG00000176399		+++	
		16	**MYOD1**	myogenic differentiation 1	ENSG00000129152		+++	
		17	**ISL2**	ISL LIM homeobox 2	ENSG00000159556		+++	
		18	**FOXF1**	forkhead box F1	ENSG00000103241		+++	
		19	**HOXB3**	homeobox B3	ENSG00000120093		+++	
		20	**HOXB4**	homeobox B4	ENSG00000182742		+++	
		21	**HOXB5**	homeobox B5	ENSG00000120075		+++	
		22	**HOXB6**	homeobox B6	ENSG00000108511		+++	
		23	**HOXB7**	homeobox B7	ENSG00000260027		+++	
		24	**HOXB8**	homeobox B8	ENSG00000120068		+++	
		25	**NFATC2**	nuclear factor of activated T-cells, calcineurin-dep. 2	ENSG00000101096	+	++	
		26	**TBX1**	T-box 1	ENSG00000184058		+++	
		1	**PRDM16**	PR domain containing 16	ENSG00000142611	+	+	
		2	**DMBX1**	diencephalon/mesencephalon homeobox 1	ENSG00000197587	+++		
		3	**GLIS1**	GLIS family zinc finger 1	ENSG00000174332	+++		
		4	**TBX15**	T-box 15	ENSG00000092607	+++		
		5	**MYOG**	myogenin (myogenic factor 4)	ENSG00000122180	+++		
		6	**HLX**	H2.0-like homeobox	ENSG00000136630	+++		
		7	**SIX2**	SIX homeobox 2	ENSG00000170577	+++		
		8	**AFF3**	AF4/FMR2 family, member 3	ENSG00000144218	+++		
		9	**DLX2**	distal-less homeobox 2	ENSG00000115844	+++		
		10	**HOXD11**	homeobox D11	ENSG00000128713	+++		
		11	**HOXD10**	homeobox D10	ENSG00000128710	+++		
		12	**HOXD9**	homeobox D9	ENSG00000128709	+++		
		13	**HOXD8**	homeobox D8	ENSG00000175879	+++		
		14	**HOXD4**	homeobox D4	ENSG00000170166	+++		
		15	**SHOX2**	short stature homeobox 2	ENSG00000168779	+++		
		16	**NKX3-2**	NK3 homeobox 2	ENSG00000109705	+++		
		17	**PRDM8**	PR domain containing 8	ENSG00000152784	+++		
		18	**PITX1**	paired-like homeodomain 1	ENSG00000069011	+++		
		19	**EGR1**	early growth response 1	ENSG00000120738	+++		
		20	**IRF4**	interferon regulatory factor 4	ENSG00000137265	+++		
		21	**FOXF2**	forkhead box F2	ENSG00000137273	+++		
		22	**SIM1**	single-minded family bHLH transcription factor 1	ENSG00000112246	+++		
		23	**LIN28B**	lin-28 homolog B (C. elegans)	ENSG00000187772	+++		
		24	**PRDM1**	PR domain containing 1, with ZNF domain	ENSG00000057657	+++		
		25	**MYB**	v-myb avian myeloblastosis viral oncogene homolog	ENSG00000118513	+++		
		26	**MEOX2**	mesenchyme homeobox 2	ENSG00000106511	+++		
		27	**HOXA6**	homeobox A6	ENSG00000106006	+++		
		28	**HOXA7**	homeobox A7	ENSG00000122592	+++		
		29	**HOXA9**	homeobox A9	ENSG00000078399	+++		
		30	**HOXA10**	homeobox A10	ENSG00000253293	+++		
		31	**HOXA11**	homeobox A11	ENSG00000005073	+++		

12	4	32	**DLX5**	distal-less homeobox 5	ENSG00000105880	+	+	
		33	**EN2**	engrailed homeobox 2	ENSG00000164778	+++		
		34	**SHOX**	short stature homeobox	ENSG00000185960	+++		
		35	**AFF2**	AF4/FMR2 family, member 2	ENSG00000155966	++		
		36	**EGR3**	early growth response 3	ENSG00000179388	+++		
		37	**SNAI2**	snail family zinc finger 2	ENSG00000019549	+++		
		38	**HNF4G**	hepatocyte nuclear factor 4, gamma	ENSG00000164749	+++		
		39	**OSR2**	odd-skipped related transciption factor 2	ENSG00000164920	++	+	
		40	**ZFP37**	ZFP37 zinc finger protein	ENSG00000136866	+++		
		41	**LMX1B**	LIM homeobox transcription factor 1, beta	ENSG00000136944	+++		
		42	**PRRX2**	paired related homeobox 2	ENSG00000167157	++		
		43	**HOXC13**	homeobox C13	ENSG00000123364	+++		
		44	**HOXC12**	homeobox C12	ENSG00000123407	+++		
		45	**HOXC11**	homeobox C11	ENSG00000123388	+++		
		46	**HOXC10**	homeobox C10	ENSG00000180818	+++		
		47	**HOXC6**	homeobox C6	ENSG00000197757	+++		
		48	**HOXC9**	homeobox C9	ENSG00000180806	+++		
		49	**HOXC8**	homeobox C8	ENSG00000037965	+++		
		50	**HOXC4**	homeobox C4	ENSG00000198353	+++		
		51	**HOXC5**	homeobox C5	ENSG00000172789	+++		
		52	**TBX3**	T-box 3	ENSG00000135111	+++		
		53	**SMAD9**	SMAD family member 9	ENSG00000120693	+++		
		54	**SIX1**	SIX homeobox 1	ENSG00000126778	+++		
		55	**SIX4**	SIX homeobox 4	ENSG00000100625	+++		
		56	**GSC**	goosecoid homeobox	ENSG00000133937	++	+	
		57	**BCL11B**	B-cell CLL/lymphoma 11B	ENSG00000127152	+++		
		58	**IRX5**	iroquois homeobox 5	ENSG00000176842	+++		
		59	**TBX2**	T-box 2	ENSG00000121068	+++		
		60	**ZNF521**	zinc finger protein 521	ENSG00000198795	+++		
		61	**TSHZ1**	teashirt zinc finger homeobox 1	ENSG00000179981	+++		
		62	**PAX1**	paired box 1	ENSG00000125813	+++		
		63	**TSHZ2**	teashirt zinc finger homeobox 2	ENSG00000182463	+++		
		64	**ZNF536**	zinc finger protein 536	ENSG00000198597	+++		
		65	**TSHZ3**	teashirt zinc finger homeobox 3	ENSG00000121297	+++		
		66	**POU2F2**	POU class 2 homeobox 2	ENSG00000028277	+++		
13		1	**ALX4**	ALX homeobox 4	ENSG00000052850	+++		
		2	**EGR2**	early growth response 2	ENSG00000122877	+++		
		3	**DBX2**	developing brain homeobox 2	ENSG00000185610	+++		
		4	**CEBPB**	CCAAT/enhancer binding protein (C/EBP), beta	ENSG00000172216	+++		
		5	**PEG3**	paternally expressed 3	ENSG00000198300	++		
14		1	**DMRT2**	doublesex and mab-3 related transcription factor 2	ENSG00000173253			++
15			–					
16	4	1	**FOS**	FBJ murine osteosarcoma viral oncogene homolog	ENSG00000170345	+++		
		2	**TBX4**	T-box 4	ENSG00000121075	+++		
17		1	**KLF17**	Kruppel-like factor 17	ENSG00000171872	+++		
		2	**EPAS1**	endothelial PAS domain protein 1	ENSG00000116016	+++		
		3	**THRB**	thyroid hormone receptor, beta	ENSG00000151090	+++		
		4	**ZFP42**	ZFP42 zinc finger protein	ENSG00000179059	+++		
		5	**FOXQ1**	forkhead box Q1	ENSG00000164379	+++		
		6	**ZNF311**	zinc finger protein 311	ENSG00000197935	+++		
		7	**BHLHA15**	basic helix-loop-helix family, member a15	ENSG00000180535	+++		
		8	**MKX**	mohawk homeobox	ENSG00000150051	+++		
		9	**VDR**	vitamin D (1,25- dihydroxyvitamin D3) receptor	ENSG00000111424	+++		
		10	**RORA**	RAR-related orphan receptor A	ENSG00000069667	+++		
		11	**FOSB**	FBJ murine osteosarcoma viral oncogene homolog B	ENSG00000125740	+++		
		12	**MEIS3**	Meis homeobox 3	ENSG00000105419	+++		
