# Supplementary material for: Human fetal mesoangioblasts reveal tissue‐dependent transcriptional signatures
Source: Stem Cells Transl Med. 2020 Jan 23;9(5):575–89. doi: 10.1002/sctm.19-0209 (PMC7180296; doi:10.1002/sctm.19-0209)
Supplement: Supplementary file 9 — Table S3 OddRatio values (P < .05) used for the generation of star‐plots in Figure 5. [file SCT3-9-575-s009.pdf]

Supplemental Table 3. OddRatio values (p< .05) used for the generation of star-plots in Figure 5.

Inf.: infinite value (set to 150 and corresponding to an enrichment of 150x.
GOBP: gene onthology biological process

	GO_id	Term	SuperCluster				GOBP_category	Concerned genes in SuperCluster #			
			1	2	3	4		1	2	3	4
1	GO:0002027	regulation of heart rate	0	0	0	3.3853553	Cardiac Development				CASQ2;EPAS1;PDE4D;TAC1;ADM;IRX5;CACNA1G
2	GO:0003007	heart morphogenesis	2.76003263	0	4.9530125	2.78957447	Cardiac Development	DHRS3;GJA5;EFNA1;TGFB2;RARB;PLXND1;GJA1;TBX20;GATA4;SOX17;CHD7;ZFPM2;GATA3;ANKRD1;PKP2;BMP4;ALDH1A2;GATA6;BMP2		HAND2;NKX2-5;TFAP2A;HEY2;TBX5;JAG1	TGFBR3;S1PR1;COL11A1;TNNT2;TNNI1;SOX11;WNT5A;SHOX2;SFRP2;VEGFA;FZD1;NRG1;SNAI2;EYA1;DCHS1;WNT11;RBP4;TBX3;SIX1;ACTC1;TBX2
3	GO:0003015	heart process	0	0	0	3.22900618	Cardiac Development				CASQ2;TNNT2;EPAS1;MYL1;DES;THRB;PDE4D;TAC1;CAV1;ADM;SCN2B;CACNA1C;NOS1;ACTC1;CACNA1H;IRX5;CACNA1G;KCNJ2
4	GO:0003130	BMP signaling pathway involved in heart induction	7.75600907	0	0	0	Cardiac Development	BMP4;BMP2			
5	GO:0003134	endodermal-mesodermal cell signaling involved in heart induction	7.75600907	0	0	0	Cardiac Development	BMP4;BMP2			
6	GO:0003138	primary heart field specification	0	Inf	0	0	Cardiac Development		MEF2C		
7	GO:0003142	cardiogenic plate morphogenesis	Inf	0	0	0	Cardiac Development	SOX17			
8	GO:0003148	outflow tract septum morphogenesis	10.8910828	0	0	0	Cardiac Development	RARB;TBX20;ZFPM2;GATA6			
9	GO:0003150	muscular septum morphogenesis	0	51.5292398	53.9082569	0	Cardiac Development		VANGL2	HEY2	
10	GO:0003151	outflow tract morphogenesis	4.55689103	0	12.756894	5.38693117	Cardiac Development	DHRS3;GJA5;RARB;PLXND1;TBX20;SOX17;ZFPM2;GATA6		HAND2;NKX2-5;TFAP2A;HEY2	SOX11;WNT5A;SFRP2;VEGFA;FZD1;EYA1;WNT11;TBX3;SIX1;TBX2
11	GO:0003161	cardiac conduction system development	0	0	29.6632997	0	Cardiac Development			NKX2-5;TBX5	
12	GO:0003162	atrioventricular node development	0	0	80.8669725	0	Cardiac Development			NKX2-5	
13	GO:0003165	cardiac Purkinje fiber development	0	0	Inf	0	Cardiac Development			NKX2-5	
14	GO:0003167	atrioventricular bundle cell differentiation	0	0	0	Inf	Cardiac Development				TBX3
15	GO:0003168	cardiac Purkinje fiber cell differentiation	0	0	Inf	0	Cardiac Development			NKX2-5	
16	GO:0003170	heart valve development	9.56984	0	20.5689252	0	Cardiac Development	GJA5;EFNA1;TGFB2;TBX20;GATA4;GATA3;BMP2		HEY2;TBX5;JAG1	
17	GO:0003171	atrioventricular valve development	0	0	36.2592593	0	Cardiac Development			HEY2;TBX5	
18	GO:0003172	sinoatrial valve development	0	Inf	0	0	Cardiac Development		MEF2C		
19	GO:0003174	mitral valve development	10.85968254	0	0	0	Cardiac Development	GJA5;EFNA1			
20	GO:0003175	tricuspid valve development	0	0	80.8669725	0	Cardiac Development			HEY2	
21	GO:0003177	pulmonary valve development	7.75600907	0	46.6243386	0	Cardiac Development	GJA5;TBX20		HEY2;JAG1	
22	GO:0003179	heart valve morphogenesis	10.6344	0	22.4413764	0	Cardiac Development	GJA5;EFNA1;TGFB2;TBX20;GATA4;GATA3;BMP2		HEY2;TBX5;JAG1	
23	GO:0003181	atrioventricular valve morphogenesis	7.75600907	0	0	0	Cardiac Development	GATA4;BMP2			
24	GO:0003184	pulmonary valve morphogenesis	7.75600907	0	46.6243386	0	Cardiac Development	GJA5;TBX20		HEY2;JAG1	
25	GO:0003185	sinoatrial valve morphogenesis	0	Inf	0	0	Cardiac Development		MEF2C		
26	GO:0003186	tricuspid valve morphogenesis	0	0	161.7431193	0	Cardiac Development			HEY2	
27	GO:0003188	heart valve formation	13.61544586	0	0	0	Cardiac Development	GJA5;EFNA1;TBX20;GATA4			
28	GO:0003193	pulmonary valve formation	Inf	0	0	0	Cardiac Development	GJA5;TBX20			
29	GO:0003195	tricuspid valve formation	0	0	161.7431193	0	Cardiac Development			HEY2	
30	GO:0003197	endocardial cushion development	6.48894965	0	0	0	Cardiac Development	TBX20;GATA4;ERBB3;BMP4;BMP2			
31	GO:0003199	endocardial cushion to mesenchymal transition involved in heart valve formation	0	0	53.9082569	0	Cardiac Development			HEY2	
32	GO:0003205	cardiac chamber development	2.35516687	3.8116289	6.8909878	3.62245989	Cardiac Development	DHRS3;GJA5;RARB;TBX20;GATA4;ZFPM2;GATA3;PKP2;BMP4;GATA6	VANGL2;SCN5A;MEF2C	HAND2;NKX2-5;HEY2;TBX5;JAG1	LMO4;TGFBR3;COL11A1;TNNT2;TNNI1;SOX11;WNT5A;SHOX2;SFRP2;FZD1;NRG1;WNT11;RBP4;TBX3;STRA6;TBX2
33	GO:0003206	cardiac chamber morphogenesis	2.5336003	0	8.2649694	3.78270432	Cardiac Development	DHRS3;GJA5;RARB;TBX20;GATA4;ZFPM2;GATA3;PKP2;GATA6		HAND2;NKX2-5;HEY2;TBX5;JAG1	TGFBR3;COL11A1;TNNT2;TNNI1;SOX11;WNT5A;SHOX2;SFRP2;FZD1;NRG1;WNT11;RBP4;TBX3;TBX2
34	GO:0003207	cardiac chamber formation	0	0	61.7628505	0	Cardiac Development			HAND2;NKX2-5;TBX5	
35	GO:0003208	cardiac ventricle morphogenesis	0	0	14.4277504	3.08218433	Cardiac Development			HAND2;NKX2-5;HEY2;TBX5;JAG1	TGFBR3;COL11A1;TNNT2;TNNI1;SOX11;SFRP2;NRG1

36	GO:0003209	cardiac atrium morphogenesis	0	0	19.7450467	0	Cardiac Development			NKX2-5;HEY2;TBX5	
37	GO:0003211	cardiac ventricle formation	0	0	70.5901202	0	Cardiac Development			HAND2;NKX2-5;TBX5	
38	GO:0003214	cardiac left ventricle morphogenesis	0	0	29.6632997	0	Cardiac Development			HEY2;TBX5	
39	GO:0003215	cardiac right ventricle morphogenesis	9.07484076	0	55.4056604	0	Cardiac Development	TBX20;GATA4;ZFPM2;GATA3		HAND2;NKX2-5;HEY2;JAG1	
40	GO:0003218	cardiac left ventricle formation	0	0	161.7431193	0	Cardiac Development			TBX5	
41	GO:0003219	cardiac right ventricle formation	0	0	53.9082569	0	Cardiac Development			HAND2	
42	GO:0003221	right ventricular cardiac muscle tissue morphogenesis	0	0	161.7431193	0	Cardiac Development			NKX2-5	
43	GO:0003222	ventricular trabecular myocardium morphogenesis	0	0	36.2592593	0	Cardiac Development			NKX2-5;HEY2	
44	GO:0003229	ventricular cardiac muscle tissue development	0	0	12.0287212	3.89176201	Cardiac Development			NKX2-5;HEY2;TBX5	TGFBR3;COL11A1;TNNT2;TNNI1;NRG1;COL14A1
45	GO:0003230	cardiac atrium development	0	0	17.626502	0	Cardiac Development			NKX2-5;HEY2;TBX5	
46	GO:0003231	cardiac ventricle development	0	5.0498272	9.1784406	3.88974933	Cardiac Development		VANGL2;SCN5A;MEF2C	HAND2;NKX2-5;HEY2;TBX5;JAG1	LMO4;TGFBR3;COL11A1;TNNT2;TNNI1;SOX11;WNT5A;SFRP2;FZD1;NRG1;WNT11;TBX3;STRA6
47	GO:0003245	cardiac muscle tissue growth involved in heart morphogenesis	0	0	0	Inf	Cardiac Development				S1PR1
48	GO:0003253	cardiac neural crest cell migration involved in outflow tract morphogenesis	0	0	80.8669725	0	Cardiac Development			HAND2	
49	GO:0003256	regulation of transcription from RNA polymerase II promoter involved in myocardial precursor cell differentiation	0	0	0	8.18395773	Cardiac Development				GREM1;TBX2
50	GO:0003263	cardioblast proliferation	0	0	46.6243386	7.01440922	Cardiac Development			HAND2;TBX5	EYA1;SIX1
51	GO:0003264	regulation of cardioblast proliferation	0	0	46.6243386	7.01440922	Cardiac Development			HAND2;TBX5	EYA1;SIX1
52	GO:0003266	regulation of secondary heart field cardioblast proliferation	0	0	46.6243386	7.01440922	Cardiac Development			HAND2;TBX5	EYA1;SIX1
53	GO:0003278	apoptosis involved in heart morphogenesis	0	0	326.4814815	0	Cardiac Development			HAND2;NKX2-5	
54	GO:0003279	cardiac septum development	4.17471502	0	10.3579009	3.29166667	Cardiac Development	DHRS3;GJA5;RARB;TBX20;GATA4;ZFPM2;GATA3;BMP4;GATA6		NKX2-5;HEY2;TBX5;JAG1	LMO4;SOX11;WNT5A;FZD1;WNT11;TBX3;STRA6;TBX2
55	GO:0003281	ventricular septum development	0	0	11.7416555	4.54697884	Cardiac Development			NKX2-5;HEY2;TBX5	LMO4;SOX11;WNT5A;FZD1;WNT11;TBX3;STRA6
56	GO:0003283	atrial septum development	5.82387009	0	35.2810414	0	Cardiac Development	GJA5;TBX20;GATA4		NKX2-5;HEY2;TBX5	
57	GO:0003285	septum secundum development	0	0	80.8669725	0	Cardiac Development			NKX2-5	
58	GO:0003294	atrial ventricular junction remodeling	Inf	0	0	0	Cardiac Development	GJA5;GJA1			
59	GO:0003300	cardiac muscle hypertrophy	0	7.9813932	0	0	Cardiac Development		PDE5A;MEF2C		
60	GO:0003308	negative regulation of Wnt receptor signaling pathway involved in heart development	18.1015873	0	0	0	Cardiac Development	SOX17;BMP2			
61	GO:0003342	proepicardium development	0	0	161.7431193	0	Cardiac Development			NKX2-5	
62	GO:0003343	septum transversum development	0	0	161.7431193	0	Cardiac Development			NKX2-5	
63	GO:0003350	pulmonary myocardium development	0	0	80.8669725	0	Cardiac Development			NKX2-5	
64	GO:0007507	heart development	2.57978334	0	3.4573453	1.84074438	Cardiac Development	DHRS3;ECE1;HSPG2;GJA5;EFNA1;TGFB2;ITGA4;ERBB4;OXTR;RARB;PLXND1;MECOM;PDLIM3;EDN1;GJA1;HDAC9;TBX20;GATA4;SOX17;CHD7;ZFPM2;WT1;GATA3;ANKRD1;PKP2;ERBB3;EFNB2;AKAP6;BMP4;ALDH1A2;ALPK3;RAMP2;ITGA3;GATA6;BMP2;JPH2;PDGFB;CXADR		HAND2;NKX2-5;TFAP2A;PLN;HEY2;HHEX;TBX5;CRIP1;JAG1	LMO4;TGFBR3;S1PR1;COL11A1;TNNT2;TNNI1;AGT;SOX11;XIRP1;WNT5A;SHOX2;EDNRA;SFRP2;PDGFRB;VEGFA;FZD1;NRG1;SNAI2;EYA1;COL14A1;DCHS1;ADM;WNT11;RBP4;TBX3;SIX1;GREM1;ACTC1;STRA6;TBX2;MB
65	GO:0007512	adult heart development	0	0	23.3029101	0	Cardiac Development			HAND2;NKX2-5	
66	GO:0008016	regulation of heart contraction	1.97878372	0	3.7744788	3.40432378	Cardiac Development	GJA5;TGFB2;FKBP1B;EDN1;GJA1;CTGF;PIK3CG;DMD;CELF2		NKX2-5;PLN;HEY2	CASQ2;TNNT2;EPAS1;DES;THRB;PDE4D;TAC1;CAV1;ADM;SCN2B;CACNA1C;NOS1;CACNA1H;IRX5;CACNA1G;KCNJ2
67	GO:0010002	cardioblast differentiation	4.79528664	0	18.1203704	4.33613445	Cardiac Development	TGFB2;GATA4;GATA6		NKX2-5;TBX5	NRG1;GREM1;TBX2
68	GO:0010659	cardiac muscle cell apoptosis	0	0	32.9271028	7.03179191	Cardiac Development			HAND2;NKX2-5;HEY2	SFRP2;CAMK2A;FNDC1;NRG1
69	GO:0010665	regulation of cardiac muscle cell apoptosis	0	0	41.1658879	8.9511298	Cardiac Development			HAND2;NKX2-5;HEY2	SFRP2;CAMK2A;FNDC1;NRG1
70	GO:0010666	positive regulation of cardiac muscle cell apoptosis	0	0	0	9.82132565	Cardiac Development				CAMK2A;FNDC1
71	GO:0010667	negative regulation of cardiac muscle cell apoptosis	0	0	98.8373832	8.18395773	Cardiac Development			HAND2;NKX2-5;HEY2	SFRP2;NRG1

72	GO:0014032	neural crest cell development	3.71732501	0	10.4895605	4.70736434	Cardiac Development	ERBB4;GBX2;EDN1;RDH10;ALDH1A2;LAMA5		HAND2;TFAP2A;JAG1	SOX11;EDNRA;GDNF;NRG1;SNAI2;RET;KITLG;EDNRB
73	GO:0014033	neural crest cell differentiation	3.14395429	0	8.9597281	4.54398004	Cardiac Development	ERBB4;GBX2;EDN1;RDH10;ALDH1A2;LAMA5		HAND2;TFAP2A;JAG1	SOX11;EDNRA;GDNF;NRG1;SNAI2;RET;KITLG;EDNRB;GSC
74	GO:0014896	muscle hypertrophy	0	7.4100295	0	0	Cardiac Development		PDE5A;MEF2C		
75	GO:0030510	regulation of BMP signaling pathway	3.51641825	0	0	3.58642836	Cardiac Development	BMPER;GATA4;GDF2;C10orf54;BMP4;SMAD6;PCSK6;ITGA3;FSTL3			GREM2;SOX11;WNT5A;SFRP2;FZD1;CAV1;CHRDL1;HTRA1;GREM1;RGMA
76	GO:0030514	negative regulation of BMP signaling pathway	0	0	0	5.49386305	Cardiac Development				GREM2;WNT5A;SFRP2;FZD1;CAV1;CHRDL1;HTRA1;GREM1
77	GO:0035050	embryonic heart tube development	0	0	6.7435668	0	Cardiac Development			HAND2;NKX2-5;HHEX	
78	GO:0035051	cardiac cell differentiation	2.43268757	0	7.9482993	2.47011662	Cardiac Development	TGFB2;RARB;GATA4;SOX17;WT1;EFNB2;AKAP6;GATA6;BMP2		HAND2;NKX2-5;HEY2;TBX5;JAG1	AGT;XIRP1;PDGFRB;VEGFA;NRG1;COL14A1;TBX3;GREM1;ACTC1;TBX2
79	GO:0035054	embryonic heart tube anterior/posterior pattern specification	54.31111111	0	0	0	Cardiac Development	GATA4;BMP2			
80	GO:0035295	tube development	2.81959321	0	4.1190008	2.85380551	Cardiac Development	WNT4;TACSTD2;GJA5;MYCN;GDF7;PKDCC;CXCR4;STAT1;GBX2;WNT7A;RARB;PLXND1;SHROOM3;SPRY1;RXFP1;EDN1;DDR1;GJA1;CTGF;TCF21;PDGFA;BMPER;TBX20;TNS3;GATA4;SOX17;CHD7;RDH10;FZD6;ZFPM2;LHX2;WT1;PGR;GATA3;GDF2;COL4A1;BMP4;ALDH1A2;SMAD6;CDH1;LHX1;ITGA3;GATA6;BMP2;LAMA5;FSTL3;CECR2		KDR;HAND2;NKX2-5;TFAP2A;PODXL;NRARP;HHEX;ACVRL1;TBX5;FLT1;JAG1;SIM2	LMO4;CHI3L1;AGT;KIF26B;SOX11;SDC1;SIX2;EPAS1;HOXD11;THRB;WNT5A;DPPA4;PROM1;AREG;EDNRA;SFRP2;GDNF;PDE4D;FGF18;ADAMTS2;VEGFA;SIM1;HOXA11;FZD1;MET;EYA1;RSPO2;TNC;DCHS1;ADM;INSC;WNT11;RET;RBP4;PTHLH;VDR;TBX3;SMAD9;SIX1;SIX4;PGF;GREM1;FGF7;STRA6;RGMA;CRISPLD2;TBX4;LAMA1;CRLF1;TSHZ3;CEBPA;WNT7B
81	GO:0035922	foramen ovale closure	Inf	0	0	0	Cardiac Development	GJA5;TBX20			
82	GO:0045823	positive regulation of heart contraction	0	0	17.165692	0	Cardiac Development			NKX2-5;HEY2	
83	GO:0048738	cardiac muscle tissue development	3.72996425	0	4.2271408	2.95924694	Cardiac Development	HSPG2;TGFB2;ERBB4;RARB;GJA1;TBX20;GATA4;ZFPM2;WT1;ANKRD1;PKP2;EFNB2;AKAP6;BMP4;ALDH1A2;GATA6;BMP2;JPH2;CXADR		NKX2-5;PLN;HEY2;TBX5	TGFBR3;S1PR1;COL11A1;TNNT2;TNNI1;AGT;XIRP1;WNT5A;PDGFRB;VEGFA;NRG1;COL14A1;RBP4;TBX3;GREM1;ACTC1;TBX2
84	GO:0048739	cardiac muscle fiber development	9.04920635	0	0	0	Cardiac Development	BMP4;CXADR			
85	GO:0051145	smooth muscle cell differentiation	3.16494195	0	7.0793076	5.71246221	Cardiac Development	WNT4;NTF3;BMP4;RAMP2;GATA6		PRDM6;HEY2	AGT;EREG;VEGFA;ADM;EDNRB;SIX1;TSHZ3;WNT7B;RCAN1
86	GO:0051890	regulation of cardioblast differentiation	6.27222698	0	23.3029101	0	Cardiac Development	TGFB2;GATA4;GATA6		NKX2-5;TBX5	
87	GO:0051891	positive regulation of cardioblast differentiation	40.79570747	0	108.8148148	0	Cardiac Development	TGFB2;GATA4;GATA6		NKX2-5;TBX5	
88	GO:0055005	ventricular cardiac myofibril development	0	0	80.8669725	0	Cardiac Development			NKX2-5	
89	GO:0055006	cardiac cell development	0	0	6.6447468	3.92555086	Cardiac Development			NKX2-5;HEY2	AGT;XIRP1;PDGFRB;VEGFA;COL14A1;TBX3;ACTC1
90	GO:0055007	cardiac muscle cell differentiation	2.543744	0	6.2292677	2.66671904	Cardiac Development	RARB;GATA4;WT1;EFNB2;AKAP6;GATA6;BMP2		NKX2-5;HEY2;TBX5	AGT;XIRP1;PDGFRB;NRG1;COL14A1;TBX3;GREM1;ACTC1
91	GO:0055008	cardiac muscle tissue morphogenesis	2.72073365	0	6.1418588	4.20534389	Cardiac Development	TBX20;ZFPM2;ANKRD1;PKP2;BMP2		NKX2-5;HEY2	TGFBR3;S1PR1;COL11A1;TNNT2;TNNI1;WNT5A;NRG1;ACTC1
92	GO:0055010	ventricular cardiac muscle tissue morphogenesis	0	0	8.5735867	3.51664255	Cardiac Development			NKX2-5;HEY2	TGFBR3;COL11A1;TNNT2;TNNI1;NRG1
93	GO:0055011	atrial cardiac muscle cell differentiation	0	0	80.8669725	0	Cardiac Development			NKX2-5	
94	GO:0055012	ventricular cardiac muscle cell differentiation	0	0	18.1203704	0	Cardiac Development			NKX2-5;HEY2	
95	GO:0055013	cardiac muscle cell development	0	0	6.92829	3.43822042	Cardiac Development			NKX2-5;HEY2	AGT;XIRP1;PDGFRB;COL14A1;TBX3;ACTC1
96	GO:0055014	atrial cardiac muscle cell development	0	0	80.8669725	0	Cardiac Development			NKX2-5	
97	GO:0055015	ventricular cardiac muscle cell development	0	0	29.6632997	0	Cardiac Development			NKX2-5;HEY2	
98	GO:0055017	cardiac muscle tissue growth	4.34381818	0	10.2704439	3.92555086	Cardiac Development	TGFB2;ERBB4;GJA1;TBX20;GATA4;AKAP6;GATA6		NKX2-5;HEY2;TBX5	TGFBR3;S1PR1;AGT;NRG1;COL14A1;RBP4;TBX2
99	GO:0055018	regulation of cardiac muscle fiber development	Inf	0	0	0	Cardiac Development	BMP4			
100	GO:0055020	positive regulation of cardiac muscle fiber development	Inf	0	0	0	Cardiac Development	BMP4			
101	GO:0055021	regulation of cardiac muscle tissue growth	5.84698768	0	15.9179982	3.27842004	Cardiac Development	ERBB4;GJA1;TBX20;GATA4;AKAP6;GATA6		NKX2-5;HEY2;TBX5	NRG1;COL14A1;RBP4;TBX2

102	GO:0055024	regulation of cardiac muscle tissue development	7.62459807	0	11.4679418	3.00095302	Cardiac Development	ERBB4;GJA1;TBX20;GATA4;EFNB2;AKAP6;BMP4;GATA6;BMP2;JPH2		NKX2-5;HEY2;TBX5	NRG1;COL14A1;RBP4;GREM1;TBX2
103	GO:0055025	positive regulation of cardiac muscle tissue development	54.31111111	0	0	0	Cardiac Development	AKAP6;BMP4			
104	GO:0055117	regulation of cardiac muscle contraction	3.08445355	0	0	2.7878589	Cardiac Development	GJA5;FKBP1B;GJA1;CTGF;PIK3CG;DMD			CASQ2;SCN2B;CACNA1C;NOS1;CACNA1G;KCNJ2
105	GO:0060038	cardiac muscle cell proliferation	5.28022261	0	14.5109951	0	Cardiac Development	TGFB2;ERBB4;GJA1;TBX20;GATA4;GATA6		NKX2-5;HEY2;TBX5	
106	GO:0060039	pericardium development	8.37628613	0	21.7481481	0	Cardiac Development	MECOM;TBX20;WT1;BMP2		HAND2;TBX5	
107	GO:0060043	regulation of cardiac muscle cell proliferation	5.92399972	0	19.7450467	0	Cardiac Development	ERBB4;GJA1;TBX20;GATA4;GATA6		NKX2-5;HEY2;TBX5	
108	GO:0060045	positive regulation of cardiac muscle cell proliferation	7.25859873	0	19.1873638	0	Cardiac Development	ERBB4;TBX20;GATA4;GATA6		HEY2;TBX5	
109	GO:0060047	heart contraction	0	0	0	3.22900618	Cardiac Development				CASQ2;TNNT2;EPAS1;MYL1;DES;THRB;PDE4D;TAC1;CAV1;ADM;SCN2B;CACNA1C;NOS1;ACTC1;CACNA1H;IRX5;CACNA1G;KCNJ2
110	GO:0060048	cardiac muscle contraction	0	0	0	2.63100775	Cardiac Development				CASQ2;MYL1;SCN2B;CACNA1C;NOS1;ACTC1;CACNA1G;KCNJ2
111	GO:0060070	canonical Wnt receptor signaling pathway	2.55607979	3.0176715	3.1609216	2.59692431	Cardiac Development	WNT4;FZD5;WNT7A;NKD2;MCC;ANKRD6;GATA4;SOX17;FZD6;GATA3;BAMBI;BICC1;CAPRIN2;FOXO1;GPRC5B;CDH1;JUP;BMP2	MLLT3;FZD4;LGR5;SOST	NKX2-5;RSPO3;NRARP;AMER2	PTPRU;PPAP2B;WNT9A;SDC1;WNT5A;PPP2R3A;SFRP2;EGR1;FZD1;DLX5;CAV1;SNAI2;RSPO2;ROR2;WNT11;WNT5B;WNT10B;GREM1;CDH3;WNT7B
112	GO:0060314	regulation of ryanodine-sensitive calcium-release channel activity	6.81379585	0	0	0	Cardiac Development	FKBP1B;DMD;JPH1;AKAP6;JPH2			
113	GO:0060317	cardiac epithelial to mesenchymal transition	5.09529014	0	0	0	Cardiac Development	EFNA1;TGFB2;BMP2			
114	GO:0060347	heart trabecula formation	0	0	25.0968661	0	Cardiac Development			NKX2-5;HEY2	
115	GO:0060371	regulation of atrial cardiomyocyte membrane depolarization	9.04920635	0	0	8.18395773	Cardiac Development	GJA5;GJA1			SCN2B;CACNA1G
116	GO:0060373	regulation of ventricular cardiomyocyte membrane depolarization	10.85968254	25.7602339	0	0	Cardiac Development	GJA5;GJA1	SCN5A		
117	GO:0060379	cardiac muscle cell myoblast differentiation	0	0	0	8.19432419	Cardiac Development				NRG1;GREM1;TBX2
118	GO:0060411	cardiac septum morphogenesis	4.44509767	0	14.4257588	3.35988142	Cardiac Development	DHRS3;GJA5;RARB;TBX20;GATA4;ZFPM2;GATA6		NKX2-5;HEY2;TBX5;JAG1	SOX11;WNT5A;FZD1;WNT11;TBX3;TBX2
119	GO:0060412	ventricular septum morphogenesis	0	0	12.0740741	5.1317535	Cardiac Development			NKX2-5;HEY2	SOX11;WNT5A;FZD1;WNT11;TBX3
120	GO:0060413	atrial septum morphogenesis	7.41349906	0	44.9107901	0	Cardiac Development	GJA5;TBX20;GATA4		NKX2-5;HEY2;TBX5	
121	GO:0060419	heart growth	4.28808446	0	8.7992323	3.32005694	Cardiac Development	TGFB2;ERBB4;GJA1;TBX20;GATA4;WT1;AKAP6;GATA6		NKX2-5;HEY2;TBX5	TGFBR3;S1PR1;AGT;NRG1;COL14A1;RBP4;TBX2
122	GO:0060420	regulation of heart growth	5.79549091	0	13.3321546	0	Cardiac Development	ERBB4;GJA1;TBX20;GATA4;WT1;AKAP6;GATA6		NKX2-5;HEY2;TBX5	
123	GO:0060421	positive regulation of heart growth	13.57539683	0	0	0	Cardiac Development	WT1;AKAP6			
124	GO:0060914	heart formation	0	0	19.1873638	0	Cardiac Development			HAND2;TBX5	
125	GO:0060922	atrioventricular node cell differentiation	0	0	161.7431193	0	Cardiac Development			NKX2-5	
126	GO:0060927	pacemaker cell fate commitment	0	0	Inf	0	Cardiac Development			NKX2-5	
127	GO:0060928	atrioventricular node cell development	0	0	161.7431193	0	Cardiac Development			NKX2-5	
128	GO:0060929	atrioventricular node cell fate commitment	0	0	Inf	0	Cardiac Development			NKX2-5	
129	GO:0060932	His-Purkinje system cell differentiation	0	0	161.7431193	0	Cardiac Development			NKX2-5	
130	GO:0060973	cell migration involved in heart development	0	0	46.6243386	7.01440922	Cardiac Development			HAND2;TBX5	PDGFRB;SNAI2
131	GO:0061026	cardiac muscle tissue regeneration	Inf	0	0	0	Cardiac Development	ERBB4			
132	GO:0061307	cardiac neural crest cell differentiation involved in heart development	0	0	54.3981481	0	Cardiac Development			HAND2;JAG1	
133	GO:0061308	cardiac neural crest cell development involved in heart development	0	0	65.2814815	0	Cardiac Development			HAND2;JAG1	
134	GO:0061309	cardiac neural crest cell development involved in outflow tract morphogenesis	0	0	81.6064815	0	Cardiac Development			HAND2;JAG1	
135	GO:0061311	cell surface receptor linked signaling pathway involved in heart development	0	0	18.2803738	0	Cardiac Development			HAND2;HEY2;JAG1	
136	GO:0061314	Notch signaling involved in heart development	0	0	40.7939815	0	Cardiac Development			HEY2;JAG1	

137	GO:0061325	cell proliferation involved in outflow tract morphogenesis	0	0	53.9082569	0	Cardiac Development			HAND2	
138	GO:0061337	cardiac conduction	9.04920635	0	0	0	Cardiac Development	GJA5;GJA1			
139	GO:0061341	non-canonical Wnt receptor signaling pathway involved in heart development	0	77.2982456	0	0	Cardiac Development		VANGL2		
140	GO:0061383	trabecula morphogenesis	0	0	9.8754209	4.10395562	Cardiac Development			NKX2-5;HEY2	TGFBR3;S1PR1;NRG1;RBP4;WNT10B
141	GO:0061384	heart trabecular morphogenesis	0	0	14.1771337	4.68593449	Cardiac Development			NKX2-5;HEY2	TGFBR3;S1PR1;NRG1;RBP4
142	GO:0071527	semaphorin-plexin signaling pathway involved in outflow tract morphogenesis	0	0	Inf	0	Cardiac Development			HAND2	
143	GO:0072358	cardiovascular system development	3.19180136	0	4.675643	2.35538268	Cardiac Development	NPPB;DHRS3;ECE1;HSPG2;WNT4;GJA4;ZC3H12A;TAL1;F3;GJA5;NPR1;EFNA1;CD34;TGFB2;CYP1B1;IL1A;IL1B;CXCR4;ITGA4;STAT1;FZD5;ERBB4;GBX2;ACKR3;OXTR;WNT7A;RARB;COL8A1;PLXND1;MECOM;APOD;CXCL8;PDLIM3;C6;EDN1;GJA1;CTGF;TCF21;PDGFA;HDAC9;ITGB8;IL6;BMPER;TBX20;PIK3CG;GATA4;SOX17;CHD7;ZFPM2;WT1;GATA3;GDF2;ACTA2;ANKRD1;PKP2;MMP19;ERBB3;FOXO1;EFNB2;COL4A1;COL4A2;AKAP6;BMP4;RHOJ;NRXN3;ALDH1A2;ALPK3;MFGE8;CDH13;CCL2;RAMP2;ITGA3;GATA6;BMP2;FOXS1;JPH2;PTGIS;LAMA5;C3;LYL1;CEACAM1;APOE;PDGFB;CXADR		EPHB2;TIE1;KDR;HAND2;NKX2-5;TFAP2A;PLN;HEY2;NRCAM;TSPAN12;EGFL7;NRARP;MMRN2;HHEX;ACVRL1;PTPRB;TBX5;FLT1;CRIP1;CDH5;JAG1	COL8A2;PPAP2B;LMO4;TGFBR3;S1PR1;COL11A1;PTGS2;TNNT2;TNNI1;CHI3L1;AGT;SOX11;EPAS1;TGFA;SCG2;XIRP1;WNT5A;EPHB1;SHOX2;PF4;EPGN;EREG;HPSE;EDNRA;SFRP2;THBS4;EGR1;PDGFRB;FGF18;VEGFA;LAMA4;MEOX2;HOXA7;FZD1;CAV1;ANGPT2;EGR3;NRG1;SNAI2;EYA1;ANGPT1;COL14A1;HAS2;COL15A1;PRRX2;DCHS1;ADM;PDE3B;WNT11;RBP4;TBX3;SIX1;PGF;GREM1;ACTC1;RORA;STRA6;ANPEP;EMP2;GPR56;NXN;SERPINF1;PLXDC1;TBX2;TBX4;SOCS3;LAMA1;SERPINB7;HMOX1;MB;WNT7B;ADM2
144	GO:0072513	positive regulation of secondary heart field cardioblast proliferation	0	0	53.9082569	24.55763689	Cardiac Development			TBX5	EYA1;SIX1
145	GO:2000137	negative regulation of cell proliferation involved in heart morphogenesis	Inf	0	0	0	Cardiac Development	BMP4			
146	GO:2000722	regulation of cardiac vascular smooth muscle cell differentiation	0	0	161.7431193	0	Cardiac Development			HEY2	
147	GO:2000723	negative regulation of cardiac vascular smooth muscle cell differentiation	0	0	Inf	0	Cardiac Development			HEY2	
148	GO:2000725	regulation of cardiac muscle cell differentiation	6.27222698	0	0	5.67166167	Cardiac Development	EFNB2;AKAP6;BMP2			NRG1;COL14A1;GREM1
149	GO:2000727	positive regulation of cardiac muscle cell differentiation	0	0	0	8.18395773	Cardiac Development				NRG1;GREM1
150	GO:2000826	regulation of heart morphogenesis	0	0	12.0740741	0	Cardiac Development			HAND2;TBX5	
1	GO:0001568	blood vessel development	4.00584308	2.3977135	6.4000333	2.98762887	Angiogenesis	NPPB;HSPG2;WNT4;GJA4;ZC3H12A;TAL1;F3;GJA5;NPR1;EFNA1;CD34;TGFB2;CYP1B1;IL1A;IL1B;CXCR4;STAT1;FZD5;GBX2;ACKR3;WNT7A;COL8A1;PLXND1;APOD;CXCL8;C6;EDN1;GJA1;CTGF;TCF21;PDGFA;HDAC9;ITGB8;IL6;BMPER;TBX20;PIK3CG;GATA4;SOX17;CHD7;ZFPM2;WT1;GDF2;ACTA2;MMP19;FOXO1;EFNB2;COL4A1;COL4A2;BMP4;NRXN3;ALDH1A2;MFGE8;CDH13;CCL2;RAMP2;GATA6;FOXS1;PTGIS;LAMA5;C3;LYL1;CEACAM1;APOE;PDGFB	OSR1;MYLK;MEF2C;ECSCR;AMOT;FZD4;ROBO4;C3AR1	EPHB2;TIE1;KDR;HAND2;NKX2-5;HEY2;NRCAM;TSPAN12;EGFL7;NRARP;MMRN2;HHEX;ACVRL1;PTPRB;TBX5;FLT1;CDH5;JAG1	COL8A2;PPAP2B;TGFBR3;S1PR1;PTGS2;CHI3L1;AGT;EPAS1;TGFA;SCG2;WNT5A;EPHB1;PF4;EPGN;EREG;HPSE;EDNRA;SFRP2;THBS4;EGR1;PDGFRB;FGF18;VEGFA;LAMA4;MEOX2;HOXA7;CAV1;ANGPT2;EGR3;EYA1;ANGPT1;HAS2;COL15A1;PRRX2;ADM;PDE3B;WNT11;TBX3;SIX1;PGF;GREM1;RORA;STRA6;ANPEP;EMP2;GPR56;SERPINF1;PLXDC1;TBX2;TBX4;SOCS3;LAMA1;SERPINB7;HMOX1;WNT7B;ADM2
2	GO:0001569	patterning of blood vessels	6.37616	0	9.3100529	0	Angiogenesis	CXCR4;GBX2;PLXND1;EDN1;TBX20;GDF2;COL4A1		NRARP;FLT1	

3	GO:0001570	vasculogenesis	3.07945806	0	19.2582211	0	Angiogenesis	ACKR3;WNT7A;SOX17;ZFPM2;WT1;GDF2;RAMP2		TIE1;KDR;NKX2-5;HEY2;EGFL7;HHEX;TBX5	
4	GO:0001885	endothelial cell development	4.0048316	0	0	3.62028603	Angiogenesis	WNT7A;ARHGEF26;MARVELD2;DMD;ICAM1			AGT;PDE4D;MET;STC1;WNT7B
5	GO:0001935	endothelial cell proliferation	4.86910096	0	6.8926887	2.14269464	Angiogenesis	F3;CD34;STAT1;GJA1;BMPER;CCL26;APOA1;GDF2;BMP4;ALDH1A2;CDH13;CCL2;BMP2;APOE;PDGFB		KDR;EGFL7;NRARP;ACVRL1	SCG2;WNT5A;THBS4;VEGFA;CAV1;EGR3;PGF;HMOX1
6	GO:0001936	regulation of endothelial cell proliferation	4.39587097	0	7.9781769	0	Angiogenesis	F3;STAT1;GJA1;CCL26;GDF2;BMP4;ALDH1A2;CDH13;CCL2;BMP2;APOE;PDGFB		KDR;EGFL7;NRARP;ACVRL1	
7	GO:0001937	negative regulation of endothelial cell proliferation	4.53423567	0	0	0	Angiogenesis	STAT1;GJA1;GDF2;APOE			
8	GO:0001938	positive regulation of endothelial cell proliferation	3.97529138	0	11.2388871	2.59160946	Angiogenesis	F3;CCL26;GDF2;BMP4;CDH13;CCL2;BMP2;PDGFB		KDR;EGFL7;NRARP;ACVRL1	SCG2;WNT5A;THBS4;VEGFA;EGR3;PGF
9	GO:0001944	vasculature development	3.90565514	2.3039914	6.1454204	2.86000494	Angiogenesis	NPPB;HSPG2;WNT4;GJA4;ZC3H12A;TAL1;F3;GJA5;NPR1;EFNA1;CD34;TGFB2;CYP1B1;IL1A;IL1B;CXCR4;STAT1;FZD5;GBX2;ACKR3;WNT7A;COL8A1;PLXND1;APOD;CXCL8;C6;EDN1;GJA1;CTGF;TCF21;PDGFA;HDAC9;ITGB8;IL6;BMPER;TBX20;PIK3CG;GATA4;SOX17;CHD7;ZFPM2;WT1;GDF2;ACTA2;MMP19;FOXO1;EFNB2;COL4A1;COL4A2;BMP4;RHOJ;NRXN3;ALDH1A2;MFGE8;CDH13;CCL2;RAMP2;GATA6;FOXS1;PTGIS;LAMA5;C3;LYL1;CEACAM1;APOE;PDGFB	OSR1;MYLK;MEF2C;ECSCR;AMOT;FZD4;ROBO4;C3AR1	EPHB2;TIE1;KDR;HAND2;NKX2-5;HEY2;NRCAM;TSPAN12;EGFL7;NRARP;MMRN2;HHEX;ACVRL1;PTPRB;TBX5;FLT1;CDH5;JAG1	COL8A2;PPAP2B;TGFBR3;S1PR1;PTGS2;CHI3L1;AGT;EPAS1;TGFA;SCG2;WNT5A;EPHB1;PF4;EPGN;EREG;HPSE;EDNRA;SFRP2;THBS4;EGR1;PDGFRB;FGF18;VEGFA;LAMA4;MEOX2;HOXA7;CAV1;ANGPT2;EGR3;EYA1;ANGPT1;HAS2;COL15A1;PRRX2;ADM;PDE3B;WNT11;TBX3;SIX1;PGF;GREM1;RORA;STRA6;ANPEP;EMP2;GPR56;SERPINF1;PLXDC1;TBX2;TBX4;SOCS3;LAMA1;SERPINB7;HMOX1;WNT7B;ADM2
10	GO:0001955	blood vessel maturation	0	0	54.3981481	0	Angiogenesis			ACVRL1;CDH5	
11	GO:0001974	blood vessel remodeling	0	8.4137766	8.8058058	0	Angiogenesis		MEF2C;TGM2	ACVRL1;JAG1	
12	GO:0002040	sprouting angiogenesis	3.27009585	0	16.4145658	2.95565217	Angiogenesis	HDAC9;BMPER;EFNB2;BMP4;CDH13;RAMP2		KDR;NRARP;MMRN2;ACVRL1;FLT1	PTGS2;VEGFA;EGR3;ANGPT1;PGF;GREM1
13	GO:0002042	cell migration involved in sprouting angiogenesis	0	0	11.6421958	3.78368164	Angiogenesis			KDR;MMRN2	PTGS2;VEGFA;EGR3;GREM1
14	GO:0002043	blood vessel endothelial cell proliferation involved in sprouting angiogenesis	13.57539683	0	81.6064815	0	Angiogenesis	BMPER;BMP4		NRARP;ACVRL1	
15	GO:0003013	circulatory system process	2.8182311	2.8795819	3.0202432	3.20965515	Angiogenesis	NPPB;ECE1;HTR1D;GJA5;NPR1;F11R;CD34;TGFB2;FKBP1B;STAT1;OXTR;EDN1;HTR1B;GJA1;CTGF;SGK1;TBX20;PIK3CG;KEL;DMD;CHD7;PTGS1;MRVI1;NAV2;P2RY2;CELF2;ACTA2;KCNMB4;ABAT;ADORA2B;SLC6A4;RAMP2;ICAM1;KCNK6;APOE;PDGFB	TBC1D8;SCN5A;PDE5A;CHRM2;AR;AMOT;C3AR1	NKX2-5;PLN;HEY2;FLI1;ACVRL1;HTR2A;YES1	CASQ2;PTGS2;TNNT2;AGT;CHRM3;EPAS1;MYL1;DES;THRB;AGTR1;MME;ADRA2C;EDNRA;NPR3;PDE4D;VEGFA;MEOX2;ELN;TAC1;CAV1;NCALD;ANGPT1;ADM;SERPING1;SCN2B;ADRA2A;CACNA1C;NTS;NOS1;EDNRB;BDKRB2;BDKRB1;ACTC1;ANPEP;CACNA1H;EMP2;IRX5;CACNA1G;ACE;KCNJ2;ADCYAP1;HMOX1;ADM2;RCAN1
16	GO:0003018	vascular process in circulatory system	3.7418628	0	0	3.59593349	Angiogenesis	NPPB;ECE1;HTR1D;GJA5;NPR1;EDN1;HTR1B;GJA1;KEL;MRVI1;P2RY2;ACTA2;KCNMB4;ADORA2B;SLC6A4;RAMP2;ICAM1;APOE			PTGS2;AGT;CHRM3;AGTR1;ADRA2C;EDNRA;VEGFA;CAV1;ANGPT1;ADM;ADRA2A;CACNA1C;NTS;NOS1;EDNRB;BDKRB2;ACE;ADCYAP1;HMOX1
17	GO:0003158	endothelium development	4.76988691	0	7.8827493	0	Angiogenesis	GJA4;GJA5;CD34;WNT7A;ARHGEF26;MARVELD2;GJA1;DMD;SOX17;GDF2;BMP4;LAMA5;ICAM1		KDR;HEY2;ACVRL1;JAG1	
18	GO:0003176	aortic valve development	40.79570747	0	0	0	Angiogenesis	EFNA1;TBX20;GATA3			
19	GO:0003180	aortic valve morphogenesis	40.79570747	0	0	0	Angiogenesis	EFNA1;TBX20;GATA3			

20	GO:0010573	vascular endothelial growth factor production	16.81884058	0	0	3.93526012	Angiogenesis	CYP1B1;IL1A;IL1B;ADAMTS3;IL6;GATA4;NDRG2;ADORA2B;CCL2;SULF2;C3		PTGS2;HPSE;RORA;GPR56
21	GO:0010574	regulation of vascular endothelial growth factor production	16.16417628	0	0	0	Angiogenesis	CYP1B1;IL1A;IL1B;IL6;GATA4;NDRG2;ADORA2B;CCL2;SULF2;C3		
22	GO:0010575	positive regulation vascular endothelial growth factor production	11.9651	0	0	0	Angiogenesis	CYP1B1;IL1A;IL1B;GATA4;ADORA2B;SULF2;C3		
23	GO:0010594	regulation of endothelial cell migration	3.10965361	0	5.1754058	2.80893851	Angiogenesis	EFNA1;WNT7A;EDN1;HDAC9;BMPER;GATA3;GDF2;BMP4;APOE;PDGFB	KDR;MMRN2;ACVRL1	PPAP2B;PTGS2;AGT;WNT5A;VEGFA;ANGPT2;STC1;ANGPT1;EMP2;SERPINF1
24	GO:0010595	positive regulation of endothelial cell migration	3.4067492	0	0	3.07916667	Angiogenesis	WNT7A;EDN1;HDAC9;GATA3;BMP4;PDGFB		PPAP2B;PTGS2;AGT;WNT5A;VEGFA;ANGPT1
25	GO:0016525	negative regulation of angiogenesis	2.86732806	0	8.2107477	2.59160946	Angiogenesis	NPPB;NPR1;STAT1;GDF2;COL4A2;CCL2	TIE1;MMRN2;HHEX	AGT;PF4;THBS4;ANGPT2;PDE3B;SERPINF1
26	GO:0030856	regulation of epithelial cell differentiation	3.07656194	0	0	2.39499677	Angiogenesis	STAT1;CDKN2B;AQP3;GATA3;GDF2;BMP4;LHX1;STAT5A		PROM1;GDNF;HOXA7;CAV1;VDR;WNT10B;TBX3
27	GO:0030948	negative regulation of vascular endothelial growth factor receptor signaling pathway	0	0	65.2814815	0	Angiogenesis		MMRN2;HHEX	
28	GO:0035162	embryonic hemopoiesis	4.94701795	0	0	0	Angiogenesis	TAL1;KIT;LMO2;GATA3		
29	GO:0035441	cell migration involved in vasculogenesis	0	0	80.8669725	0	Angiogenesis		TBX5	
30	GO:0035904	aorta development	0	0	13.5856481	4.47267472	Angiogenesis		HEY2;JAG1	PDGFRB;EYA1;SIX1;TBX2
31	GO:0035905	ascending aorta development	0	0	80.8669725	0	Angiogenesis		HEY2	
32	GO:0035909	aorta morphogenesis	0	0	15.5291005	5.17979921	Angiogenesis		HEY2;JAG1	PDGFRB;EYA1;SIX1;TBX2
33	GO:0035910	ascending aorta morphogenesis	0	0	80.8669725	0	Angiogenesis		HEY2	
34	GO:0035924	cellular response to vascular endothelial growth factor stimulus	0	0	13.5402387	0	Angiogenesis		KDR;MMRN2;HHEX;FLT1	
35	GO:0043534	blood vessel endothelial cell migration	2.93681231	0	7.1361235	0	Angiogenesis	EFNA1;HDAC9;APOA1;GDF2;EFNB2;APOE;PDGFB	KDR;MMRN2;ACVRL1	
36	GO:0043535	regulation of blood vessel endothelial cell migration	3.02392344	0	6.7835648	0	Angiogenesis	EFNA1;HDAC9;GDF2;APOE;PDGFB	MMRN2;ACVRL1	
37	GO:0043537	negative regulation of blood vessel endothelial cell migration	0	0	13.5856481	0	Angiogenesis		MMRN2;ACVRL1	
38	GO:0043542	endothelial cell migration	3.64666738	0	4.9646758	2.59732915	Angiogenesis	EFNA1;CYP1B1;DPP4;WNT7A;PLXND1;EDN1;HDAC9;BMPER;APOA1;GATA3;GDF2;EFNB2;BMP4;CDH13;APOE;PDGFB	KDR;PRSS3;MMRN2;ACVRL1	PTGS2;AGT;SCG2;WNT5A;VEGFA;MET;ANGPT2;EGR3;STC1;ANGPT1;GREM1;EMP2;SERPINF1
39	GO:0045446	endothelial cell differentiation	3.78802321	0	9.4668464	2.57448606	Angiogenesis	WNT7A;ARHGEF26;MARVELD2;DMD;SOX17;GDF2;BMP4;LAMA5;ICAM1	KDR;HEY2;ACVRL1;JAG1	S1PR1;AGT;PDE4D;MET;STC1;NRG1;WNT7B
40	GO:0045603	positive regulation of endothelial cell differentiation	6.78611111	0	0	0	Angiogenesis	GDF2;BMP4		
41	GO:0045765	regulation of angiogenesis	3.50800584	0	6.588835	2.48527246	Angiogenesis	NPPB;F3;NPR1;EFNA1;CD34;CYP1B1;IL1A;IL1B;STAT1;PLXND1;C6;HDAC9;IL6;GATA4;GDF2;COL4A2;CCL2;RAMP2;GATA6;PTGIS;C3	TIE1;KDR;TSPAN12;MMRN2;HHEX;ACVRL1;FLT1	PTGS2;CHI3L1;AGT;WNT5A;PF4;SFRP2;THBS4;VEGFA;ANGPT2;ADM;PDE3B;PGF;GREM1;EMP2;SERPINF1;HMOX1;ADM2
42	GO:0045766	positive regulation of angiogenesis	3.72188005	0	4.6354259	2.77695516	Angiogenesis	F3;CD34;CYP1B1;IL1A;IL1B;C6;HDAC9;GATA4;GDF2;RAMP2;GATA6;PTGIS;C3	KDR;ACVRL1;FLT1	PTGS2;CHI3L1;WNT5A;SFRP2;VEGFA;ANGPT2;ADM;PGF;GREM1;HMOX1;ADM2
43	GO:0048010	vascular endothelial growth factor receptor signaling pathway	0	0	17.0217707	0	Angiogenesis		KDR;MMRN2;HHEX;FLT1	

44	GO:0048514	blood vessel morphogenesis	4.0747614	0	6.9004372	2.97601222	Angiogenesis	NPPB;HSPG2;WNT4;ZC3H12A;TAL1;F3;GJA5;NPR1;EFNA1;CD34;TGFB2;CYP1B1;IL1A;IL1B;CXCR4;STAT1;FZD5;GBX2;ACKR3;WNT7A;COL8A1;PLXND1;APOD;CXCL8;C6;EDN1;GJA1;CTGF;TCF21;PDGFA;HDAC9;IL6;BMPER;TBX20;PIK3CG;GATA4;SOX17;CHD7;ZFPM2;WT1;GDF2;MMP19;EFNB2;COL4A1;COL4A2;BMP4;NRXN3;MFGE8;CDH13;CCL2;RAMP2;GATA6;PTGIS;LAMA5;C3;CEACAM1;APOE;PDGFB		EPHB2;TIE1;KDR;HAND2;NKX2-5;HEY2;NRCAM;TSPAN12;EGFL7;NRARP;MMRN2;HHEX;ACVRL1;PTPRB;TBX5;FLT1;JAG1	COL8A2;S1PR1;PTGS2;CHI3L1;AGT;EPAS1;TGFA;SCG2;WNT5A;EPHB1;PF4;EPGN;EREG;HPSE;EDNRA;SFRP2;THBS4;PDGFRB;FGF18;VEGFA;MEOX2;HOXA7;CAV1;ANGPT2;EGR3;EYA1;ANGPT1;HAS2;COL15A1;PRRX2;ADM;PDE3B;WNT11;SIX1;PGF;GREM1;RORA;STRA6;ANPEP;EMP2;GPR56;SERPINF1;PLXDC1;TBX2;TBX4;LAMA1;HMOX1;WNT7B;ADM2
45	GO:0048844	artery morphogenesis	0	0	10.4895605	4.70736434	Angiogenesis			HAND2;HEY2;JAG1	PDGFRB;VEGFA;EYA1;PRRX2;WNT11;SIX1;STRA6;TBX2
46	GO:0050880	regulation of blood vessel size	4.12088147	0	0	3.71840684	Angiogenesis	NPPB;ECE1;HTR1D;GJA5;NPR1;EDN1;HTR1B;GJA1;KEL;MRVI1;P2RY2;ACTA2;KCNMB4;ADORA2B;SLC6A4;ICAM1;APOE			PTGS2;AGT;CHRM3;AGTR1;ADRA2C;EDNRA;CAV1;ADM;ADRA2A;CACNA1C;NTS;NOS1;EDNRB;BDKRB2;ACE;ADCYAP1;HMOX1
47	GO:0060414	aorta smooth muscle tissue morphogenesis	0	77.2982456	0	0	Angiogenesis		MYLK		
48	GO:0060840	artery development	0	0	12.0590051	3.874601	Angiogenesis			HAND2;HEY2;ACVRL1;JAG1	PDGFRB;VEGFA;EYA1;PRRX2;WNT11;SIX1;STRA6;TBX2
49	GO:0060841	venous blood vessel development	0	0	25.0968661	0	Angiogenesis			NKX2-5;ACVRL1	
50	GO:0060948	cardiac vascular smooth muscle cell development	0	0	80.8669725	0	Angiogenesis			HEY2	
51	GO:0060976	coronary vasculature development	0	0	35.2810414	0	Angiogenesis			HAND2;HEY2;TBX5	
52	GO:0060977	coronary vasculature morphogenesis	0	0	61.7628505	0	Angiogenesis			HAND2;HEY2;TBX5	
53	GO:0060979	vasculogenesis involved in coronary vascular morphogenesis	0	0	161.7431193	0	Angiogenesis			TBX5	
54	GO:0060980	cell migration involved in coronary vasculogenesis	0	0	Inf	0	Angiogenesis			TBX5	
55	GO:0060981	cell migration involved in coronary angiogenesis	0	0	0	Inf	Angiogenesis				PDGFRB
56	GO:0061156	pulmonary artery morphogenesis	0	0	81.6064815	0	Angiogenesis			HEY2;JAG1	
57	GO:0071603	endothelial cell-cell adhesion	54.31111111	0	0	0	Angiogenesis	CYP1B1;JUP			
58	GO:0072359	circulatory system development	3.19180136	0	4.675643	2.35538268	Angiogenesis	NPPB;DHRS3;ECE1;HSPG2;WNT4;GJA4;ZC3H12A;TAL1;F3;GJA5;NPR1;EFNA1;CD34;TGFB2;CYP1B1;IL1A;IL1B;CXCR4;ITGA4;STAT1;FZD5;ERBB4;GBX2;ACKR3;OXTR;WNT7A;RARB;COL8A1;PLXND1;MECOM;APOD;CXCL8;PDLIM3;C6;EDN1;GJA1;CTGF;TCF21;PDGFA;HDAC9;ITGB8;IL6;BMPER;TBX20;PIK3CG;GATA4;SOX17;CHD7;ZFPM2;WT1;GATA3;GDF2;ACTA2;ANKRD1;PKP2;MMP19;ERBB3;FOXO1;EFNB2;COL4A1;COL4A2;AKAP6;BMP4;RHOJ;NRXN3;ALDH1A2;ALPK3;MFGE8;CDH13;CCL2;RAMP2;ITGA3;GATA6;BMP2;FOXS1;JPH2;PTGIS;LAMA5;C3;LYL1;CEACAM1;APOE;PDGFB;CXADR		EPHB2;TIE1;KDR;HAND2;NKX2-5;TFAP2A;PLN;HEY2;NRCAM;TSPAN12;EGFL7;NRARP;MMRN2;HHEX;ACVRL1;PTPRB;TBX5;FLT1;CRIP1;CDH5;JAG1	COL8A2;PPAP2B;LMO4;TGFBR3;S1PR1;COL11A1;PTGS2;TNNT2;TNNI1;CHI3L1;AGT;SOX11;EPAS1;TGFA;SCG2;XIRP1;WNT5A;EPHB1;SHOX2;PF4;EPGN;EREG;HPSE;EDNRA;SFRP2;THBS4;EGR1;PDGFRB;FGF18;VEGFA;LAMA4;MEOX2;HOXA7;FZD1;CAV1;ANGPT2;EGR3;NRG1;SNAI2;EYA1;ANGPT1;COL14A1;HAS2;COL15A1;PRRX2;DCHS1;ADM;PDE3B;WNT11;RBP4;TBX3;SIX1;PGF;GREM1;ACTC1;RORA;STRA6;ANPEP;EMP2;GPR56;NXN;SERPINF1;PLXDC1;TBX2;TBX4;SOCS3;LAMA1;SERPINB7;HMOX1;MB;WNT7B;ADM2
59	GO:0090050	positive regulation of cell migration involved in sprouting angiogenesis	0	0	0	9.82132565	Angiogenesis				PTGS2;VEGFA
60	GO:2000181	negative regulation of blood vessel morphogenesis	Inf	0	0	0	Angiogenesis	WNT4			

1	GO:0007043	cell-cell junction assembly	5.83399913	0	0	0	Cell Adhesion	GJA4;GJA5;CGN;F11R;FZD5;MARVELD2;OCLN;GJA1;MPP7;CD9;PKP2;CDH1;JUP;RAMP2;PARD6B			
2	GO:0007154	cell communication	1.73791104	1.6680664	2.032078	1.76293705	Cell Adhesion	AGRN;ARHGEF16;NPPB;DHRS3;HSPG2;WNT4;HTR1D;GJB3;GJA4;DLGAP3;TACSTD2;ROR1;GBP2;ABCA4;F3;SORT1;KCNA3;KCND3;GJA5;CGN;NPR1;EFNA1;F11R;RGS5;SELP;SELE;PIK3C2B;CD34;TRAF5;TGFB2;SIPA1L2;OR2W3;FAM110C;GDF7;FKBP1B;CYP1B1;EFEMP1;ARHGAP25;VAX2;ATP6V1B1;VAMP8;MERTK;IL1A;IL1B;LIMS2;CXCR4;TNFAIP6;PLA2R1;ITGA4;CERKL;GULP1;STAT1;STAT4;CASP10;GPR1;FZD5;ERBB4;SERPINE2;PID1;ACKR3;RAB17;OXTR;SLC6A11;NUP210;WNT7A;FGD5;KAT2B;RARB;VIPR1;ALS2CL;MST1R;SEMA3B;ARHGEF3;PLXND1;ARHGEF26;MECOM;TNIK;KLHL6;IL1RAP;APOD;ZFYVE28;D4S234E;CNGA1;KIT;EPHA5;CXCL8;FRAS1;SPRY1;RXFP1;SLC25A4;TLR3;NKD2;PLCXD3;HCN1;FST;PLK2;ARHGEF28;RASGRF2;GPR150;MCC;CSF2;SPOCK1;GFRA3;CD14;ARHGEF37;TENM2;SLIT3;STC2;NEDD [illegible]	PLCH2;LPAR3;VANGL2;RGS4;PKP1;CNIH3;OSR1;KCNK3;SCN9A;PLCL1;IGFBP2;MYRIP;UBA7;SUCNR1;ARAP2;APBB2;GABRB1;PDE5A;HHIP;PTGER4;MEF2C;NR2F1;EFNA5;NRG2;GRIK2;PLEKHG1;DOCK4;KCND2;CHRM2;EPHB6;CNKSR2;AR;FGF16;AMOT;MLLT3;FZD4;SORL1;APLP2;AKR1C3;ANK3;SRGN;C3AR1;FGD4;KIF5A;LGR5;SYT1;PELI2;RPS27L;NLRP1;SOST;PECAM1;CBLN2;TGM2;RASAL3;CELSR1	EPHB2;TIE1;KCNN3;TNFSF4;IL24;PLXNA2;IRF6;GPR39;RBP1;TNK2;KCTD8;TXK;KDR;SNCA;GRID2;LRAT;SPOCK3;HAND2;PCDH1;NKX2-5;TFAP2A;GCNT2;PLN;HEY2;RSPO3;NRCAM;TSPAN12;TRIM55;KCNB2;CDKN2A;TJP2;TLE1;SHC3;EGFL7;NRARP;GUCY1A2;ARHGAP20;NRGN;MMRN2;HHEX;ARHGDIB;ACVRL1;GRASP;TBX5;AMER2;FLT1;HTR2A;PLEK2;SIPA1L1;CRIP1;CNTNAP1;YES1;JAG1;PLVAP;GPR4;LRRC4B;CACNG7;SIM2	PRDM16;RAP1GAP;GPR3;PTPRU;TRABD2B;PCSK9;PPAP2B;DAB1;PDE4B;DIRAS3;PTGER3;PTGFR;TGFBR3;S1PR1;PTPN22;NGF;CASQ2;CRABP2;AIM2;BRINP2;ANGPTL1;RGS16;PTGS2;CHI3L1;NFASC;KCNH1;KCNK2;WNT9A;AGT;KCNK1;CHRM3;GREM2;SOX11;SDC1;ALK;EPAS1;TGFA;TRABD2A;DUSP2;IL1RL1;INHBB;ARHGEF4;SCN2A;DLX2;HOXD11;PDE1A;IGFBP5;PTPRN;SCG2;DOCK10;IRS1;CCL20;ECEL1;SRGAP3;THRB;ITGA9;CCK;PTH1R;WNT5A;CADPS;EPHA3;CBLB;BOC;MGLL;TRH;EPHB1;PPP2R3A;AGTR1;SHOX2;NLGN1;PEX5L;ADRA2C;SORCS2;GPR78;CPZ;FGFBP1;LPHN3;CXCL6;CXCL1;PF4;CXCL5;EPGN;EREG;AREG;HPSE;AGPAT9;SPARCL1;BMPR1B;UGT8;EDNRA;SFRP2;TENM3;PLEKHG4B;CTNND2;NPR3;IL7R;SLC1A3;GDNF;PDE4D;F2RL2;MCTP1;PCSK1;RGMB;CXCL14;EGR1;CXXC5;PDGFRB;CAMK2A;CD74;GRIA1;WWC1;FGF18;SNCB;IRF4;TNXB;HLA-DOA;GRM4;TREM1;VEGFA;RCAN2;HCRTR2;BMP5;EPHA7;SIM1;PRDM1;CD24;LAMA4;LAMA2;ENPP1;PDE [illegible]
3	GO:0007155	cell adhesion	3.52954972	1.9468343	4.0795921	2.91359356	Cell Adhesion	FBLIM1;WNT4;CLCA2;EFNA1;CD1D;SLAMF7;F11R;SELP;SELL;SELE;CD34;TGFB2;MYCN;CYP1B1;MERTK;FBLN7;IL1B;CNTNAP5;LIMS2;TNFAIP6;DPP4;ITGA4;SERPINE2;ACKR3;FBLN2;WNT7A;COL8A1;CD96;CLSTN2;APOD;KIT;IGFBP7;CXCL8;PCDH10;SORBS2;CDH6;HAPLN1;SPOCK1;PCDHA1;PCDHA2;PCDHA5;PCDHA8;PCDHA9;PCDHA11;PCDHA12;PCDHA13;PCDHAC1;PCDHAC2;TENM2;NEDD9;DDR1;CTGF;DACT2;ITGB8;EPDR1;PIK3CG;GPM6B;BMX;DMD;DMTN;LYN;CNTNAP3B;SVEP1;PPFIBP2;PLEKHA7;FERMT3;APOA1;SORBS1;CD9;CLSTN3;MGP;PKP2;AMIGO2;RND1;ERBB3;PLXNC1;NUAK1;PCDH20;EFNB2;NRXN3;JAG2;SMAD6;ITGA11;MFGE8;MSLN;IL32;CDH8;CDH11;CDH1;ADAMTS18;CDH13;VTN;CCL2;JUP;STAT5A;ITGA3;LGALS3BP;DSC3;DSC2;BMP2;CASS4;LAMA5;FSTL3;ICAM1;ICAM4;MAG;CD22;AP [illegible]	PKP1;SLC7A11;EDIL3;EFNA5;CLDN2;CNTNAP3;FZD4;CADM1;ANK3;LMO7;FES;TGM2;CELSR1	KDR;GRID2;CDH10;PCDH1;GCNT2;NRCAM;PODXL;SRPX;COL4A6;CDKN2A;EGFL7;NRARP;ESAM;FEZ1;COL13A1;CPXM2;ARHGDIB;ACVRL1;PCDH17;CDH5;CNTNAP1;CD93;CLDN14	AJAP1;PTPRU;COL8A2;PPAP2B;DAB1;S1PR1;COL11A1;CADM3;DPT;LAMC2;MYBPH;NFASC;AGT;KIF26B;COL6A3;SNED1;ITGA9;WNT5A;EPHA3;BOC;LSAMP;COL6A6;EPHB1;NLGN1;SPON2;PCDH7;HPSE;SPP1;BMPR1B;NDNF;PCDH18;SFRP2;DCHS2;TENM3;CTNND2;PDZD2;THBS4;RGMB;TNXB;PRPH2;VEGFA;COL19A1;EPHA7;CD24;LAMA4;LAMA2;SMOC2;COL28A1;GPNMB;HOXA7;CLDN4;COL26A1;CNTNAP2;NLGN4X;EGFL6;PCDH19;ANGPT2;NRG1;SNAI2;ANGPT1;COL14A1;APBA1;ROR2;ECM2;COL15A1;TNC;LAMC3;DCHS1;SPON1;PDE3B;NRXN2;FAT3;MPZL3;OPCML;IGSF9B;KIAA1462;RET;CDH23;PLAU;COL17A1;ITGB7;KITLG;SMOC1;FLRT2;GREM1;ISLR;ACAN;EMP2;GPR56;CDH3;CDH15;MFAP4;EMILIN2;LAMA1;CPXM1;FLRT3;COL5A3;EMR2;COMP;SIGLEC10;HAS1;FPR2;WNT7B;DSCAM;COL6A1;COL6A2

4	GO:0007160	cell-matrix adhesion	2.64428827	0	3.4639523	1.92334637	Cell Adhesion	WNT4;CD34;ITGA4;APOD;DDR1;CTGF;EPDR1;GPM6B;DMD;DMTN;PPFIBP2;SORBS1;ITGA11;CDH13;VTN;ITGA3;LAMA5		KDR;CDKN2A;COL13A1;ACVRL1	AGT;SNED1;EPHA3;HPSE;TNXB;VEGFA;HOXA7;ECM2;PLAU;COL17A1;ITGB7;GREM1;EMP2;COL5A3
5	GO:0007162	negative regulation of cell adhesion	3.57216478	0	7.7261905	2.39774689	Cell Adhesion	CYP1B1;SERPINE2;APOD;SPOCK1;DACT2;DMTN;APOA1;RND1;ERBB3;CDH1;ADAMTS18;CDH13;BMP2		GCNT2;PODXL;CDKN2A;ARHGDIB;ACVRL1	DAB1;VEGFA;CD24;HOXA7;ANGPT2;SNAI2;ANGPT1;TNC;PDE3B;DSCAM
6	GO:0007229	integrin-mediated signaling pathway	4.52731028	0	0	0	Cell Adhesion	LIMS2;ITGA4;NEDD9;CTGF;ITGB8;DMTN;FERMT3;APOA1;ITGA11;ADAMTS18;ITGA3;LAMA5;CEACAM1			
7	GO:0007267	cell-cell signaling	1.82361135	2.2686803	0	2.63354684	Cell Adhesion	AGRN;WNT4;HTR1D;GJA4;DLGAP3;KCNA3;KCND3;EFNA1;CD34;TGFB2;FKBP1B;MERTK;IL1B;TNFAIP6;FZD5;SERPINE2;RAB17;OXTR;SLC6A11;WNT7A;VIPR1;SEMA3B;KIT;EPHA5;SPRY1;SLC25A4;HCN1;PLK2;RASGRF2;EDN1;ALDH5A1;HTR1B;GJA1;PDGFA;IL6;CAMK2B;CCL26;FRMPD4;GPM6B;DMD;MAOA;OPHN1;GATA4;PLAT;LYN;CHD7;KCNA4;CPT1A;UCP2;ARRB1;PGR;GATA3;NTF3;CLSTN3;PKP2;KCNMB4;EFNB2;BMP4;NRXN3;ABAT;CDH8;SLC6A4;CCL2;LHX1;DLGAP1;TNFRSF11A;MBP;BMP2;KCNS1;BST2;KCNK6;APOE;GIPR;GRIN2D;CACNG8;PDGFB	LPAR3;CNIH3;KCNK3;PLCL1;MYRIP;GABRB1;MEF2C;EFNA5;GRIK2;KCND2;EPHB6;AR;FGF16;FZD4;KIF5A;SYT1;CBLN2		NGF;CASQ2;PTGS2;KCNH1;KCNK2;WNT9A;AGT;KCNK1;CHRM3;SOX11;INHBB;HOXD11;IRS1;CCL20;WNT5A;CADPS;MGLL;TRH;EPHB1;NLGN1;ADRA2C;FGFBP1;CXCL6;CXCL5;EREG;AREG;SFRP2;CTNND2;SLC1A3;GDNF;PCSK1;CXCL14;EGR1;CAMK2A;GRIA1;FGF18;SNCB;GRM4;HCRTR2;CD24;LAMA2;PDE7B;HOXA11;INHBA;ADCY1;FZD1;TAC1;BHLHA15;NPTX2;VGF;MET;LRRC4;NLGN4X;ZDHHC15;RPS6KA6;GABRA3;EGR3;NEFL;NRG1;NCALD;KCNQ3;ARC;APBA1;GABBR2;TNC;ADM;PDE3B;SLC1A2;NRXN2;SHANK2;SCN2B;IGSF9B;EGR2;KCNMA1;GRID1;RBP4;ADRA2A;CACNA1C;KCNA1;ABCC9;PTHLH;HOXC11;TBX3;NOS1;GJB2;TRPC4;SIX1;SIX4;PGF;TSHR;GPR68;GABRA5;GABRG3;GREM1;FGF7;CPEB1;SHISA9;GPR56;CACNA1G;KCNJ2;NPTX1;ADCYAP1;SNAP25;SYNDIG1;NTSR1;TNFSF9;CD70;CACNA1A;KCNN1;RASD2;KCNJ6;KCNJ15
8	GO:0010643	cell communication by chemical coupling	54.31111111	0	0	0	Cell Adhesion	GJA5;GJA1			
9	GO:0010644	cell communication by electrical coupling	6.78611111	0	0	0	Cell Adhesion	GJA5;GJA1			
10	GO:0010647	positive regulation of cell communication	2.23989948	0	2.4994213	1.93549801	Cell Adhesion	WNT4;F3;GJA5;SELP;TRAF5;TGFB2;FAM110C;GDF7;CYP1B1;IL1B;LIMS2;PLA2R1;CASP10;ERBB4;SERPINE2;ACKR3;OXTR;WNT7A;MST1R;KIT;TLR3;PLK2;CSF2;EDN1;ANKRD6;GJA1;TPD52L1;CTGF;PDGFA;CARD11;IL6;CHN2;BMPER;PIK3CG;GATA4;DMTN;LYN;LY96;GDF6;CDKN2B;TLR4;TRIM22;ARRB1;APOA1;GATA3;MPP7;BAMBI;GDF2;C10orf54;ANKRD1;SORBS1;AFAP1L2;CAPRIN2;ERBB3;AKAP6;BMP4;GPRC5B;CDH13;KSR1;LGALS9;VTN;CCL2;JUP;TNFRSF11A;BMP2;SULF2;PTGIS;C3;ICAM1;BST2;PDGFB		IL24;TXK;KDR;SNCA;HAND2;GCNT2;CDKN2A;NRARP;HHEX;ACVRL1;FLT1;HTR2A;CNTNAP1;JAG1	TGFBR3;NGF;PTGS2;CHI3L1;WNT9A;AGT;SOX11;TGFA;IGFBP5;IRS1;WNT5A;PPP2R3A;SHOX2;NLGN1;ADRA2C;FGFBP1;PF4;EPGN;EREG;HPSE;EDNRA;SFRP2;SLC1A3;EGR1;CXXC5;PDGFRB;CD74;WWC1;FGF18;GRM4;VEGFA;BMP5;CD24;LAMA2;AKAP12;INHBA;DLX5;TAC1;CAV1;NRG1;EYA1;ANGPT1;RSPO2;GAS1;ROR2;PRRX2;IGF2;SHANK2;WNT11;AKR1C2;ADRA2A;WNT10B;KITLG;KSR2;TNFRSF19;STRA6;SEMA7A;EMP2;GPR56;CDH3;CSF3;SECTM1;ADCYAP1;SNAP25;CRLF1;MEIS3;HMOX1;RASD2;WNT7B

11	GO:0010648	negative regulation of cell communication	2.1984073	0	2.4835909	2.1833075	Cell Adhesion	DHRS3;WNT4;RGS5;IL1B;STAT1;SERPINE2;PID1;MECOM;APOD;ZFYVE28;CXCL8;SPRY1;NKD2;FST;PLK2;MCC;SLIT3;HTR1B;ANKRD6;TCF21;BMPER;TBX20;CAMK2B;DUSP4;SOX17;LYN;FZD6;DEPTOR;RGS3;TLR4;ADAMTSL2;DUSP8;ARRB1;APOA1;GATA3;OPTN;BAMBI;BICC1;ERBB3;IRAK3;PAWR;FOXO1;NDRG2;SMAD6;SLC9A3R2;CDH1;SLC6A4;ITGA3;SLC9A3R1;SALL3;BMP2;SULF2;FSTL3;GIPR;PIK3IP1		SNCA;NKX2-5;TFAP2A;HEY2;TLE1;EGFL7;NRARP;MMRN2;HHEX;AMER2;HTR2A	PRDM16;TRABD2B;DAB1;TGFBR3;PTPN22;PTGS2;AGT;GREM2;TRABD2A;DUSP2;IL1RL1;DLX2;IGFBP5;IRS1;WNT5A;CBLB;MGLL;PPP2R3A;SFRP2;EGR1;CD74;GRIA1;WWC1;IRF4;BMP5;PRDM1;ENPP1;FZD1;CAV1;RPS6KA6;CHRDL1;SNAI2;CYP7B1;GAS1;ROR2;ASPN;ADM;PDE3B;SHANK2;WNT11;MMP3;DUSP5;ADRA2A;HTRA1;WNT5B;CHST11;GSC;DLK1;GREM1;RORA;CILP;NXN;NGFR;SOCS3;APCDD1;LDLRAD4;STMN3;PALM3;ZNF536;WNT7B
12	GO:0010652	positive regulation of cell communication by chemical coupling	Inf	0	0	0	Cell Adhesion	GJA5;GJA1			
13	GO:0010810	regulation of cell-substrate adhesion	2.83531702	0	5.3277541	3.04874723	Cell Adhesion	WNT4;FBLN2;COL8A1;APOD;SPOCK1;DDR1;GPM6B;DMD;DMTN;CDH13;VTN;ITGA3		KDR;GCNT2;CDKN2A;ACVRL1	EPHA3;SPP1;NDNF;VEGFA;SMOC2;HOXA7;COL26A1;EGFL6;ANGPT2;ECM2;PLAU;SMOC1;GREM1;EMP2
14	GO:0016264	gap junction assembly	16.3154213	0	0	0	Cell Adhesion	GJA5;GJA1;PKP2			
15	GO:0016337	cell-cell adhesion	3.7578475	2.5714822	4.6589155	2.04558621	Cell Adhesion	FBLIM1;WNT4;CD1D;SELP;SELE;CD34;TGFB2;MYCN;CYP1B1;IL1B;LIMS2;DPP4;ITGA4;SERPINE2;WNT7A;CLSTN2;PCDH10;CDH6;PCDHA1;PCDHA2;PCDHA5;PCDHA8;PCDHA9;PCDHA11;PCDHA12;PCDHA13;PCDHAC1;PCDHAC2;TENM2;CTGF;PIK3CG;LYN;PLEKHA7;FERMT3;APOA1;CLSTN3;MGP;PKP2;AMIGO2;PCDH20;NRXN3;CDH8;CDH11;CDH1;ADAMTS18;CDH13;CCL2;JUP;DSC3;DSC2;BMP2;FSTL3;ICAM1;ICAM4;CEACAM1;TTYH1;ARVCF;CXADR	PKP1;SLC7A11;EFNA5;CLDN2;CADM1;ANK3;LMO7;CELSR1	GRID2;CDH10;PCDH1;GCNT2;NRCAM;PODXL;NRARP;ESAM;COL13A1;PCDH17;CDH5;CD93;CLDN14	PTPRU;COL8A2;PPAP2B;DAB1;COL11A1;CADM3;NFASC;KIF26B;WNT5A;NLGN1;PCDH7;BMPR1B;PCDH18;DCHS2;TENM3;CTNND2;THBS4;TNXB;VEGFA;COL19A1;EPHA7;CD24;CLDN4;NLGN4X;PCDH19;COL14A1;ROR2;DCHS1;NRXN2;FAT3;IGSF9B;RET;CDH23;ITGB7;ACAN;CDH3;CDH15;WNT7B
16	GO:0022407	regulation of cell-cell adhesion	3.66	0	4.9651657	0	Cell Adhesion	WNT4;IL1B;DPP4;SERPINE2;LYN;FERMT3;APOA1;CDH1;ADAMTS18;CCL2;BMP2;FSTL3		GCNT2;PODXL;NRARP	
17	GO:0022408	negative regulation of cell-cell adhesion	3.89018	0	0	0	Cell Adhesion	SERPINE2;APOA1;CDH1;ADAMTS18;BMP2			
18	GO:0022409	positive regulation of cell-cell adhesion	0	0	6.92829	0	Cell Adhesion			GCNT2;PODXL	

19	GO:0022610	biological adhesion	3.56792495	1.9449208	4.0755474	2.91047222	Cell Adhesion	FBLIM1;WNT4;CLCA2;GBP2;EFNA1;CD1D;SLAMF7;F11R;SELP;SELL;SELE;CD34;TGFB2;MYCN;CYP1B1;MERTK;FBLN7;IL1B;CNTNAP5;LIMS2;TNFAIP6;DPP4;ITGA4;SERPINE2;ACKR3;FBLN2;WNT7A;COL8A1;CD96;CLSTN2;APOD;KIT;IGFBP7;CXCL8;PCDH10;SORBS2;CDH6;HAPLN1;SPOCK1;PCDHA1;PCDHA2;PCDHA5;PCDHA8;PCDHA9;PCDHA11;PCDHA12;PCDHA13;PCDHAC1;PCDHAC2;TENM2;NEDD9;DDR1;CTGF;DACT2;ITGB8;EPDR1;PIK3CG;GPM6B;BMX;DMD;DMTN;LYN;CNTNAP3B;SVEP1;PPFIBP2;PLEKHA7;FERMT3;APOA1;SORBS1;CD9;CLSTN3;MGP;PKP2;AMIGO2;RND1;ERBB3;PLXNC1;NUAK1;PCDH20;EFNB2;NRXN3;JAG2;SMAD6;ITGA11;MFGE8;MSLN;IL32;CDH8;CDH11;CDH1;ADAMTS18;CDH13;VTN;CCL2;JUP;STAT5A;ITGA3;LGALS3BP;DSC3;DSC2;BMP2;CASS4;LAMA5;FSTL3;ICAM1;ICAM4;MAG;CD[illegible]	PKP1;SLC7A11;EDIL3;EFNA5;CLDN2;CNTNAP3;FZD4;CADM1;ANK3;LMO7;FES;TGM2;CELSR1	KDR;GRID2;CDH10;PCDH1;GCNT2;NRCAM;PODXL;SRPX;COL4A6;CDKN2A;EGFL7;NRARP;ESAM;FEZ1;COL13A1;CPXM2;ARHGDIB;ACVRL1;PCDH17;CDH5;CNTNAP1;CD93;CLDN14	AJAP1;PTPRU;COL8A2;PPAP2B;DAB1;S1PR1;COL11A1;CADM3;DPT;LAMC2;MYBPH;NFASC;AGT;KIF26B;COL6A3;SNED1;ITGA9;WNT5A;EPHA3;BOC;LSAMP;COL6A6;EPHB1;NLGN1;SPON2;PCDH7;HPSE;SPP1;BMPR1B;NDNF;PCDH18;SFRP2;DCHS2;TENM3;CTNND2;PDZD2;THBS4;RGMB;TNXB;PRPH2;VEGFA;COL19A1;EPHA7;CD24;LAMA4;LAMA2;SMOC2;COL28A1;GPNMB;HOXA7;CLDN4;COL26A1;CNTNAP2;NLGN4X;EGFL6;PCDH19;ANGPT2;NRG1;SNAI2;ANGPT1;COL14A1;APBA1;ROR2;ECM2;COL15A1;TNC;LAMC3;DCHS1;SPON1;PDE3B;NRXN2;FAT3;MPZL3;OPCML;IGSF9B;KIAA1462;RET;CDH23;PLAU;COL17A1;ITGB7;KITLG;SMOC1;FLRT2;GREM1;ISLR;ACAN;EMP2;GPR56;CDH3;CDH15;MFAP4;EMILIN2;LAMA1;CPXM1;FLRT3;COL5A3;EMR2;COMP;SIGLEC10;HAS1;FPR2;WNT7B;DSCAM;COL6A1;COL6A2
20	GO:0022617	extracellular matrix disassembly	2.83071775	0	0	5.81471404	Cell Adhesion	HSPG2;DPP4;COL4A4;COL8A1;MFI2;DDR1;CTSV;MMP19;COL4A1;COL4A2;MMP15;CDH1;LAMA5			COL8A2;COL11A1;LAMC2;COL6A3;COL6A6;SPP1;COL25A1;COL23A1;COL19A1;COL10A1;ELN;COL26A1;TIMP1;COL14A1;COL15A1;MMP8;MMP3;MMP12;MMP13;COL17A1;MMP9;COL9A3;COL5A3;ADAMTS5;COL6A1;COL6A2
21	GO:0030155	regulation of cell adhesion	3.44274827	2.9303035	3.6302589	2.79718485	Cell Adhesion	WNT4;EFNA1;TGFB2;CYP1B1;IL1B;DPP4;SERPINE2;FBLN2;COL8A1;APOD;CXCL8;SPOCK1;DDR1;DACT2;PIK3CG;GPM6B;DMD;DMTN;LYN;FERMT3;APOA1;RND1;ERBB3;NUAK1;JAG2;CDH1;ADAMTS18;CDH13;VTN;CCL2;STAT5A;ITGA3;BMP2;LAMA5;FSTL3;ICAM1	EDIL3;EFNA5;FZD4;ANK3;FES;TGM2	KDR;GCNT2;PODXL;CDKN2A;NRARP;ARHGDIB;ACVRL1	PPAP2B;DAB1;S1PR1;KIF26B;WNT5A;EPHA3;SPP1;NDNF;SFRP2;VEGFA;EPHA7;CD24;LAMA4;LAMA2;SMOC2;HOXA7;COL26A1;EGFL6;ANGPT2;NRG1;SNAI2;ANGPT1;ECM2;TNC;PDE3B;RET;PLAU;SMOC1;GREM1;EMP2;GPR56;LAMA1;DSCAM
22	GO:0030198	extracellular matrix organization	3.57917076	0	2.6261417	5.49293924	Cell Adhesion	AGRN;HSPG2;F11R;TGFB2;CYP1B1;EFEMP1;DPP4;ITGA4;COL4A4;FBLN2;COL8A1;MFI2;ADAMTS3;RXFP1;HAPLN1;DDR1;CTGF;PDGFA;ITGB8;GPM6B;DMD;TNFRSF11B;CTSV;WT1;MMP19;NTN4;COL4A1;COL4A2;BMP4;ITGA11;MMP15;CDH1;VTN;RAMP2;ITGA3;TTR;BMP2;SULF2;LAMA5;ICAM1;ICAM4;APLP1;PDGFB		MFAP2;KDR;SNCA;TFAP2A;COL4A6;COL13A1	COL8A2;COL24A1;COL11A1;OLFML2B;DPT;LAMC2;AGT;ERO1LB;SDC1;COL6A3;ITGA9;COL6A6;LEPREL1;SPP1;COL25A1;NDNF;SFRP2;COL23A1;ADAMTS2;FOXF2;TNXB;COL21A1;COL19A1;LAMA4;COL10A1;LAMA2;SMOC2;COL28A1;ELN;COL26A1;EGFL6;TIMP1;COL14A1;HAS2;COL22A1;ECM2;COL15A1;TNC;OLFML2A;LMX1B;LAMC3;MMP8;MMP3;MMP12;MMP13;MPZL3;MKX;ADAMTS14;KAZALD1;COL17A1;ITGB7;LUM;SMOC1;GREM1;ACAN;CRISPLD2;MFAP4;LAMA1;MMP9;COL9A3;COL5A3;COMP;HAS1;ADAMTS5;COL6A1;COL6A2

23	GO:0033622	integrin activation	6.79531002	0	0	0	Cell Adhesion	FBLIM1;SELP;FERMT3			
24	GO:0033623	regulation of integrin activation	6.78611111	0	0	0	Cell Adhesion	FBLIM1;SELP			
25	GO:0033624	negative regulation of integrin activation	0	154.6052632	0	0	Cell Adhesion		PTGER4		
26	GO:0033627	cell adhesion mediated by integrin	5.37734065	0	0	3.19671558	Cell Adhesion	EFNA1;TGFB2;CYP1B1;DPP4;PIK3CG;LYN;FERMT3;ITGA11;VTN;ICAM1			WNT5A;SFRP2;CD24;SNAI2;PDE3B;RET;PLAU
27	GO:0033628	regulation of cell adhesion mediated by integrin	4.66247805	0	0	4.21352968	Cell Adhesion	EFNA1;TGFB2;CYP1B1;DPP4;PIK3CG;LYN;FERMT3			WNT5A;SFRP2;CD24;SNAI2;PDE3B;RET;PLAU
28	GO:0033630	positive regulation of cell adhesion mediated by integrin	0	0	0	4.92052023	Cell Adhesion				WNT5A;SFRP2;CD24;RET
29	GO:0033632	regulation of cell-cell adhesion mediated by integrin	7.75600907	0	0	0	Cell Adhesion	DPP4;FERMT3			
30	GO:0034329	cell junction assembly	4.31846989	0	5.1319662	1.84062831	Cell Adhesion	FBLIM1;WNT4;GJA4;GJA5;CGN;F11R;LIMS2;FZD5;APOD;CDH6;MARVELD2;OCLN;GJA1;GPM6B;DMTN;MPP7;SORBS1;CD9;PKP2;CDH8;CDH11;CDH1;CDH13;JUP;RAMP2;PARD6B;LAMA5		KDR;CDH10;ACVRL1;CDH5;CNTNAP1;CLDN14	CADM3;LAMC2;NFASC;EPHA3;UGT8;VEGFA;SNAI2;WNT11;COL17A1;GJB2;GREM1;CDH3;CDH15;EPB41L3
31	GO:0034331	cell junction maintenance	11.65250965	0	0	0	Cell Adhesion	SHROOM2;PLEKHA7;PKP2			
32	GO:0043062	extracellular structure organization	3.56852302	0	2.6190789	5.47544643	Cell Adhesion	AGRN;HSPG2;F11R;TGFB2;CYP1B1;EFEMP1;DPP4;ITGA4;COL4A4;FBLN2;COL8A1;MFI2;ADAMTS3;RXFP1;HAPLN1;DDR1;CTGF;PDGFA;ITGB8;GPM6B;DMD;TNFRSF11B;CTSV;WT1;MMP19;NTN4;COL4A1;COL4A2;BMP4;ITGA11;MMP15;CDH1;VTN;RAMP2;ITGA3;TTR;BMP2;SULF2;LAMA5;ICAM1;ICAM4;APLP1;PDGFB		MFAP2;KDR;SNCA;TFAP2A;COL4A6;COL13A1	COL8A2;COL24A1;COL11A1;OLFML2B;DPT;LAMC2;AGT;ERO1LB;SDC1;COL6A3;ITGA9;COL6A6;LEPREL1;SPP1;COL25A1;NDNF;SFRP2;COL23A1;ADAMTS2;FOXF2;TNXB;COL21A1;COL19A1;LAMA4;COL10A1;LAMA2;SMOC2;COL28A1;ELN;COL26A1;EGFL6;TIMP1;COL14A1;HAS2;COL22A1;ECM2;COL15A1;TNC;OLFML2A;LMX1B;LAMC3;MMP8;MMP3;MMP12;MMP13;MPZL3;MKX;ADAMTS14;KAZALD1;COL17A1;ITGB7;LUM;SMOC1;GREM1;ACAN;CRISPLD2;MFAP4;LAMA1;MMP9;COL9A3;COL5A3;COMP;HAS1;ADAMTS5;COL6A1;COL6A2
33	GO:0045168	cell-cell signaling involved in cell fate commitment	3.40291069	0	0	3.07615774	Cell Adhesion	WNT4;FZD5;SPRY1;BMP4;BMP2			HOXD11;GDNF;HOXA11;HOXC11;SIX1
34	GO:0045216	cell-cell junction organization	5.49622519	0	4.6476216	0	Cell Adhesion	GJA4;GJA5;CGN;F11R;TGFB2;LIMS2;FZD5;CDH6;MARVELD2;OCLN;GJA1;SHROOM2;PLEKHA7;MPP7;CD9;PKP2;CDH8;CDH11;CDH1;CDH13;JUP;RAMP2;PARD6B;CXADR		CDH10;CDH5;CNTNAP1;CLDN14	
35	GO:0045217	cell-cell junction maintenance	9.04920635	0	0	0	Cell Adhesion	SHROOM2;PLEKHA7			
36	GO:0045720	negative regulation of integrin biosynthetic process	0	Inf	0	0	Cell Adhesion		AR		
37	GO:0045726	positive regulation of integrin biosynthetic process	0	77.2982456	0	0	Cell Adhesion		AR		
38	GO:0045785	positive regulation of cell adhesion	1.91259218	0	0	2.99026549	Cell Adhesion	WNT4;TGFB2;IL1B;FBLN2;COL8A1;DMD;CDH13;VTN;CCL2;ITGA3;FSTL3			PPAP2B;KIF26B;WNT5A;SPP1;NDNF;SFRP2;VEGFA;CD24;SMOC2;COL26A1;EGFL6;NRG1;ANGPT1;ECM2;RET;SMOC1;EMP2;GPR56
39	GO:0048041	focal adhesion assembly	3.14395429	0	0	0	Cell Adhesion	WNT4;APOD;GPM6B;DMTN;SORBS1;LAMA5			
40	GO:0051042	negative regulation of calcium-independent cell-cell adhesion	Inf	0	0	0	Cell Adhesion	BMP2			
41	GO:0051893	regulation of focal adhesion assembly	3.19876358	0	9.0509259	0	Cell Adhesion	WNT4;APOD;GPM6B;DMTN		KDR;ACVRL1	
42	GO:0060352	cell adhesion molecule production	27.15396825	0	0	0	Cell Adhesion	IL1B;APOA1			
43	GO:0060353	regulation of cell adhesion molecule production	Inf	0	0	0	Cell Adhesion	IL1B;APOA1			
44	GO:0070830	tight junction assembly	7.75300384	0	0	0	Cell Adhesion	CGN;F11R;FZD5;MARVELD2;OCLN;GJA1;MPP7;PKP2;CDH1;RAMP2;PARD6B			
45	GO:0090109	regulation of cell-substrate junction assembly	3.19876358	0	9.0509259	0	Cell Adhesion	WNT4;APOD;GPM6B;DMTN		KDR;ACVRL1	

46	GO:2001046	positive regulation of integrin-mediated signaling pathway	13.57539683	0	0	0	Cell Adhesion	LIMS2;DMTN			
1	GO:0003012	muscle system process	2.34813644	0	0	2.87647647	Skeletal Muscle Development	HTR1D;GJA5;LMOD1;FKBP1B;IL1B;OXTR;VIPR1;EDN1;GJA1;CTGF;TBX20;CAMK2B;PIK3CG;DMD;GATA4;MRVI1;ACTA2;SORBS1;AKAP6;MYH13;MYH8;MYH4;MYH2;ADORA2B;GATA6;SULF2			CASQ2;PTGS2;CACNA1S;TNNT2;TNNI1;MYOG;MYBPH;AGT;CHRM3;MYL1;DES;ADRA2C;EDNRA;GDNF;PDE4D;CAV1;COL14A1;SNTB1;TNNT3;SCN2B;KCNMA1;ADRA2A;CACNA1C;NOS1;EDNRB;BDKRB2;ACTC1;CACNA1H;MYH3;CACNA1G;KCNJ2;TSHZ3;HMOX1;MB
2	GO:0006936	muscle contraction	2.19303303	0	0	3.08164703	Skeletal Muscle Development	HTR1D;GJA5;LMOD1;FKBP1B;OXTR;VIPR1;EDN1;GJA1;CTGF;TBX20;PIK3CG;DMD;MRVI1;ACTA2;SORBS1;MYH13;MYH8;MYH4;MYH2;ADORA2B;SULF2			CASQ2;PTGS2;CACNA1S;TNNT2;TNNI1;MYBPH;AGT;CHRM3;MYL1;DES;ADRA2C;EDNRA;GDNF;PDE4D;CAV1;SNTB1;TNNT3;SCN2B;KCNMA1;ADRA2A;CACNA1C;NOS1;EDNRB;BDKRB2;ACTC1;CACNA1H;MYH3;CACNA1G;KCNJ2;TSHZ3;MB
3	GO:0006937	regulation of muscle contraction	2.23854831	0	3.8039557	3.18686698	Skeletal Muscle Development	GJA5;FKBP1B;OXTR;EDN1;GJA1;CTGF;PIK3CG;DMD;MRVI1;ADORA2B		NKX2-5;PLN;KCNB2	CASQ2;PTGS2;TNNT2;TNNI1;MYBPH;CHRM3;ADRA2C;CAV1;TNNT3;SCN2B;ADRA2A;CACNA1C;NOS1;CACNA1G;KCNJ2
4	GO:0006941	striated muscle contraction	0	0	0	3.28039064	Skeletal Muscle Development				CASQ2;CACNA1S;TNNI1;MYBPH;MYL1;TNNT3;SCN2B;CACNA1C;NOS1;ACTC1;MYH3;CACNA1G;KCNJ2;MB
5	GO:0006942	regulation of striated muscle contraction	2.55266573	0	0	3.64750519	Skeletal Muscle Development	GJA5;FKBP1B;GJA1;CTGF;PIK3CG;DMD			CASQ2;TNNI1;MYBPH;TNNT3;SCN2B;CACNA1C;NOS1;CACNA1G;KCNJ2
6	GO:0007517	muscle organ development	2.71567677	0	0	3.65921213	Skeletal Muscle Development	AGRN;SORT1;BTG2;VAMP5;TANC1;ERBB4;FAM65B;GJA1;TCF21;HDAC9;IL6;TBX20;KEL;DMD;FHL1;GATA4;SOX17;MSC;JPH1;ZFPM2;ASS1;WT1;TAGLN;PTPLA;ANKRD1;BHLHE41;PKP2;EFNB2;COL4A1;AKAP6;BMP4;ITGA11;GATA6;BMP2;JPH2;LAMA5;TNFSF14			TGFBR3;S1PR1;COL11A1;CACNA1S;TNNT2;TNNI1;MYOG;KCNH1;HLX;SOX11;HOXD10;HOXD9;IGFBP5;COL6A3;WNT5A;PDZRN3;BOC;SHOX2;PITX1;CXCL14;EGR1;COL19A1;LAMA2;MEOX2;ELN;CAV1;MET;EGR3;NRG1;COL14A1;TNC;ALX4;MKX;EGR2;RBP4;WNT10B;SCN8A;TBX3;NOS1;SGCG;SIX1;SIX4;FOS;GSC;GREM1;ACTC1;STRA6;CACNA1H;MYLPF;MYH3;TBX2;RCAN1
7	GO:0007519	skeletal muscle tissue development	2.01085646	0	0	3.59824102	Skeletal Muscle Development	AGRN;SORT1;BTG2;VAMP5;TANC1;TCF21;HDAC9;KEL;DMD;SOX17;MSC;PTPLA;ANKRD1;BHLHE41;COL4A1;BMP4;TNFSF14			CACNA1S;MYOG;KCNH1;HLX;SOX11;HOXD10;HOXD9;IGFBP5;PDZRN3;BOC;SHOX2;PITX1;CXCL14;EGR1;COL19A1;MEOX2;ELN;CAV1;MET;TNC;MKX;EGR2;WNT10B;TBX3;NOS1;SIX1;SIX4;FOS;CACNA1H;MYLPF;RCAN1
8	GO:0007520	myoblast fusion	0	0	0	7.57314362	Skeletal Muscle Development				CACNA1S;KCNH1;NOS1;CACNA1H
9	GO:0007521	muscle cell fate determination	0	154.6052632	0	0	Skeletal Muscle Development		MEF2C		
10	GO:0010656	negative regulation of muscle cell apoptosis	0	0	24.6883178	5.17979921	Skeletal Muscle Development			HAND2;NKX2-5;HEY2	SFRP2;NRG1;ADCYAP1;HMOX1
11	GO:0010657	muscle cell apoptosis	0	0	17.4707051	4.93759071	Skeletal Muscle Development			HAND2;NKX2-5;HEY2;CDKN2A	PDE1A;SFRP2;CAMK2A;FNDC1;NRG1;ADCYAP1;HMOX1
12	GO:0010658	striated muscle cell apoptosis	0	0	27.4345794	5.78986739	Skeletal Muscle Development			HAND2;NKX2-5;HEY2	SFRP2;CAMK2A;FNDC1;NRG1
13	GO:0010660	regulation of muscle cell apoptosis	0	0	19.5305216	5.57601011	Skeletal Muscle Development			HAND2;NKX2-5;HEY2;CDKN2A	PDE1A;SFRP2;CAMK2A;FNDC1;NRG1;ADCYAP1;HMOX1
14	GO:0010662	regulation of striated muscle cell apoptosis	0	0	32.9271028	7.03179191	Skeletal Muscle Development			HAND2;NKX2-5;HEY2	SFRP2;CAMK2A;FNDC1;NRG1

15	GO:0010663	positive regulation of striated muscle cell apoptosis	0	0	0	9.82132565	Skeletal Muscle Development				CAMK2A;FNDC1
16	GO:0010664	negative regulation of striated muscle cell apoptosis	0	0	54.8971963	0	Skeletal Muscle Development			HAND2;NKX2-5;HEY2	
17	GO:0014706	striated muscle tissue development	2.60821918	0	0	3.61251932	Skeletal Muscle Development	AGRN;HSPG2;SORT1;BTG2;TGFB2;VAMP5;TANC1;ERBB4;RARB;GJA1;TCF21;HDAC9;TBX20;KEL;DMD;GATA4;SOX17;MSC;ZFPM2;WT1;PTPLA;ANKRD1;BHLHE41;PKP2;EFNB2;COL4A1;AKAP6;BMP4;ALDH1A2;GATA6;BMP2;JPH2;TNFSF14;CXADR			TGFBR3;S1PR1;COL11A1;CACNA1S;TNNT2;TNNI1;MYOG;KCNH1;HLX;AGT;SOX11;HOXD10;HOXD9;IGFBP5;XIRP1;WNT5A;PDZRN3;BOC;SHOX2;PITX1;CXCL14;EGR1;PDGFRB;VEGFA;COL19A1;MEOX2;ELN;CAV1;MET;NRG1;EYA1;COL14A1;TNC;MKX;EGR2;RBP4;WNT10B;TBX3;NOS1;SIX1;SIX4;FOS;GREM1;ACTC1;CACNA1H;MYLPF;TBX2;EYA2;RCAN1
18	GO:0014707	branchiomeric skeletal muscle development	54.31111111	0	0	0	Skeletal Muscle Development	TCF21;MSC			
19	GO:0014737	positive regulation of muscle atrophy	0	0	0	Inf	Skeletal Muscle Development				MYOG
20	GO:0014812	muscle cell migration	0	0	0	5.05622657	Skeletal Muscle Development				IGFBP5;PDE4D;THBS4;PDGFRB;MET;NET1;PLAU;SIX1;SIX4;ACE
21	GO:0014855	striated muscle cell proliferation	4.95962823	0	13.703271	0	Skeletal Muscle Development	TGFB2;ERBB4;GJA1;TBX20;GATA4;GATA6		NKX2-5;HEY2;TBX5	
22	GO:0014861	regulation of skeletal muscle contraction via membrane action potential	0	0	0	Inf	Skeletal Muscle Development				KCNJ2
23	GO:0014873	response to muscle activity involved in regulation of muscle adaptation	0	0	0	Inf	Skeletal Muscle Development				MYOG;AGT
24	GO:0014897	striated muscle hypertrophy	0	7.7814159	0	0	Skeletal Muscle Development		PDE5A;MEF2C		
25	GO:0014902	myotube differentiation	2.83442982	0	0	3.14486586	Skeletal Muscle Development	SORT1;TANC1;DMD;PTPLA;BHLHE41			CACNA1S;MYOG;KCNH1;MET;NOS1;CACNA1H
26	GO:0016202	regulation of striated muscle tissue development	3.72289632	0	3.9908822	3.10355572	Skeletal Muscle Development	AGRN;ERBB4;GJA1;HDAC9;TBX20;GATA4;SOX17;BHLHE41;EFNB2;AKAP6;BMP4;GATA6;BMP2;JPH2;TNFSF14		NKX2-5;HEY2;TBX5	MYOG;BOC;SHOX2;CXCL14;NRG1;COL14A1;MKX;RBP4;WNT10B;TBX3;SIX1;SIX4;GREM1;TBX2
27	GO:0030049	muscle filament sliding	3.10718835	0	0	5.40144231	Skeletal Muscle Development	DMD;MYH8;MYH4;MYH2			TNNT2;TNNI1;MYL1;DES;TNNT3;ACTC1;MYH3
28	GO:0030239	myofibril assembly	0	6.6198456	0	4.82246171	Skeletal Muscle Development		MEF2C;KRT19		CASQ2;TNNT2;XIRP1;PDGFRB;MYPN;SIX4;ACTC1;MYH3
29	GO:0033002	muscle cell proliferation	3.47098321	0	0	3.37527418	Skeletal Muscle Development	NPR1;TGFB2;STAT1;ERBB4;APOD;EDN1;GJA1;IL6;TBX20;GATA4;NDRG2;BMP4;CDH13;GATA6;PDGFB			TGFBR3;S1PR1;PTGS2;AGT;PDE1A;IGFBP5;EREG;EDNRA;NPR3;PDE4D;PDGFRB;NRG1;OGN;RBP4;TBX2;HMOX1
30	GO:0033275	actin-myosin filament sliding	3.10718835	0	0	5.40144231	Skeletal Muscle Development	DMD;MYH8;MYH4;MYH2			TNNT2;TNNI1;MYL1;DES;TNNT3;ACTC1;MYH3
31	GO:0035914	skeletal muscle cell differentiation	0	0	0	2.92711718	Skeletal Muscle Development				CACNA1S;MYOG;KCNH1;SOX11;EGR1;MET;EGR2;NOS1;SIX1;FOS;CACNA1H
32	GO:0042692	muscle cell differentiation	2.07044711	0	0	3.45631129	Skeletal Muscle Development	AGRN;WNT4;SORT1;BTG2;TANC1;RARB;EDN1;HDAC9;KEL;DMD;GATA4;WT1;PTPLA;ANKRD1;NTF3;BHLHE41;KRT8;EFNB2;COL4A1;AKAP6;BMP4;RAMP2;WFIKKN2;GATA6;BMP2;TNFSF14;CXADR			CASQ2;CACNA1S;TNNT2;MYOG;KCNH1;AGT;SOX11;SDC1;EPAS1;IGFBP5;XIRP1;PDZRN3;BOC;SHOX2;EREG;PITX1;CXCL14;EGR1;PDGFRB;VEGFA;MET;NRG1;COL14A1;TNC;IGF2;ADM;MKX;EGR2;MYPN;WNT10B;TBX3;NOS1;EDNRB;SIX1;SIX4;FOS;GREM1;ACTC1;RORA;CACNA1H;CDH15;MYH3;TBX2;TSHZ3;WNT7B;RCAN1
33	GO:0042693	muscle cell fate commitment	0	0	0	7.01440922	Skeletal Muscle Development				MYOG;TBX2
34	GO:0043500	muscle adaptation	3.02715655	0	0	0	Skeletal Muscle Development	IL1B;EDN1;CAMK2B;GATA4;AKAP6;GATA6			

35	GO:0045445	myoblast differentiation	0	0	0	4.28107155	Skeletal Muscle Development				MYOG;SDC1;EPAS1;BOC;PITX1;CXCL14;MKX;WNT10B;TBX3
36	GO:0045661	regulation of myoblast differentiation	0	0	0	3.84700615	Skeletal Muscle Development				MYOG;BOC;CXCL14;MKX;TBX3
37	GO:0045662	negative regulation of myoblast differentiation	0	0	0	4.09499759	Skeletal Muscle Development				CXCL14;MKX;TBX3
38	GO:0045844	positive regulation of striated muscle tissue development	9.07484076	0	0	0	Skeletal Muscle Development	GJA1;SOX17;AKAP6;BMP4			
39	GO:0045932	negative regulation of muscle contraction	0	0	0	3.93526012	Skeletal Muscle Development				PTGS2;ADRA2C;ADRA2A;KCNJ2
40	GO:0048625	myoblast cell fate commitment	0	0	0	16.37079731	Skeletal Muscle Development				EPAS1;PITX1
41	GO:0048634	regulation of muscle organ development	3.94179035	0	3.9265794	3.04874723	Skeletal Muscle Development	AGRN;ERBB4;GJA1;HDAC9;IL6;TBX20;GATA4;SOX17;BHLHE41;EFNB2;AKAP6;BMP4;GATA6;BMP2;JPH2;TNFSF14		NKX2-5;HEY2;TBX5	MYOG;BOC;SHOX2;CXCL14;NRG1;COL14A1;MKX;RBP4;WNT10B;TBX3;SIX1;SIX4;GREM1;TBX2
42	GO:0048636	positive regulation of muscle organ development	9.07484076	0	0	0	Skeletal Muscle Development	GJA1;SOX17;AKAP6;BMP4			
43	GO:0048641	regulation of skeletal muscle tissue development	0	0	0	3.22308715	Skeletal Muscle Development				MYOG;BOC;SHOX2;CXCL14;MKX;WNT10B;TBX3;SIX1;SIX4
44	GO:0048644	muscle organ morphogenesis	0	0	0	4.1967683	Skeletal Muscle Development				TGFBR3;S1PR1;COL11A1;TNNT2;TNNI1;WNT5A;SHOX2;NRG1;GSC;ACTC1
45	GO:0048741	skeletal muscle fiber development	0	5.5849478	0	3.10047584	Skeletal Muscle Development		KY;MEF2C;COL4A5;ANK3		CACNA1S;MYOG;PDZRN3;BOC;SHOX2;CXCL14;TNC;MKX;WNT10B;TBX3;SIX1;SIX4;RCAN1
46	GO:0048742	regulation of skeletal muscle fiber development	0	0	0	3.72795086	Skeletal Muscle Development				MYOG;BOC;SHOX2;CXCL14;MKX;TBX3;SIX1;SIX4
47	GO:0048743	positive regulation of skeletal muscle fiber development	0	0	0	49.11815562	Skeletal Muscle Development				MYOG;SHOX2
48	GO:0048747	muscle fiber development	2.1669729	4.7758668	0	2.84748329	Skeletal Muscle Development	AGRN;HDAC9;KEL;DMD;BHLHE41;COL4A1;BMP4;WFIKKN2;TNFSF14;CXADR	KY;MEF2C;COL4A5;ANK3		CACNA1S;MYOG;PDZRN3;BOC;SHOX2;CXCL14;VEGFA;TNC;MKX;WNT10B;TBX3;SIX1;SIX4;RCAN1
49	GO:0051146	striated muscle cell differentiation	2.28933757	0	0	3.28979725	Skeletal Muscle Development	AGRN;SORT1;BTG2;TANC1;RARB;EDN1;HDAC9;KEL;DMD;GATA4;WT1;PTPLA;ANKRD1;BHLHE41;KRT8;EFNB2;COL4A1;AKAP6;BMP4;WFIKKN2;GATA6;BMP2;TNFSF14;CXADR			CASQ2;CACNA1S;TNNT2;MYOG;KCNH1;AGT;SOX11;SDC1;IGFBP5;XIRP1;PDZRN3;BOC;SHOX2;CXCL14;EGR1;PDGFRB;VEGFA;MET;NRG1;COL14A1;TNC;IGF2;MKX;EGR2;MYPN;WNT10B;TBX3;NOS1;SIX1;SIX4;FOS;GREM1;ACTC1;CACNA1H;MYH3;RCAN1
50	GO:0051147	regulation of muscle cell differentiation	0	0	0	3.10664464	Skeletal Muscle Development				MYOG;BOC;SHOX2;EREG;CXCL14;NRG1;COL14A1;MKX;TBX3;SIX1;SIX4;GREM1;CDH15;TSHZ3;RCAN1
51	GO:0051148	negative regulation of muscle cell differentiation	0	0	11.4679418	3.00095302	Skeletal Muscle Development			PRDM6;NKX2-5;HEY2	EREG;CXCL14;MKX;TBX3;RCAN1
52	GO:0051149	positive regulation of muscle cell differentiation	0	0	0	2.9249465	Skeletal Muscle Development				MYOG;BOC;SHOX2;NRG1;GREM1;CDH15;TSHZ3
53	GO:0051153	regulation of striated muscle cell differentiation	2.89332452	0	0	3.28171665	Skeletal Muscle Development	AGRN;EDN1;HDAC9;BHLHE41;EFNB2;AKAP6;BMP4;BMP2;TNFSF14			MYOG;BOC;SHOX2;CXCL14;NRG1;COL14A1;MKX;TBX3;SIX1;SIX4;GREM1
54	GO:0051155	positive regulation of striated muscle cell differentiation	0	0	0	4.09947013	Skeletal Muscle Development				MYOG;SHOX2;NRG1;GREM1
55	GO:0051451	myoblast migration	0	0	0	24.62572254	Skeletal Muscle Development				THBS4;NET1;SIX1;SIX4
56	GO:0055001	muscle cell development	2.30067315	3.9207921	0	3.45208071	Skeletal Muscle Development	AGRN;EDN1;HDAC9;KEL;DMD;GATA4;ANKRD1;BHLHE41;KRT8;COL4A1;AKAP6;BMP4;RAMP2;WFIKKN2;TNFSF14;CXADR	KY;MEF2C;COL4A5;ANK3;KRT19		CASQ2;CACNA1S;TNNT2;MYOG;AGT;SDC1;XIRP1;PDZRN3;BOC;SHOX2;CXCL14;PDGFRB;VEGFA;COL14A1;TNC;ADM;MKX;MYPN;WNT10B;TBX3;SIX1;SIX4;ACTC1;MYH3;RCAN1

57	GO:0055002	striated muscle cell development	2.21077876	4.6952663	0	3.62765501	Skeletal Muscle Development	AGRN;EDN1;HDAC9;KEL;DMD;ANKRD1;BHLHE41;KRT8;COL4A1;BMP4;WFIKKN2;TNFSF14;CXADR	KY;MEF2C;COL4A5;ANK3;KRT19	CASQ2;CACNA1S;TNNT2;MYOG;SDC1;XIRP1;PDZRN3;BOC;SHOX2;CXCL14;PDGFRB;VEGFA;TNC;MKX;MYPN;WNT10B;TBX3;SIX1;SIX4;ACTC1;MYH3;RCAN1
58	GO:0060415	muscle tissue morphogenesis	0	0	0	4.20004944	Skeletal Muscle Development			TGFBR3;S1PR1;COL11A1;TNNT2;TNNI1;WNT5A;SHOX2;NRG1;ACTC1
59	GO:0060537	muscle tissue development	2.51656315	0	0	3.65429363	Skeletal Muscle Development	AGRN;HSPG2;SORT1;BTG2;TGFB2;VAMP5;TANC1;ERBB4;RARB;GJA1;TCF21;HDAC9;TBX20;KEL;DMD;GATA4;SOX17;MSC;ZFPM2;WT1;PTPLA;ANKRD1;BHLHE41;PKP2;EFNB2;COL4A1;AKAP6;BMP4;ALDH1A2;GATA6;BMP2;JPH2;TNFSF14;CXADR		TGFBR3;S1PR1;COL11A1;CACNA1S;TNNT2;TNNI1;MYOG;KCNH1;HLX;AGT;SOX11;HOXD10;HOXD9;IGFBP5;XIRP1;WNT5A;PDZRN3;BOC;SHOX2;PITX1;CXCL14;EGR1;PDGFRB;VEGFA;COL19A1;MEOX2;ELN;CAV1;MET;NRG1;EYA1;COL14A1;TNC;MKX;EGR2;RBP4;WNT10B;TBX3;NOS1;SIX1;SIX4;FOS;GREM1;ACTC1;STRA6;CACNA1H;MYLPF;TBX2;EYA2;TSHZ3;RCAN1
60	GO:0060538	skeletal muscle organ development	2.19713628	0	0	3.60267738	Skeletal Muscle Development	AGRN;SORT1;BTG2;VAMP5;TANC1;TCF21;HDAC9;KEL;DMD;SOX17;MSC;ASS1;WT1;PTPLA;ANKRD1;BHLHE41;COL4A1;BMP4;TNFSF14		CACNA1S;MYOG;KCNH1;HLX;SOX11;HOXD10;HOXD9;IGFBP5;PDZRN3;BOC;SHOX2;PITX1;CXCL14;EGR1;COL19A1;MEOX2;ELN;CAV1;MET;TNC;MKX;EGR2;WNT10B;TBX3;NOS1;SIX1;SIX4;FOS;STRA6;CACNA1H;MYLPF;RCAN1
61	GO:0061061	muscle structure development	2.47012124	0	0	3.71024859	Skeletal Muscle Development	AGRN;WNT4;SORT1;BTG2;VAMP5;TANC1;ERBB4;RARB;EDN1;FAM65B;GJA1;TCF21;HDAC9;IL6;TBX20;KEL;DMD;FHL1;GATA4;SOX17;MSC;JPH1;ZFPM2;ASS1;WT1;TAGLN;PTPLA;ANKRD1;NTF3;BHLHE41;PKP2;KRT8;EFNB2;COL4A1;AKAP6;BMP4;ITGA11;RAMP2;WFIKKN2;GATA6;BMP2;JPH2;LAMA5;TNFSF14;CXADR		TGFBR3;S1PR1;COL11A1;CASQ2;CACNA1S;TNNT2;TNNI1;MYOG;KCNH1;HLX;AGT;SOX11;SDC1;EPAS1;HOXD10;HOXD9;IGFBP5;COL6A3;XIRP1;WNT5A;PDZRN3;BOC;PPP2R3A;SHOX2;EREG;PITX1;CXCL14;EGR1;PDGFRB;VEGFA;COL19A1;LAMA2;MEOX2;ELN;CAV1;MET;EGR3;NRG1;COL14A1;TNC;IGF2;ADM;ALX4;MKX;EGR2;MYPN;RBP4;WNT10B;SCN8A;TBX3;NOS1;SGCG;EDNRB;SIX1;SIX4;FOS;GSC;GREM1;ACTC1;RORA;STRA6;CACNA1H;MYLPF;CDH15;MYH3;TBX2;TSHZ3;WNT7B;RCAN1
62	GO:0090257	regulation of muscle system process	2.17363811	0	0	3.09429356	Skeletal Muscle Development	GJA5;FKBP1B;OXTR;EDN1;GJA1;CTGF;CAMK2B;PIK3CG;DMD;MRVI1;AKAP6;ADORA2B		CASQ2;PTGS2;TNNT2;TNNI1;MYOG;MYBPH;AGT;CHRM3;ADRA2C;CAV1;COL14A1;TNNT3;SCN2B;ADRA2A;CACNA1C;NOS1;CACNA1G;KCNJ2
1	GO:0000187	activation of MAPK activity	0	0	0	2.07337159	General Terms			ALK;TGFA;WNT5A;ADRA2C;EPGN;CD74;GRM4;MET;ADRA2A;FPR1;WNT7B
2	GO:0001508	regulation of action potential	1.92099543	5.4047619	0	2.11842105	General Terms	CXCR4;RARB;TNFRSF21;KEL;MYRF;NTF3;CD9;KCNMB4;AKAP6;MBP	SCN9A;SCN5A;GRIK2;KCND2;ANK3	NFASC;SCN2A;UGT8;LAMA2;TAC1;DPP6;CLU;NDRG1;EGR2;SCN8A;CACNA1G;EPB41L3
2	GO:0001706	endoderm formation	5.96358171	0	0	0	General Terms	ITGA4;COL8A1;TBX20;GATA4;SOX17;COL4A2;MMP15;VTN;LHX1;GATA6		
3	GO:0007223	Wnt receptor signaling pathway, calcium modulating pathway	0	77.2982456	0	49.11815562	General Terms		FZD4	WNT5A;ROR2
3	GO:0007492	endoderm development	3.91341295	0	0	2.74111757	General Terms	ITGA4;COL8A1;TBX20;GATA4;SOX17;COL4A2;MMP15;VTN;LHX1;GATA6		COL11A1;INHBA;ARC;MMP8;PTHLH;HOXC11;MMP9;COL6A1
4	GO:0007493	endodermal cell fate determination	Inf	0	0	0	General Terms	SOX17;GATA6		

4	GO:0008593	regulation of Notch signaling pathway	0	0	13.5402387	0	General Terms			HEY2;EGFL7;NRARP;JAG1	
5	GO:0010990	regulation of SMAD protein complex assembly	18.1015873	0	0	0	General Terms	TBX20;SMAD6			
5	GO:0010991	negative regulation of SMAD protein complex assembly	18.1015873	0	0	0	General Terms	TBX20;SMAD6			
6	GO:0016055	Wnt receptor signaling pathway	2.10714931	2.7410306	2.8736422	2.15642561	General Terms	WNT4;VAX2;FZD5;WNT7A;TNIK;NKD2;MCC;ANKRD6;GATA4;SOX17;FZD6;GATA3;BAMBI;BICC1;GRK5;CAPRIN2;FOXO1;NDRG2;GPRC5B;CDH1;JUP;ITGA3;SLC9A3R1;BMP2;SULF2	VANGL2;MLLT3;FZD4;LGR5;SOST;CELSR1	NKX2-5;RSPO3;TLE1;NRARP;HHEX;AMER2	PTPRU;TRABD2B;PPAP2B;WNT9A;SDC1;TRABD2A;WNT5A;PPP2R3A;CPZ;SFRP2;EGR1;FZD1;DLX5;CAV1;SNAI2;RSPO2;ROR2;WNT11;WNT5B;WNT10B;WIF1;GSC;GREM1;CDH3;NXN;APCDD1;RSPO4;WNT7B
6	GO:0019722	calcium-mediated signaling	2.66421558	0	0	2.65241228	General Terms	SELE;FKBP1B;CXCR4;CXCL8;TENM2;EDN1;GPR143;DMD;DMTN;AKAP6;CDH13			PTGFR;CASQ2;AGTR1;MCTP1;RCAN2;CD24;BHLHA15;NCALD;CACNA1C;KSR2;HOMER2;RCAN1
7	GO:0030111	regulation of Wnt receptor signaling pathway	2.40547421	0	3.7860404	2.44242372	General Terms	WNT4;FZD5;WNT7A;NKD2;MCC;ANKRD6;SOX17;FZD6;BAMBI;BICC1;CAPRIN2;FOXO1;GPRC5B;CDH1;JUP;ITGA3;BMP2;SULF2		NKX2-5;TLE1;NRARP;HHEX;AMER2	TRABD2B;PPAP2B;TRABD2A;WNT5A;PPP2R3A;SFRP2;EGR1;FZD1;DLX5;CAV1;SNAI2;RSPO2;ROR2;WNT11;WNT5B;WNT10B;GSC;GREM1;NXN;APCDD1
7	GO:0030177	positive regulation of Wnt receptor signaling pathway	3.11416787	0	0	0	General Terms	WNT4;WNT7A;ANKRD6;BAMBI;CAPRIN2;GPRC5B;JUP;BMP2;SULF2			
8	GO:0030178	negative regulation of Wnt receptor signaling pathway	2.33489983	0	3.9584715	3.58961314	General Terms	WNT4;NKD2;MCC;ANKRD6;SOX17;FZD6;BICC1;FOXO1;CDH1;BMP2		NKX2-5;TLE1;AMER2	TRABD2B;TRABD2A;WNT5A;PPP2R3A;SFRP2;EGR1;FZD1;CAV1;SNAI2;ROR2;WNT11;WNT5B;GSC;GREM1;NXN;APCDD1
8	GO:0030509	BMP signaling pathway	3.02405465	0	0	3.46393862	General Terms	GDF7;FST;BMPER;GATA4;GDF6;GDF2;C10orf54;BMP4;SMAD6;PCSK6;ITGA3;BMP2;FSTL3			TGFBR3;GREM2;SOX11;WNT5A;BMPR1B;SFRP2;RGMB;EGR1;FZD1;DLX5;CAV1;CHRDL1;HTRA1;SMAD9;GREM1;RGMA
9	GO:0030513	positive regulation of BMP signaling pathway	4.18495835	0	0	0	General Terms	GATA4;GDF2;C10orf54;BMP4			
9	GO:0032318	regulation of Ras GTPase activity	1.60216718	4.1546047	0	0	General Terms	AGRN;ARHGEF16;WNT4;EVI5;ASAP2;FGD5;ALS2CL;ARHGEF3;ARHGEF26;EPHA5;SPRY1;ARHGEF28;RASGRF2;ARHGEF37;ADAP1;CCL26;OPHN1;EPS8L2;NTF3;ICAM1	TBC1D8;ARAP2;EFNA5;PLEKHG1;DOCK4;DENND3;FGD4;PECAM1;RASAL3		
10	GO:0032905	transforming growth factor beta1 production	9.04920635	0	0	0	General Terms	CD2AP;GATA6			
10	GO:0032906	transforming growth factor beta2 production	9.04920635	0	0	8.18395773	General Terms	TGFB2;GATA6			WNT11;CDH3
11	GO:0032908	regulation of transforming growth factor beta1 production	9.04920635	0	0	0	General Terms	CD2AP;GATA6			
11	GO:0032909	regulation of transforming growth factor beta2 production	9.04920635	0	0	8.18395773	General Terms	TGFB2;GATA6			WNT11;CDH3
12	GO:0035332	positive regulation of hippo signaling cascade	0	0	0	Inf	General Terms				SOX11
12	GO:0035567	non-canonical Wnt receptor signaling pathway	0	17.6076076	0	3.51664255	General Terms		VANGL2;MLLT3;FZD4;CELSR1		WNT5A;SFRP2;FZD1;ROR2;WNT11
13	GO:0035987	endodermal cell differentiation	4.66247805	0	0	2.86103726	General Terms	ITGA4;COL8A1;SOX17;COL4A2;MMP15;VTN;GATA6			COL11A1;INHBA;MMP8;MMP9;COL6A1
13	GO:0043266	regulation of potassium ion transport	0	13.5459792	0	6.15955137	General Terms		KCNE3;ANK3		CASQ2;DPP6;ADRA2A;NOS1;ADCYAP1
14	GO:0043405	regulation of MAP kinase activity	2.67948579	4.0594665	0	1.88060337	General Terms	EFNA1;IL1B;CXCR4;FZD5;MST1R;KIT;SPRY1;EDN1;TPD52L1;PDGFA;PIK3CG;DUSP4;LYN;RGS3;TLR4;DUSP8;ERCC6;NTF3;IRAK3;BMP4;ADORA2B;TNFRSF11A;BMP2;APOE;PDGFB	LPAR3;VANGL2;RGS4;PDE5A;MEF2C;FZD4;SORL1		ALK;TGFA;DUSP2;WNT5A;ADRA2C;EPGN;SFRP2;PDGFRB;CD74;TNXB;GRM4;VEGFA;CAV1;MET;TENM1;DUSP5;ADRA2A;KITLG;FPR1;WNT7B

14	GO:0043406	positive regulation of MAP kinase activity	2.46147197	3.9207921	0	1.93339299	General Terms	EFNA1;IL1B;CXCR4;FZD5;MST1R;KIT;EDN1;TPD52L1;PDGFA;PIK3CG;TLR4;ERCC6;NTF3;ADORA2B;TNFRSF11A;BMP2;PDGFB	LPAR3;VANGL2;PDE5A;MEF2C;FZD4		ALK;TGFA;WNT5A;ADRA2C;EPGN;PDGFRB;CD74;GRM4;VEGFA;MET;TENM1;ADRA2A;KITLG;FPR1;WNT7B
15	GO:0043407	negative regulation of MAP kinase activity	3.73041004	0	0	0	General Terms	IL1B;SPRY1;DUSP4;LYN;RGS3;DUSP8;IRAK3;BMP4;APOE			
15	GO:0043408	regulation of MAPKKK cascade	2.28037511	2.6814014	0	1.48997494	General Terms	TGFB2;IL1B;FZD5;ERBB4;ACKR3;WNT7A;MECOM;KIT;SPRY1;TLR3;EDN1;TPD52L1;CTGF;PDGFA;IL6;BMPER;DUSP4;LYN;RGS3;TLR4;DUSP8;ARRB1;ERCC6;FOXO1;NDRG2;BMP4;KSR1;SLC9A3R1;TNFRSF11A;BMP2;ICAM1;PDGFB	LPAR3;VANGL2;RGS4;GRIK2;AR;FZD4;PELI2		CHI3L1;AGT;DUSP2;WNT5A;EPHB1;EDNRA;SFRP2;PDGFRB;CD74;WWC1;FGF18;TNXB;GRM4;VEGFA;EPHA7;CAV1;RPS6KA6;ANGPT1;IGF2;DUSP5;KSR2;SEMA7A;ADCYAP1;WNT7B
16	GO:0043409	negative regulation of MAPKKK cascade	2.91013888	0	0	0	General Terms	MECOM;SPRY1;DUSP4;LYN;RGS3;TLR4;DUSP8;FOXO1;NDRG2;SLC9A3R1			
16	GO:0043410	positive regulation of MAPKKK cascade	2.47457428	0	0	1.74324955	General Terms	TGFB2;IL1B;ERBB4;ACKR3;WNT7A;KIT;TLR3;TPD52L1;CTGF;PDGFA;IL6;BMPER;TLR4;ARRB1;BMP4;KSR1;TNFRSF11A;BMP2;ICAM1;PDGFB			CHI3L1;AGT;WNT5A;EDNRA;PDGFRB;CD74;WWC1;FGF18;GRM4;VEGFA;ANGPT1;IGF2;KSR2;SEMA7A;ADCYAP1;WNT7B
17	GO:0044328	canonical Wnt receptor signaling pathway involved in positive regulation of endothelial cell migration	0	0	0	Inf	General Terms				PPAP2B
17	GO:0044329	canonical Wnt receptor signaling pathway involved in positive regulation of cell-cell adhesion	0	0	0	Inf	General Terms				PPAP2B
18	GO:0045165	cell fate commitment	1.97630564	0	3.3377496	2.87506082	General Terms	WNT4;TAL1;TGFB2;GDF7;FZD5;ERBB4;SPRY1;IL6;GATA4;SOX17;WT1;GATA3;NTF3;BMP4;JAG2;GATA6;BMP2		KDR;NKX2-5;TFAP2A;HEY2;JAG1	LMO4;MYOG;SIX2;EPAS1;DLX2;HOXD11;HOXD10;SFRP2;GDNF;PITX1;IRF4;PRDM1;HOXA11;EBF2;NRG1;EYA1;GAS1;SOX5;HOXC11;HOXC10;TBX3;SIX1;GSC;BCL11B;TBX2;EYA2
18	GO:0045597	positive regulation of cell differentiation	1.97703775	0	3.1003643	2.55221239	General Terms	WNT4;LIN28A;ZC3H12A;TAL1;TACSTD2;TGFB2;GDF7;FKBP1B;PKDCC;CXCR4;SERPINE2;RARB;KIT;CSF2;GJA1;CTGF;IL6;RARRES2;DMD;GATA4;SOX17;LYN;GDF6;CDKN2B;IL15RA;GATA3;BAMBI;GDF2;C10orf54;NTF3;CAPRIN2;EFNB2;AKAP6;BMP4;GPRC5B;LHX1;STAT5A;GATA6;BMP2;TNFSF14;APOE		EPHB2;TNFSF4;NKX2-5;GCNT2;NRCAM;FEZ1;ACVRL1;TBX5;MEDAG;HTR2A;JAG1	DAB1;NGF;BRINP2;PTGS2;MYOG;HLX;WNT9A;AGT;SOX11;HOXD11;BOC;AGTR1;SHOX2;ADRA2C;PROM1;PF4;BMPR1B;SFRP2;IL7R;GDNF;CD74;FGF18;VEGFA;PRDM1;MYB;HOXA11;INHBA;DLX5;NAP1L2;EGR3;NEFL;NRG1;SNAI2;BRINP1;WNT5B;SOX5;VDR;WNT10B;WIF1;KITLG;SMAD9;FOS;GREM1;SEMA7A;RLTPR;CDH15;SERPINF1;CSF3;NGFR;SOCS3;CEBPB;TNFSF9;TSHZ3;CEBPA;WNT7B;DSCAM
19	GO:0045746	negative regulation of Notch signaling pathway	0	0	24.6883178	0	General Terms			HEY2;EGFL7;NRARP	
19	GO:0046578	regulation of Ras protein signal transduction	1.5878884	3.3954366	0	0	General Terms	AGRN;ARHGEF16;WNT4;EVI5;ASAP2;FGD5;ALS2CL;ARHGEF3;ARHGEF26;EPHA5;SPRY1;ARHGEF28;RASGRF2;ARHGEF37;ADAP1;CCL26;OPHN1;EPS8L2;ARRB1;APOA1;NTF3;ITGA3;ICAM1;APOE	TBC1D8;ARAP2;EFNA5;PLEKHG1;DOCK4;DENND3;FGD4;PECAM1;RASAL3		
20	GO:0048333	mesodermal cell differentiation	0	0	0	3.73021094	General Terms				SIX2;SFRP2;HOXA11;INHBA;EYA2
20	GO:0048638	regulation of developmental growth	2.11620429	0	4.890566	3.71330359	General Terms	AGRN;ERBB4;GJA1;TBX20;GATA4;WT1;AKAP6;BMP4;GATA6;APOE		NKX2-5;HEY2;NRCAM;TBX5	NGF;WNT5A;MGLL;PPP2R3A;SPP1;SFRP2;VEGFA;PLXNA4;NRK;NRG1;COL14A1;RBP4;WNT10B;SIX1;SIX4;SEMA7A;TBX2;DSCAM

21	GO:0051924	regulation of calcium ion transport	2.06329884	0	0	2.38464362	General Terms	FKBP1B;GJA1;CAMK2B;DMD;LYN;JPH1;AKAP6;CEMIP;CCL2;JPH2;ICAM1;PDGFB			NGF;CASQ2;PTGS2;AGT;EDNRA;PDE4D;PDGFRB;CAMK2A;CAV1;STC1;ADRA2A;CACNA1C;NOS1;BDKRB1;MCHR1
21	GO:0060316	positive regulation of ryanodine-sensitive calcium-release channel activity	7.75600907	0	0	0	General Terms	AKAP6;JPH2			
22	GO:0060389	pathway-restricted SMAD protein phosphorylation	5.33708568	0	0	2.79584923	General Terms	TGFB2;GDF7;BMPER;GDF6;GDF2;BMP4;SMAD6;BMP2			TGFBR3;BMP5;INHBA;GREM1;LDLRAD4
22	GO:0060393	regulation of pathway-restricted SMAD protein phosphorylation	5.03145263	0	0	0	General Terms	GDF7;BMPER;GDF6;GDF2;BMP4;SMAD6;BMP2			
23	GO:0060804	positive regulation of Wnt receptor signaling pathway by BMP signaling pathway	Inf	0	0	0	General Terms	BMP2			
23	GO:0060828	regulation of canonical Wnt receptor signaling pathway	3.03878191	0	0	2.3597263	General Terms	WNT4;FZD5;WNT7A;NKD2;MCC;ANKRD6;SOX17;FZD6;BAMBI;BICC1;CAPRIN2;FOXO1;GPRC5B;CDH1;JUP;BMP2			WNT5A;PPP2R3A;SFRP2;EGR1;FZD1;DLX5;CAV1;SNAI2;RSPO2;ROR2;WNT11;WNT5B;WNT10B;GREM1
24	GO:0070371	ERK1 and ERK2 cascade	2.85325171	0	0	1.79341878	General Terms	ERBB4;ACKR3;OXTR;SPRY1;CTGF;PDGFA;IL6;BMPER;LYN;TLR4;ARRB1;NDRG2;BMP4;SLC9A3R1;TNFRSF11A;BMP2;ICAM1;PDGFB			CHI3L1;AGT;EPHB1;EDNRA;PDGFRB;CD74;FGF18;VEGFA;EPHA7;RPS6KA6;ANGPT1;SEMA7A;ADCYAP1
24	GO:0070372	regulation of ERK1 and ERK2 cascade	2.89168925	0	0	1.77308541	General Terms	ERBB4;ACKR3;SPRY1;CTGF;PDGFA;IL6;BMPER;LYN;TLR4;ARRB1;NDRG2;BMP4;SLC9A3R1;TNFRSF11A;BMP2;ICAM1;PDGFB			CHI3L1;EPHB1;EDNRA;PDGFRB;CD74;FGF18;VEGFA;EPHA7;RPS6KA6;ANGPT1;SEMA7A;ADCYAP1
25	GO:0070373	negative regulation of ERK1 and ERK2 cascade	4.25563198	0	0	0	General Terms	SPRY1;LYN;TLR4;NDRG2;SLC9A3R1			
25	GO:0070374	positive regulation of ERK1 and ERK2 cascade	3.02405465	0	3.8338931	0	General Terms	ERBB4;ACKR3;CTGF;PDGFA;IL6;BMPER;TLR4;ARRB1;BMP4;TNFRSF11A;BMP2;ICAM1;PDGFB		KDR;GCNT2;HTR2A	
26	GO:0071604	transforming growth factor beta production	6.40389659	0	0	5.78986739	General Terms	CD34;TGFB2;CD2AP;GATA6			PTGS2;WNT11;CDH3;SERPINB7
26	GO:0071634	regulation of transforming growth factor beta production	6.40389659	0	0	5.78986739	General Terms	CD34;TGFB2;CD2AP;GATA6			PTGS2;WNT11;CDH3;SERPINB7
27	GO:0071636	positive regulation of transforming growth factor beta production	0	0	0	7.37445887	General Terms				PTGS2;WNT11;SERPINB7
27	GO:0071804	cellular potassium ion transport	0	4.2265927	0	2.91417269	General Terms		KCNK3;KCND2;KCNE3		KCNK2;KCNK1;KCNE4;KCNK5;KCNQ3;KCNMA1;KCNA1;ABCC9;KCNJ2;KCNN1;KCNJ6;KCNJ15
28	GO:0071805	potassium ion transmembrane transport	0	4.2265927	0	2.91417269	General Terms		KCNK3;KCND2;KCNE3		KCNK2;KCNK1;KCNE4;KCNK5;KCNQ3;KCNMA1;KCNA1;ABCC9;KCNJ2;KCNN1;KCNJ6;KCNJ15
28	GO:0072091	regulation of stem cell proliferation	0	0	0	6.56262042	General Terms				NGF;SOX11;SNAI2;TBX3
29	GO:0090090	negative regulation of canonical Wnt receptor signaling pathway	3.21997352	0	0	3.24245742	General Terms	WNT4;NKD2;MCC;ANKRD6;SOX17;FZD6;BICC1;FOXO1;CDH1;BMP2			WNT5A;PPP2R3A;SFRP2;EGR1;FZD1;CAV1;SNAI2;ROR2;WNT11;WNT5B;GREM1
29	GO:0090263	positive regulation of canonical Wnt receptor signaling pathway	3.08445355	0	0	3.32005694	General Terms	WNT4;WNT7A;BAMBI;CAPRIN2;GPRC5B;JUP			PPP2R3A;SFRP2;DLX5;CAV1;RSPO2;ROR2;WNT10B
30	GO:2000035	regulation of stem cell division	0	0	0	24.55763689	General Terms				SFRP2;NAP1L2
31	GO:2000648	positive regulation of stem cell proliferation	0	0	0	8.19432419	General Terms				NGF;SOX11;TBX3
