# Supplementary material for: Human fetal mesoangioblasts reveal tissue‐dependent transcriptional signatures
Source: Stem Cells Transl Med. 2020 Jan 23;9(5):575–89. doi: 10.1002/sctm.19-0209 (PMC7180296; doi:10.1002/sctm.19-0209)
Supplement: Supplementary file 10 — Table S4 Interaction report of up‐ and down‐regulated genes in V‐ versus Sk‐MABs (as depicted in Figure 7). [file SCT3-9-575-s010.pdf]

Supplementary Table 4. Interaction report of up- and down-regulated genes in V- versus Sk-MABs, as depicted in Fig. 7.

	Interaction Report		Transcriptional regulation	Log FC	p-value
Non interacting	Activin A			3.677	2.57467E-19
	FGF12			5.000	0.000278128
	Isl1			5.050	0.000335804
	L-type Ca^{2+} channel, α1C			4.485	3.94807E-15
	Myoglobin			10.628	1.53585E-05
	MSX-2			2.040	0.015490649
	SALL4			2.315	0.007016532
	DHA2			2.509	2.23433E-05
	PDLIM3			4.679	2.52278E-37
	Mixl1			4.039	0.005405008
	SMAD6			1.703	3.90722E-08
	SOX6			2.125	0.022154846
	MyHC			3.607	0.003855508
Interacting	Cardiac Troponin C	PAX3	Activation	3.607	0.003855508
	MYOD	SLUG	Activation	0.626	2.7879E-07
		ACTC	Activation	2.286	0.000699196
	PAX3	TBX2	Activation	2.471	1.01988E-14
	TBX2	MYOD	Activation	3.334	2.22384E-20
	CRP3 (MLP)	Phospholamban	Activation	4.448	2.41106E-35
	Phospholamban	TNNC1	Activation	10.061	0.042439732
	PCAM1	VE-cadherin	Activation	2.475	2.10996E-16
	GATA4	Hand2	Activation	4.028	7.40488E-11
		MYOD	Activation	2.607	1.15622E-06
		ACTC	Activation	5.304	0.000961133
	PCAF	MYOD	Activation	4.441	0.000486783
		E2F1	Activation	5.937	2.50151E-13
	TGFβ2	PAX3	Activation	4.133	9.47051E-35
	BMP 4	BMPR1B	Activation	4.984	3.4984E-46
		PAX3	Inhibition	-2.216	2.11628E-07

Upregulated in Sk

Down-regulated in Sk

FC : fold-change
